# Synthesis of *N*‐Heterocyclic Carbenes and Their Complexes by Chloronium Ion Abstraction from 2‐Chloroazolium Salts Using Electron‐Rich Phosphines

**DOI:** 10.1002/anie.202202190

**Published:** 2022-05-17

**Authors:** Matthias D. Böhme, Tobias Eder, Maike B. Röthel, Patrick D. Dutschke, Lukas F. B. Wilm, F. Ekkehardt Hahn, Fabian Dielmann

**Affiliations:** ^1^ Institut für Anorganische und Analytische Chemie Westfälische Wilhelms-Universität Münster Corrensstraße 39 48149 Münster Germany; ^2^ Institute of General and Theoretical Chemistry Leopold-Franzens Universität Innsbruck Innrain 80–82 6020 Innsbruck Austria

**Keywords:** Chloronium Ion Abstraction, Electron-Rich Phosphines, Heterobimetallic Complexes, N-Heterocyclic Carbenes, Oxidative Addition

## Abstract

*N*‐Heterocyclic carbenes (NHCs) are commonly prepared by deprotonation of azolium salts using strong anionic bases. This reaction is often unselective, yielding alkali metal NHC complexes or dimerized NHCs. Alternatively, free NHCs are obtained by the dechlorination of 2‐chloroazolium salts using electron‐rich phosphines. PPh_3_, PCy_3_, and P*t*Bu_3_ are unsuitable for Cl^+^ abstraction, while the sterically encumbered tris(1,3‐*tert*‐butylimidazolidin‐2‐ylidenamino)phosphine **1** selectively removes Cl^+^ from 2‐chloroazolium salts. Since bulky **1** does not bind to metal complexes, it was used for the preparation of NHC complexes via in situ Cl^+^ abstraction from 2‐chloroazolium salts. The dechlorination was employed for the site‐selective monometallation with Ir^I^, Ir^III^, Rh^I^, Rh^III^, and Ru^II^ of a bis‐NHC precursor composed of a 2‐chlorobenzimidazolium and a 2‐chlorobenzimidazole group, followed by the preparation of the heterobimetallic Ir^III^/Pd^II^ complex [**18**](BF_4_)_2_ by a dechlorination/oxidative addition reaction sequence.

## Graphical abstract

Chloronium ion abstraction from 2‐chloroimidazolium salts using tris(1,3‐*tert*‐butylimidazolin‐2‐ylidenamino)phosphine **1** rapidly affords the free *N*‐heterocyclic carbenes (NHCs) and chlorophosphonium ions (**1**‐Cl)^+^. The dechlorination can be used for the site‐selective metallation of unsymmetric 2‐chloroazole/2‐chloroazolium bis‐NHC precursors by phosphine‐mediated chloronium ion abstraction followed by an oxidative addition reaction to give heterobimetallic complexes.

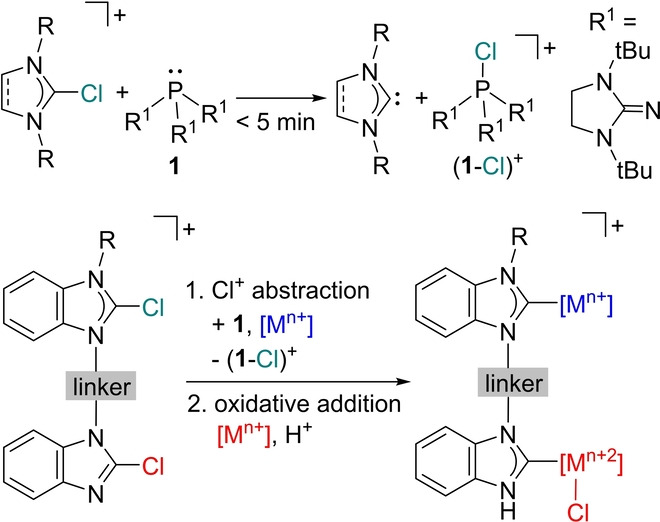

## Introduction

*N*‐Heterocyclic carbenes (NHCs) have emerged as important compounds in synthetic chemistry^[1]^ with profound impact on organic synthesis,^[2]^ coordination chemistry and catalysis,[^1^, ^3^] and for the preparation of luminescent materials^[4]^ and metallosupramolecular assemblies.^[5]^ While free NHCs are normally obtained by deprotonation of easily accessible azolium salts[^1^, ^3^] or by the reductive desulfurization of azol‐2‐thiones,^[6]^ a number of alternative methods for the preparation of NHC complexes have been described.^[1]^

The most common procedure for the preparation of metal‐NHC complexes is based on a ligand exchange reaction and therefore depends on whether or not the required NHCs are accessible as discrete species.[^1^, ^3^] Consequently, isolable five‐membered ring NHCs are most commonly used as ancillary ligands in NHC complexes, while transient carbenes have been used less frequently in spite of their promising chemical and physical properties. To exploit the full potential of transient carbenes such as imidazolidin‐2‐ylidenes or benzimidazolin‐2‐ylidenes bearing less sterically demanding *N*,*N*′‐substituents as ligands,^[6b]^ it is necessary to develop practical alternative methods for their incorporation into metal complexes.

A first step in this direction was the development of the generally applicable Ag_2_O route where Ag^I^‐NHC complexes are prepared from azolium salts and Ag_2_O with subsequent transmetallation of the NHC ligand to other transition metals.^[7]^ Since there is no need to isolate the free NHC, this method gives access to complexes bearing NHCs that cannot be isolated as stable free species.

Reductive methods starting from azolium salts have also been described. Cavell demonstrated the formation of NHC complexes by the oxidative addition of the C2−H or C2−X (X=halogen) bond of azolium salts to low‐valent transition metals.^[8]^ This method was later extended to neutral 2‐halogeno‐*N*‐alkylazoles^[9]^ and unsubstituted 2‐halogenoazoles^[10]^ giving access to complexes bearing protic NH,NR‐ and NH,NH‐NHC ligands, which are unstable when not bound to a metal center.^[11]^ Even complexes of the freely unknown CAAC ligands with *N*‐alkyl substituents have been prepared by oxidative addition.^[12]^ However, the oxidative addition strategy only works for a narrow range of metals in low oxidation states leading to NHC complexes where the metal oxidation state is increased by +2.

**Scheme 1:**
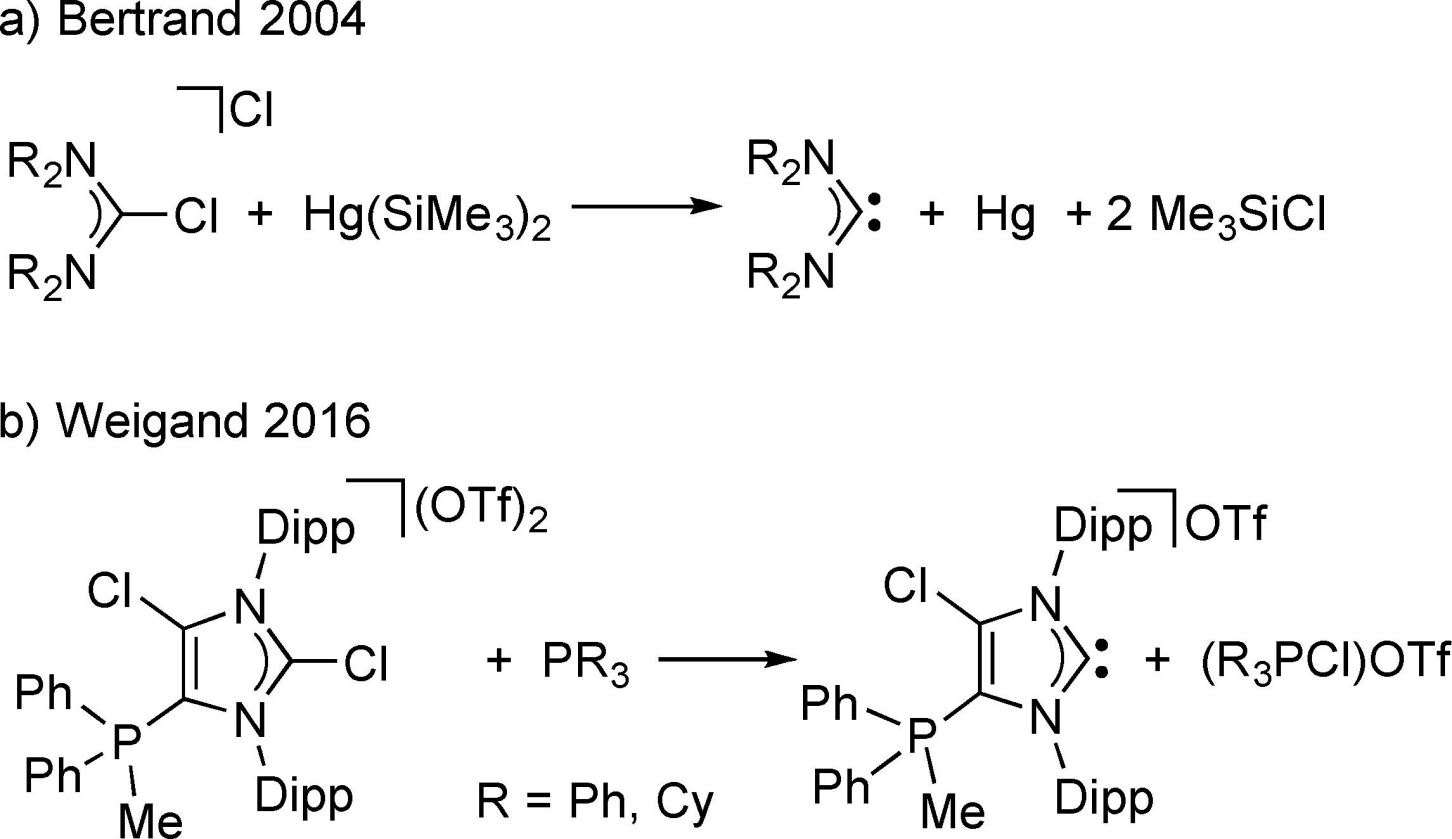
Reported chloronium ion abstractions for the generation of carbenes.

An alternative reductive activation was introduced by Bertrand who used Hg(SiMe_3_)_2_ for the dechlorination of 2‐chloroformamidinium salts under mild conditions (Scheme 1a).^[13]^ However, the method suffers from the need to handle toxic mercury reagents. Weigand showed that a cationic 5‐phosphonio‐substituted NHC can be generated by chloronium ion transfer to PCy_3_ or PPh_3_ (Scheme 1b).^[14]^ In addition, some late transition metal complexes were prepared in a one‐pot procedure using the cationic NHC generated by Cl^+^ ion abstraction. However, this study is limited to one C4‐Cl/C5‐phosphonio‐substituted, electron deficient, cationic imidazolin‐2‐ylidene with low basicity obtained from a dicationic imidazolium precursor.

Indeed, the abstraction of chloronium ions from 2‐chloroazolium salts with phosphines can only become a general method for the generation of NHCs, if the chloronium ion affinity of the used phosphine is higher than that of the NHC generated. Steric shielding of the phosphine is required to prevent the irreversible formation of imidazolio‐phosphonium adducts.^[15]^ Herein, we report a general, metal‐free route to NHCs by dechlorination of various 2‐chloroazolium salts, including C4/C5‐unsubstituted derivatives, using sterically encumbered, electron‐rich phosphines. This procedure constitutes a new approach for the generation of NHCs and enables the site‐selective metallation of suitable poly‐NHC precursors with different metals.

## Results and Discussion

**Scheme 2:**
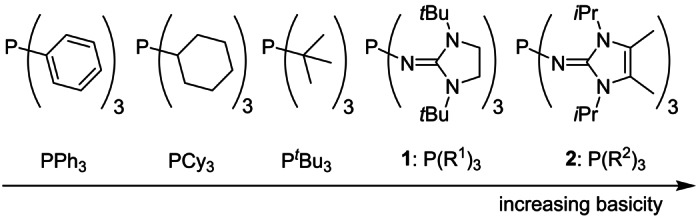
Electron‐rich phosphines used in this study.

To test the possibility of the chloronium ion abstraction by phosphines, the compounds shown in Scheme 2 were investigated. In addition to the commercially available phosphines PPh_3_, PCy_3_, and P*t*Bu_3_, two particularly electron‐rich imidazolin‐2‐ylidenaminophosphines (IAPs) **1** and **2** were included. IAP **2** has been described previously as one of the strongest non‐ionic superbases (p*K*_BH_^+^(MeCN)=38.8)^[16]^ and is therefore expected to be highly reactive towards azolium C2−Cl bonds. The new phosphine **1** was developed to increase the selectivity of the chloronium ion abstraction. It contains three bulky 1,3‐di‐*tert*‐butylimidazolidin‐2‐ylidenamino substituents (R^1^) which should allow the generation of highly nucleophilic carbenes by restricting access to the phosphorus center and thus preventing the formation of carbene adducts.

### Synthesis of IAP 1

**Scheme 3:**
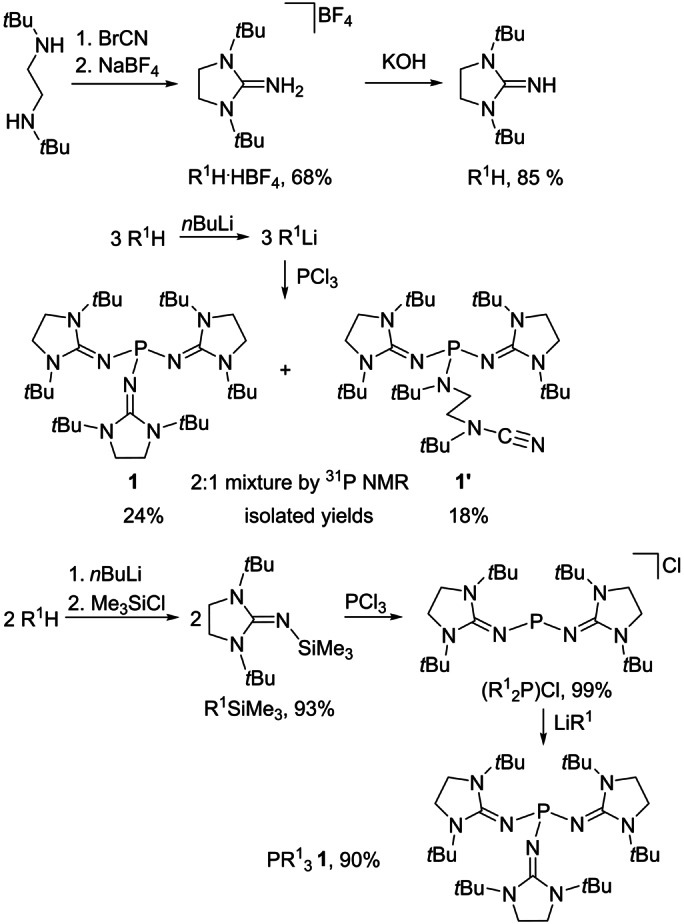
Synthesis of phosphines **1** and **1′**.

**Figure 1:**
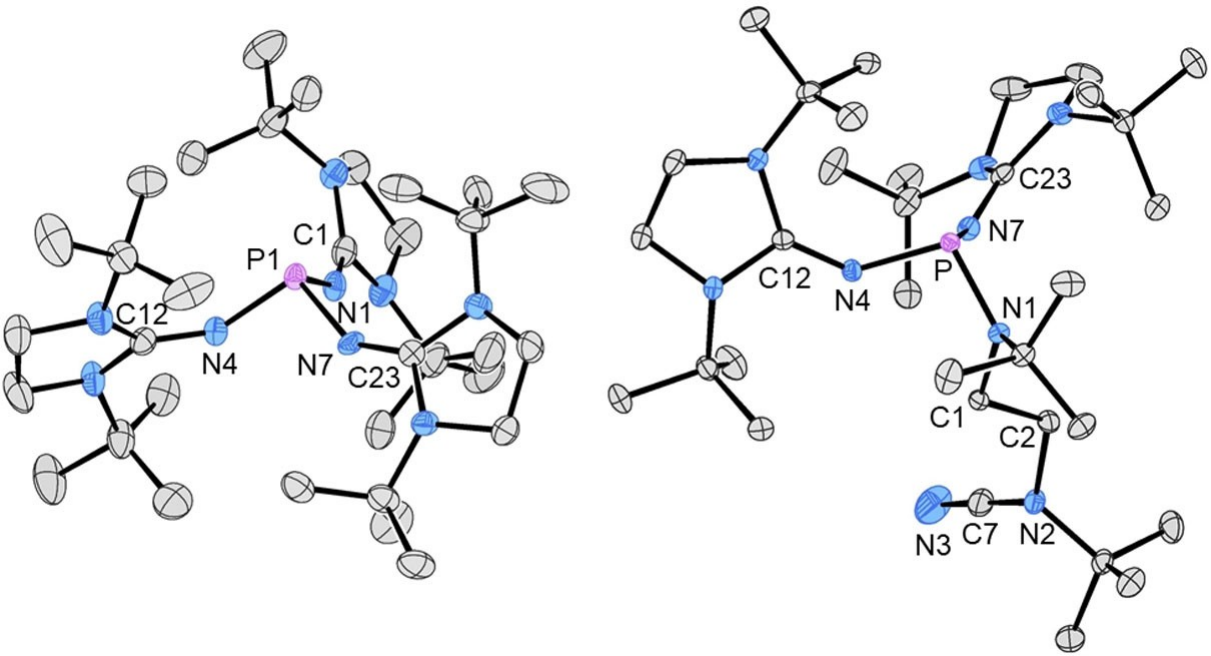
Molecular structures of **1** (left) and **1′** (right). Hydrogen atoms are omitted for clarity and thermal ellipsoids are set at 50 % probability. The PN_3_ unit of **1** is positionally disordered and only one of the two essentially identical orientations of the PN_3_ unit is depicted (see the Supporting Information). Selected bond lengths [Å] and angles [°] for **1** (bond parameters for one of the two PN_3_ orientations): P1–N1 1.699(7), P1–N4 1.680(7), P1–N7 1.700(12), N1–C1 1.213(8), N4–C12 1.312(7), N7–C23 1.260(14); N1‐P1‐N4 97.4(3), N1‐P1‐N7 98.5(5), N4‐P1‐N7 98.9(4), P1‐N1‐C1 143.0(4), P1‐N4‐C12 143.3(4), P1‐N7‐C23 145.0(6). Selected bond lengths [Å] and angles [°] for **1′**: P–N1 1.7168(11), P–N4 1.7020(11), P–N7 1.6859(12), N1–C1 1.464(2), N4–C12 1.280(2), N7–C23 1.279(2), N2–C7 1.326(2), N3–C7 1.153(2); N1‐P‐N4 102.83(5), N1‐P‐N7 98.78(5), N4‐P‐N7 97.92(6), P‐N1‐C1 118.77(8), P‐N4‐C12 136.68(10), P‐N7‐C23 136.36(11), N2‐C7‐N3 178.6(2).

Based on a published procedure,^[17]^ the *N*‐heterocyclic imine R^1^H was synthesized by the reaction of *N*,*N*′‐*tert*‐butylethylenediamine with cyanogen bromide followed by deprotonation with KOH in 58 % overall yield (Scheme 3, top). The synthesis of phosphine **1** from PCl_3_ and three equivs. of LiR^1^ gave a 2 : 1 mixture of phosphines **1** and **1′**, from which the desired phosphine **1** was isolated after workup in only 24 % yield (Scheme 3, center; see the Supporting Information). Phosphine **1′** was isolated form the reaction mixture in 18 % yield. An X‐ray diffraction study revealed that phosphine **1′** contains two heterocyclic R^1^ groups and one acylic amino group with a pending cyanamide function (Figure 1, right). The formation of **1′** presumably results from a lithium‐mediated isomerization of one R^1^ group.

In an alternative attempt, the bulky imine groups were introduced stepwise by first generating the phosphenium salt (R^1^_2_P)Cl from the reaction of R^1^SiMe_3_ with PCl_3_ under elimination of Me_3_SiCl. Subsequent treatment of (R^1^_2_P)Cl with a third equiv. of LiR^1^ gave phosphine **1** after recrystallization from *n*‐hexane as a white crystalline solid in 90 % yield (Scheme 3, bottom; see the Supporting Information). Contrary to IAPs with less bulky *N*‐heterocyclic imine substituents,^[16]^ phosphine **1** does not form coordination compounds with LiCl since the exocyclic imine nitrogen atoms are sterically shielded by the *tert*‐butyl groups.

An X‐ray diffraction study with crystals of **1** (see the Supporting Information) revealed a propeller‐like arrangement of the diaminoheterocycles around the phosphorus center with twist angles of about 46° between the imidazolidine rings and the plane spanned by the exocyclic nitrogen atoms (Figure 1, left). The six bulky *tert*‐butyl groups effectively encapsulate the reactive phosphorous atom. The metric parameters do not indicate the presence of any steric strain in **1**.

### Reactivity of phosphines 1 and 2

**Scheme 4:**
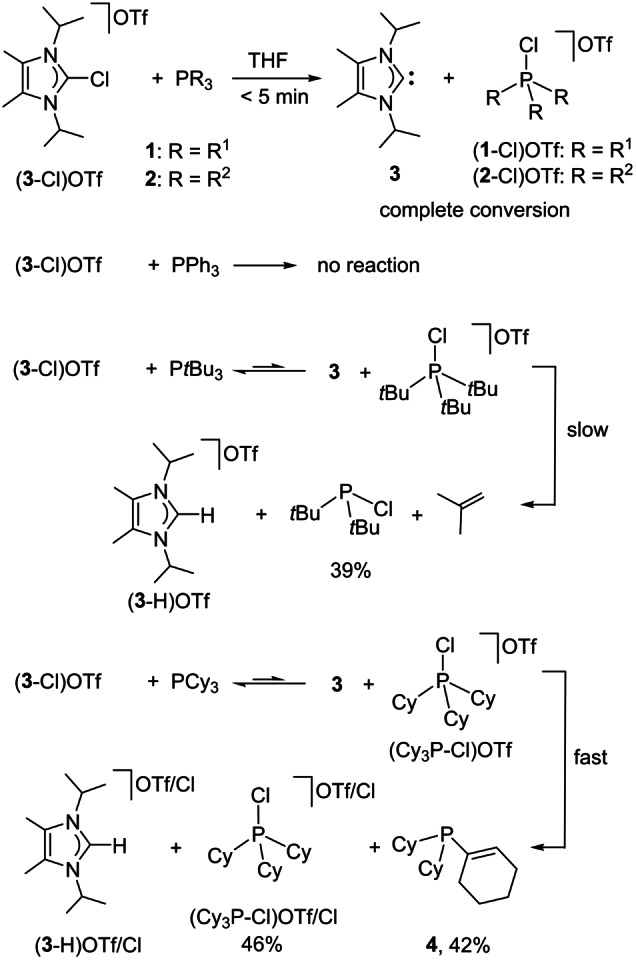
Reaction of (**3**‐Cl)OTf with different phosphines in THF at room temperature. Quantitative conversion to carbene **3** was observed with phosphines **1** or **2**. An equilibrium state, followed by partial decomposition reactions, was observed with alkyl phosphines (yields were determined by ^31^P NMR spectroscopy).

The tetrakisalkyl‐substituted 2‐chloroimidazolium salt (**3**‐Cl)OTf was initially selected to test the chloronium ion abstraction with the electron‐rich phosphines **1** and **2** (Scheme 4). Mixing of (**3**‐Cl**)**OTf with IAPs **1** or **2** in THF at room temperature afforded, within minutes, a mixture of the free carbene **3** and the chlorophosphonium salts (**1**‐Cl**)**OTf and (**2**‐Cl**)**OTf, respectively (Scheme 4, top; see the Supporting Information section 1.18). The quantitative Cl^+^ ion transfer was confirmed by shifting of the ^31^P NMR resonance from *δ*=84.3 ppm (**1**) or 79.6 ppm (**2**)^[16a]^ for the free phosphines, to *δ*=−53.9 ppm (**1**‐Cl)OTf) or −21.2 ppm (**2**‐Cl)OTf) for the chlorophosphonium salts. Carbene **3** was isolated from the mixture by extraction with *n*‐hexane. It showed the characteristic ^13^C NMR resonance at *δ*=207.4 ppm for the C_NHC_ carbon atom (Figures S71 and S81).^[6a]^

**Figure 2:**
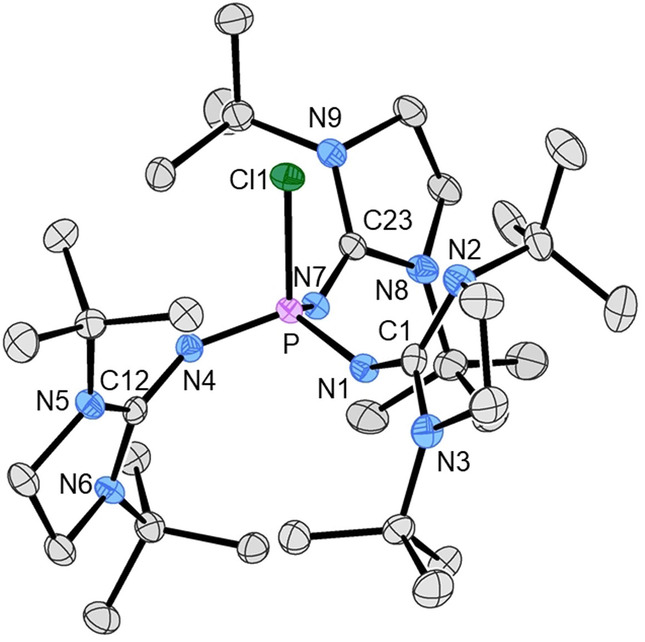
Molecular structure of (**1**‐Cl)^+^ in (**1**‐Cl)Cl⋅THF. Hydrogen atoms are omitted for clarity and thermal ellipsoids are set at 50 % probability. Selected bond lengths [Å] and angles [°]: P1–Cl1 2.0858(8), P1–N1 1.594(2), P1–N4 1.557(2), P1–N7 1.589(2), N1–C1 1.304(3), N4–C12 1.308(3), N7–C23 1.302(3); Cl1‐P1‐N1 107.91(8), Cl1‐P1‐N4 105.57(8), Cl1‐P1‐N7 109.70(8), N1‐P1‐N4 113.28(11), N1‐P1‐N7 109.99(10), N4‐P1‐N7 110.24(10), P1‐N1‐C1 138.0(2), P1‐N4‐C12 148.0(2), P1‐N7‐C23 137.0(2).

The chlorophosphonium salt (**1**‐Cl)Cl has been prepared separately by the oxidative chlorination of IAP **1** with hexachloroethane (see the Supporting Information, section 1.8). It was used for comparison of its NMR parameters to those of (**1**‐Cl)OTf and its molecular structure was determined by X‐ray diffraction. The structure determination of (**1**‐Cl)Cl⋅THF confirmed the formation of the P−Cl bond at a distorted tetrahedral phosphorus atom (Figure 2). Formation of the chlorophosphonium cation leads to a shortening of the P−N bond lengths in (**1**‐Cl)Cl compared to **1**. In addition, the angles at the phosphorus atom approach the tetrahedral value with a concurrent expansion of the N−P−N angles from 97.4(3)–98.9(4)° in **1** to 109.99(10)–113.28(11)° for (**1**‐Cl)Cl.

PPh_3_ does not react with (**3**‐Cl)OTf at ambient temperature (Scheme 4; see the Supporting Information section 1.19), suggesting a significantly lower chloronium ion affinity of PPh_3_ compared to that of **1** or **2**. However, the more basic alkylphosphines PCy_3_ and P*t*Bu_3_ react with (**3**‐Cl)OTf, albeit non‐selectively (Scheme 4). ^31^P NMR monitoring of the reaction of P*t*Bu_3_ with (**3**‐Cl)OTf reveals an equilibrium state at 27 % Cl^+^ uptake by the phosphine. The formed chlorophosphonium salt (*t*Bu_3_P−Cl)OTf decomposed slowly via *t*Bu deprotonation by the generated carbene **3**, resulting in a Hoffmann elimination of 2‐methylpropene and formation of *t*Bu_2_PCl (see the Supporting Information, section 1.20).

The reaction of PCy_3_ with (**3**‐Cl)OTf produces a mixture of (**3**‐H)OTf/Cl, (Cy_3_P−Cl)OTf/Cl and a new phosphine **4**. The formation of **4** can be rationalized by cyclohexyl deprotonation at the initially formed (Cy_3_P−Cl)OTf by the generated free NHC **3**, giving rise to the corresponding phosphonium ylide intermediate, which converts to the phosphine **4** upon HCl elimination. This assumption was verified by adding another equiv. of **3** to the reaction mixture, producing **4** in quantitative yield (see the Supporting Information, section 1.21).

These experiments demonstrate that the generation of free carbenes from classical, monocationic 2‐chloroazolium salts requires phosphines with sufficient chloronium ion affinity, such as **1** and **2**, and the absence of acidic protons in the formed chlorophosphonium salts. Consequently, only phosphines **1** and **2** were used in the subsequent studies.

### Selectivity of chloronium ion abstraction

**Scheme 5:**
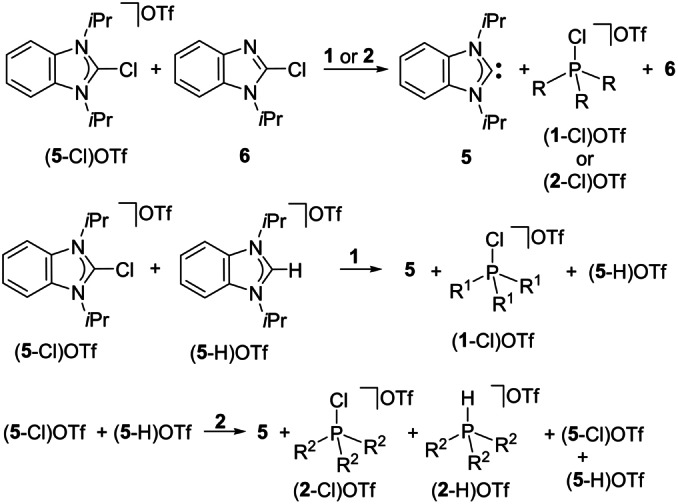
Selectivity of the chloronium ion abstraction using phosphines **1** and **2**.

In order to explore the selectivity of the dechlorination reaction with regards to the NHC precursor, an equimolar mixture of 2‐chlorobenz‐imidazolium triflate (**5**‐Cl)OTf and the neutral 2‐chloro‐*N*‐*iso*propyl‐benzimidazole **6** was treated with phosphines **1** or **2** (Scheme 5, top). The product mixtures obtained were analyzed by NMR spectroscopy, revealing that both phosphines react selectively with the imidazolium salt (**5**‐Cl)OTf, as indicated by the characteristic ^13^C NMR resonance of the formed carbene **5** at *δ*=222.5 ppm and the ^31^P resonances of the chlorophosphonium salts (**1**‐Cl)OTf and (**2**‐Cl)OTf, respectively. The neutral 2‐chloro‐*N*‐*iso*propylbenzimidazole **6** remained unchanged in the reaction mixture (Scheme 5, top; see the Supporting Information, section 1.22).

The behavior of **1** or **2** towards an equimolar mixture of the 2‐chlorobenzimidazolium salt (**5**‐Cl)OTf and C2−H benzimidazolium salt (**5**‐H)OTf was also studied (Scheme 5, bottom; see the Supporting Information, section 1.23). The *t*Bu substituted phosphine **1** reacts selectively by abstraction of a chloronium ion from (**5**‐Cl)OTf, while no reaction was observed with the C2−H benzimidazolium salt (**5**‐H)OTf. We assume that this can be attributed to the good accessibility of the chloride in (**5**‐Cl)OTf, while the shorter C2−H bond in (**5**‐H)OTf renders the proton less accessible.

In contrast to the selective dechlorination achieved with **1**, the less sterically encumbered phosphine **2** reacts with both benzimidazolium salts (**5**‐Cl)OTf and (**5**‐H)OTf to give an approximately 2 : 1 mixture of the chlorophosphonium salt (**2**‐Cl)OTf and the protonated phosphine (**2**‐H)OTf together with the free NHC **5** and some unreacted benzimidazolium salts (**5**‐Cl)OTf and (**5**‐H)OTf (see the Supporting Information, section 1.23). The benzimidazolium salt (**5**‐H)OTf remaining in the reaction mixtures, and the NHC **5** obtained with both **1** and **2**, gave only one set of resonances due to dynamic proton exchange between (**5**‐H)OTf and NHC **5** (see the Supporting Information, section 1.23). From the amounts of phosphonium salts (**2**‐Cl)OTf and (**2**‐H)OTf obtained, we conclude that the 2‐chlorobenzimidazolium salt (**5**‐Cl)OTf is more suitable for an attack by IAP **2** compared to its C2‐protonated congener (**5**‐H)OTf (see the Supporting Information, Scheme S10, Figure S105). This observation underlines the superiority of the bulky substituted phosphine **1** in the selective chloronium ion abstraction compared to the less bulky substituted phosphine **2**.

### Carbene scope

**Scheme 6:**
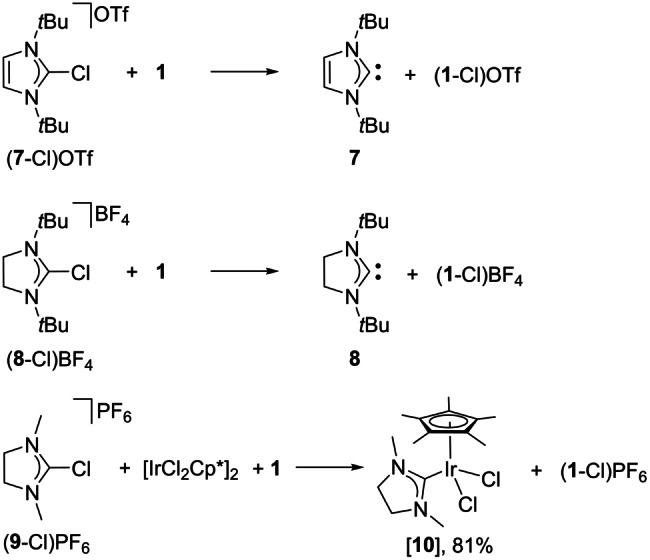
Scope of the chloronium ion abstraction using **1**.

To demonstrate the general applicability of the chloronium ion abstraction for the generation of NHCs, the C4/C5 unsubstituted azolium salts (**7**‐Cl)OTf and (**8**‐Cl)BF_4_ have been prepared (see the Supporting Information, sections 1.16 and 1.17) and were reacted with phosphine **1** (see the Supporting Information, section 1.24). Both azolium cations were readily dechlorinated by the phosphine to give the free carbenes **7** (*δ*(C_NHC_)=212.9 ppm, Figure S107) and **8** (*δ*(C_NHC_=238.6 ppm, Figure S111) together with the chlorophosphonium salts (**1**‐Cl)OTf and (**1**‐Cl)BF_4_, respectively. In addition, the sterically non‐demanding *N*,*N*′‐substituted imidazolidinium salt (**9**‐Cl)PF_6_ was prepared. Upon C2‐deprotonation the NHC **9** is initially formed, which is not stable but dimerizes to the entetramine **9=9**.[^18a^, ^18b^] Therefore, NHC **9** was generated from (**9**‐Cl)PF_6_ by dechlorination with **1** in the presence of [IrCl_2_Cp*]_2_ leading directly to the NHC complex [Ir(**9**)Cl_2_Cp*] [**10**] (Scheme 6). Complex [**10**] was isolated in 81 % yield featuring resonance for the C_NHC_ carbon atom at *δ*(C_NHC_)=186.3 ppm, which is characteristic for an imidazolidin‐2‐ylidene coordinated to Ir^III^ (*δ*(C_NHC_)=187.6 ppm).^[18c]^

### Coordination of IAPs 1 and 2 to transition metals

**Scheme 7:**
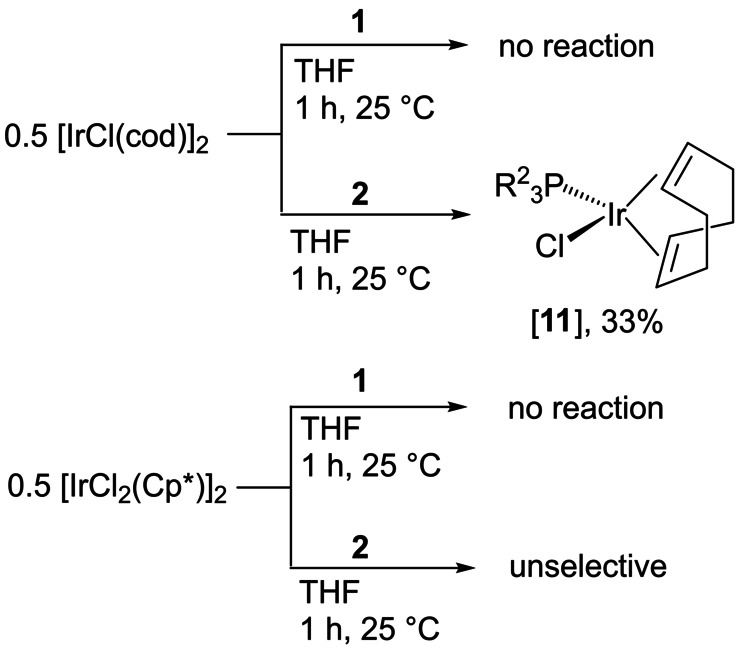
Reactivity of phosphines **1** and **2** with iridium complexes.

Phosphines **1** and **2** were reacted with Ir^I^ complex [IrCl(cod)]_2_ and Ir^III^ complex [IrCl_2_Cp*]_2_ (Scheme 7; see the Supporting Information, section 1.26) in order to evaluate the ability of the phosphines to coordinate to transition metals. Such undesirable coordination might consume the phosphine and prevent the in situ dechlorination reaction and subsequent metal‐NHC complex formation, as depicted in Scheme 6, bottom.

Complex [IrCl(cod)]_2_ reacted with the sterically less demanding phosphine **2** to give the phosphine complex [**11**], as indicated by ^31^P NMR spectroscopy (*δ*(^31^P)=31.8 ppm, Figure S119). With [IrCl_2_Cp*]_2_, IAP **2** gave a mixture containing several phosphorus‐containing species (see the Supporting Information, Figure S116). Contrary to the coordination of **2**, phosphine **1** showed no reaction with either of the two iridium complexes (Figures S114 and S115) and also did not react with [Ni(CO)_4_] as the preparation of [Ni(**1**)(CO)_3_] was attempted to rank the electron‐donating ability of **1**.

**Figure 3:**
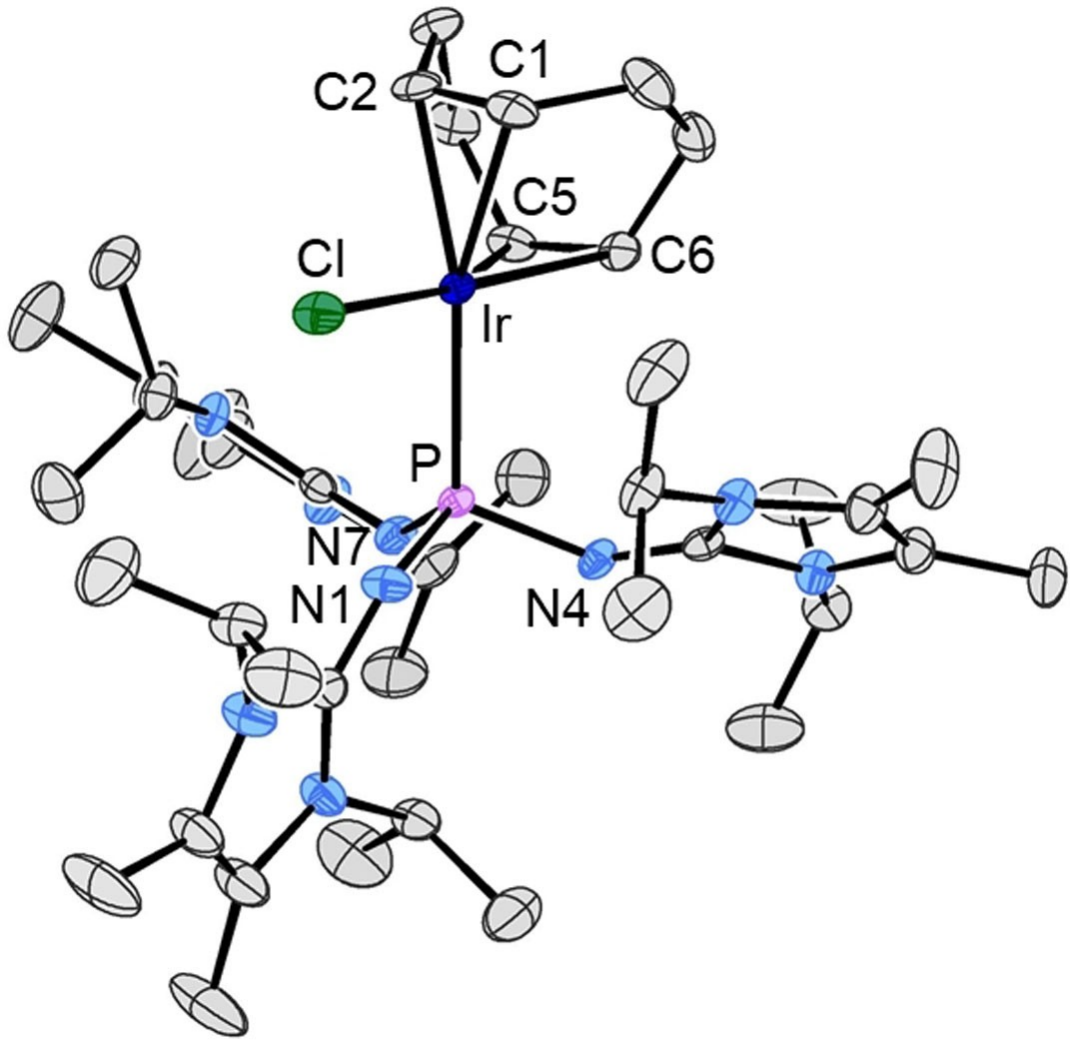
Molecular structure of [Ir(**2**)Cl(cod)] [**11**]. Hydrogen atoms are omitted for clarity and thermal ellipsoids are set at 50 % probability. Selected bond lengths [Å] and angles [°]: Ir–P 2.3473(6), Ir–Cl 2.3635(6), Ir–C1 2.187(2), Ir–C2 2.199(2), Ir–C5 2.099(2), Ir–C6 2.111(3), P–N1 1.639(2), P–N4 1.652(2), P–N7 1.641(2); Cl‐Ir‐P 93.36(2), Ir‐P‐N1 111.97(8), Ir‐P‐N4 113.09(8), Ir‐P‐N7 115.07(8), N1‐P‐N4 107.33(11), N1‐P‐N7 108.97(12), N4‐P‐N7 99.51(11).

Crystals of [**11**] were grown by slow diffusion of diethyl ether into a THF solution of [**11**]. The molecular structure of [**11**] is depicted in Figure 3. The structure analysis confirms coordination of one phosphine **2** to the Ir^I^ center. As expected, the iridium atom in [**11**] is coordinated in a distorted square‐planar fashion. Comparable metric parameters fall in the typical range observed previously for related Ir^I^ complexes.^[19]^ The Ir−C bond lengths in a *trans* position with respect to the phosphine are longer than those in a *trans* position relative to the chlorido ligand, which is caused by the stronger *σ*‐donor/*π*‐acceptor properties of the phosphine compared to the chlorido ligand.

In summary, phosphine **1** appears perfectly suitable for the generation of free carbenes from 2‐chloroazolium salts. The synthesis of NHC‐metal complexes via the in situ generation of NHCs is also possible owing to the steric demand of phosphine **1**, which exhibits a low tendency for metal coordination. Chloronium ion abstraction by **1** occurs selectively from 2‐chloroazolium salts, while **1** does not react with neutral 2‐chloroazoles. The reaction of **1** with a mixture of C2−Cl and C2−H benzimidazolium salts (Scheme 5) produces exclusively the NHC by chloronium ion abstraction while deprotonation of the C2−H benzimidazolium cation was not observed. The chloronium ion abstraction by **1** is applicable to various azolium salts and does not require electron‐poor or C4/C5‐substituted imidazolium NHC precursors. It constitutes a general alternative to the deprotonation of C2−H azolium precursors and is particularly useful when the relatively harsh conditions for the deprotonation reaction are not tolerated.

### Site‐selective metallation of bis‐NHC precursors

**Scheme 8:**
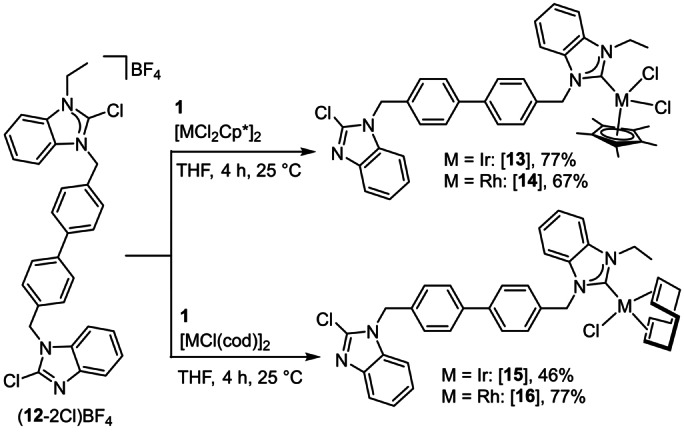
Site‐selective chloronium ion abstraction and metallation of a 2‐chlorobenzimidazolium/2‐chlorobenzimidazole bis‐NHC precursor using IAP **1**.

The facile and highly selective insertion of metal ions into the C−Cl bond has been utilized for the preparation of heterobimetallic complexes from single‐frame bis‐NHC precursors.^[20]^ Such complexes have found multiple applications in tandem^[3]^ and cooperative catalysis.^[20e]^ Since symmetric bis‐NHC precursors cannot be metallated site‐selectively, they have to be modified in a way that metallation of the two NHC precursors occurs by two chemically different reactions. Initial experiments have been performed with a bis‐NHC precursor composed of a C2−H azolium and a 2‐halogenoazole group. In a deprotonation/metallation reaction sequence, only the azolium group reacts to give a metal‐NHC moiety, while the 2‐halogenoazole remains unchanged. A subsequent oxidative addition of the C2−X bond of the 2‐halogenoazole then generates the heterobimetallic complex in a site‐selective manner.[^20a^, ^20b^] Subsequently, the halogenoazole/halogenoazolium bis‐NHC precursor (**12**‐2Cl)BF_4_ was prepared (Scheme 8).^[20c]^ It was found that in an oxidative addition, the 2‐halogenoazolium group reacts selectively first to give a metal‐NHC moiety, while the neutral halogenoazole subsequently can undergo a second oxidative addition under more drastic conditions with formation of a heterobimetallic complex. It should be noted that the activation of the C2−X bonds of this bis‐NHC precursor by oxidative addition exclusively leads to complexes where the oxidation state of both metals has been increased by +2. Contrary to this, the chloronium ion abstraction from a 2‐halogenoazolium NHC precursor followed by metallation does not change the oxidation state of the metal.

The site‐selective metallation of the NHC precursors in (**12‐**2Cl)BF_4_ was studied by chloronium ion abstraction with **1** in the presence of Ir^III^, Rh^III^, Ir^I^, or Rh^I^ complexes (Scheme 8). In all cases, the chloronium ion abstraction from the 2‐chlorobenzimidazolium site was observed with formation of complexes [**13**]–[**16**] in moderate to good yields. As expected from the previous studies (Scheme 5, top), the 2‐chlorobenzimidazole group did not participate in these reactions. In addition, coordination of the phosphine **1** was not observed in any case and the oxidation state of the metal center did not change.

Complexes [**13**]–[**16**] were fully characterized by NMR spectroscopy and mass spectrometry (see the Supporting Information). The ^13^C{^1^H} NMR spectra feature the characteristic C_NHC_ resonances at *δ*=171.1 and *δ*=185.6 ppm (d, ^1^ *J*=56.4 Hz) for the Ir^III^ complex [**13**] and the Rh^III^ complex and [**14**], respectively. These values are in good agreement with those recorded for related Ir^III[21, 22]^ and Rh^III[21]^ complexes. The C_NHC_ resonances are shifted to lower field in the Ir^I^ ([**15**]: *δ*=192.5 ppm) and Rh^I^ ([**16**]: *δ*=197.2 ppm, d, ^1^ *J*=51.0 Hz) complexes in accord with previous observations[^23^, ^24^] and theoretical predictions.^[25]^

**Figure 4:**
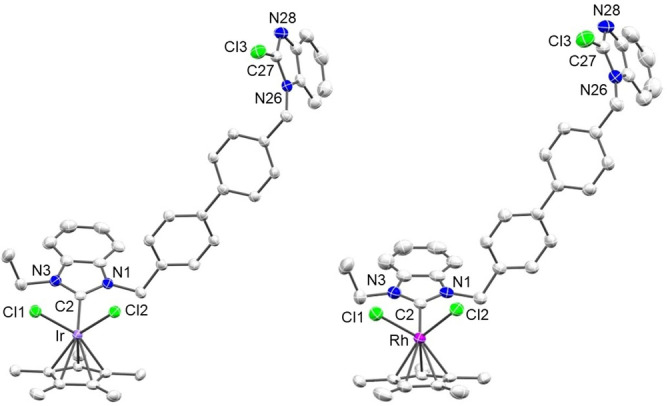
Molecular structures of [**13**] (left) and [**14**] (right). Hydrogen atoms are omitted for clarity and thermal ellipsoids are set at 50 % probability. Selected bond lengths [Å] and angles [°] for [**13**] and [**14**]: M–Cl1 2.4138(12) [2.4134(10)], M–Cl2 2.4095(12) [2.4063(9)], M–C2 2.030(5) [2.044(4)], range M–C_Cp*_ 2.148(5)–2.226(5) [2.139(3)–2.222(4)], N1–C2 1.369(7) [1.364(5)], N3–C2 1.359(6) [1.355(5)], Cl3–C27 1.695(6) [1.698(4)], N26–C27 1.362(7) [1.348(5)], N28–C27 1.309(7) [1.309(5)]; Cl1‐M‐Cl2 86.19(4) [88.25(3)], Cl1‐M‐C2 93.35(14) [94.27(11)], Cl2‐M‐C2 93.06(14) [93.90(10)], N1‐C2‐N3 105.2(4) [105.4(3)], N26‐C27‐N28 115.5(5) [115.6(4)].

**Figure 5:**
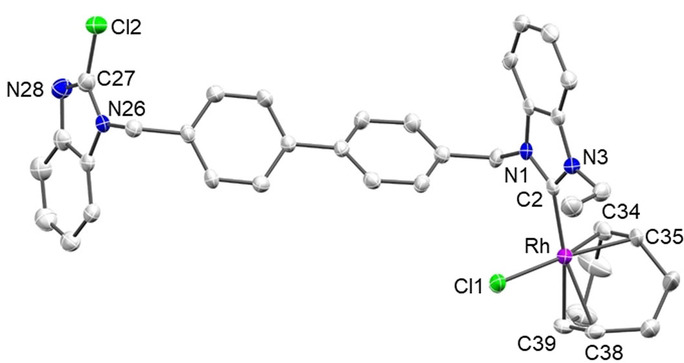
Molecular structures of [**16**]. Hydrogen atoms are omitted for clarity and thermal ellipsoids are set at 50 % probability. Selected bond lengths [Å] and angles [°]: Rh–Cl1 2.3627(11), Rh–C2 2.001(4), Rh–C34 2.094(5), Rh–C35 2.102(4), Rh–C38 2.199(4), Rh–C39 2.209(4), N1–C2 1.347(6), N3–C2 1.348(5), N26–C27 1.344(6), N28–C27 1.299(6), Cl2–C27 1.708(5); Cl1‐Rh‐C2 89.58(12), N1‐C2‐N3 106.2(4), N26‐C27‐N28 115.7(4).

X‐ray diffraction molecular structure determinations with crystals of [**13**], [**14**] (Figure 4), and [**16**] (Figure 5) confirmed the site‐selective metallation of the 2‐chlorobenzimidazolium site. The conceivable oxidative addition of a C−Cl bond to the Ir^I^ or Rh^I^ precursors,[^20b^, ^20c^] normally requiring more drastic reaction conditions, was not observed in any case. In fact, the isolation of the Ir^I^ and Rh^I^ complexes [**15**] and [**16**] confirmed that the NHC generation was achieved by chloronium ion abstraction rather than by oxidative addition, which would have caused an increase of the oxidation state of the metal center.

Complexes [**13**] and [**14**] feature essentially identical M−Cl, M−C_NHC_, and M−C_Cp_ bond lengths in the expected range.[^22^, ^23^] The N−C2 bond lengths in the metallated diaminoheterocycles are identical within experimental error while the N−C27 bonds of the 2‐chlorbenzimidazole group differ significantly in lengths ([**13**]: N26−C27 1.362(7) Å, N28−C27 1.309(7) Å; [**14**]: N26−C27 1.348(5) Å, N28−C27 1.309(5) Å). The differences between the two diaminoheterocycles is also reflected in the N1−C2−N3 ([**13**]: 105.2(4)°; [**14**]: 105.4(3)°) and N26−C27−N28 ([**13**]: 115.5(5)°; [**14**]: 115.6(4)°) bond angles.

Compared to Rh^III^ complex [**14**], Rh^I^ complex [**16**] (Figure 5) features slightly shorter Rh−Cl and Rh−C_NHC_ bond lengths owing to the lower coordination number of the metal center. As was observed for [**11**] (Figure 3), the Rh−C_cod_ bond lengths differ depending on the donor in *trans* position with respect to the double bonds.

**Scheme 9:**
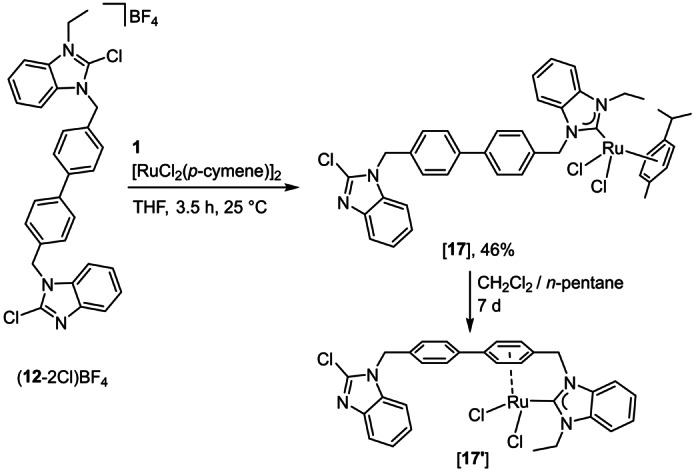
Synthesis of Ru^II^ complexes [**17**] and [**17′**].

In addition, chloronium ion abstraction from (**12**‐2Cl)BF_4_ in the presence of [RuCl_2_(*p*‐cymene)]_2_ was used for the preparation of Ru^II^ complex [**17**] (Scheme 9). The ^13^C{^1^H} NMR spectrum of [**17**] exhibited the characteristic resonances for the C_NHC_ carbon atom at *δ*=190.8 ppm and for the chlorinated benzimidazole carbon atom at *δ*=141.1 ppm. In addition, 10 resonances for the coordinated *p*‐cymene ligand were recorded (see the Supporting Information).

**Figure 6:**
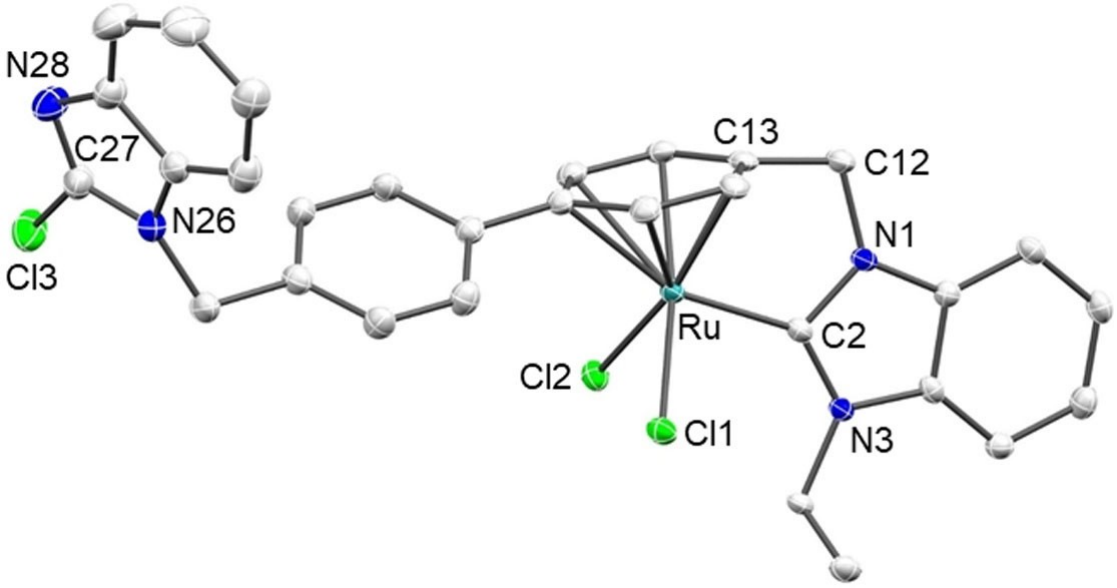
Molecular structures of [**17′**]. Hydrogen atoms are omitted for clarity and thermal ellipsoids are set at 50 % probability. Selected bond lengths [Å] and angles [°]: Ru–Cl1 2.4075(5), M–Cl2 2.3988(5), Ru–C2 2.044(2), range Ru–C_Ph_ 2.112(2)–2.286(2), N1–C2 1.368(3), N3–C2 1.348(3), N26‐C27 1.357(3), N28–C27 1.290(3), Cl3–C27 1.724(3); Cl1‐Ru‐Cl2 88.27(2), Cl1‐Ru‐C2 92.67(6), Cl2‐Ru‐C2 89.65(6), N1‐C2‐N3 106.1(2), N26‐C27‐N28 116.5(2).

Recrystallization of [**17**] from a CH_2_Cl_2_/*n*‐pentane solution over 7 days resulted in a ligand rearrangement to give complex [**17′**] (Scheme 9). The substitution of a *p*‐cymene ligand from Ru^II^ upon coordination of a benzyl‐substituted NHC is not uncommon and has been described previously.^[26]^ The X‐ray diffraction analysis with crystals of [**17′**] (Figure 6) confirmed the formation of a [RuCl_2_(NHC)(η^6^‐phenyl)] complex with a dangling 2‐chlorobenzimidazole arm. Metric parameters in [**17′**] compare well to equiv. parameters found in related ruthenium(II) complexes.^[26]^

**Scheme 10:**
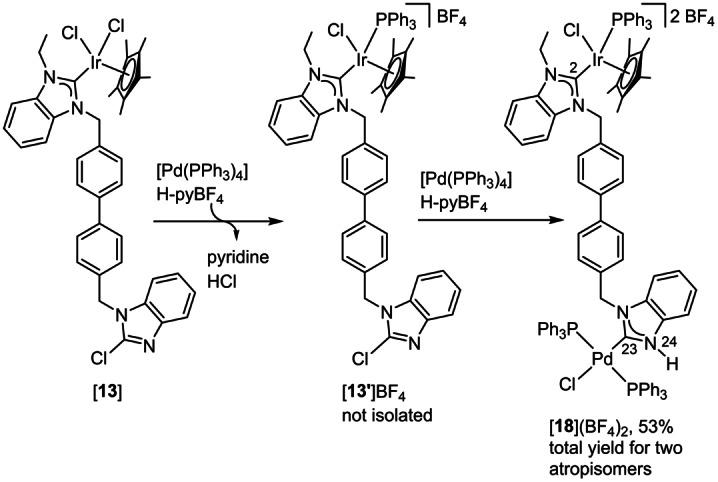
Synthesis of heterobimetallic [**18**](BF_4_)_2_ by metallation of [**13**].

Finally, the heterobimetallic complex [**18**](BF_4_)_2_ was prepared (Scheme 10). A sample of the mononuclear iridium(III) complex [**13**] was reacted with 2 equiv. of [Pd(PPh_3_)_4_] in the presence of 2 equiv. of pyridinium tetrafluoroborate. As indicated by NMR spectroscopy, the reaction proceeded by an initial exchange of a chlorido ligand at the iridium center for a phosphine ligand leading to the cationic complex [**13′**]BF_4_ and H‐pyCl. This complex was not isolated. In the next step, the C−Cl bond of the 2‐chlorobenzimidazole was oxidatively added to the second equiv. of [Pd(PPh_3_)_4_] followed by N‐protonation of the initially formed azolato complex.^[9]^ When only one equiv. of [Pd(PPh_3_)_4_] was used, only the ligand exchange at iridium to give [**13′**]BF_4_ but no oxidative addition of the 2‐chlorobenzimidazole moiety was observed.

The expected *cis* arrangement of the NHC and the chlorido ligand after the oxidative addition to palladium was not observed as it would lead to the coordination of two good *σ*‐donors (NHC and PPh_3_) in *trans* positions. Such an arrangement is unstable owing to the transphobia effect,^[27]^ and thus, the *trans* NHC/chlorido complex [**18**](BF_4_)_2_ was obtained. Closely related phosphine/halogenido ligand rearrangement reactions have been described.[^9^, ^20d^]

The phosphine‐chlorido ligand exchange generates a chiral center at the iridium atom leading to a mixture of two atropisomers of [**18**](BF_4_)_2_. The major isomer **A** (54 %) has been isolated and was fully characterized by NMR spectroscopy and mass spectrometry (identical mass values for both atropisomers were observed, see the Supporting Information, section 1.32). The resonance for atom H24 of the protic NHC was observed in the ^1^H NMR spectrum at *δ*=11.18. The ^13^C{^1^H} NMR spectrum features the resonances for the two carbene carbon atoms (*δ*(C23)=171.8, t, ^2^ *J*_CP_=8.7 Hz; *δ*(C2)=159.5, d, ^2^ *J*_CP_=12.1 Hz) in the expected range with the correct multiplicity due to coupling to the phosphorus atoms. Owing to the chiral iridium center, the phenyl groups of the PPh_3_ ligands are magnetically different and the complete assignment of the resonances is presented in the Supporting Information, section 1.32.

**Figure 7:**
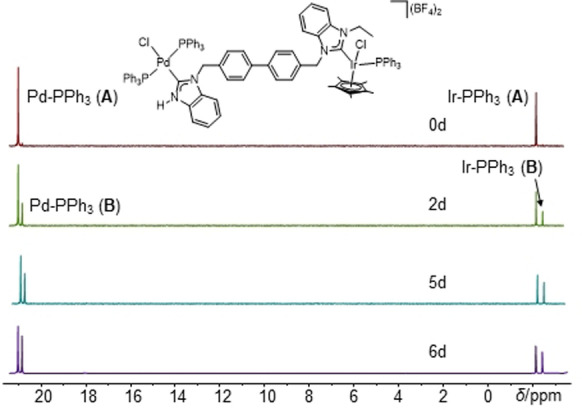
Time‐dependent ^31^P NMR spectra of [**18**](BF_4_)_2_ (isomer **A**) showing the partial conversion to isomer **B**.

Atropisomer **A** of [**18**](BF_4_)_2_ dissolved in CH_2_Cl_2_ partially converts over 6 days into atropisomer **B**, generating back the atropisomer mixture (54 : 46) observed during the synthesis of [**18**](BF_4_)_2_. This conversion has been monitored by ^1^H NMR spectroscopy (see the Supporting Information, Figure S136) and by ^31^P NMR spectroscopy (Figure 7). The time‐dependent ^31^P NMR spectra show that, at *t*=0, atropisomer **A** is exclusively present. After 2 days atropisomer **B** can be detected. After 6 days the equilibrium state is reached and no further changes of the composition of the mixture are observed.

## Conclusion

We describe a general method for the generation of NHCs from 2‐chloroazolium salts by chloronium ion abstraction with phosphines. While NHCs such as **5** (by deprotonation through heating of the benzimidazolium hydrogen carbonate^[28]^) or **8** (by reductive desulfurization of the corresponding thione^[18a]^) have been prepared previously, our new method offers selected advantages. Using the sterically demanding tris(1,3‐*tert*‐butylimidazolin‐2‐ylidenamino)‐phosphine **1**, the chloronium ion abstraction is highly chemoselective. While 2‐chlorobenzimidazolium salts are rapidly dechlorinated by **1**, 2‐chlorobenzimidazoles and C2−H benzimidazolium salts do not participate in competitive reactions with **1**. In addition, phosphine **1** did not coordinate to various transition metal centers, while the sterically less demanding phosphine **2** was shown to coordinate to Ir^I^ complexes. The reluctance of **1** to bind to metals was utilized to synthesize complex [**10**] via in situ generation of an unstable NHC, demonstrating the great potential of the dechlorination approach for the challenging synthesis of metal complexes bearing highly reactive carbenes. Furthermore, the chloronium ion abstraction allowed the site‐selective metallation of the 2‐chlorobenzimidazolium group in an unsymmetric 2‐chlorobenzimidazolium/2‐chlorobenzimidazole bis‐NHC precursor. A site‐selective, stepwise metallation was also used for the generation of the heterobimetallic complex [**18**](BF_4_)_2_ from the unsymmetric bis‐NHC precursor (**12**‐2Cl)BF_4_. Further studies will be directed towards unsymmetric bis‐NHC precursors composed of C2−Cl and C2−H azolium groups, which will allow the site‐selective metallation of single frame poly‐NHC ligands by chloronium ion abstraction/metallation and deprotonation/metallation.

## Conflict of interest

The authors declare no conflict of interest.

1

## Supporting information

As a service to our authors and readers, this journal provides supporting information supplied by the authors. Such materials are peer reviewed and may be re‐organized for online delivery, but are not copy‐edited or typeset. Technical support issues arising from supporting information (other than missing files) should be addressed to the authors.

Supporting Information

Supporting Information

## Data Availability

The data that support the findings of this study are available in the Supporting Information of this article.
