# Supplementary material for: Synthesis of N‐Heterocyclic Carbenes and Their Complexes by Chloronium Ion Abstraction from 2‐Chloroazolium Salts Using Electron‐Rich Phosphines
Source: Angew Chem Int Ed Engl. 2022 May 17;61(28):e202202190. doi: 10.1002/anie.202202190 (PMC9401039; doi:10.1002/anie.202202190)

checkCIF/PLATON report

Structure factors have been supplied for datablock(s) c2c

THIS REPORT IS FOR GUIDANCE ONLY. IF USED AS PART OF A REVIEW PROCEDURE FOR PUBLICATION, IT SHOULD NOT REPLACE THE EXPERTISE OF AN EXPERIENCED CRYSTALLOGRAPHIC REFEREE.

No syntax errors found. CIF dictionary Interpreting this report

Datablock: c2c

```
Bond precision:  C-C = 0.0025 A          Wavelength=0.71073

Cell:           a=28.1897(8)     b=18.4244(5)        c=21.7162(6)
                alpha=90         beta=100.636(2)     gamma=90
Temperature:    100 K

                Calculated                   Reported
Volume          11085.2(5)                   11085.1(5)
Space group     C 2/c                        C 1 2/c 1
Hall group      : -C 2yc                     -C 2yc
Moiety formula  C33 H66 N9 P                 C33 H66 N9 P
Sum formula     C33 H66 N9 P                 C33 H66 N9 P
Mr              619.92                       619.91
Dx,g cm-3       1.114                        1.114
Z               12                           12
Mu (mm-1)       0.109                        0.109
F000            4104.0                       4104.0
F000'           4106.25
h,k,lmax        37,24,28                     37,24,28
Nref            13822                        13787
Tmin,Tmax       0.971,0.992                  0.881,1.000
Tmin'           0.971

Correction method= # Reported T Limits: Tmin=0.881 Tmax=1.000
AbsCorr = MULTI-SCAN

Data completeness= 0.997          Theta(max)= 28.323

R(reflections)= 0.0519( 10539)    wR2(reflections)= 0.1444( 13787)

S = 1.012                   Npar= 708
```

The following ALERTS were generated. Each ALERT has the format
test-name_ALERT_alert-type_alert-level.
Click on the hyperlinks for more details of the test.

Alert level C

```
PLAT220_ALERT_2_C Non-Solvent  Resd 1  C   Ueq(max)/Ueq(min) Range        3.3 Ratio
PLAT906_ALERT_3_C Large K Value in the Analysis of Variance ......      2.418 Check
PLAT911_ALERT_3_C Missing FCF Refl Between Thmin & STh/L=    0.600         12 Report
```

Alert level G

```
PLAT083_ALERT_2_G SHELXL Second Parameter in WGHT  Unusually Large      11.80 Why ?
PLAT300_ALERT_4_G Atom Site Occupancy of P2         Constrained at        0.5 Check
PLAT301_ALERT_3_G Main Residue  Disorder ..............(Resd  1  )        23% Note
PLAT301_ALERT_3_G Main Residue  Disorder ..............(Resd  2  )         2% Note
PLAT720_ALERT_4_G Number of Unusual/Non-Standard Labels ..........          8 Note
PLAT790_ALERT_4_G Centre of Gravity not Within Unit Cell: Resd.  #          2 Note
              C33 H66 N9 P
PLAT910_ALERT_3_G Missing # of FCF Reflection(s) Below Theta(Min).          4 Note
PLAT912_ALERT_4_G Missing # of FCF Reflections Above STh/L=  0.600         20 Note
PLAT933_ALERT_2_G Number of OMIT Records in Embedded .res File ...          9 Note
PLAT978_ALERT_2_G Number C-C Bonds with Positive Residual Density.         10 Info
```

---

0 **ALERT level A** = Most likely a serious problem - resolve or explain
0 **ALERT level B** = A potentially serious problem, consider carefully
3 **ALERT level C** = Check. Ensure it is not caused by an omission or oversight
10 **ALERT level G** = General information/check it is not something unexpected

0 ALERT type 1 CIF construction/syntax error, inconsistent or missing data
4 ALERT type 2 Indicator that the structure model may be wrong or deficient
5 ALERT type 3 Indicator that the structure quality may be low
4 ALERT type 4 Improvement, methodology, query or suggestion
0 ALERT type 5 Informative message, check

It is advisable to attempt to resolve as many as possible of the alerts in all categories. Often the minor alerts point to easily fixed oversights, errors and omissions in your CIF or refinement strategy, so attention to these fine details can be worthwhile. In order to resolve some of the more serious problems it may be necessary to carry out additional measurements or structure refinements. However, the purpose of your study may justify the reported deviations and the more serious of these should normally be commented upon in the discussion or experimental section of a paper or in the "special_details" fields of the CIF. checkCIF was carefully designed to identify outliers and unusual parameters, but every test has its limitations and alerts that are not important in a particular case may appear. Conversely, the absence of alerts does not guarantee there are no aspects of the results needing attention. It is up to the individual to critically assess their own results and, if necessary, seek expert advice.

Publication of your CIF in IUCr journals

A basic structural check has been run on your CIF. These basic checks will be run on all CIFs submitted for publication in IUCr journals (*Acta Crystallographica*, *Journal of Applied Crystallography*, *Journal of Synchrotron Radiation*); however, if you intend to submit to *Acta Crystallographica Section C* or *E* or *IUCrData*, you should make sure that full publication checks are run on the final version of your CIF prior to submission.

Publication of your CIF in other journals

Please refer to the *Notes for Authors* of the relevant journal for any special instructions relating to CIF submission.

PLATON version of 19/10/2018; check.def file version of 15/10/2018

Datablock c2c - ellipsoid plot

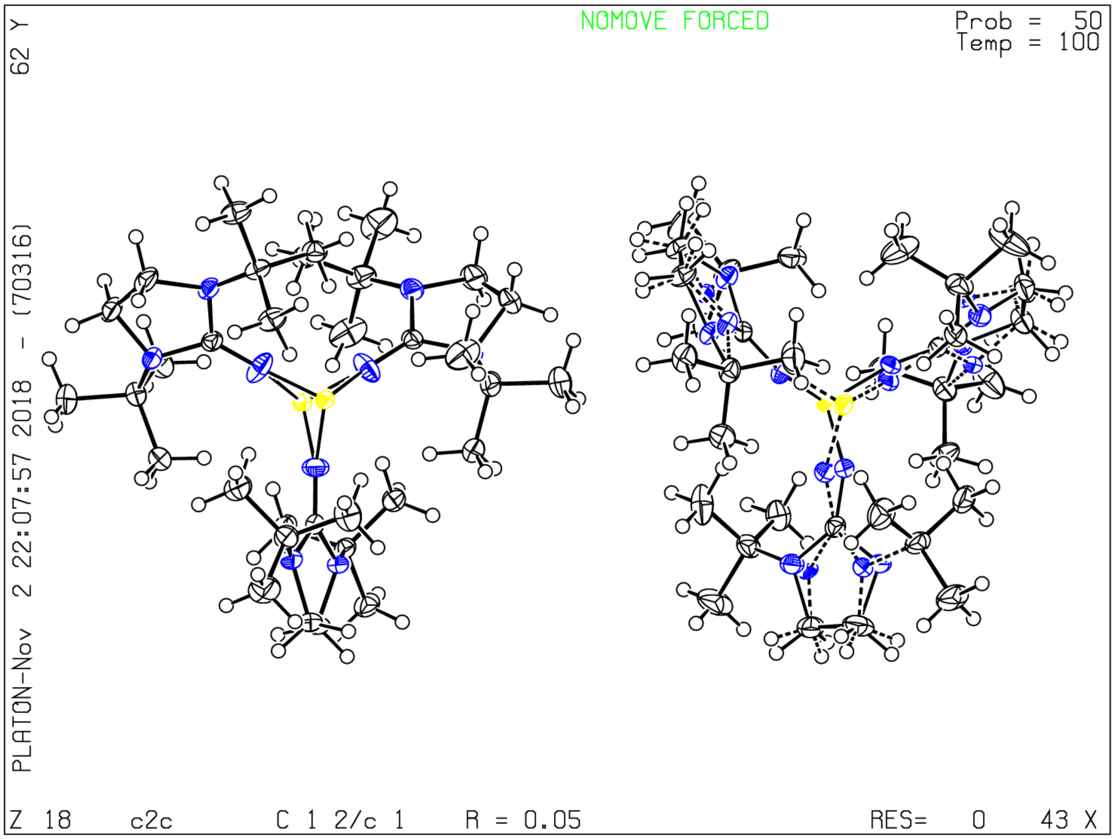

checkCIF/PLATON report

Structure factors have been supplied for datablock(s) cc_a

THIS REPORT IS FOR GUIDANCE ONLY. IF USED AS PART OF A REVIEW PROCEDURE FOR PUBLICATION, IT SHOULD NOT REPLACE THE EXPERTISE OF AN EXPERIENCED CRYSTALLOGRAPHIC REFEREE.

No syntax errors found.   CIF dictionary   Interpreting this report

Datablock: cc_a

```
Bond precision:  C-C = 0.0020 A          Wavelength=0.71073

Cell:            a=13.7379(2)     b=15.6992(2)        c=17.3874(2)
                 alpha=90         beta=103.029(1)     gamma=90
Temperature:     100 K

                 Calculated                 Reported
Volume           3653.47(8)                 3653.47(8)
Space group      C c                        C 1 c 1
Hall group       C -2yc                     C -2yc
Moiety formula   C33 H66 N9 P               C33 H66 N9 P
Sum formula      C33 H66 N9 P               C33 H66 N9 P
Mr               619.92                     619.91
Dx,g cm-3        1.127                      1.127
Z                4                          4
Mu (mm-1)        0.110                      0.110
F000             1368.0                     1368.0
F000'            1368.75
h,k,lmax         19,22,24                   19,22,24
Nref             11133[ 5572]               11032
Tmin,Tmax        0.956,0.968                0.856,1.000
Tmin'            0.936

Correction method= # Reported T Limits: Tmin=0.856 Tmax=1.000
AbsCorr = MULTI-SCAN

Data completeness= 1.98/0.99      Theta(max)= 30.513

R(reflections)= 0.0285( 10756)    wR2(reflections)= 0.0752( 11032)

S = 1.049                  Npar= 406
```

The following ALERTS were generated. Each ALERT has the format
test-name_ALERT_alert-type_alert-level.
Click on the hyperlinks for more details of the test.

Alert level C

```
PLAT220_ALERT_2_C Non-Solvent  Resd 1  N   Ueq(max)/Ueq(min) Range        3.9 Ratio
PLAT911_ALERT_3_C Missing FCF Refl Between Thmin & STh/L=    0.600          2 Report
```

Alert level G

```
PLAT230_ALERT_2_G Hirshfeld Test Diff for   N2       --C7        .        6.2 s.u.
PLAT912_ALERT_4_G Missing # of FCF Reflections Above STh/L=  0.600          8 Note
PLAT913_ALERT_3_G Missing # of Very Strong Reflections in FCF ....          1 Note
PLAT978_ALERT_2_G Number C-C Bonds with Positive Residual Density.         13 Info
```

```
   0 ALERT level A = Most likely a serious problem - resolve or explain
   0 ALERT level B = A potentially serious problem, consider carefully
   2 ALERT level C = Check. Ensure it is not caused by an omission or oversight
   4 ALERT level G = General information/check it is not something unexpected

   0 ALERT type 1 CIF construction/syntax error, inconsistent or missing data
   3 ALERT type 2 Indicator that the structure model may be wrong or deficient
   2 ALERT type 3 Indicator that the structure quality may be low
   1 ALERT type 4 Improvement, methodology, query or suggestion
   0 ALERT type 5 Informative message, check
```

It is advisable to attempt to resolve as many as possible of the alerts in all categories. Often the minor alerts point to easily fixed oversights, errors and omissions in your CIF or refinement strategy, so attention to these fine details can be worthwhile. In order to resolve some of the more serious problems it may be necessary to carry out additional measurements or structure refinements. However, the purpose of your study may justify the reported deviations and the more serious of these should normally be commented upon in the discussion or experimental section of a paper or in the "special_details" fields of the CIF. checkCIF was carefully designed to identify outliers and unusual parameters, but every test has its limitations and alerts that are not important in a particular case may appear. Conversely, the absence of alerts does not guarantee there are no aspects of the results needing attention. It is up to the individual to critically assess their own results and, if necessary, seek expert advice.

Publication of your CIF in IUCr journals

A basic structural check has been run on your CIF. These basic checks will be run on all CIFs submitted for publication in IUCr journals (*Acta Crystallographica*, *Journal of Applied Crystallography*, *Journal of Synchrotron Radiation*); however, if you intend to submit to *Acta Crystallographica Section C* or *E* or *IUCrData*, you should make sure that full publication checks are run on the final version of your CIF prior to submission.

Publication of your CIF in other journals

Please refer to the *Notes for Authors* of the relevant journal for any special instructions relating to CIF submission.

PLATON version of 23/04/2018; check.def file version of 23/04/2018

Datablock cc_a - ellipsoid plot

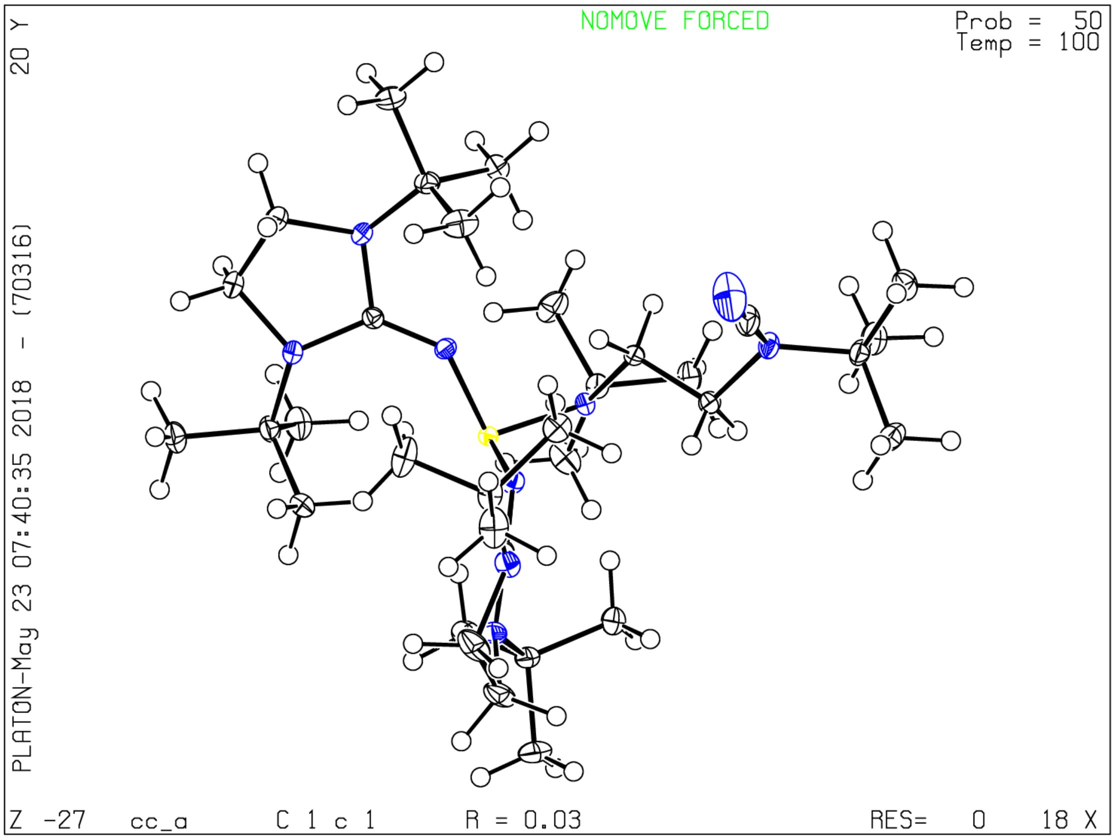

checkCIF/PLATON report

Structure factors have been supplied for datablock(s) p-1_a

THIS REPORT IS FOR GUIDANCE ONLY. IF USED AS PART OF A REVIEW PROCEDURE FOR PUBLICATION, IT SHOULD NOT REPLACE THE EXPERTISE OF AN EXPERIENCED CRYSTALLOGRAPHIC REFEREE.

No syntax errors found. CIF dictionary Interpreting this report

Datablock: p-1_a

```
Bond precision:  C-C = 0.0016 A          Wavelength=0.71073

Cell:          a=9.3397(2)       b=11.9445(3)      c=18.3451(5)
               alpha=84.113(1)   beta=88.737(1)    gamma=71.159(1)
Temperature:   100 K

                Calculated                Reported
Volume          1926.54(8)                1926.54(8)
Space group     P -1                      P -1
Hall group      -P 1                      -P 1
Moiety formula  C33 H66 Cl N9 P, Cl       C33 H66 Cl N9 P, Cl
Sum formula     C33 H66 Cl2 N9 P          C33 H66 Cl2 N9 P
Mr              690.82                    690.81
Dx,g cm-3       1.191                     1.191
Z               2                         2
Mu (mm-1)       0.245                     0.245
F000            752.0                     752.0
F000'           752.97
h,k,lmax        13,17,26                  13,17,26
Nref            11778                     11646
Tmin,Tmax       0.895,0.920               0.890,1.000
Tmin'           0.880

Correction method= # Reported T Limits: Tmin=0.890 Tmax=1.000
AbsCorr = MULTI-SCAN

Data completeness= 0.989          Theta(max)= 30.525

R(reflections)= 0.0348( 10777)    wR2(reflections)= 0.0972( 11646)

S = 1.029                 Npar= 427
```

The following ALERTS were generated. Each ALERT has the format
test-name_ALERT_alert-type_alert-level.
Click on the hyperlinks for more details of the test.

Alert level B

```
PLAT230_ALERT_2_B Hirshfeld Test Diff for    Cl1      --P1       .       15.3 s.u.
```

Alert level C

```
PLAT220_ALERT_2_C Non-Solvent  Resd 1  C   Ueq(max)/Ueq(min) Range        3.8 Ratio
PLAT911_ALERT_3_C Missing FCF Refl Between Thmin & STh/L=    0.600         12 Report
```

Alert level G

```
PLAT154_ALERT_1_G The s.u.'s on the Cell Angles are Equal ..(Note)      0.001 Degree
PLAT304_ALERT_4_G Non-Integer Number of Atoms in ...... Resd  2          0.50 Check
PLAT304_ALERT_4_G Non-Integer Number of Atoms in ...... Resd  3          0.50 Check
PLAT912_ALERT_4_G Missing # of FCF Reflections Above STh/L=  0.600        121 Note
PLAT933_ALERT_2_G Number of OMIT Records in Embedded .res File ...          1 Note
PLAT978_ALERT_2_G Number C-C Bonds with Positive Residual Density.         14 Info
```

```
   0 ALERT level A = Most likely a serious problem - resolve or explain
   1 ALERT level B = A potentially serious problem, consider carefully
   2 ALERT level C = Check. Ensure it is not caused by an omission or oversight
   6 ALERT level G = General information/check it is not something unexpected

   1 ALERT type 1 CIF construction/syntax error, inconsistent or missing data
   4 ALERT type 2 Indicator that the structure model may be wrong or deficient
   1 ALERT type 3 Indicator that the structure quality may be low
   3 ALERT type 4 Improvement, methodology, query or suggestion
   0 ALERT type 5 Informative message, check
```

It is advisable to attempt to resolve as many as possible of the alerts in all categories. Often the minor alerts point to easily fixed oversights, errors and omissions in your CIF or refinement strategy, so attention to these fine details can be worthwhile. In order to resolve some of the more serious problems it may be necessary to carry out additional measurements or structure refinements. However, the purpose of your study may justify the reported deviations and the more serious of these should normally be commented upon in the discussion or experimental section of a paper or in the "special_details" fields of the CIF. checkCIF was carefully designed to identify outliers and unusual parameters, but every test has its limitations and alerts that are not important in a particular case may appear. Conversely, the absence of alerts does not guarantee there are no aspects of the results needing attention. It is up to the individual to critically assess their own results and, if necessary, seek expert advice.

Publication of your CIF in IUCr journals

A basic structural check has been run on your CIF. These basic checks will be run on all CIFs submitted for publication in IUCr journals (*Acta Crystallographica*, *Journal of Applied Crystallography*, *Journal of Synchrotron Radiation*); however, if you intend to submit to *Acta Crystallographica Section C* or *E* or *IUCrData*, you should make sure that full publication checks are run on the final version of your CIF prior to submission.

Publication of your CIF in other journals

Please refer to the *Notes for Authors* of the relevant journal for any special instructions relating to CIF submission.

PLATON version of 19/10/2018; check.def file version of 15/10/2018

Datablock p-1_a - ellipsoid plot

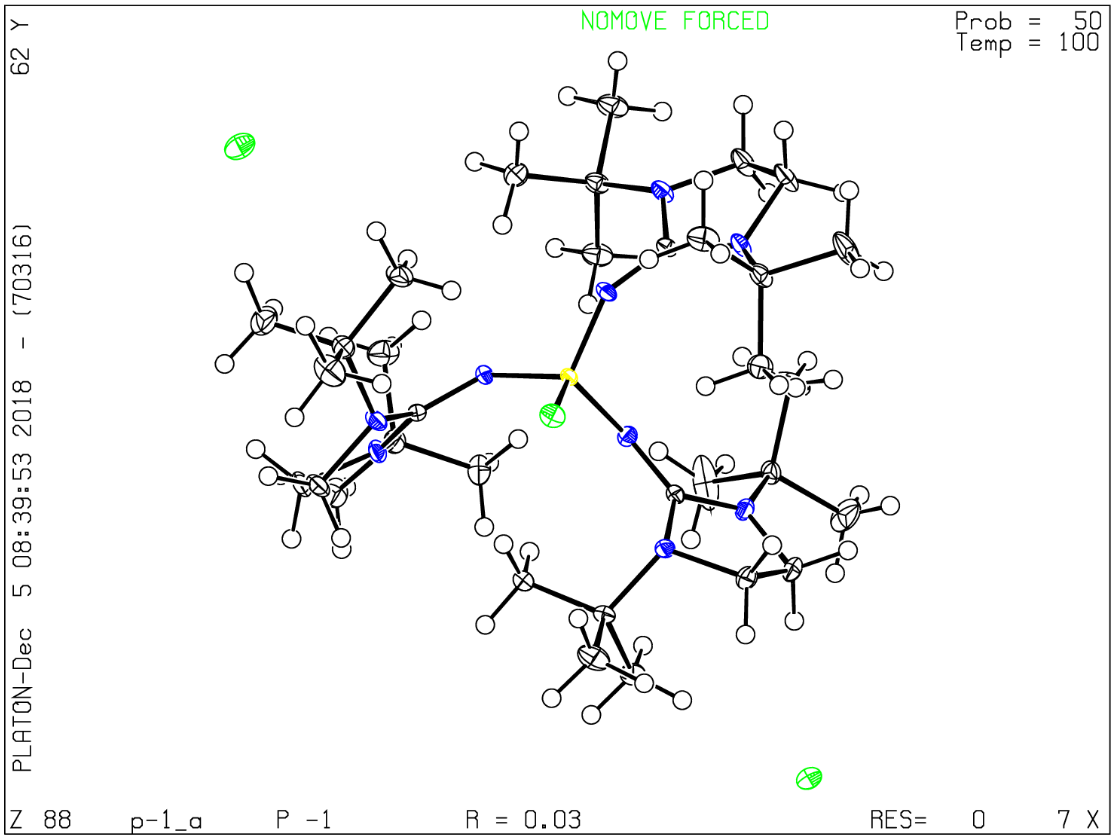

checkCIF/PLATON report

Structure factors have been supplied for datablock(s) p21n_a

THIS REPORT IS FOR GUIDANCE ONLY. IF USED AS PART OF A REVIEW PROCEDURE FOR PUBLICATION, IT SHOULD NOT REPLACE THE EXPERTISE OF AN EXPERIENCED CRYSTALLOGRAPHIC REFEREE.

No syntax errors found.   CIF dictionary   Interpreting this report

Datablock: p21n_a

```
Bond precision:  C-C = 0.0041 A          Wavelength=0.71073

Cell:            a=15.9563(4)     b=16.7655(5)        c=17.4161(5)
                 alpha=90         beta=113.596(2)     gamma=90
Temperature:     100 K
```

	Calculated	Reported
Volume	4269.5(2)	4269.5(2)
Space group	P 21/n	P 1 21/n 1
Hall group	-P 2yn	-P 2yn
Moiety formula	C33 H66 Cl N9 P, C4 H8 O, Cl	C33 H66 Cl N9 P, C4 H8 O, Cl
Sum formula	C37 H74 Cl2 N9 O P	C37 H74 Cl2 N9 O P
Mr	762.92	762.92
Dx,g cm-3	1.187	1.187
Z	4	4
Mu (mm-1)	0.229	0.229
F000	1664.0	1664.0
F000'	1666.02	
h,k,lmax	21,22,23	21,22,23
Nref	10722	10643
Tmin,Tmax	0.952,0.962	0.836,1.000
Tmin'	0.926	

```
Correction method= # Reported T Limits: Tmin=0.836 Tmax=1.000
AbsCorr = MULTI-SCAN

Data completeness= 0.993          Theta(max)= 28.396

R(reflections)= 0.0589( 7645)     wR2(reflections)= 0.1578( 10643)

S = 1.040                  Npar= 469
```

The following ALERTS were generated. Each ALERT has the format
test-name_ALERT_alert-type_alert-level.
Click on the hyperlinks for more details of the test.

Alert level C

```
PLAT220_ALERT_2_C NonSolvent   Resd 1  C   Ueq(max)/Ueq(min) Range        3.1 Ratio
PLAT243_ALERT_4_C High   'Solvent' Ueq as Compared to Neighbors of        O36 Check
PLAT244_ALERT_4_C Low    'Solvent' Ueq as Compared to Neighbors of        C36 Check
PLAT340_ALERT_3_C Low Bond Precision on  C-C Bonds ...............    0.00412 Ang.
PLAT906_ALERT_3_C Large K Value in the Analysis of Variance ......      4.093 Check
```

Alert level G

```
PLAT398_ALERT_2_G Deviating  C-O-C    Angle From 120 for O36          108.2 Degree
PLAT720_ALERT_4_G Number of Unusual/Non-Standard Labels ..........        1 Note
PLAT883_ALERT_1_G No Info/Value for _atom_sites_solution_primary .    Please Do !
PLAT910_ALERT_3_G Missing # of FCF Reflection(s) Below Theta(Min).        1 Note
PLAT912_ALERT_4_G Missing # of FCF Reflections Above STh/L=  0.600       80 Note
PLAT978_ALERT_2_G Number C-C Bonds with Positive Residual Density.        4 Info
```

---

0 **ALERT level A** = Most likely a serious problem - resolve or explain
0 **ALERT level B** = A potentially serious problem, consider carefully
5 **ALERT level C** = Check. Ensure it is not caused by an omission or oversight
6 **ALERT level G** = General information/check it is not something unexpected

1 ALERT type 1 CIF construction/syntax error, inconsistent or missing data
3 ALERT type 2 Indicator that the structure model may be wrong or deficient
3 ALERT type 3 Indicator that the structure quality may be low
4 ALERT type 4 Improvement, methodology, query or suggestion
0 ALERT type 5 Informative message, check

It is advisable to attempt to resolve as many as possible of the alerts in all categories. Often the minor alerts point to easily fixed oversights, errors and omissions in your CIF or refinement strategy, so attention to these fine details can be worthwhile. In order to resolve some of the more serious problems it may be necessary to carry out additional measurements or structure refinements. However, the purpose of your study may justify the reported deviations and the more serious of these should normally be commented upon in the discussion or experimental section of a paper or in the "special_details" fields of the CIF. checkCIF was carefully designed to identify outliers and unusual parameters, but every test has its limitations and alerts that are not important in a particular case may appear. Conversely, the absence of alerts does not guarantee there are no aspects of the results needing attention. It is up to the individual to critically assess their own results and, if necessary, seek expert advice.

Publication of your CIF in IUCr journals

A basic structural check has been run on your CIF. These basic checks will be run on all CIFs submitted for publication in IUCr journals (*Acta Crystallographica*, *Journal of Applied Crystallography*, *Journal of Synchrotron Radiation*); however, if you intend to submit to *Acta Crystallographica Section C* or *E* or *IUCrData*, you should make sure that full publication checks are run on the final version of your CIF prior to submission.

Publication of your CIF in other journals

Please refer to the *Notes for Authors* of the relevant journal for any special instructions relating to CIF submission.

PLATON version of 13/07/2021; check.def file version of 13/07/2021

Datablock p21n_a - ellipsoid plot

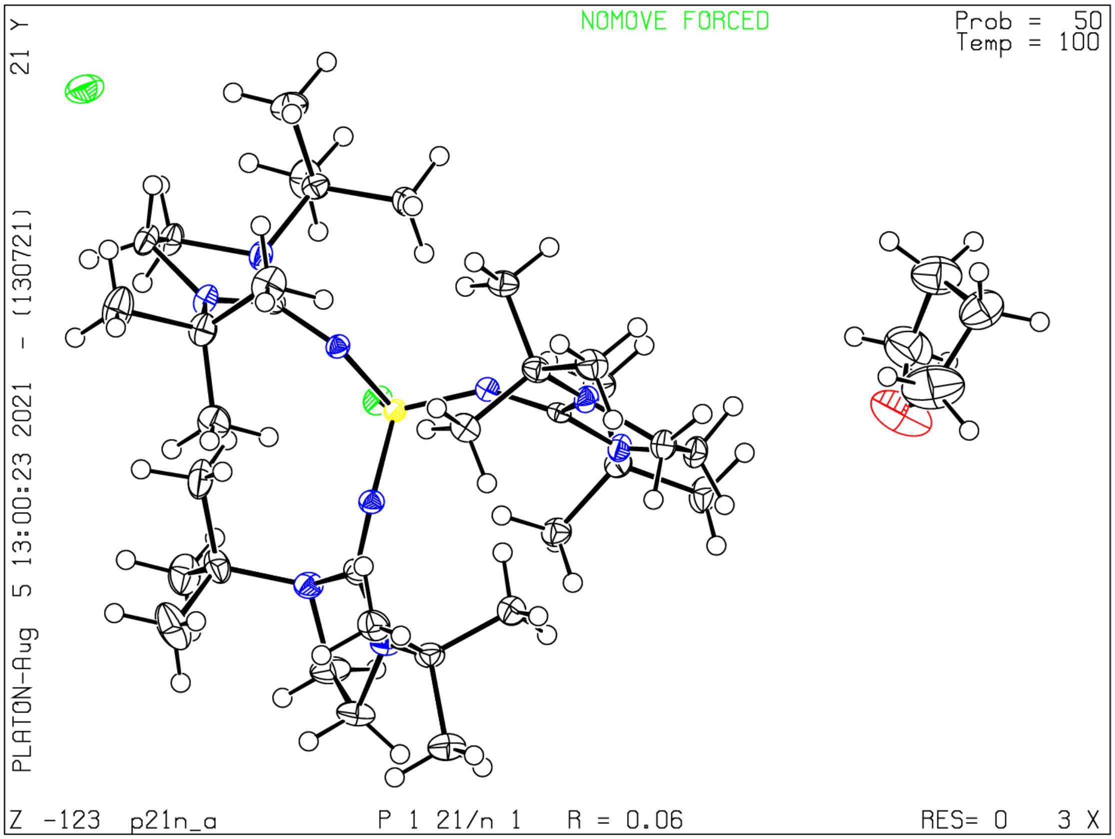

checkCIF/PLATON report

You have not supplied any structure factors. As a result the full set of tests cannot be run.

THIS REPORT IS FOR GUIDANCE ONLY. IF USED AS PART OF A REVIEW PROCEDURE FOR PUBLICATION, IT SHOULD NOT REPLACE THE EXPERTISE OF AN EXPERIENCED CRYSTALLOGRAPHIC REFEREE.

No syntax errors found. CIF dictionary Interpreting this report

Datablock: compound_11

```
Bond precision:   C-C = 0.0044 A           Wavelength=0.71073

Cell:           a=10.4679(6)       b=11.8606(7)      c=20.8708(13)
                alpha=80.210(2)    beta=83.116(2)    gamma=76.388(2)
Temperature:    153 K
```

	Calculated	Reported
Volume	2473.1(3)	2473.1(3)
Space group	P -1	P -1
Hall group	-P 1	-P 1
Moiety formula	C41 H72 Cl Ir N9 P [+ solvent]	C41 H72 Cl Ir N9 P
Sum formula	C41 H72 Cl Ir N9 P [+ solvent]	C41 H72 Cl Ir N9 P
Mr	949.72	949.69
Dx,g cm-3	1.275	1.275
Z	2	2
Mu (mm-1)	2.821	2.821
F000	980.0	980.0
F000'	977.87	
h,k,lmax	13,14,26	13,14,26
Nref	10224	10185
Tmin,Tmax	0.448,0.693	0.528,0.745
Tmin'	0.369	

```
Correction method= # Reported T Limits: Tmin=0.528 Tmax=0.745
AbsCorr = MULTI-SCAN

Data completeness= 0.996            Theta(max)= 26.484
```

R(reflections)= 0.0232(9143) wR2(reflections)= 0.0510(10185)

S = 1.025 Npar= 496

The following ALERTS were generated. Each ALERT has the format
test-name_ALERT_alert-type_alert-level.
Click on the hyperlinks for more details of the test.

Alert level C

```
PLAT220_ALERT_2_C NonSolvent   Resd 1  C   Ueq(max)/Ueq(min) Range        4.1 Ratio
PLAT242_ALERT_2_C Low    'MainMol' Ueq as Compared to Neighbors of        C14 Check
PLAT242_ALERT_2_C Low    'MainMol' Ueq as Compared to Neighbors of        C17 Check
```

Alert level G

```
PLAT013_ALERT_1_G N.O.K.   _shelx_hkl_checksum Found in CIF ......     Please Check
PLAT154_ALERT_1_G The s.u.'s on the Cell Angles are Equal ..(Note)      0.002 Degree
PLAT606_ALERT_4_G Solvent Accessible VOID(S) in Structure ........          ! Info
PLAT868_ALERT_4_G ALERTS Due to the Use of _smtbx_masks Suppressed          ! Info
PLAT933_ALERT_2_G Number of OMIT Records in Embedded .res File ...         16 Note
```

---

0 **ALERT level A** = Most likely a serious problem - resolve or explain
0 **ALERT level B** = A potentially serious problem, consider carefully
3 **ALERT level C** = Check. Ensure it is not caused by an omission or oversight
5 **ALERT level G** = General information/check it is not something unexpected

2 ALERT type 1 CIF construction/syntax error, inconsistent or missing data
4 ALERT type 2 Indicator that the structure model may be wrong or deficient
0 ALERT type 3 Indicator that the structure quality may be low
2 ALERT type 4 Improvement, methodology, query or suggestion
0 ALERT type 5 Informative message, check

It is advisable to attempt to resolve as many as possible of the alerts in all categories. Often the minor alerts point to easily fixed oversights, errors and omissions in your CIF or refinement strategy, so attention to these fine details can be worthwhile. In order to resolve some of the more serious problems it may be necessary to carry out additional measurements or structure refinements. However, the purpose of your study may justify the reported deviations and the more serious of these should normally be commented upon in the discussion or experimental section of a paper or in the "special_details" fields of the CIF. checkCIF was carefully designed to identify outliers and unusual parameters, but every test has its limitations and alerts that are not important in a particular case may appear. Conversely, the absence of alerts does not guarantee there are no aspects of the results needing attention. It is up to the individual to critically assess their own results and, if necessary, seek expert advice.

Publication of your CIF in IUCr journals

A basic structural check has been run on your CIF. These basic checks will be run on all CIFs submitted for publication in IUCr journals (*Acta Crystallographica*, *Journal of Applied Crystallography*, *Journal of Synchrotron Radiation*); however, if you intend to submit to *Acta Crystallographica Section C* or *E* or *IUCrData*, you should make sure that full publication checks are run on the final version of your CIF prior to submission.

Publication of your CIF in other journals

Please refer to the *Notes for Authors* of the relevant journal for any special instructions relating to CIF submission.

PLATON version of 13/07/2021; check.def file version of 13/07/2021

Datablock compound_11 - ellipsoid plot

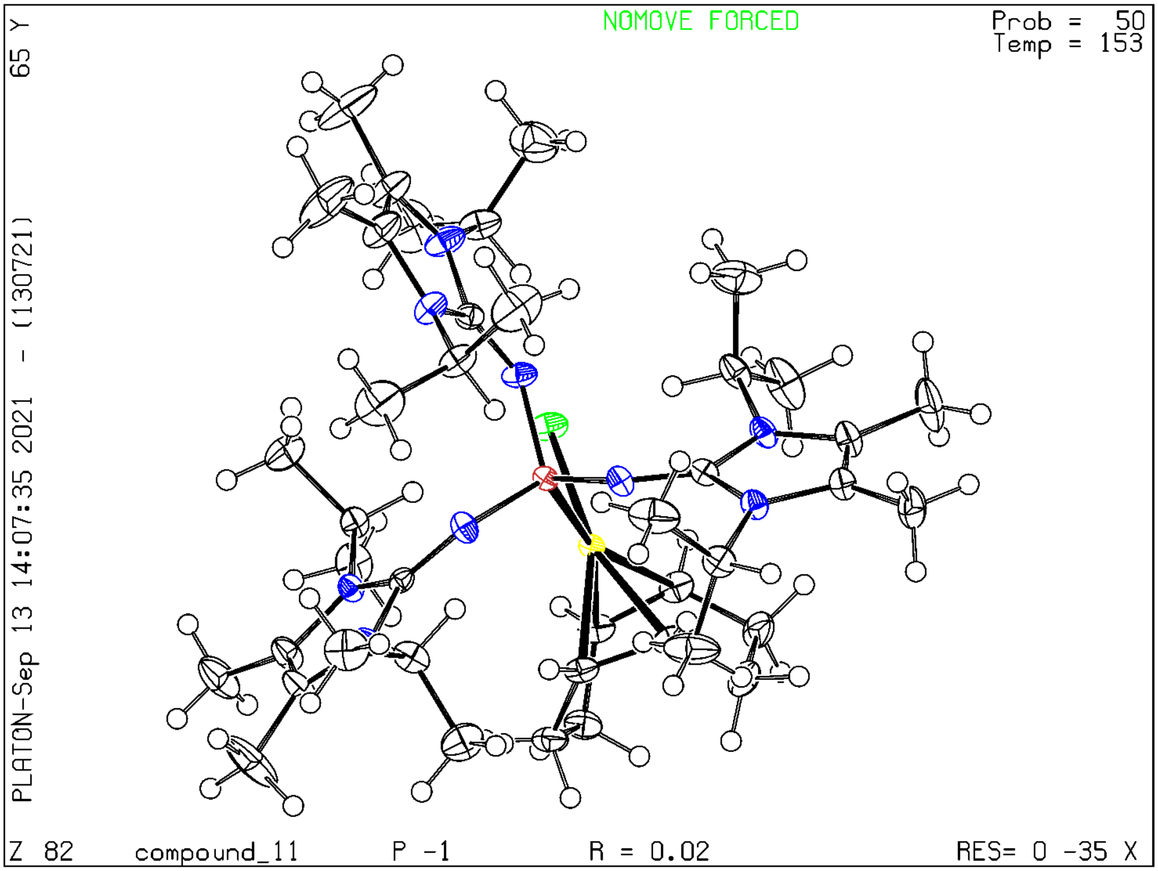

checkCIF/PLATON report

Structure factors have been supplied for datablock(s) p2n_a

THIS REPORT IS FOR GUIDANCE ONLY. IF USED AS PART OF A REVIEW PROCEDURE FOR PUBLICATION, IT SHOULD NOT REPLACE THE EXPERTISE OF AN EXPERIENCED CRYSTALLOGRAPHIC REFEREE.

No syntax errors found.   CIF dictionary   Interpreting this report

Datablock: p2n_a

```
Bond precision:   C-C = 0.0077 A          Wavelength=0.71073

Cell:             a=7.3017(2)     b=24.7872(8)       c=19.3229(6)
                  alpha=90        beta=90.715(2)     gamma=90
Temperature:      100 K

                Calculated                 Reported
Volume          3496.96(18)                3496.95(18)
Space group     P 21/n                     P 1 21/n 1
Hall group      -P 2yn                     -P 2yn
Moiety formula  C40 H40 Cl3 Ir N4          C40 H40 Cl3 Ir N4
Sum formula     C40 H40 Cl3 Ir N4          C40 H40 Cl3 Ir N4
Mr              875.33                     875.31
Dx,g cm-3       1.663                      1.663
Z               4                          4
Mu (mm-1)       4.083                      4.083
F000            1744.0                     1744.0
F000'           1740.56
h,k,lmax        9,32,24                    9,31,24
Nref            7895                       7793
Tmin,Tmax       0.488,0.651                0.780,1.000
Tmin'           0.479

Correction method= # Reported T Limits: Tmin=0.780 Tmax=1.000
AbsCorr = MULTI-SCAN

Data completeness= 0.987          Theta(max)= 27.312

R(reflections)= 0.0404( 6568)     wR2(reflections)= 0.1052( 7793)

S = 1.086                   Npar= 439
```

The following ALERTS were generated. Each ALERT has the format
test-name_ALERT_alert-type_alert-level.
Click on the hyperlinks for more details of the test.

Alert level B

```
PLAT094_ALERT_2_B Ratio of Maximum / Minimum Residual Density ....       4.26 Report
PLAT973_ALERT_2_B Check Calcd Positive Resid. Density on       Ir1       1.69 eA-3
```

Alert level C

```
PLAT906_ALERT_3_C Large K Value in the Analysis of Variance ......      2.075 Check
PLAT911_ALERT_3_C Missing FCF Refl Between Thmin & STh/L=    0.600          9 Report
PLAT971_ALERT_2_C Check Calcd Resid. Dens.  0.79A   From Ir1             2.44 eA-3
PLAT971_ALERT_2_C Check Calcd Resid. Dens.  0.95A   From Ir1             2.08 eA-3
PLAT971_ALERT_2_C Check Calcd Resid. Dens.  0.93A   From Ir1             1.91 eA-3
PLAT971_ALERT_2_C Check Calcd Resid. Dens.  0.99A   From Ir1             1.84 eA-3
PLAT971_ALERT_2_C Check Calcd Resid. Dens.  0.94A   From Ir1             1.54 eA-3
```

Alert level G

```
PLAT083_ALERT_2_G SHELXL Second Parameter in WGHT  Unusually Large       8.06 Why ?
PLAT380_ALERT_4_G Incorrectly? Oriented X(sp2)-Methyl Moiety .....        C43 Check
PLAT432_ALERT_2_G Short Inter X...Y Contact  Cl3       ..C19             3.24 Ang.
                                                     1+x,y,z  =      1_655 Check
PLAT910_ALERT_3_G Missing # of FCF Reflection(s) Below Theta(Min).          1 Note
PLAT912_ALERT_4_G Missing # of FCF Reflections Above STh/L=  0.600         92 Note
PLAT933_ALERT_2_G Number of OMIT Records in Embedded .res File ...          2 Note
PLAT941_ALERT_3_G Average HKL Measurement Multiplicity ...........        4.2 Low
PLAT978_ALERT_2_G Number C-C Bonds with Positive Residual Density.          1 Info
```

```
   0 ALERT level A = Most likely a serious problem - resolve or explain
   2 ALERT level B = A potentially serious problem, consider carefully
   7 ALERT level C = Check. Ensure it is not caused by an omission or oversight
   8 ALERT level G = General information/check it is not something unexpected

   0 ALERT type 1 CIF construction/syntax error, inconsistent or missing data
  11 ALERT type 2 Indicator that the structure model may be wrong or deficient
   4 ALERT type 3 Indicator that the structure quality may be low
   2 ALERT type 4 Improvement, methodology, query or suggestion
   0 ALERT type 5 Informative message, check
```

It is advisable to attempt to resolve as many as possible of the alerts in all categories. Often the minor alerts point to easily fixed oversights, errors and omissions in your CIF or refinement strategy, so attention to these fine details can be worthwhile. In order to resolve some of the more serious problems it may be necessary to carry out additional measurements or structure refinements. However, the purpose of your study may justify the reported deviations and the more serious of these should normally be commented upon in the discussion or experimental section of a paper or in the "special_details" fields of the CIF. checkCIF was carefully designed to identify outliers and unusual parameters, but every test has its limitations and alerts that are not important in a particular case may appear. Conversely, the absence of alerts does not guarantee there are no aspects of the results needing attention. It is up to the individual to critically assess their own results and, if necessary, seek expert advice.

Publication of your CIF in IUCr journals

A basic structural check has been run on your CIF. These basic checks will be run on all CIFs submitted for publication in IUCr journals (*Acta Crystallographica*, *Journal of Applied Crystallography*, *Journal of Synchrotron Radiation*); however, if you intend to submit to *Acta Crystallographica Section C* or *E* or *IUCrData*, you should make sure that full publication checks are run on the final version of your CIF prior to submission.

Publication of your CIF in other journals

Please refer to the *Notes for Authors* of the relevant journal for any special instructions relating to CIF submission.

PLATON version of 22/03/2021; check.def file version of 19/03/2021

Datablock p2n_a - ellipsoid plot

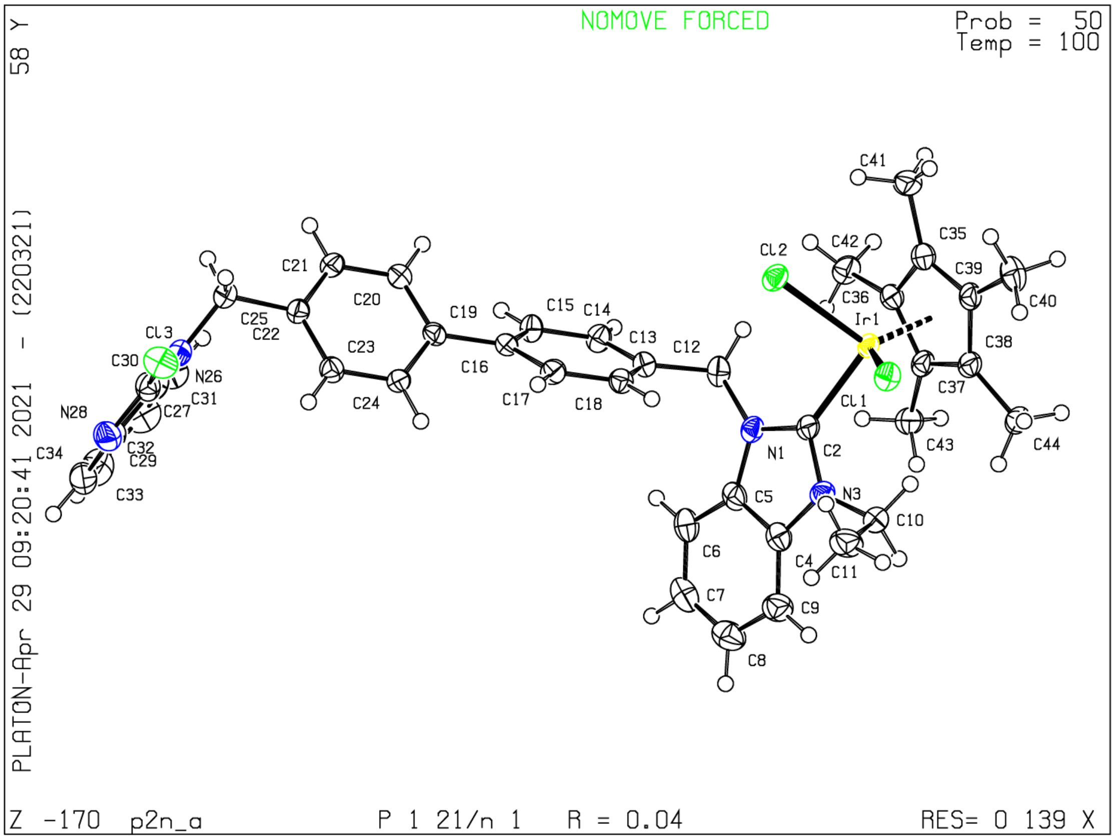

checkCIF/PLATON report

Structure factors have been supplied for datablock(s) p2n_a

THIS REPORT IS FOR GUIDANCE ONLY. IF USED AS PART OF A REVIEW PROCEDURE FOR PUBLICATION, IT SHOULD NOT REPLACE THE EXPERTISE OF AN EXPERIENCED CRYSTALLOGRAPHIC REFEREE.

No syntax errors found.   CIF dictionary   Interpreting this report

Datablock: p2n_a

```
Bond precision:  C-C = 0.0056 A          Wavelength=0.71073

Cell:            a=7.2839(3)     b=24.8808(11)      c=19.3651(8)
                 alpha=90        beta=91.103(3)     gamma=90
Temperature:     150 K

                Calculated                  Reported
Volume          3508.9(3)                   3508.9(3)
Space group     P 21/n                      P 1 21/n 1
Hall group      -P 2yn                      -P 2yn
Moiety formula  C40 H40 Cl3 N4 Rh           C40 H40 Cl3 N4 Rh
Sum formula     C40 H40 Cl3 N4 Rh           C40 H40 Cl3 N4 Rh
Mr              786.02                      786.02
Dx,g cm-3       1.488                       1.488
Z               4                           4
Mu (mm-1)       0.751                       0.751
F000            1616.0                      1616.0
F000'           1613.72
h,k,lmax        9,32,25                     9,32,24
Nref            7981                        7881
Tmin,Tmax       0.888,0.924                 0.735,1.000
Tmin'           0.879

Correction method= # Reported T Limits: Tmin=0.735 Tmax=1.000
AbsCorr = MULTI-SCAN

Data completeness= 0.987          Theta(max)= 27.391

R(reflections)= 0.0490( 5853)     wR2(reflections)= 0.1310( 7881)

S = 1.067                 Npar= 439
```

The following ALERTS were generated. Each ALERT has the format
test-name_ALERT_alert-type_alert-level.
Click on the hyperlinks for more details of the test.

Alert level C

```
PLAT094_ALERT_2_C Ratio of Maximum / Minimum Residual Density ....       2.17 Report
PLAT906_ALERT_3_C Large K Value in the Analysis of Variance ......      2.882 Check
PLAT911_ALERT_3_C Missing FCF Refl Between Thmin & STh/L=    0.600          3 Report
```

Alert level G

```
PLAT910_ALERT_3_G Missing # of FCF Reflection(s) Below Theta(Min).          2 Note
PLAT912_ALERT_4_G Missing # of FCF Reflections Above STh/L=  0.600         94 Note
PLAT933_ALERT_2_G Number of OMIT Records in Embedded .res File ...          3 Note
PLAT978_ALERT_2_G Number C-C Bonds with Positive Residual Density.          1 Info
```

```
   0 ALERT level A = Most likely a serious problem - resolve or explain
   0 ALERT level B = A potentially serious problem, consider carefully
   3 ALERT level C = Check. Ensure it is not caused by an omission or oversight
   4 ALERT level G = General information/check it is not something unexpected

   0 ALERT type 1 CIF construction/syntax error, inconsistent or missing data
   3 ALERT type 2 Indicator that the structure model may be wrong or deficient
   3 ALERT type 3 Indicator that the structure quality may be low
   1 ALERT type 4 Improvement, methodology, query or suggestion
   0 ALERT type 5 Informative message, check
```

It is advisable to attempt to resolve as many as possible of the alerts in all categories. Often the minor alerts point to easily fixed oversights, errors and omissions in your CIF or refinement strategy, so attention to these fine details can be worthwhile. In order to resolve some of the more serious problems it may be necessary to carry out additional measurements or structure refinements. However, the purpose of your study may justify the reported deviations and the more serious of these should normally be commented upon in the discussion or experimental section of a paper or in the "special_details" fields of the CIF. checkCIF was carefully designed to identify outliers and unusual parameters, but every test has its limitations and alerts that are not important in a particular case may appear. Conversely, the absence of alerts does not guarantee there are no aspects of the results needing attention. It is up to the individual to critically assess their own results and, if necessary, seek expert advice.

Publication of your CIF in IUCr journals

A basic structural check has been run on your CIF. These basic checks will be run on all CIFs submitted for publication in IUCr journals (*Acta Crystallographica*, *Journal of Applied Crystallography*, *Journal of Synchrotron Radiation*); however, if you intend to submit to *Acta Crystallographica Section C* or *E* or *IUCrData*, you should make sure that full publication checks are run on the final version of your CIF prior to submission.

Publication of your CIF in other journals

Please refer to the *Notes for Authors* of the relevant journal for any special instructions relating to CIF submission.

PLATON version of 13/07/2021; check.def file version of 13/07/2021

Datablock p2n_a - ellipsoid plot

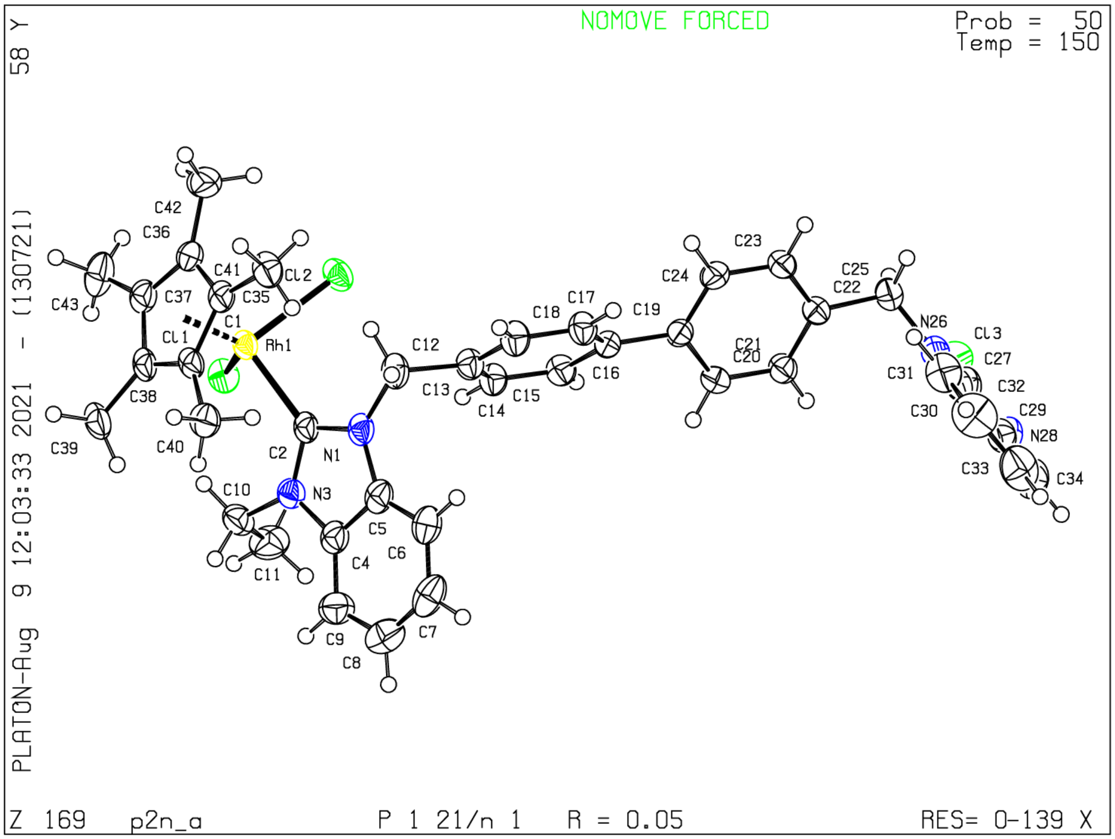

checkCIF/PLATON report

Structure factors have been supplied for datablock(s) p2c_a

THIS REPORT IS FOR GUIDANCE ONLY. IF USED AS PART OF A REVIEW PROCEDURE FOR PUBLICATION, IT SHOULD NOT REPLACE THE EXPERTISE OF AN EXPERIENCED CRYSTALLOGRAPHIC REFEREE.

No syntax errors found. CIF dictionary Interpreting this report

Datablock: p2c_a

Bond precision: C-C = 0.0065 A Wavelength=0.71073

Cell:	a=9.4335(6)	b=20.1853(10)	c=18.5268(10)
	alpha=90	beta=99.013(4)	gamma=90
Temperature:	100 K		

	Calculated	Reported
Volume	3484.3(3)	3484.3(3)
Space group	P 21/c	P 1 21/c 1
Hall group	-P 2ybc	-P 2ybc
Moiety formula	C38 H37 Cl2 N4 Rh [+ solvent]	C38 H37 Cl2 N4 Rh, [+ solventS]
Sum formula	C38 H37 Cl2 N4 Rh [+ solvent]	C38 H37 Cl2 N4 Rh
Mr	723.53	723.52
Dx,g cm-3	1.379	1.379
Z	4	4
Mu (mm-1)	0.676	0.676
F000	1488.0	1488.0
F000'	1485.10	
h,k,lmax	11,25,23	11,25,23
Nref	7420	7318
Tmin,Tmax	0.915,0.934	0.598,1.000
Tmin'	0.706	

Correction method= # Reported T Limits: Tmin=0.598 Tmax=1.000
AbsCorr = MULTI-SCAN

Data completeness= 0.986 Theta(max)= 26.768

R(reflections)= 0.0569(5808) wR2(reflections)= 0.1434(7318)

S = 1.072 Npar= 407

The following ALERTS were generated. Each ALERT has the format
test-name_ALERT_alert-type_alert-level.
Click on the hyperlinks for more details of the test.

Alert level C

```
PLAT094_ALERT_2_C Ratio of Maximum / Minimum Residual Density ....       2.05 Report
PLAT213_ALERT_2_C Atom C36             has ADP max/min Ratio .....        3.2 prolat
PLAT220_ALERT_2_C NonSolvent   Resd 1  C   Ueq(max)/Ueq(min) Range        3.8 Ratio
PLAT241_ALERT_2_C High   'MainMol' Ueq as Compared to Neighbors of        C33 Check
PLAT241_ALERT_2_C High   'MainMol' Ueq as Compared to Neighbors of        C36 Check
PLAT242_ALERT_2_C Low    'MainMol' Ueq as Compared to Neighbors of        C37 Check
PLAT906_ALERT_3_C Large K Value in the Analysis of Variance ......      4.435 Check
PLAT911_ALERT_3_C Missing FCF Refl Between Thmin & STh/L=    0.600          6 Report
PLAT977_ALERT_2_C Check Negative Difference Density on H37A             -0.36 eA-3
```

Alert level G

```
PLAT002_ALERT_2_G Number of Distance or Angle Restraints on AtSite          8 Note
PLAT003_ALERT_2_G Number of Uiso or Uij Restrained non-H Atoms ...          8 Report
PLAT083_ALERT_2_G SHELXL Second Parameter in WGHT  Unusually Large       9.21 Why ?
PLAT176_ALERT_4_G The CIF-Embedded .res File Contains SADI Records          1 Report
PLAT177_ALERT_4_G The CIF-Embedded .res File Contains DELU Records          1 Report
PLAT187_ALERT_4_G The CIF-Embedded .res File Contains RIGU Records          1 Report
PLAT605_ALERT_4_G Largest Solvent Accessible VOID in the Structure        142 A**3
PLAT860_ALERT_3_G Number of Least-Squares Restraints ............          70 Note
PLAT910_ALERT_3_G Missing # of FCF Reflection(s) Below Theta(Min).          2 Note
PLAT912_ALERT_4_G Missing # of FCF Reflections Above STh/L=  0.600         95 Note
PLAT933_ALERT_2_G Number of OMIT Records in Embedded .res File ...          4 Note
PLAT978_ALERT_2_G Number C-C Bonds with Positive Residual Density.          2 Info
```

---

0 **ALERT level A** = Most likely a serious problem - resolve or explain
0 **ALERT level B** = A potentially serious problem, consider carefully
9 **ALERT level C** = Check. Ensure it is not caused by an omission or oversight
12 **ALERT level G** = General information/check it is not something unexpected

0 ALERT type 1 CIF construction/syntax error, inconsistent or missing data
12 ALERT type 2 Indicator that the structure model may be wrong or deficient
4 ALERT type 3 Indicator that the structure quality may be low
5 ALERT type 4 Improvement, methodology, query or suggestion
0 ALERT type 5 Informative message, check

It is advisable to attempt to resolve as many as possible of the alerts in all categories. Often the minor alerts point to easily fixed oversights, errors and omissions in your CIF or refinement strategy, so attention to these fine details can be worthwhile. In order to resolve some of the more serious problems it may be necessary to carry out additional measurements or structure refinements. However, the purpose of your study may justify the reported deviations and the more serious of these should normally be commented upon in the discussion or experimental section of a paper or in the "special_details" fields of the CIF. checkCIF was carefully designed to identify outliers and unusual parameters, but every test has its limitations and alerts that are not important in a particular case may appear. Conversely, the absence of alerts does not guarantee there are no aspects of the results needing attention. It is up to the individual to critically assess their own results and, if necessary, seek expert advice.

Publication of your CIF in IUCr journals

A basic structural check has been run on your CIF. These basic checks will be run on all CIFs submitted for publication in IUCr journals (*Acta Crystallographica*, *Journal of Applied Crystallography*, *Journal of Synchrotron Radiation*); however, if you intend to submit to *Acta Crystallographica Section C* or *E* or *IUCrData*, you should make sure that full publication checks are run on the final version of your CIF prior to submission.

Publication of your CIF in other journals

Please refer to the *Notes for Authors* of the relevant journal for any special instructions relating to CIF submission.

PLATON version of 13/07/2021; check.def file version of 13/07/2021

Datablock p2c_a - ellipsoid plot

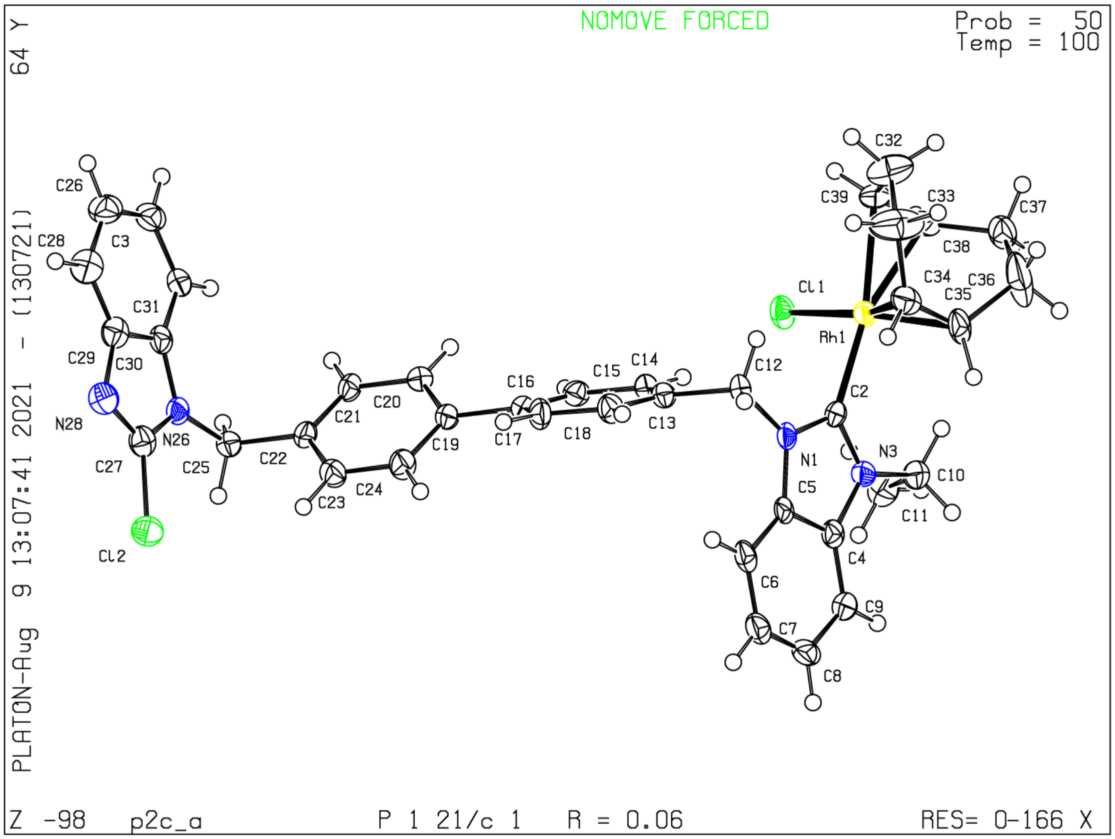

checkCIF/PLATON report

Structure factors have been supplied for datablock(s) p2c_a

THIS REPORT IS FOR GUIDANCE ONLY. IF USED AS PART OF A REVIEW PROCEDURE FOR PUBLICATION, IT SHOULD NOT REPLACE THE EXPERTISE OF AN EXPERIENCED CRYSTALLOGRAPHIC REFEREE.

No syntax errors found. CIF dictionary Interpreting this report

Datablock: p2c_a

```
Bond precision:  C-C = 0.0031 A          Wavelength=0.71073

Cell:           a=11.4755(3)     b=14.0115(3)        c=16.6181(4)
                alpha=90         beta=105.898(1)     gamma=90
Temperature:    100 K

                Calculated                    Reported
Volume          2569.81(11)                   2569.80(11)
Space group     P 2/c                         P 1 2/c 1
Hall group      -P 2yc                        -P 2yc
Moiety formula  C30 H25 Cl3 N4 Ru             C30 H25 Cl3 N4 Ru
Sum formula     C30 H25 Cl3 N4 Ru             C30 H25 Cl3 N4 Ru
Mr              648.96                        648.96
Dx,g cm-3       1.677                         1.677
Z               4                             4
Mu (mm-1)       0.952                         0.952
F000            1312.0                        1312.0
F000'           1309.05
h,k,lmax        14,17,21                      14,17,21
Nref            5691                          5681
Tmin,Tmax       0.921,0.952                   0.859,1.000
Tmin'           0.791

Correction method= # Reported T Limits: Tmin=0.859 Tmax=1.000
AbsCorr = NONE

Data completeness= 0.998          Theta(max)= 27.128

R(reflections)= 0.0269( 5247)     wR2(reflections)= 0.0721( 5681)

S = 1.057                   Npar= 344
```

The following ALERTS were generated. Each ALERT has the format
test-name_ALERT_alert-type_alert-level.
Click on the hyperlinks for more details of the test.

Alert level C

```
PLAT094_ALERT_2_C Ratio of Maximum / Minimum Residual Density ....       2.50 Report
PLAT230_ALERT_2_C Hirshfeld Test Diff for    C32      --C33      .        6.5 s.u.
PLAT911_ALERT_3_C Missing FCF Refl Between Thmin & STh/L=    0.600          3 Report
PLAT971_ALERT_2_C Check Calcd Resid. Dens.  1.30A   From C34             1.52 eA-3
PLAT971_ALERT_2_C Check Calcd Resid. Dens.  0.71A   From C32             1.51 eA-3
```

Alert level G

```
PLAT003_ALERT_2_G Number of Uiso or Uij Restrained non-H Atoms ...          2 Report
PLAT177_ALERT_4_G The CIF-Embedded .res File Contains DELU Records          1 Report
PLAT178_ALERT_4_G The CIF-Embedded .res File Contains SIMU Records          1 Report
PLAT186_ALERT_4_G The CIF-Embedded .res File Contains ISOR Records          1 Report
PLAT187_ALERT_4_G The CIF-Embedded .res File Contains RIGU Records          1 Report
PLAT232_ALERT_2_G Hirshfeld Test Diff (M-X)  Ru1      --Cl1      .        7.0 s.u.
PLAT232_ALERT_2_G Hirshfeld Test Diff (M-X)  Ru1      --Cl2      .        6.0 s.u.
PLAT333_ALERT_2_G Large Aver C6-Ring C-C Dist C13      -C18      .       1.42 Ang.
PLAT860_ALERT_3_G Number of Least-Squares Restraints ............          16 Note
PLAT910_ALERT_3_G Missing # of FCF Reflection(s) Below Theta(Min).          2 Note
PLAT912_ALERT_4_G Missing # of FCF Reflections Above STh/L=  0.600          6 Note
PLAT933_ALERT_2_G Number of OMIT Records in Embedded .res File ...          3 Note
PLAT978_ALERT_2_G Number C-C Bonds with Positive Residual Density.         14 Info
```

0 **ALERT level A** = Most likely a serious problem - resolve or explain
0 **ALERT level B** = A potentially serious problem, consider carefully
5 **ALERT level C** = Check. Ensure it is not caused by an omission or oversight
13 **ALERT level G** = General information/check it is not something unexpected

0 ALERT type 1 CIF construction/syntax error, inconsistent or missing data
10 ALERT type 2 Indicator that the structure model may be wrong or deficient
3 ALERT type 3 Indicator that the structure quality may be low
5 ALERT type 4 Improvement, methodology, query or suggestion
0 ALERT type 5 Informative message, check

It is advisable to attempt to resolve as many as possible of the alerts in all categories. Often the minor alerts point to easily fixed oversights, errors and omissions in your CIF or refinement strategy, so attention to these fine details can be worthwhile. In order to resolve some of the more serious problems it may be necessary to carry out additional measurements or structure refinements. However, the purpose of your study may justify the reported deviations and the more serious of these should normally be commented upon in the discussion or experimental section of a paper or in the "special_details" fields of the CIF. checkCIF was carefully designed to identify outliers and unusual parameters, but every test has its limitations and alerts that are not important in a particular case may appear. Conversely, the absence of alerts does not guarantee there are no aspects of the results needing attention. It is up to the individual to critically assess their own results and, if necessary, seek expert advice.

Publication of your CIF in IUCr journals

A basic structural check has been run on your CIF. These basic checks will be run on all CIFs submitted for publication in IUCr journals (*Acta Crystallographica*, *Journal of Applied Crystallography*, *Journal of Synchrotron Radiation*); however, if you intend to submit to *Acta Crystallographica Section C* or *E* or *IUCrData*, you should make sure that full publication checks are run on the final version of your CIF prior to submission.

Publication of your CIF in other journals

Please refer to the *Notes for Authors* of the relevant journal for any special instructions relating to CIF submission.

PLATON version of 13/07/2021; check.def file version of 13/07/2021

Datablock p2c_a - ellipsoid plot

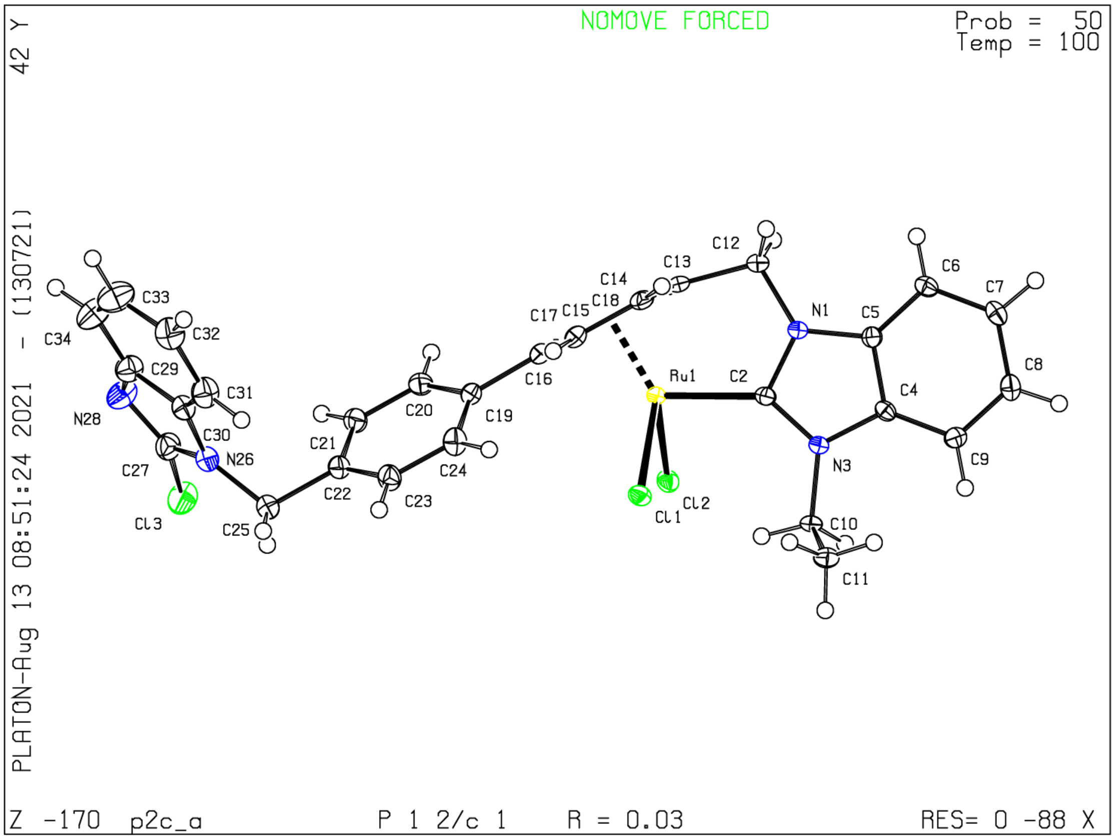

Supplement: Supplementary file 1 — Supporting Information [file ANIE-61-0-s001.pdf]
