# Supplementary material for: Synthesis of N‐Heterocyclic Carbenes and Their Complexes by Chloronium Ion Abstraction from 2‐Chloroazolium Salts Using Electron‐Rich Phosphines
Source: Angew Chem Int Ed Engl. 2022 May 17;61(28):e202202190. doi: 10.1002/anie.202202190 (PMC9401039; doi:10.1002/anie.202202190)
Supplement: Supplementary file 2 — Supporting Information [file ANIE-61-0-s002.pdf]

Angewandte Chemie
Eine Zeitschrift der Gesellschaft Deutscher Chemiker

Supporting Information

Synthesis of *N*-Heterocyclic Carbenes and Their Complexes by Chloronium Ion Abstraction from 2-Chloroazolium Salts Using Electron-Rich Phosphines

M. D. Böhme, T. Eder, M. B. Röthel, P. D. Dutschke, L. F. B. Wilm, F. E. Hahn, F. Dielmann**

Supplementary Information

Table of Contents

**1.**	**Experimental procedures**	**S3**
1.1.	General remarks	S3
1.2.	Synthesis of 1,3-di-*tert*-butylimidazolidine-2-iminium tetrafluoroborate ($R^1H\cdot HBF_4$)	S4
1.3.	Synthesis of 1,3-di-*tert*-butylimidazolidine-2-imine (R^1H)	S4
1.4.	Synthesis of 1,3-di-*tert*-butyl-*N*-(trimethylsilyl)imidazolidine-2-imine (R^1SiMe_3)	S5
1.5.	Synthesis of phosphenium salt (R^1_2P)Cl	S5
1.6.	Synthesis of phosphine **1**	S5
1.7.	Synthesis of the mixture of phosphines **1** and **1'** from the reaction of LiR^1 and PCl_3	S6
1.8.	Synthesis of the chlorophosphonium salt (**1**-Cl)Cl	S7
1.9.	Synthesis of the chlorophosphonium salt (**2**-H)Cl	S7
1.10.	Synthesis of the chlorophosphonium salt (Cy_3P-Cl)OTf	S7
1.11.	Synthesis of the cyclohex-1-en-1-yldicyclohexylphosphine **4**	S8
1.12.	Synthesis of 2-chloro-1,3-di-*iso*-propyl-4,5-dimethylimidazolium triflate (**3**-Cl)OTf	S8
1.13.	Synthesis of 2-chloro-1,3-di-*iso*-propylbenzimidazolium triflate (**5**-Cl)OTf	S9
1.14.	Synthesis of 1,3-di-*iso*-propylbenzimidazolin-2-ylidene **5**	S9
1.15.	Synthesis of 1,3-di-*iso*-propylbenzimidazolium triflate (**5**-H)OTf	S10
1.16.	Synthesis of 2-chloro-1,3-di-*tert*-butylimidazolium triflate (**7**-Cl)OTf	S10
1.17.	Synthesis of 2-chloro-1,3-di-*tert*-butylimidazolidinium tetrafluoroborate (**8**-Cl)BF_4	S10
1.18.	Reaction of the chloroimidazolium salt (**3**-Cl)OTf with IAPs **1** and **2**	S11
	1.18.1. Analytical data of (**1**-Cl)OTf	S12
	1.18.2. Analytical data for (**2**-Cl)OTf	S12
1.19.	Reaction of triphenylphosphine with (**3**-Cl)OTf	S12
1.20.	Reaction of tri-*tert*-butylphosphine with (**3**-Cl)OTf	S12
1.21.	Reaction of the chloroimidazolium salts (**3**-Cl)BF_4 or (**3**-Cl)OTf with tricyclohexylphosphine PCy_3	S14

1.22. Chloronium ion abstraction from 2-chlorobenzimidazolium *vs* 2-chlorbenzimidazole using phosphines **1** and **2** S16
1.23. Chloronium ion abstraction *vs* C2-deprotonation from 2-chlorinated and 2-protonated benzimidazolium salts with phosphines **1** and **2** S17
1.24. Chloronium ion abstraction from salts (**7**-Cl)OTf and (**8**-Cl)BF_4 with IAP **1** S19
1.25. Dechlorination of (**9**-Cl)PF_6 with IAP **1** in the presence of [$IrCl_2Cp^*$] S19
1.25.1. Synthesis of [Ir(**9**)Cl_2Cp^*] [**10**] S20
1.26. Reactivity of phosphines **1** and **2** towards iridium(I) and iridium(III) complexes S20
1.26.1. Synthesis of complex [**11**] S21
1.27. Synthesis of complex [**13**] S22
1.28. Synthesis of complex [**14**] S22
1.29. Synthesis of complex [**15**] S23
1.30. Synthesis of complex [**16**] S24
1.31. Synthesis of complex [**17**] S24
1.32. Synthesis of complex [**18**]$(BF_4)_2$ S25

2. X-ray crystallography S27
2.1. General remarks S27
2.2. Crystallographic data for **1** S27
2.3. Crystallographic data for **1'** S28
2.4. Crystallographic data for (**1**-Cl)Cl·THF S28
2.5. Crystallographic data for (**1**-Cl)Cl S29
2.6. Crystallographic data for [**11**] S30
2.7. Crystallographic data for [**13**] S30
2.8. Crystallographic data for [**14]** S31
2.9. Crystallographic data for [**16**] S32
2.10. Crystallographic data for [**17'**] S33

3. NMR Spectra S34

4. References S102

1. Experimental procedures

1.1. General remarks

Unless noted otherwise, all manipulations were performed under an inert atmosphere of dry argon, using standard Schlenk and drybox techniques. Dry and oxygen-free solvents were employed. All glassware was oven-dried at 160 °C prior to use. ^{1}H, ^{13}C, ^{19}F, ^{31}P, and ^{29}Si NMR spectra were recorded at 300 K on Agilent DD2 600, Bruker AVANCE I 400, Bruker AVANCE III 400 or Bruker AVANCE II 200 spectrometers. Chemical shifts are given in parts per million (ppm) relative to $SiMe_4$ (^{1}H, ^{13}C, ^{29}Si), 85% H_3PO_4 (^{31}P), CCl_3F (^{19}F) and they were referenced to the residual solvent signals (C_6D_6: ^{1}H $\delta_H = 7.16$, ^{13}C $\delta_C = 128.06$; CD_3CN: $\delta_H = 1.94$, ^{13}C $\delta_C = 118.26$; $CDCl_3$: $\delta_H = 7.26$, ^{13}C $\delta_C = 77.16$; CD_2Cl_2: $\delta_H = 5.32$, ^{13}C $\delta_C = 53.84$; THF-d_8: ^{1}H $\delta_H = 1.72$, ^{13}C $\delta_C = 67.21$) or internally by the instrument after locking and shimming to the deuterated solvent (^{11}B, ^{19}F, ^{31}P). Chemical shifts (δ) are reported in ppm. NMR multiplicities are abbreviated as follows: s = singlet, d = doublet, t = triplet, p = pentet, sept = septet, m = multiplet, br = broad signal. Mass spectra were recorded using an Orbitrap LTQ XL (Thermo Scientific) spectrometer or an Orbitrap QExactive (Thermo Scientific) spectrometer. Elemental analyses were determined by the microanalytic laboratory of the Westfälische Wilhelms-Universität Münster.

Tris-(1,3-di-*iso*-propyl-4,5-dimethylimidazolin-2-ylidenamino)phosphine **2**,[S1] 1,3-di-*iso*-propyl-4,5-dimethylimidazolin-2-ylidene **3**,[S2] 2-chloro-1,3-di-*iso*-propyl-4,5-dimethyl-imidazolium tetrafluoroborate (**3**-Cl)BF_4,[S3] 2-chloro-1,3-di-*iso*-propyl-4,5-dimethyl-imidazolium chloride (**3**-Cl)Cl,[S4] 1,3-di-*iso*-propyl-benzimidazolium bromide,[S5] 2-chloro-1-*iso*-propyl-benzimidazole **6**,[S6] 2-chloro-1,3-di-*tert*-butyl-imidazolium chloride (**7**-Cl)Cl,[S7] 2-chloro-1,3-di-*tert*-butyl-imidazolidinium chloride (**8**-Cl)Cl,[S7] 2-chloro-1,3-dimethyl-imidazolidinium hexafluorophosphate (**9**-Cl)PF_6[S8] and (**11**-2Cl)(BF_4)[S9] were synthesized following literature procedures. All other compounds were purchased from commercial sources (Sigma Aldrich, Alfa Aesar, abcr GmbH, Chempur, Tokyo Chemical Industry). Phosphorous trichloride was purified by distillation. Trimethylsilyl chloride was dried over CaH_2 and distilled. Triphenylphosphine was sublimed before use. All other chemicals were used as received.

1.2. Synthesis of 1,3-di-*tert*-butylimidazolidine-2-iminium tetrafluoroborate $R^1H\cdot HBF_4$

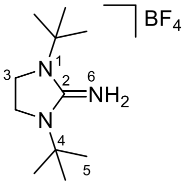

A solution of cyanogen bromide (10.00 g, 94.41 mmol) in toluene (30 mL) was added dropwise to a stirred solution of *N,N*-di-*tert*-butylethylene diamine (12.51 g, 72.60 mmol) in toluene (150 mL) heated to reflux. (*Note: The reaction is less selective if the toluene solution is not heated, or the solution is not stirred properly.)* After completed addition, the reaction mixture was heated to reflux for 10 hours leading to the precipitation of a white solid. After cooling the suspension to 21 °C, the solid was separated by filtration and washed with diethyl ether (3 × 40 mL). The solid residue was dissolved in water (20 mL) and a solution of $NaBF_4$ (23.92 g, 217.85 mmol) in water (40 mL) was added, resulting in the precipitation of $R^1H\cdot HBF_4$. The precipitate was collected and washed with ice-cold water (15 mL). A second fraction of $R^1H\cdot HBF_4$ was obtained from the combined aqueous solutions by evaporation of about one third of the water at ambient temperature. The formed crystals were collected by filtration and washed with ice-cold water (5 mL). The isolated solids were combined and dried at 80 °C *in vacuo*. Yield: 14.03 g (49.20 mmol, 68%). ^{1}H NMR (500 MHz, CD_3CN): δ = 6.02 (s, 2H, NH_2), 3.57 (s, 4H, H3), 1.40 (s,18H, H5). $^{13}C\{^1H\}$ NMR (101 MHz, CD_3CN): δ = 157.2 (C2), 56.0 (C4), 44.4 (C3), 28.0 (C5). ^{19}F NMR (471 MHz, CD_3CN): δ = –151.5. ^{11}B NMR (160 MHz, CD_3CN): δ = –1.2. HRMS (ESI, positive): *m/z* (%) = 198.1957 (100, calcd for $[R^1H+H]^+$ 198.1970). Elemental analysis: C 46.22, H 8.21, N 14.64. Calcd for $C_{11}H_{24}BF_4N_3$: C 46.33, H 8.48, N 14.74.

1.3. Synthesis of 1,3-di-*tert*-butylimidazolidine-2-imine R^1H

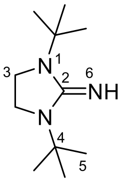

To a suspension of $R^1H\cdot HBF_4$ (33.00 g, 115.73 mmol) in diethyl ether (150 mL) was added an aqueous solution (75 mL) of KOH (12.99 g, 231.52 mmol) and the mixture was stirred vigorously for 1 hour at ambient temperature. After this reaction time, brine (50 ml) was added to the mixture and the organic and aqueous phase were separated. For better yield, the remaining amount of product in the aqueous phase was extracted with diethyl ether again (3 × 100 mL). The organic phases were combined and dried over $MgSO_4$. After filtration, the solvent was removed *in vacuo* to afford a yellowish oil, from which R^1H was isolated by sublimation (45 °C, 10^{-3} mbar) as a white, crystalline solid. Yield: 19.523 g (98.92 mmol, 85%). ^{1}H NMR (400 MHz, C_6D_6): δ = 5.32 (s br, 1H, NH), 2.80–2.62 (m, 4H, H3), 1.56 (s, 9H, H5), 1.09 (s, 9H, H5'). $^{13}C\{^1H\}$ NMR (101 MHz, C_6D_6): δ = 160.8 (C2), 53.7 (C4), 52.3 (C4'), 42.7 (C3), 42.1 (C3'), 27.9 (C5), 27.4 (C5'). The NMR spectroscopic data of the isolated R^1H are in agreement with those published.[10] HRMS (ESI, positive): *m/z* (%) = 198.1966 (100, calcd for $[R^1H+H]^+$ 198.1970).

1.4. Synthesis of 1,3-di-*tert*-butyl-*N*-(trimethylsilyl)imidazolidine-2-imine R^1SiMe_3

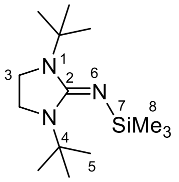

A 1.6 *M* hexane solution of *n*-butyl lithium (48.00 mmol, 30.00 mL) was slowly added to a stirred solution of R^1H (9.47 g, 47.99 mmol) in THF (50 mL) at –78 °C. The reaction mixture was kept at –78 °C for one hour and was then allowed to warm up to ambient temperature to give a clear solution. Then, trimethylsilyl chloride (5.43 g, 50.00 mmol, 6.35 mL) was added at –78 °C and the reaction mixture was stirred for 12 h at ambient temperature. The volatiles were removed *in vacuo* and the residue was extracted with *n*-hexane (60 mL). Evaporation of the *n*-hexane and subsequent purification by sublimation (55 °C at 10^{-3} mbar) gave R^1SiMe_3 as a colorless, crystalline solid. Yield: 12.03 g (44.64 mmol, 93%). ^1H NMR (500 MHz, C_6D_6): δ (ppm) = 2.75 (s, 4H, H3), 1.27 (s, 18H, H5), 0.44 (s, 9H, H8). $^{13}C\{^1H\}$ NMR (126 MHz, C_6D_6): δ (ppm) = 147.3 (C2), 52.7 (C4), 41.8 (C3), 27.8 (C5), 4.3 (C8). $^{29}Si\{^1H\}$DEPT (19.5) NMR (79 MHz, C_6D_6): δ (ppm) = –25.3. The NMR spectroscopic data of the isolated R^1SiMe_3 are in agreement with those published.[S10] HRMS (ESI, positive): *m/z* (%) = 198.1962 (100, calcd for $[R^1SiMe_3–SiMe_3+2H]^+$ 198.1970), 270.2359 (10, calcd. for $[R^1SiMe_3+H]^+$ 270.2366).

1.5. Synthesis of phosphenium salt $(R^1_2P)Cl$

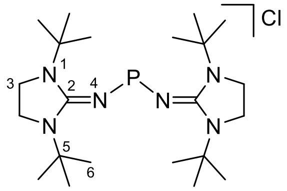

A sample of the N-heterocyclic imine R^1SiMe_3 (3.081 g, 11.433 mmol) was dissolved in THF (30 mL) and PCl_3 (0.5 mL, 0.785 g, 5.716 mmol) was added dropwise to the solution at –78 °C. The reaction mixture was allowed to warm up to ambient temperature and was stirred for 12 h. The volatiles were removed *in vacuo*. The residue was washed with THF (3 × 10 mL) and dried *in vacuo*. Phosphenium salt $(R^1_2P)Cl$ was obtained as a colorless solid. Yield: 2.623 g (5.714 mmol, 99%). ^1H NMR (400 MHz, CD_3CN): δ = 3.58 (s, 8H, H3), 1.43 (s, 36H, H6). $^{13}C\{^1H\}$ NMR (101 MHz, CD_3CN): δ = 159.2 (d, $^2J_{CP}$ = 21.5 Hz, C2), 56.4 (C5), 43.9 (C3), 29.5 (d, $^5J_{CP}$ = 3.7 Hz, C6). ^{31}P NMR (162 MHz, CD_3CN): δ = 238.2 (s). ^{35}Cl NMR (39 MHz, CD_3CN): δ = 41.7. HRMS (ESI, positive): *m/z* (%) = 423.3354 (100, calcd for $[R^1_2P]^+$ 423.3360). Elemental analysis: C 57.41, H 9.34, N 18.16. Calcd for $(R^1_2P)Cl$: C 57.56, H 9.66, N 18.31.

1.6. Synthesis of phosphine 1

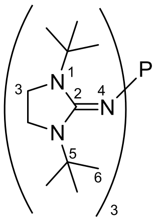

A 1.6 *M n*-hexane solution of *n*-butyl lithium (3.04 mmol, 1.9 mL) was added dropwise to a stirred solution of R^1H (0.601 g, 3.050 mmol) in THF (10 mL) at –78 °C. The reaction mixture was stirred for 30 min at –78 °C and then allowed to warm up to ambient temperature to give a clear solution, which was directly transferred into a stirred suspension of $(R^1_2P)Cl$ (1.400 g, 3.050 mmol) in THF (10 mL) at –78 °C. The reaction

mixture was stirred for 14 h at ambient temperature. The volatiles were then removed *in vacuo* and the residue was extracted with *n*-hexane (20 mL). Storing the saturated hexane solution at –40 °C led to the formation of colorless crystals of **1**, which were isolated by filtration and dried *in vacuo***.** Yield: 1.684 g (2.73 mmol, 90%). ^{1}H NMR (400 MHz, C_6D_6): δ = 2.87 (s, 12H, H3), 1.57 (s, 54H, H6). ^{13}C{^{1}H} NMR (101 MHz, C_6D_6): δ = 146.6 (d, $^2J_{CP}$ = 24.2 Hz, C2), 53.2 (C5), 42.1 (C3), 28.7 (d, $^5J_{CP}$ = 8.6 Hz, C6). ^{31}P NMR (162 MHz, C_6D_6): δ = 84.3. HRMS (ESI, positive): *m/z* (%) = 620.5237 (100, calcd for [**1**+H]$^+$ 620.5252). Elemental analysis: C 63.74, H 10.48, N 20.10. Calcd for **1**: C 63.93, H 10.73, N 20.34.

1.7. Synthesis of the mixture of phosphines 1 and 1' from the reaction of LiR^1 and PCl_3

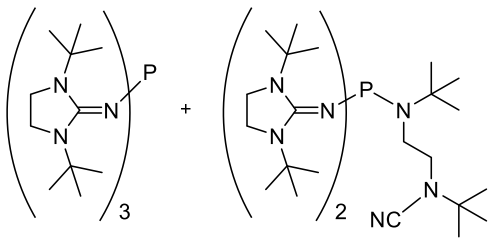

A solution of R^{1}H (677 mg, 3.430 mmol) in THF (10 mL) was cooled to –78 °C and a 1.6 *M n*-hexane solution of *n*-butyl lithium (3.42 mmol, 2.14 mL) was added dropwise. The reaction mixture was stirred at –78 °C for 1 h. Subsequently, PCl_3 (157 mg, 0.1 mL, 1.14 mmol) was added slowly and the mixture was stirred at –78 °C for 30 min. The mixture was then allowed to warm up to ambient temperature and stirred for additional 12 h. The solvents were removed *in vacuo* and the mixture was extracted with *n*-hexane (20 mL). The volume of the *n*-hexane filtrate was reduced to give a saturated solution containing **1** and **1'**, which were separated by fractional crystallization at –40 °C (first fraction **1'**). Yield for phosphine **1'** 128 mg, 0.206 mmol, 18%, yield for phosphine **1** 170 mg, 0.274 mmol, 24%.

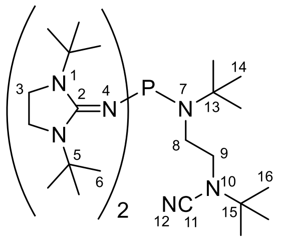

Note: The NMR signals for **1'** *were assigned using 2D NMR experiments. The data recorded for phosphine* ***1*** *obtained from the mixture of phosphines are identical to those reported in section 1.6.* NMR data for **1'**: ^{1}H NMR (400 MHz, C_6D_6): δ = 3.91–3.84 (m, 2H, H8), 3.12–3.04 (m, 2H, H9), 2.80 (s, 8H, H3), 1.49–145 (m, 45H, H6, H14), 1.10 (s, 9H, H16). ^{13}C{^{1}H} NMR (101 MHz, C_6D_6,): δ = 151.2 (d, $^2J_{CP}$ = 29.3 Hz, C2), 115.9 (C11), 55.4 (d, $^2J_{CP}$ = 24.3 Hz, C13), 55.3 (C15), 54.1 (C5), 47.1 (C9), 42.7 (C3), 42.5 (d, $^2J_{CP}$ = 6.5 Hz, C8), 30.9 (d, $^3J_{CP}$ = 14.2 Hz, C14), 29.3 (d, $^5J_{CP}$ = 8.1 Hz, C6), 27.5 (C16). ^{31}P NMR (162 MHz, C_6D_6): δ = 89.9.

1.8. Synthesis of chlorophosphonium salt (1-Cl)Cl

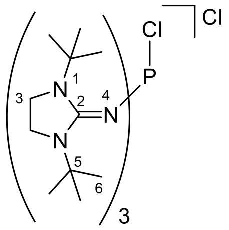

The compound was prepared to determine the molecular structure and to compare the NMR spectra with those of (**1**-Cl)Cl obtained from dechlorination experiments. Hexachloroethane (118 mg, 0.500 mmol) was added to a stirred solution of phosphine **1** (0.310 g, 0.500 mmol) in THF (3 mL). After 30 min the formed precipitate was collected by filtration, washed with diethyl ether (3 × 2mL) and dried *in vacuo*. Compound (**1**-Cl)Cl was obtained as a colorless, crystalline solid. Yield: 325 mg (0.470 mmol, 94%). ^{1}H NMR (400 MHz, CD_3CN): δ (ppm) = 3.37 (d, $^{5}J_{PH}$ = 1.6 Hz, 12H, H3), 1.39 (s, 36H, H6). ^{13}C{^{1}H} NMR (101 MHz, CD_3CN): δ (ppm) = 155.8 (d, $^{2}J_{PC}$ = 20.0 Hz, C2), 57.6 (C5), 44.2 (C3), 29.0 (C6). ^{31}P NMR (162 MHz, CD_3CN): δ (ppm) = –52.9. HRMS (ESI, positive): *m/z* (%) = 654.4861 (100, calcd for [**1**-Cl]$^{+}$ 654.4867).

1.9. Synthesis of chlorophosphonium salt (2-H)Cl

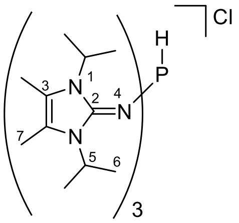

The compound was prepared for comparison of the spectroscopic parameters with those of (**2**-H)OTf. The synthetic route is based on a literature procedure using $P(NMe_2)_2Cl$ as phosphorus precursor.[S11] A sample of bis(di-*iso*-propylamino)chlorophosphine (0.503 g, 1.885 mmol) and 1,3-di-*iso*-propyl-4,5-dimethylimidazolin-2-imine (R^2H) (1.104 g, 5.655 mmol) were dissolved in THF (20 mL) and the stirred mixture was heated to reflux for 5 h. Subsequently, the volatiles were removed under reduced pressure and the residue was then dried at 110 °C overnight *in vacuo* to afford (**2**-H)Cl as an off-white solid in quantitative yield. ^{1}H NMR (400 MHz, CD_3CN): δ (ppm) = 7.80 (d, $^{1}J_{PH}$ = 532.1 Hz, 1H, PH), 4.81 (sept, $^{3}J_{HH}$ = 7.1 Hz, 6H, H5), 2.14 (s, 18, H7), 1.56 (d, $^{3}J_{HH}$ = 7.1 Hz, 36H, H6). ^{31}P NMR (162 MHz, CD_3CN) δ (ppm) = –26.47 (d, $^{1}J_{PH}$ = 532.1 Hz). The NMR spectroscopic data are in agreement with those previously published.[S12]

1.10. Synthesis of the chlorophosphonium salt (Cy_3P-Cl)OTf

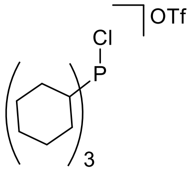

The compound was prepared for comparison of the spectroscopic parameters obtained for the same compound from dechlorination experiments. It also served as starting material for the synthesis of vinylenephosphine **4**. Hexachloroethane (237 mg, 1.000 mmol) was added to a stirred solution of tricyclohexylphosphine (280 mg, 1.000 mmol) in THF (3 mL). After 30 min a precipitate had formed which was isolated by filtration and dried *in vacuo*. The reaction product was dissolved in dichloromethane and AgOTf (270 mg, 1.050 mmol) was added. The reaction mixture was stirred for 15 min at ambient temperature in the dark. The resulting suspension was filtered.

The solvent of the filtrate was removed under reduced pressure and the crude product was recrystallized by slow addition of diethyl ether to a saturated dichloromethane solution at –40 °C. Compound (Cy_3P-Cl)OTf as isolated as a colorless, crystalline solid. Yield: 358 mg, 0.77 mmol, 77%) ^{1}H NMR (400 MHz, THF-d_8): δ = 3.28–3.16 (m, 3H), 2.22–2.10 (m, 6H), 1.93–1.82 (m, 6H), 1.77–1.51 (15H), 1.42–1.27 (3H). ^{13}C{^{1}H} NMR (101 MHz, THF-d_8,): δ = 122.2 (q, $^1J_{CF}$ = 322.9 Hz, CF_3), 36.0 (d, $^1J_{CP}$ = 30.1 Hz, CH), 27.0 (d, J_{CP} = 4.1 Hz, CH_2), 26.4 (d, J_{CP} = 14.3, CH_2), 25.8 (CH_2). ^{19}F NMR (THF-d_8, 376 MHz): δ (ppm) = –78.7. ^{31}P NMR (162 MHz, THF-d_8): δ = 105.9. The NMR spectroscopic data are in agreement with those published for (Cy_3P-Cl)Cl.[S13]

1.11. Synthesis of the cyclohex-1-en-1-yldicyclohexylphosphine 4

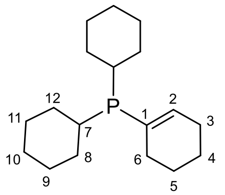

To a stirred solution of (Cy_3P-Cl)OTf (93 mg, 0.200 mmol) in THF (2 mL) was added a solution of NHC 1,3-di-*iso*-propyl-4,5-dimethylimidazolin-2-ylidene **3** (70 mg, 0.388 mmol) in THF (2 mL). The mixture was stirred for 120 min. Subsequently, the solvent was removed *in vacuo* and the residue was extracted with *n*-hexane (4 mL). Removal of the solvent and sublimation (60 °C at 10^{-3} mbar) gave **4** as a white solid. Yield: 30 mg, (0.108 mmol, 54%). *Note: The CH_2 groups of the cyclohexyl substituents in **4** are diastereotopic, which has been previously reported for PCy_2Ph.[S14] The NMR signals were assigned using 2D NMR experiments.* ^{1}H NMR (400 MHz, C_6D_6): δ = 6.44 (dt, $^3J_{PH}$ = 17.2 Hz, , $^3J_{HH}$ = 3.8 Hz, 1H, H2), 2.07–2.01 (m, 2H, H6), 2.00–1.94 (m, 2H, H3), 1.93–1.84 (m, 2H, H12a), 1.80–1.59 (m, 10H, H7, H8a, H9a, H10a, H11a), 1.57–1.42 (m, 4H, H4, H5), 1.41–1.13 (m, 10H, H8b, H9b, H10b, H11b, H12b). ^{13}C{^{1}H} NMR (101 MHz, C_6D_6): δ = 141.5 (d, $^2J_{PC}$ = 43.4 Hz, C2), 134.7 (d, $^1J_{PC}$ = 19.4 Hz, C1), 33.2 (d, $^1J_{PC}$ = 13.8 Hz, C7), 31.5 (d, $^2J_{PC}$ = 17.6 Hz, C12), 30.7 (d, $^2J_{PC}$ = 10.0 Hz, C8), 27.8–27.5 (m, C6, C9, C11), 27.3 (d, $^3J_{PC}$ = 15.1 Hz, C3), 27.1 (d, $^4J_{PC}$ = 1.0 Hz, C10), 23.8 (d, $^3J_{PC}$ = 1.0 Hz, C5), 22.8 (C4). ^{31}P NMR (162 MHz, C_6D_6): δ = 10.3. HRMS (ESI, positive): *m/z* (%) = 279.2225 (100, calcd for [**4**+H]$^+$ 279.2236).

1.12. Synthesis of 2-chloro-1,3-di-*iso*-propyl-4,5-dimethylimidazolium triflate (3-Cl)OTf

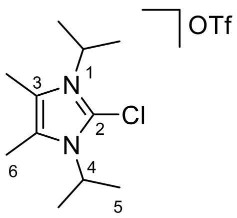

A sample of 2-chloro-1,3-diisopropyl-4,5-dimethylimidazolium chloride (0.251 g, 1.00 mol) was dissolved in dichloromethane (3 ml) and AgOTf (0.270 g, 1.050 mmol) was added. The reaction mixture was stirred for 15 min at ambient temperature in the dark. The resulting suspension was filtered. The solvent of the filtrate was removed under reduced pressure and the crude product was recrystallized by slow diffusion of diethyl ether into a saturated dichloromethane solution of the compound at –40 °C. Compound (**3**-Cl)OTf was isolated as a colorless, crystalline solid. Yield: 0.281 g (0.770 mmol, 77%). ^{1}H NMR (400 MHz,

CD_3CN): δ (ppm) = 4.81 (sept, $^3J_{HH}$ = 7.0 Hz, 2H, H4), 2.29 (s, 6H, H6), 1.56 (d, $^3J_{HH}$ = 7.0 Hz, 12H, H5). $^{13}C\{^1H\}$ NMR (101 MHz, CD_3CN): δ (ppm) = 128.9 (C3), 128.2 (C2), 122.2 (q, $^1J_{CF}$ = 321 Hz, CF_3), 53.4 (C4), 20.5 (C5), 10.1 (C6). ^{19}F NMR (376 MHz, CD_3CN): δ (ppm) = –79.3.

1.13. Synthesis of 2-chloro-1,3-di-*iso*-propylbenzimidazolium triflate (5-Cl)OTf

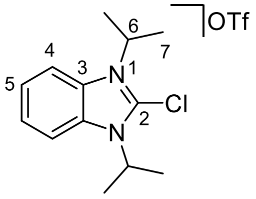

To a mixture of 1,3-di-*iso*-propylbenzimidazolium bromide (1.50 g, 5.30 mmol) and NaH (0.381 g, 15.9 mmol) was added THF (30 mL) at –78 °C. The suspension was stirred for 12 h at ambient temperature and was then filtered into a flask containing a solution of hexachloroethane (1.68 g, 7.10 mmol) in THF (30 mL) at –78 °C. The cold bath was then removed and the mixture was stirred for 4 h at ambient temperature. The precipitate formed was isolated by filtration, washed with Et_2O (3 × 10 mL) and added to an aqueous solution (100 mL) of NaOTf (4.13 g, 24.0 mmol). After stirring the mixture for 30 min., the formed precipitate was isolated by filtration, and washed with water (3 × 10 mL) and Et_2O (2 × 10 mL). The solid was dried *in vacuo* at 105 °C for 18 h to afford compound (**5**-Cl)OTf as a white solid. Yield: 1.45 g (3.75 mmol, 70%). ^1H NMR (400 MHz, CD_3CN): δ = 8.05–8.01 (m, 2H, Ar-H), 7.69–7.64 (m, 2H, Ar-H), 5.19 (sept, 2H, $^3J_{HH}$ = 7.0 Hz, H6), 1.73 (d, 12H, $^3J_{HH}$ = 7.0 Hz, H7). ^{13}C NMR (101 MHz, CD_3CN): δ = 139.3 (C2), 131.3 (C3), 127.7 (C4), 122.2 (q, $^1J_{CF}$ = 321 Hz, CF_3), 115.5 (C5), 54.4 (C6), 20.4 (C7). ^{19}F NMR (376 MHz, CD_3CN): δ (ppm) = –79.3.

1.14. Synthesis of 1,3-di-*iso*-propylbenzimidazolin-2-ylidene 5

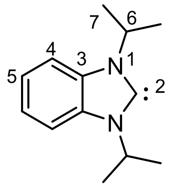

THF (20 mL) was added to 1,3-di-*iso*-propylbenzimidazolium bromide (500 mg, 1.77 mmol) and NaH (127 mg, 5.30 mmol) at –78 °C. The reaction mixture was stirred for 12 h at ambient temperature and was then filtered. The volatiles of the filtrate were removed *in vacuo*. After sublimation (40 °C, 10^{-3} mbar), NHC **5** was isolated as a light-yellow solid. Yield: 291 mg (1.44 mmol, 81%). ^1H NMR (400 MHz, C_6D_6): δ (ppm) = 7.07 (m, 4H, Ar-H), 4.43 (sept, 2H, $^3J_{HH}$ = 6.7 Hz, H6), (d, 12H, $^3J_{HH}$ = 6.7 Hz, H7). ^{13}C NMR (101 MHz, C_6D_6): δ (ppm) = 222.5 (C2), 135.2 (C3), 121.1, 110.3 (Ar-C), 49.6 (C6), 23.4 (C7).

1.15. Synthesis of 1,3-di-*iso*-propylbenzimidazolium triflate (5-H)OTf

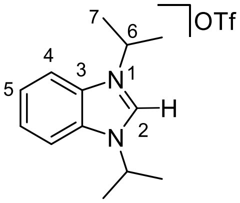

Compound (**5**-H)OTf was synthesized by a modified literature procedure.[S15] Samples of 1,3-di-*iso*-propylbenzimidazolium bromide (500 mg, 1.77 mmol) and AgOTf (473 mg, 1.85 mmol) were dissolved in dichloromethane (5 mL) and the mixture was stirred for 2 h. The suspension was filtered, the filtrate was concentrated and the solution was stored at –40 °C. The formed crystals were separated from the mother liquor and dried *in vacuo*. Compound (**5**-H)OTf was isolated as a colorless, crystalline solid. Yield: 520 mg (1.48 mmol, 84%). ^{1}H NMR (400 MHz, CD_3CN): δ(ppm) = 9.01 (s, 1H, H2), 7.95–7.90 (m, 2H, Ar-H), 7.71–7.67 (m, 2H, Ar-H), 4.97 (sept, 2H, $^3J_{HH}$ = 6.7 Hz, H6), 1.68 (d, 12H, $^3J_{HH}$ = 6.7 Hz, H7). ^{19}F NMR (376 MHz, CD_3CN): δ(ppm) = –79.3 (CF_3). The NMR spectroscopic data of (**5**-H)OTf are in agreement with the published data.[S15]

1.16. Synthesis of 2-chloro-1,3-di-*tert*-butylimidazolium triflate (7-Cl)OTf

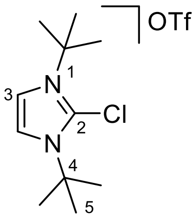

The reaction was carried out with 0.251 g (1.0 mmol) of 2-chloro-1,3-di-*tert*-butyl-imidazolidinium chloride as described for the synthesis of 2-chloro-1,3-di-*iso*-propyl-4,5-dimethylimidazolium triflate (**3**-Cl)OTf from 2-chloro-1,3-di-*iso*-propyl-4,5-dimethylimidazolium chloride (**3**-Cl)Cl (Section 1.12). Compound (**7**-Cl)OTf was isolated as a colorless, crystalline solid. Yield: 0.254 g (0.696 mmol, 70%). ^{1}H NMR (300 MHz, $CDCl_3$): δ(ppm) = 7.69 (s, 2H, H3), 1.78 (s, 18H, H5). ^{13}C{^{1}H} NMR (75 MHz, $CDCl_3$): δ (ppm) = 127.8 (C2), 121.1 (C3), 121.0 (q, $^1J_{CF}$ = 321 Hz, CF_3), 64.7 (C4), 28.9 (C5). ^{19}F NMR (376 MHz, $CDCl_3$): δ(ppm) = –78.2.

1.17. Synthesis of 2-chloro-1,3-di-*tert*-butylimidazolidinium tetrafluoroborate (8-Cl)BF_4

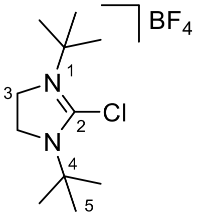

A sample of 2-chloro-1,3-di-*tert*-butyl-imidazolidinium chloride (**8**-Cl)Cl (0.506 g, 2.00 mmol) was dissolved in $CHCl_3$ (20 mL) and the solution was transferred to a separation funnel. An aqueous solution (20 mL) of $NaBF_4$ (1.100 g, 10.00 mmol) was added and the two-phase mixture was shaken vigorously. The organic phase was separated. For a better yield, the remaining aqueous phase was extracted once with $CHCl_3$ (5 mL). The combined organic fractions were dried over $MgSO_4$, filtered, and the solvent was removed *in vacuo*. Recrystallization of the solid at –40 °C from CH_2Cl_2/Et_2O yielded (**8**-Cl)BF_4 a colorless, crystalline solid. Yield: 0.438 g (1.44 mmol, 72%). ^{1}H NMR (400 MHz, $CDCl_3$): δ = 4.13 (s, 4H, H3), 1.56 (s, 18H, H5), ^{19}F NMR (471 MHz, $CDCl_3$): δ(ppm) = –153.3. ^{11}B{^{1}H} NMR (160 MHz, $CDCl_3$): δ(ppm) = – 0.98.

1.18. Reaction of the chloroimidazolium salt (3-Cl)OTf with IAPs 1 and 2

In a Schlenk flask, phosphine **1** or **2** (61.9 mg 0.100 mmol of **1** or 61.3 mg, 0.100 mmol of **2**) was dissolved in THF (2 mL) and (**3**-Cl)OTf (36.4 mg, 0.100 mmol) was added. The mixture was stirred at ambient temperature. After 5 min, the solution was analyzed by ^{31}P, ^{1}H, ^{19}F and ^{13}C NMR spectroscopy indication the complete conversion (Scheme S1). After removing of the solvent *in vacuo*, *n*-hexane (4 mL) was added and the suspension was stirred vigorously for 4 h. Carbene **3** was isolated after filtration and removal of the solvent. The solid residue containing the chlorophosphonium salt was dissolved in a minimum amount of THF. Addition of diethyl ether and storing the solution at –40 °C gave compounds (**1**-Cl)OTf and (**2**-Cl)OTf as crystalline solids. The isolated yields are shown in Table 1.

*Note: The extraction of **3** was less efficient in case of phosphine **2** due to the formation of an oily residue after removal of the solvent. The attempted separation of **3** by sublimation was unsuccessful, presumably due to the reaction of **3** with (**2**-Cl)OTf at elevated temperature.*

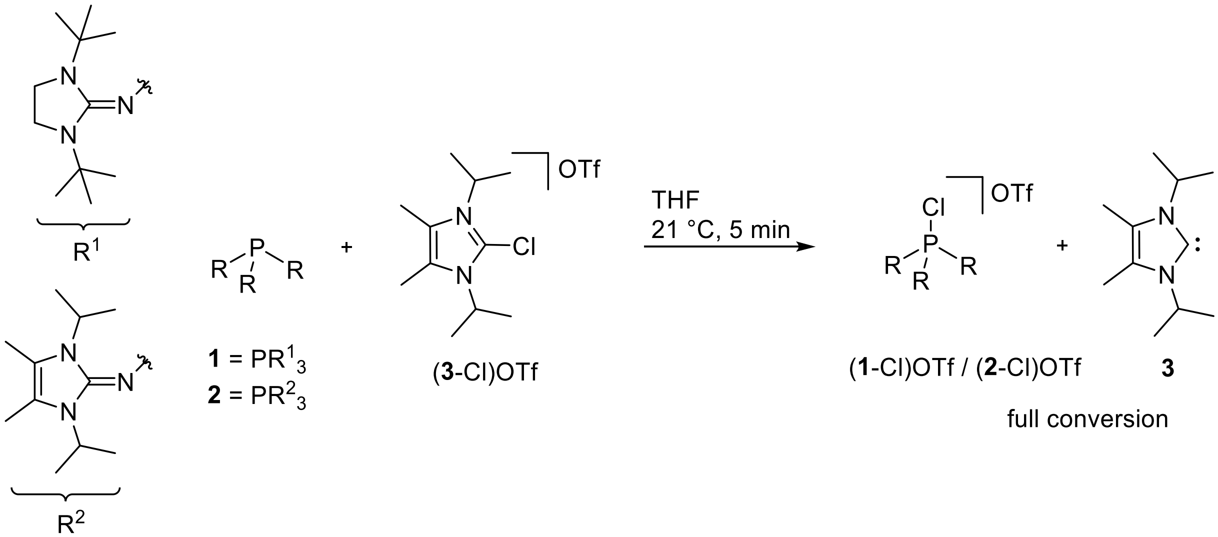

Scheme S1. Chloronium abstraction from (**3**-Cl)OTf with IAPs **1** and **2**.

Table S1. Isolated yields of phosphonium salts (**1**-Cl)OTf and (**2**-Cl)OTf and of carbene **3**.

Phosphine	isolated yield of (R_3P-Cl)OTf	isolated yield of NHC **3**
PR^1_3 **1**	84%	94%
PR^2_3 **2**	64%	72%

1.18.1. Analytical data of (1-Cl)OTf

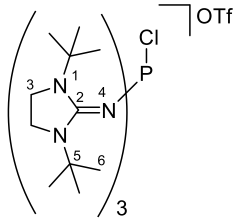

Yield: 68 mg (0.084 mmol, 84%). ^{1}H NMR (400 MHz, CD_2Cl_2): δ = 3.36 (s, 12H, (H3), 1.40 (s, 54H, H6). $^{13}C\{^{1}H\}$ NMR (101 MHz, CD_2Cl_2): δ = 154.8 (d, $^{2}J_{CP}$ = 20.9 Hz, C2), 121.4 (q, $^{1}J_{CF}$ = 321.5 Hz, CF_3), 57.2 (C5), 43.7 (C3), 28.9 (C6). ^{19}F NMR (CD_2Cl_2, 376 MHz): δ = –78.9. ^{31}P NMR (162 MHz, CD_2Cl_2): δ = –53.9. HRMS (ESI, positive): *m/z* (%) = 654.4839 (100, calcd for [**1**-Cl]$^+$ 654.4862). The NMR data of (**1**-Cl)OTf (Figures S66–S69) are consistent with those of (**1**-Cl)Cl obtained by direct chlorination of **1** (see section 1.8, Figures S31–S33).

1.18.2. Analytical data for (2-Cl)OTf

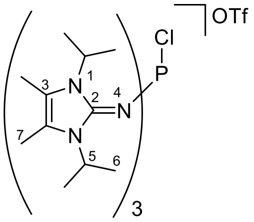

Yield: 51 mg (0.064 mmol, 64%). ^{1}H NMR (400 MHz, CD_2Cl_2): δ = 5.05 (sept, $^{3}J_{HH}$ = 7.1 Hz, 6H, H5), 2.20 (s, 18H, H7), 1.39 (d, $^{3}J_{HH}$ = 7.1 Hz, 36H, H6). $^{13}C\{^{1}H\}$ NMR (101 MHz, CD_2Cl_2): δ = 144.8 (C2), 119.6 (C3), 48.0 (C5), 21.6 (C6), 10.2 (C7). ^{19}F NMR (CD_2Cl_2, 376 MHz): δ = –78.9. ^{31}P NMR (162 MHz, CD_2Cl_2): δ = –21.2. HRMS (ESI, positive): *m/z* (%) = 648.4370 (100, calcd for [**2**-Cl]$^+$ 648.43923 (Figures S76–S79). The NMR spectra of the isolated NHC **3** are shown in Figures S80–S81.

1.19. Reaction of triphenylphosphine with (3-Cl)OTf

Triphenylphosphine (26.2 mg 0.100 mmol) was dissolved in THF (2 mL) and (**3**-Cl)OTf (36.4 mg, 0.100 mmol) was added. The mixture was stirred at ambient temperature for 72 h and analyzed by NMR spectroscopy (Scheme S2), showing only the resonances for the starting material.

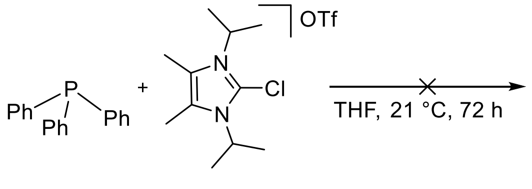

(**3**-Cl)OTf

Scheme S2. Reaction of PPh_3 with (**3**-Cl)OTf leading to no transformation.

1.20. Reaction of tri-*tert*-butylphosphine with (3-Cl)OTf

Tri-*tert*-butylphosphine (20.2 mg 0.100 mmol) was dissolved in THF (2 mL) and (**3**-Cl)OTf (36.4 mg, 0.100 mmol) was added. The mixture was stirred at ambient temperature for 3 h. Quantitative ^{31}P NMR spectroscopy revealed an equilibrium state between tri-*tert*-butyl phosphine (δ(^{31}P) = 62.9 ppm) and the

chlorophosphonium salt (tBu_3P-Cl)OTf ($\delta(^{31}P)$ = 123.6 ppm) in the ratio of 73:27 (Scheme S3, Figure S82). After storing the reaction mixture at ambient temperature for 3 d, the characteristic resonance of the imidazolium proton of (**3**-H)OTf was detected in the ^{1}H NMR spectrum (Figure S83) and the ^{31}P NMR spectrum shows a new resonances of tBu_2PCl ($\delta(^{31}P)$ = 147.3 ppm, Figure S84). The yields for (tBu_3P-Cl)OTf and tBu_2P-Cl were determined using quantitative ^{31}P NMR spectroscopy (Figure S84). The proposed mechanism for the formation of tBu_2PCl is depicted in Scheme S4.

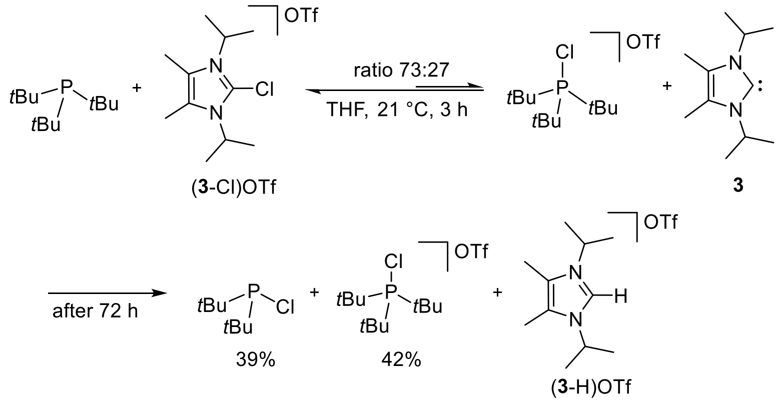

Scheme S3. Reaction of PtBu$_3$ with (**3**-Cl)OTf.

Proposed reaction mechanism for the formation of tBu_2PCl

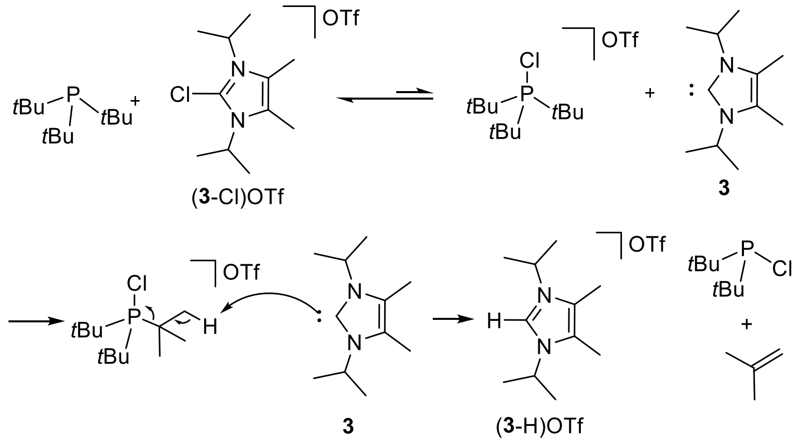

Scheme S4. Proposed mechanism for the formation of tBu_2PCl.

1.21. Reaction of the chloroimidazolium salts (3-Cl)BF_4 or (3-Cl)OTf with tricyclohexylphosphine PCy_3

Tricyclohexylphosphine (28.0 mg, 100 mmol) was dissolved in THF (2 mL) and (**3**-Cl)BF_4 (30.2 mg, 0.100 mmol) was added. The reaction mixture was stirred at ambient temperature for 3 h (Scheme S5, top). The ^{31}P and ^{19}F NMR spectrum of the reaction mixture showed several new resonances indicating decomposition of the BF_4 anion and the formation of multiple species (Figures S85–S86).

By contrast, the reaction of PCy_3 with the triflate salt (**3**-Cl)OTf gave a mixture of chlorophosphonium salt (Cy_3P-Cl)OTf/Cl, the cyclohex-1-en-dicyclohexylphosphine **4** and the dechlorinated imidazolium salt (**3**-H)OTf/Cl after 72 h (Scheme S5, bottom). Phosphine **4** was identified via ^{1}H NMR spectroscopy (Figure S87, see Section 1.11). The triflate anion did not participate in the reaction as evidenced by ^{19}F NMR spectroscopy (Figure S88). NMR yields for the phosphorus compounds (Cy_3P-Cl)OTf/Cl and **4** were determined by quantitative ^{31}P NMR spectroscopy (Figure S89).

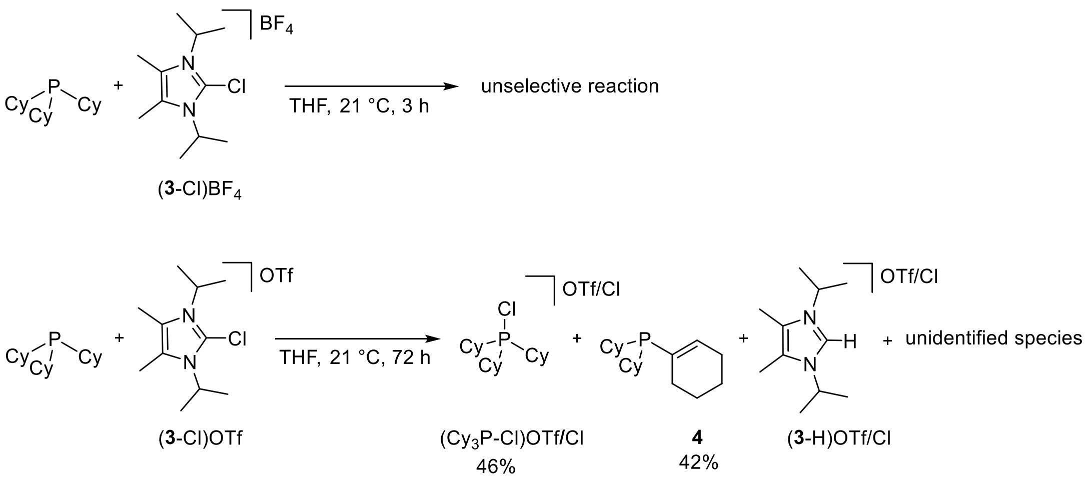

Scheme S5. Reaction of $PCy_3$with (**3**-Cl)BF_4 (top) and of PCy_3 with (**3**-Cl)OTf (bottom). Yields of phosphorous containing species were determined by quantitative ^{31}P NMR spectroscopy.

Proposed reaction mechanism for the formation of 4

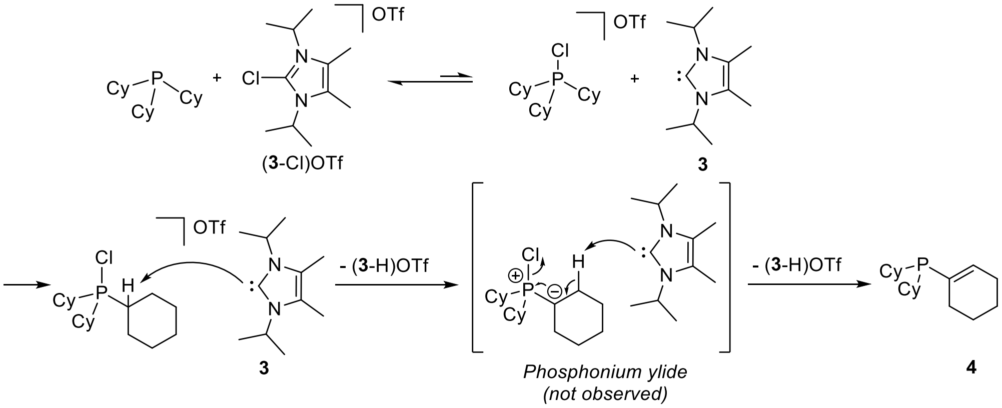

Scheme S6. Proposed mechanism for the formation of **4** via stepwise deprotonation of (Cy_3P-Cl)OTf by two molecules of carbene **3**.

Additional experiment to support the proposed mechanism for the formation of 4

As depicted in Scheme S6, the formation of vinylenephosphine **4** requires two equivalents of NHC **3**. However, the stoichiometric reaction of PCy_3 with (**3**-Cl)OTf produces one equivalent of **3** giving **4** in a maximum yield of 50%. According to this hypothesis, the addition of one equivalent of NHC **3** to the reaction mixture should lead to complete conversion of chlorophosphonium salt (Cy_3P-Cl)OTf to vinylenephosphine **4**. This mechanistic proposal was confirmed by the following experiment: A THF solution of PCy_3 (28.0 mg, 0.100 mmol) was added to (**3**-Cl)OTf (36.4 mg, 0.100 mmol) in an NMR tube and the tube was shaken vigorously for 30 min. Then, one equivalent of carbene **3** (18.0 mg, 0.100 mmol) was added to the mixture. The reaction progress was monitored using ^{31}P NMR spectroscopy (Figure S90). The ^{31}P NMR spectrum indicated full conversion of (Cy_3P-Cl)OTf to vinylenephosphine **4** (Scheme 7).

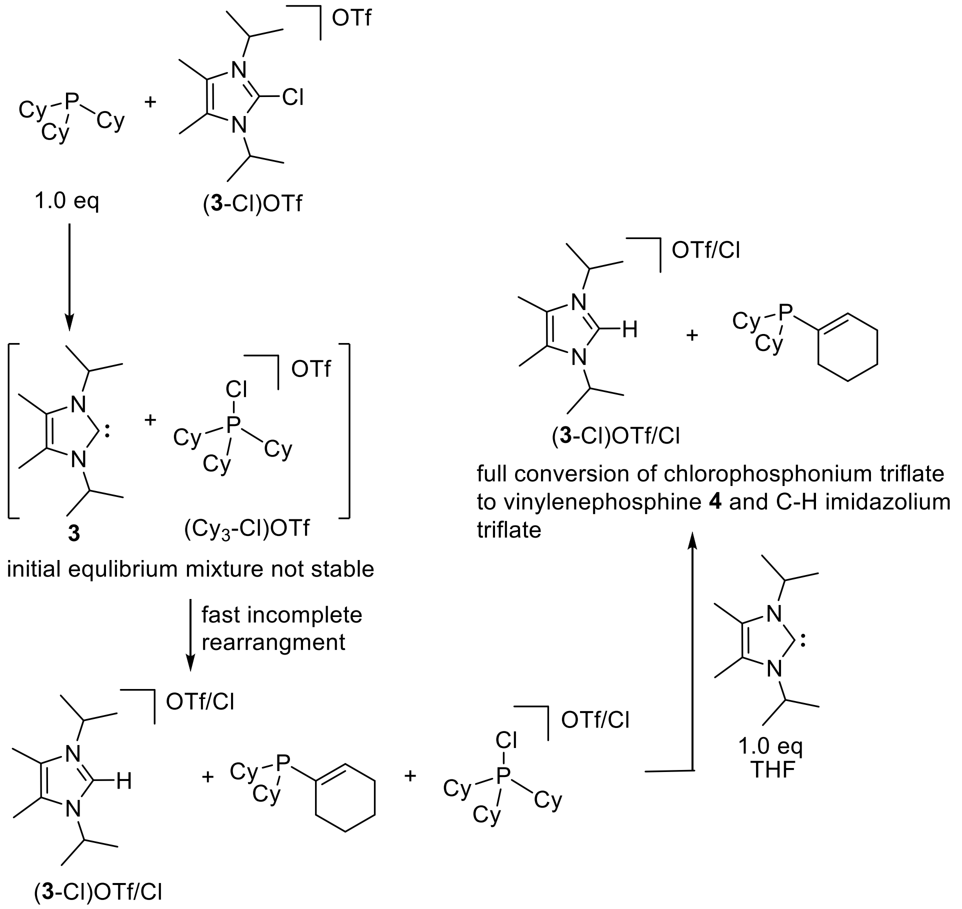

Scheme S7. Full conversion of PCy_3 to **4** by adding one equivalent of carbene **3** to the reaction mixture of PCy_3 and (**3**-Cl)OTf (yields were determined by ^{31}P NMR spectroscopy, Figure S90)

1.22. Chloronium ion abstraction from 2-chlorobenzimidazolium *vs* 2-chlorobenzimidazole using phosphines 1 and 2

Phosphine **1** (50.2 mg 0.081 mmol) or **2** (49.7 mg, 0.081 mmol) was dissolved in THF (2 mL) and an equimolar mixture of the salt 2-chloro-1,3-di-*iso*-propylbenzimidazolium triflate (**5**-Cl)OTf (31.2 mg, 0.081 mmol) and the neutral compound 2-chloro-1-*iso*-propylbenzimidazole **6** (15.8 mg, 0.081 mmol, 1.0 eq.) was added. The mixture was stirred for 16 h at ambient temperature (Scheme S8). Analysis of the reaction mixture using ^{31}P, ^{1}H and ^{13}C NMR spectroscopy confirmed that with both phosphines chloronium abstraction proceeded exclusively from the 2-chlorobenzimidazolium cation in (**5**-Cl)OTf while no reaction was observed for the neutral chlorobenzimidazole **6** (Figures S91–S98). Note that all relevant species have been characterized by NMR spectroscopy in previous experiments.

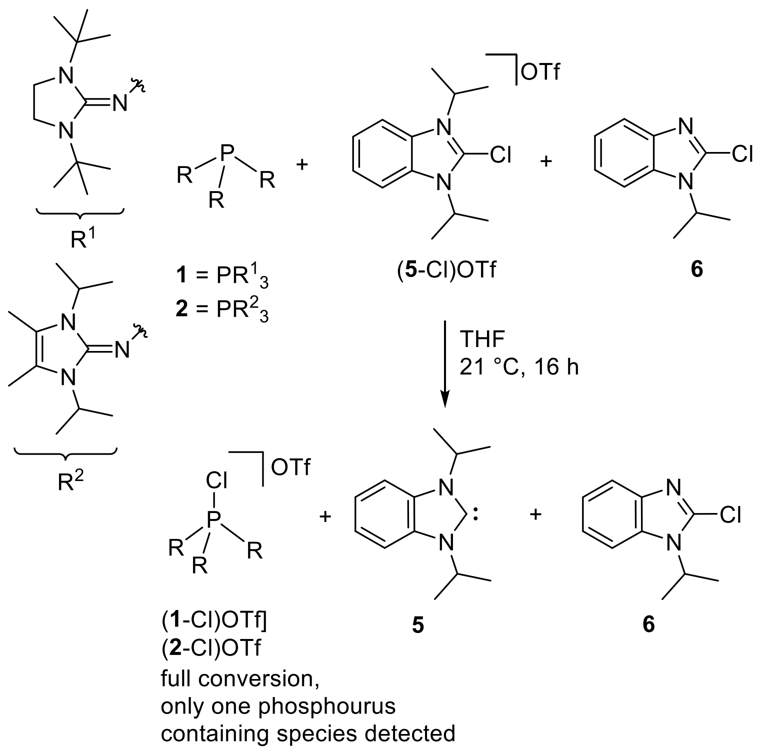

Scheme S8. Selective chloronium ion abstraction from (**5**-Cl)OTf in the presence of **6** with IAPs **1** and **2**.

1.23. Chloronium ion abstraction *vs* C2-deprotonation from 2-chlorinated and 2-protonated benzimidazolium salts with phosphines 1 and 2

Phosphine **1** (50.2 mg 0.081 mmol) or **2** (49.7 mg, 0.081 mmol) was dissolved in THF (2 mL) and an equimolar mixture of 2-chloro-1,3-di-*iso*-propylbenzimidazolium triflate (**5**-Cl)OTf (31.2 mg, 0.081 mmol) and 1,3-di-*iso*-propylbenzimidazolium triflate (**5**-H)OTf (28.5 mg, 0.081 mmol) was added (Schemes S9 and S10). The mixture was stirred for 16 h at ambient temperature. The reaction progress was monitored in both cases using ^{31}P, ^{1}H and ^{13}C NMR spectroscopy (Figures S99–S105). The yields of the phosphoniumchlorides (**1**-Cl)OTf and (**2**-Cl)OTf were determined by quantitative ^{31}P NMR spectroscopy (Figures S102 and S105). IAP **1** reacted exclusively under chloronium abstraction from (**5**-Cl)OTf to give the NHC **5** associated with unreacted (**5**-H)OTf (Scheme S9, Figures S99–S100). In contrast, IAP **2** gave a mixture of NHC **5**, the 2-chlorobenzimidazolium salt (**5**-Cl)OTf and the benzimidazolium salt (**5**-H)OTf (Scheme S10, Figures S103–S104).

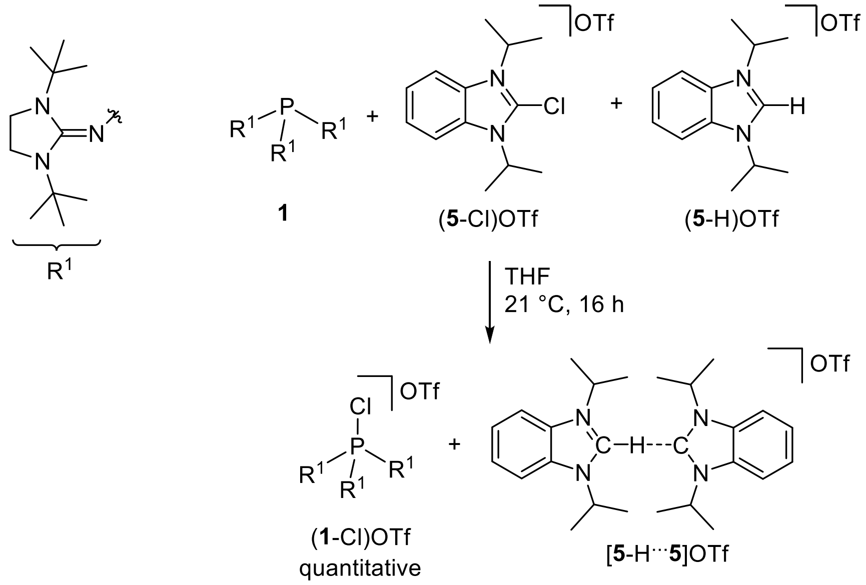

Scheme S9. Selective abstraction of chloronium ions with IAP **1** from the mixture (**5**-Cl)OTf/(**5**-H)OTf.

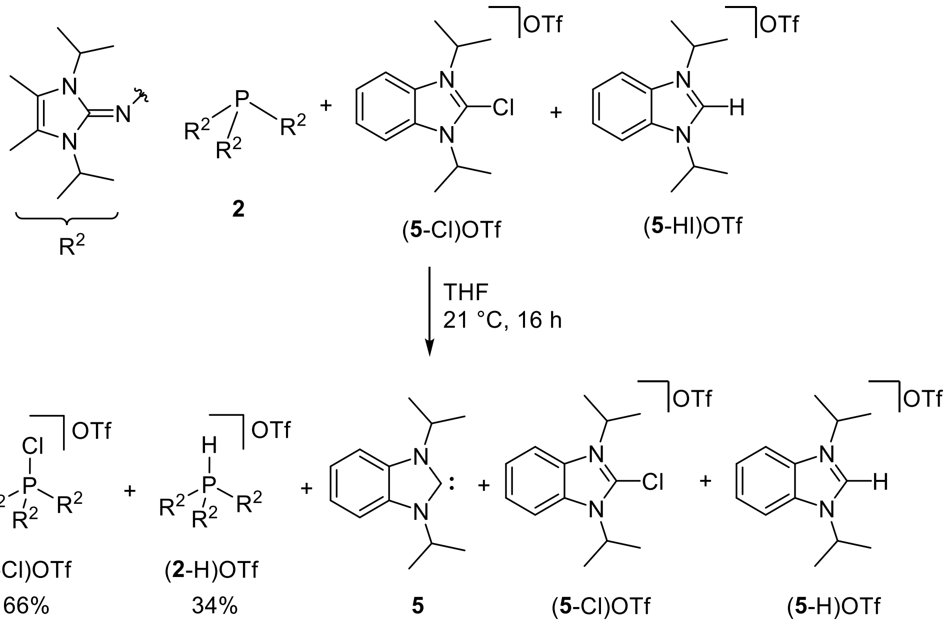

Scheme S10. Unselective reaction of IAP **2** with the mixture (**5**-Cl)OTf/(**5**-H)OTf to give the phosphonium salts (**2**-Cl)OTf and (**2**-H)OTf (yields of phosphonium salts were determined by ^{31}P NMR spectroscopy, Figure S105).

1.24. Chloronium ion abstraction from 2-chlorazolium salts (7-Cl)OTf and (8-Cl)BF_4 with IAP 1

Differently to the azolium salts (**3**-Cl)OTf and (**5**-Cl)OTf (Schemes S1 and S9), the imidazolium and imidazolidinum salts (**7**-Cl)OTf and (**8**-Cl)BF_4 feature C4/C5 unsubstituted diaminoheterocycles. Dechlorination of (**7**-Cl)OTf and (**8**-Cl)BF_4 with phosphine **1** proceeds rapidly giving the free NHCs **7** and **8** together with the phosphonium salts (**1**-Cl)OTf and (**1**-Cl)BF_4, respectively (Scheme S11). The reaction products were characterized by NMR spectroscopy Figures S106–S111). In both cases, complete conversion of the phosphine to (**1**-Cl)$^+$ was observed. The characteristic C_{NHC} resonance was detected for **7** in the $^{13}C\{^1H\}$ NMR spectrum of the reaction mixture of (**7**-Cl)OTf + **1** at δ = 212.9 ppm (Figure S107). In the case of the reaction of (**8**-Cl)BF_4 + **1** the C_{NHC} resonance for carbene **8** was not detected in the $^{13}C\{^1H\}$ NMR spectrum of the reaction mixture (Figure S109). The free NHC was therefore isolated by evaporation of the volatiles from the reaction mixture and subsequent extraction with *n*-hexane (Figures S110–S111). The $^{13}C\{^1H\}$ NMR spectrum shows the resonance for the C_{NHC} atom in the expected range at δ = 238.6 ppm (Figure S111).

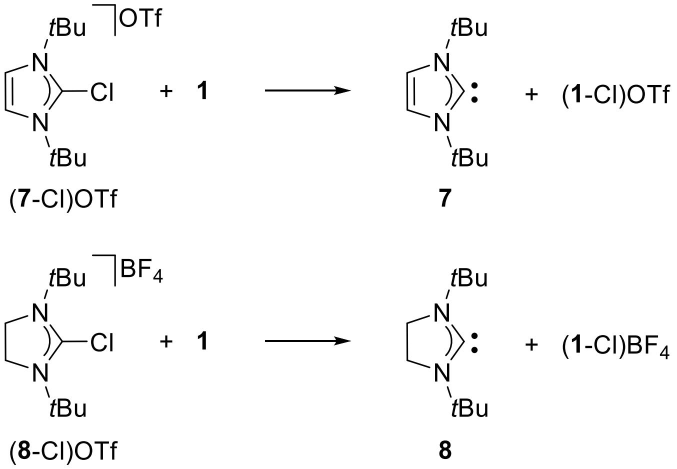

Scheme S11. Reaction of (**7**-Cl)OTf and (**8**-Cl)BF_4 with phosphine **1** to give the phosphonium salts 1-Cl)OTf and (**1**-Cl)BF_4 and the carbenes **7** and **8**.

1.25. Dechlorination of (9-Cl)PF_6 with IAP 1 in the presence of [$IrCl_2Cp^*$]$_2$

Finally, chloronium ion abstraction from the sterically non-demanding N,N'-substituted, saturated imidazolidinium salt (**9**-Cl)PF_6 was investigated (Scheme 12). Since it was expected that dechlorination of (**9**-Cl)PF_6 would yield the free carbene **9**, which is known to dimerize to the entetramine, the dechlorination was performed in the presence of [$IrCl_2Cp^*$]$_2$ to trap NHC **9**. This strategy was successful, leading to formation of complex [Ir(**9**)Cl_2Cp^*] [**10**]. The complex was characterized by ^1H, $^{13}C\{^1H\}$ NMR spectroscopy (Figures 112–113).

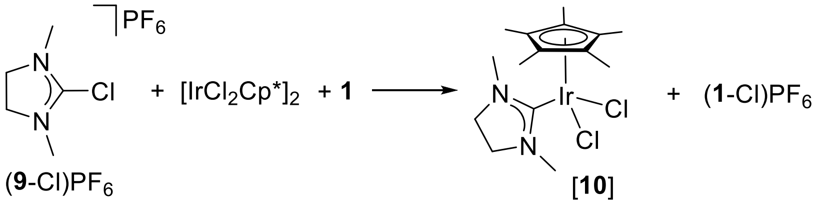

Scheme S12. Dechlorination of (**9**-Cl)PF_6 with **1** in the presence of $[IrCl_2Cp^*]_2$ to give [Ir(**9**)Cl_2Cp*] [**10**].

1.25.1. Synthesis of [Ir(9)Cl_2Cp*] [10]

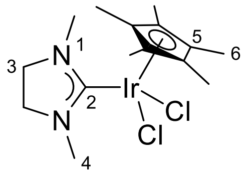

Phosphine **1** (62 mg, 0.10 mmol) and $[IrCl_2(Cp^*)]_2$ (40 mg, 0.050 mmol) were suspended in trifluorotoluene and the mixture was stirred for 30 minutes. Subsequently, (**9**-Cl)PF_6 (28 mg, 0.10 mmol) was added and the mixture was stirred at ambient temperature for 2 h. The solvent was then removed *in vacuo*. The crude reaction product was purified by column chromatography (SiO_2, CH_2Cl_2:MeOH, 98:2, R_f = 0.25) to give [Ir(**9**)Cl_2Cp*] as an orange solid. (*Note: If complex [Ir(***9***)Cl_2Cp*] is contaminated with small amounts of (***1***-Cl)PF_6, purification can be achieved by diffusion of Et_2O into a saturated $CHCl_3$ solution of the complex, leading to the crystallization of (***1***-Cl)PF_6).* Yield: 40 mg (0.81 mmol, 81%). ^{1}H NMR (300 MHz, $CDCl_3$): δ= 3.74–3.64 (m, 4H, H3), 3.30 (s, 6H, H4), 1.61 (s, 15H, H6). ^{13}C{^{1}H} NMR (75 MHz, $CDCl_3$): δ= 186.3 (C2), 88.9 (C5), 52.1 (C3), 38.8 (C4), 9.2 (C6). HRMS (ESI, positive ions) *m/z* (%) = 461.1314 (100, calcd for [**9**–Cl]$^+$ 461.1330).

1.26. Reactivity of phosphines 1 and 2 towards iridium(I) and iridium(III) complexes

Phosphine **1** (29.8 mg, 0.048 mmol) or **2** (29.5 mg, 0.048 mmol) was dissolved in THF and $[Ir(cod)Cl]_2$ (16.1 mg, 0.024 mmol) or $[IrCp^*Cl_2]_2$ (19.1 mg, 0.024 mmol) was added. The reaction progress was monitored using ^{31}P NMR spectroscopy. Phosphine **1** did not react with any of the iridium complexes (Scheme S13, Figures S114 and S115).

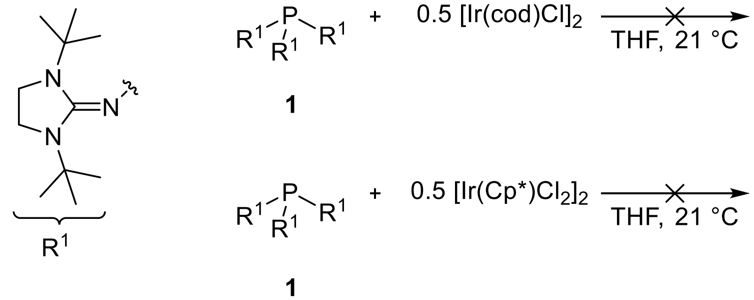

Scheme S13. No reaction of **1** with $[Ir(cod)Cl]_2$ or $[IrCp^*Cl_2]_2$.

Phosphine **2** reacted with the iridium complexes (Scheme S14). The ^{31}P NMR spectrum of the mixture of phosphine **2** and $[Ir(Cp^*)Cl_2]_2$ features several resonances (Figure S116). Two of these were assigned to **2** (at δ = 77.2 ppm) and $(\mathbf{2}\text{-H})^+$ (at δ = –26.2 ppm), the latter one indicating that the phosphine must have deprotonated a Cp* ligand of $[Ir(Cp^*)Cl_2]_2$.

In the ^{31}P NMR spectrum of the reaction mixture of phosphine **2** with $[Ir(cod)Cl]_2$, only one resonance at δ = 31.8 ppm was observed (Figure S119). This was assigned to iridium(I) complex [**11**] (see section 1.26.1 for full characterization of [**11**]).

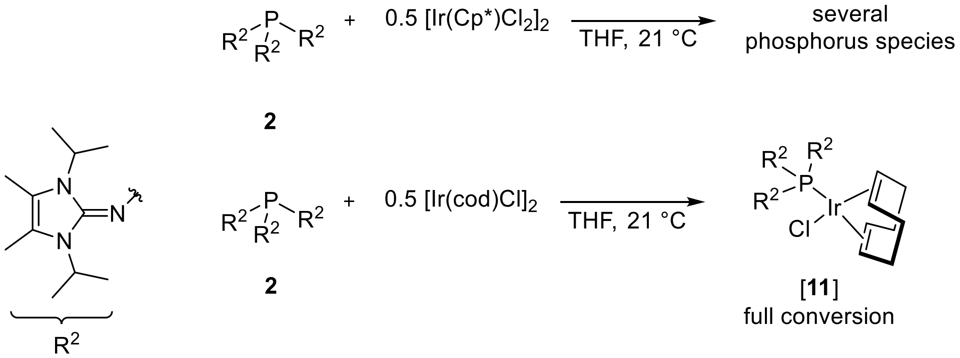

Scheme S14. Reaction of **2** with $[IrCp^*Cl_2]_2$ and $[Ir(cod)Cl]_2$. Full conversion to [**11**] was observed according to ^{31}P NMR spectroscopy.

1.26.1. Synthesis of complex [11]

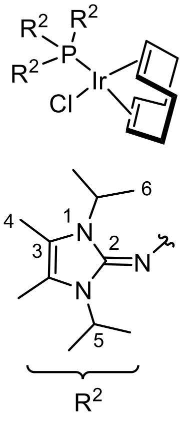

Phosphine **2** (29.5 mg, 0.048 mmol) was dissolved in THF (2 mL) and the iridium complex ($[IrCl(cod)]_2$ (16.1 mg, 0.024 mmol) was added. The mixture was stirred for 1 h. Complex [**11**] was crystallized by slow diffusion of diethyl ether into the reaction solution. The complex was obtained as a crystalline, orange solid. Yield: 15 mg (0.016 mmol, 33%). ^{1}H NMR (400 MHz, C_6D_6): δ = 5.75 (sept, $^3J_{HH}$ = 7.0 Hz, 6H, H5), 5.08–5.01 (m, 2H, cod-CH), 3.83–3.78 (m, 2H, cod-CH), 2.30–2.18 (m, 4H, cod-CH_2), 1.81 (s, 18H, H4), 1.75–1.70 (m, 4H, cod-CH_2), 1.44 (d, $^3J_{HH}$ = 7.0 Hz, 36H, H6). $^{13}C\{^1H\}$ NMR (101 MHz, C_6D_6): δ = 144.7 (d, $^2J_{CP}$ = 2.8 Hz, C2), 115.6 (C3), 85.1 (d, $^2J_{CP}$ = 20.7 Hz, cod-CH), 50.4 (d, $^2J_{CP}$ = 1.5 Hz, cod-CH), 46.2 (C5), 34.5 (d, $^2J_{CP}$ = 3.9 Hz, cod-CH_2), 29.9 (d, $^2J_{CP}$ = 2.9 Hz, cod-CH_2), 22.2 (C6), 10.4 (C4). ^{31}P NMR (101 MHz, C_6D_6): δ = 31.8.

1.27. Synthesis of complex [13]

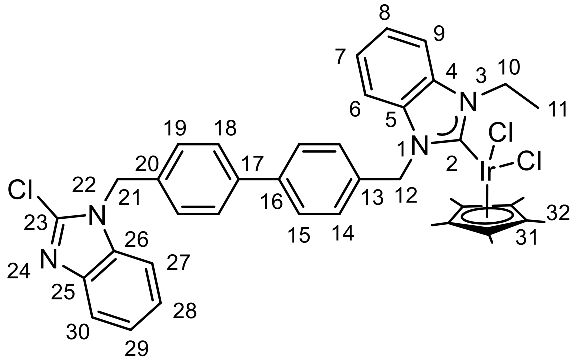

Equimolar amounts of ligand precursor (**12**-2Cl)BF_4 (60 mg, 0.10 mmol), phosphine **1** (62 mg, 0.10 mmol) and $[IrCl_2(Cp^*)]_2$ (39.8 mg, 0.050 mmol) were suspended in distilled THF (8 mL). The mixture was stirred at 25 °C for 4 h. After evaporation of the solvent the solid residue was purified by column chromatography on silica (eluent CH_2Cl_2:MeOH, 98:2) to give [**13**] as yellow solid. (*Note: complex [**13**] slowly decomposes on silica. The column chromatography has therefore to be carried out quickly.*) Yield: 67.1 mg (0.077 mmol, 77%). ^{1}H NMR (500 MHz, CD_2Cl_2): δ = 7.71–7.64 (m, 1H, H30), 7.57–7.53 (m, 2H, H18), 7.53–7.48 (m, 3H, H15, H9), 7.32–7.29 (m, 1H, H27), 7.28–7.21 (m, 7H, H8, H14, H19, H28, H29), 7.10–7.02 (m, 1H, H7), 7.00–6.95 (m, 1H, H6), 6.42 (d, $^2J_{HH}$ = 15.8 Hz, 1H, H12), 5.58 (d, $^2J_{HH}$ = 15.8 Hz, 1H, H12'), 5.43 (s, 2H, H21), 5.01 (dq, $^3J_{HH}$ = 7.0, $^2J_{HH}$ =14.3 Hz, 1H, H10), 4.46 (dq, $^3J_{HH}$ = 7.0, $^2J_{HH}$ =14.3 Hz, 1H, H10'), 1.66 (t, $^2J_{HH}$ = 7.0 Hz, 3H, H11), 1.57 (s, 15H, H32). ^{13}C{H} NMR (126 MHz, CD_2Cl_2): δ (ppm) = 171.1 (C2), 142.3 (C25), 141.1 (C23), 140.9 (C17), 139.7 (C16), 137.0 (C13), 135.7 (C5), 135.6 (C26), 135.4 (C4), 134.8 (C20), 127.9 (C19), 127.8 (C14), 127.8 (C18), 127.3 (C15), 123.6 (C28), 123.3 (C8), 123.2 (C7), 123.1 (C29), 119.7 (C30), 112.6 (C6), 111.5 (C9), 110.4 (C27), 90.1 (C31), 53.2 (C12), 48.0 (C21), 44.6 (C10), 16.6 (C11), 9.2 (C32). HRMS (ESI, positive ions) *m/z* (%) = 839.2235 (100, calcd for [**13**–Cl]$^+$ 839.2247).

1.28. Synthesis of complex [14]

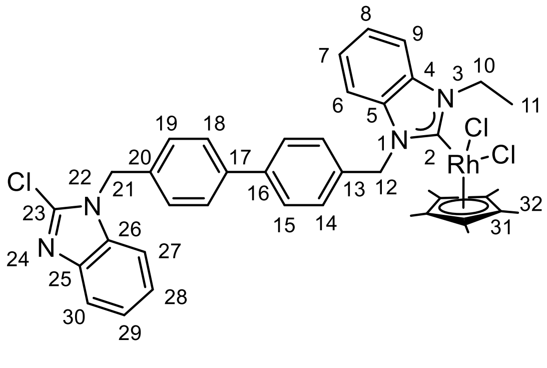

Equimolar amounts of ligand precursor (**12**-2Cl**)**(BF_4) (60 mg, 0.10 mmol), **1** (62 mg, 0.10 mmol) and $[RhCl_2(Cp^*)]_2$ (30.9 mg, 0.050 mmol) were suspended in distilled THF (8 mL). The mixture was stirred at 25 °C for 4 h. After evaporation of the solvent the solid residue was purified by column chromatography on silica (eluent CH_2Cl_2:MeOH, 98:2) to give [**14**] as an orange solid. Yield: 52.4 mg (0.067 mmol, 67%). ^{1}H NMR (400 MHz, CD_2Cl_2): δ = 7.71–7.63 (m, 1H, H30), 7.57–7.52 (m, 3H, H9, H18), 7.51–7.48 (m, 2H, H15), 7.32–7.22 (m, 8H, H8, H14, H19, H27, H28, H29), 7.15–7.05 (m, 1H, H7), 7.05–7.00 (m, 1H, H6), 6.56 (d, $^2J_{HH}$ = 15.7 Hz, 1H, H12), 5.63 (d, $^2J_{HH}$ = 15.7 Hz, 1H, H12'), 5.43 (s, 2H, H21), 5.14 (dq, $^3J_{HH}$ = 7.0, $^2J_{HH}$ =14.1

Hz, 1H, H10), 4.50 (dq, $^3J_{HH}$ = 7.0, $^2J_{HH}$ =14.1 Hz, 1H, H10'), 1.64 (t, $^3J_{HH}$ = 7.0 Hz, 3H, H11), 1.56 (s, 15H, H32). ^{13}C{H} NMR (101 MHz, CD_2Cl_2): δ(ppm) = 185.6 (d, $^1J_{RhC}$ = 56.4 Hz, C2), 142.4 (C25), 141.1 (C23), 140.9 (C17), 139.8 (C16), 136.9 (C13), 136.1 (d, $^3J_{RhC}$ = 1.0 Hz, C5), 135.8 (d, $^3J_{RhC}$ = 1.1 Hz, C4), 135.7 (C26), 134.8 (C20), 128.0 (C14), 127.9 (C19), 127.8 (C18), 127.3 (C15), 123.7 (C28), 123.4 (C8), 123.3 (C7), 123.1 (C29), 119.7 (C30), 112.6 (C6), 111.5 (C9), 110.4 (C27), 97.4 (d, $^1J_{RhC}$ = 6.9 Hz, C31), 53.5 (C12), 48.1 (C21), 45.0 (C10), 16.4 (C11), 9.6 (C32). HRMS (ESI, positive ions) *m/z* (%) = 749.1678 (100, calcd for [**14**–Cl]$^+$ 749.1685).

1.29. Synthesis of complex [15]

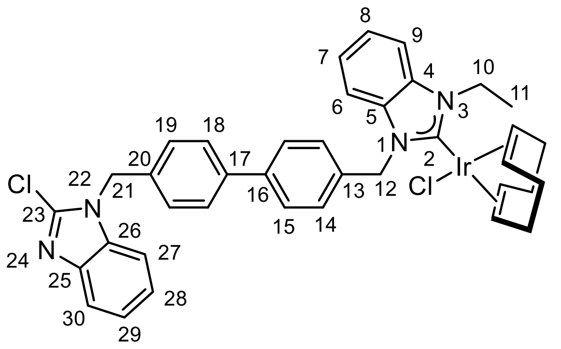

Equimolar amounts of ligand precursor (**12**-2Cl)(BF_4) (30 mg, 0.05 mmol), **1** (31 mg, 0.05 mmol) and $[IrCl(cod)]_2$ (16.8 mg, 0.025 mmol) were suspended in distilled THF (8 mL) and the mixture was stirred at 25 °C for 4 h. After evaporation of the solvent the solid residue was purified by column chromatography on basic alumina (eluent CH_2Cl_2:MeOH, 99:1) to give [**15**] as a yellow solid. Yield: 18.9 mg (0.023 mmol, 46%). ^{1}H NMR (400 MHz, CD_2Cl_2): δ = 7.71–7.64 (m, 1H, H30), 7.58–7.50 (m, 4H, H15, H18), 7.49–7.45 (m, 2H, H14), 7.41–7.33 (m, 1H, H9), 7.32–7.28 (m, 1H, H27), 7.28–7.24 (m, 4H, H19, H28, H29), 7.20 (ddd, $^4J_{HH}$ = 1.4 Hz, $^3J_{HH}$ = 7.0, 8.2 Hz, 1H, H8), 7.10–7.01 (m, 2H, H6, H7), 6.38 (d, $^2J_{HH}$ = 15.6 Hz, 1H, H12), 5.78 (d, $^2J_{HH}$ = 15.6 Hz, 1H, H12'), 5.43 (s, 2H, H21), 4.94 (dq, $^3J_{HH}$ = 7.3, $^2J_{HH}$ = 14.7 Hz, 1H, H10), 4.72 (dq, $^3J_{HH}$ = 7.3, $^2J_{HH}$ = 14.7 Hz, 1H, H10'), 4.7–4.6 (m, 2H, cod-CH), 3.07 (td, $^3J_{HH}$ = 2.7, $^3J_{HH}$ = 7.0 Hz, 1H, cod-CH), 2.94 (td, $^3J_{HH}$ = 2.7, $^3J_{HH}$ = 7.3 Hz, 1H, cod-CH), 2.39–2.06 (m, 4H, cod-CH_2), 1.88–1.67 (m, 3H, cod-CH_2), 1.62–1.58 (m, 4H, H11, cod-CH_2). ^{13}C{H} NMR (101 MHz, CD_2Cl_2): δ = 192.5 (C2), 142.4 (C25), 141.1 (C23), 140.8 (C17), 140.1 (C16), 135.9 (C13), 135.7 (C26), 135.2 (C5), 135.1 (C4), 134.9 (C20), 128.3 (C14), 127.8 (C18), 127.8 (C19), 127.6 (C15), 123.7 (C28), 123.1 (C29), 122.9 (C8), 122.7 (C7), 119.7 (C30), 111.5 (C6), 110.4 (C27), 110.3 (C9), 87.3 (cod-CH), 86.9 (cod-CH), 53.3 (cod-CH), 53.0 (cod-CH), 52.6 (C12), 48.0 (C21), 43.7 (C10), 34.1 (cod-CH_2), 33.6 (cod-CH_2), 30.0 (cod-CH_2), 29.5 (cod-CH_2), 15.1 (C11). HRMS (ESI, positive ions) *m/z* (%) = 777.2342 (100, calcd for [**15**–Cl]$^+$ 777.2329).

1.30. Synthesis of complex [16]

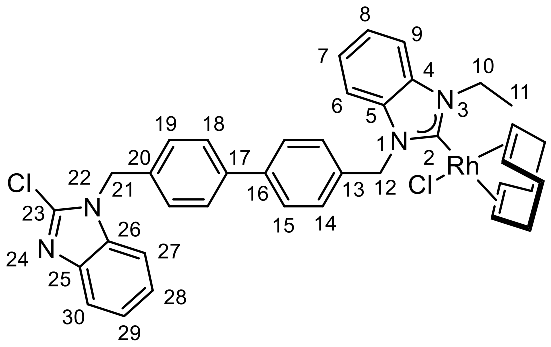

Equimolar amounts of ligand precursor (**12**-2Cl)(BF_4) (60 mg, 0.10 mmol), **1** (62 mg, 0.10 mmol) and [Rh(cod)Cl]$_2$ (24.7 mg, 0.050 mmol) were suspended in distilled THF (8 mL) and the mixture was stirred at 25 °C for 4 h. After evaporation of the solvent, the solid residue was purified by column chromatography on silica (eluent CH_2Cl_2:MeOH, 98:2) to give [**16**] as an orange solid. Yield: 55.7 mg (0.077 mmol, 77%). ^{1}H NMR (400 MHz, CD_2Cl_2): δ = 7.72–7.64 (m, 1H, H30), 7.63–7.42 (m, 6H, H14, H15, H18), 7.40–7.34 (m, 1H, H9), 7.34–7.23 (m, 5H, H19, H27, H28, H29), 7.23–7.15 (m, 1H, H8), 7.10–6.97 (m, 2H, H6, H7), 6.47 (d, $^2J_{HH}$ = 15.7 Hz, 1H, H12), 5.95 (d, $^2J_{HH}$ = 15.7 Hz, 1H, H12'), 5.43 (s, 2H, H21), 5.17–5.03 (m, 3H, H10, 2 × cod-CH), 4.88 (dq, $^3J_{HH}$ = 7.2, $^2J_{HH}$ = 14.3 Hz, 1H, H10'), 3.49–3.41 (m, 1H, cod-CH), 3.37–3.29 (m, 1H, cod-CH), 2.58–2.47 (m, 1H, cod-CH_2), 2.46–2.35 (m, 2H, cod-CH_2), 2.34–2.21 (m, 1H, cod-CH_2), 2.05–1.93 (m, 3H, cod-CH_2), 1.93–1.84 (m, 1H, cod-CH_2), 1.66 (t, $^3J_{HH}$ = 7.2 Hz, 3H, H11). ^{13}C{H} NMR (101 MHz, CD_2Cl_2): δ= 197.2 (d, $^1J_{RhC}$ = 51.0 Hz, C2), 142.4 (C25), 141.1 (C23), 140.8 (C17), 140.1 (C16), 136.1 (C13), 135.7 (C26), 135.1 (d, $^3J_{RhC}$ = 1.0 Hz, C5), 135.1 (d, $^3J_{RhC}$ = 1.01 Hz, C4), 134.9 (C20), 128.4 (C14), 127.9 (C19), 127.8 (C18), 127.6 (C15), 123.7 (C28), 123.1 (C29), 122.7 (C8), 122.6 (C7), 119.8 (C30), 111.3 (C6), 110.4 (C27), 110.3 (C9), 100.6 (d, $^1J_{RhC}$ = 6.6 Hz, cod-CH), 100.4 (d, $^1J_{RhC}$ = 6.7 Hz, cod-CH), 69.4 (d, $^1J_{RhC}$ = 12.0 Hz, cod-CH), 69.3 (d, $^1J_{RhC}$ = 12.1 Hz, cod-CH), 53.0 (C12), 48.0 (C21), 44.1 (C10), 33.5 (cod-CH_2), 33.0 (cod-CH_2), 29.3 (cod-CH_2), 29.0 (cod-*C*H_2), 15.1 (C11). HRMS (ESI, positive ions) *m/z* (%) = 687.1760 (100, calcd for [**16**-Cl]$^+$ 687.1762).

1.31. Synthesis of complex [17]

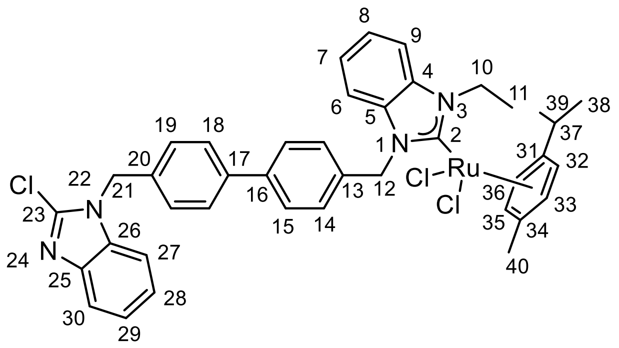

Equimolar amounts of ligand precursor (**12**-2Cl)(BF_4) (30 mg, 0.05 mmol), **1** (31 mg, 0.05 mmol) and [$RuCl_2$(*p*-cymene)]$_2$ (15.3 mg, 0.025 mmol) were suspended in distilled THF (8 mL) and the mixture was stirred at 25 °C for 3.5 h. After evaporation of the solvent the solid residue was purified by column chromatography on silica (eluent freshly distilled solvents CH_2Cl_2:MeOH, 98:2) to give [**17**] as a brown air sensitive solid. Yield: 17.8 mg (0.023 mmol, 46%). ^{1}H NMR (400 MHz, CD_2Cl_2):

δ = 7.70–7.65 (m, 1H, H30), 7.60–7.48 (m, 5H, H9, H15, H18), 7.34–7.21 (m, 6H, H8, H19, H27, H28, H29), 7.20–7.16 (m, 2H, H14), 7.15–7.12 (m, 1H, H7), 7.10–7.05 (m, 1H, H6), 6.3–6.0 (m br, 2H, H12), 5.48 (s br, 1H, H32), 5.43 (s, 2H, H21), 5.33 (s br, 1H, H36) 5.09 (s br, 2H, H10, H33), 4.94 (s br, 1H, H35), 4.57 (s br, 1H, H10'), 2.93 (h, $^3J_{HH}$ = 6.9 Hz, 1H, H37), 1.95 (s, 3H, H40), 1.63 (t, $^3J_{HH}$ = 7.1 Hz, 3H, H11), 1.31–1.22 (m, 6H, H38, H39). ^{13}C{H} NMR (126 MHz, CD_2Cl_2): δ = 190.8 (C2), 142.4 (C25), 141.1 (C23), 140.7 (C17), 139.8 (C16), 137.5 (C13), 136.2 (C5), 135.7 (C26), 135.5 (C4), 134.9 (C20), 127.8 (C18), 127.8 (C19), 127.6 (C15), 127.4 (C14), 123.6 (C28), 123.3 (C7), 123.2 (C8), 123.1 (C29), 119.8 (C30), 112.1 (C6), 111.3 (C9), 110.3 (C27), 110.2 (C31), 99.2 (C34), 88.0 (C32), 86.5 (C36), 83.3 (C35), 82.9 (C33), 53.1 (C12), 48.0 (C21), 45.5 (C10), 31.1 (C37), 23.3 (C38), 21.9 (C39), 18.8 (C40), 16.4 (C11). MS (ESI, positive ions) *m/z* (%) = 747.1603 (18, calcd for [**17**–Cl] 747.1600).

Note: Slow diffusion of *n*-pentane into a CH_2Cl_2 solution of [**17**] over one week at ambient temperature resulted in the formation of the crystalline complex [**17'**] where the ruthenium atom is η^6-coordinated by one of the ligand's benzyl groups with loss of the *p*-cymene ligand.

1.32. Synthesis of complex [18](BF_4)$_2$

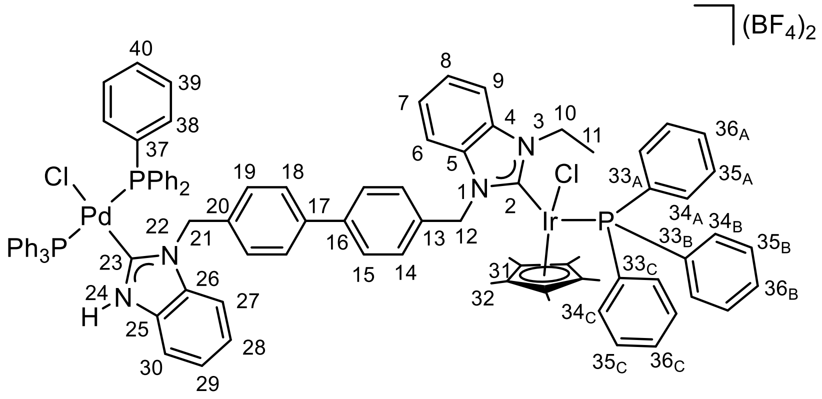

Samples of [**13**] (65.7 mg, 0.075 mmol), [Pd(PPh_3)$_4$] (174 mg, 0.151 mmol) and pyridinium tetrafluoroborate (25 mg, 0.15 mmol) were suspended in freshly distilled THF (10 mL). The mixture was stirred for 3 d at 90 °C in a sealed flask. After cooling to 25 °C diethyl ether (20 mL) was added. The resulting precipitate was isolated and washed twice with diethyl ether (15 mL each). After drying *in vacuo* complex [**18**](BF_4)$_2$ was obtained as an orange solid. Yield: 79.4 mg (0.04 mmol, 53%). Due to the chiral center at the iridium atom, two atropisomers were detected by NMR spectroscopy in a ration of **A**:**B** 54:46. Atropisomer **A** precipitated from a concentrated THF solution after stirring for 16 h at 90 °C. The precipitate was isolated and washed with diethyl ether (10 mL). After drying *in vacuo* pure atropisomer **A** was obtained. Atropisomer **A** converts slowly to isomer **B** in CH_2Cl_2 at ambient temperature over 6 d, giving back the initially obtained isomer mixture. The limited stability of atropisomer **A** allowed the NMR spectroscopic characterization. NMR data for atropisomer **A**: ^{1}H NMR (500 MHz, CD_2Cl_2): δ = 11.18 (s, 1H, H24), 7.69 (d, $^3J_{HH}$ = 8.3 Hz, 1H, H27), 7.64–7.52 (m, 14H, H36$_A$, H36$_C$, H38), 7.45–7.38 (m, 9H, H8, H19, H34$_C$, H35$_A$, H35$_B$), 7.34–7.22 (m, 19H, H36$_B$, H39, H40), 7.21–7.13 (m, 8H,

H9, H15, H28, H29, H30, $H34_A$), 7.12–7.03 (m, 5H, H7, H18, $H35_B$), 6.80–6.69 (m, 3H, H6, $H34_B$), 6.56–6.51 (m, 2H, H14), 5.48 (d, $^2J_{HH}$ = 16.6 Hz, 1H, H12), 5.21–5.06 (m, 3H, H10, H21), 4.67 (d, $^2J_{HH}$ = 16.6 Hz, 1H, H12), 4.43 (dq, $^3J_{HH}$ = 6.9, $^2J_{HH}$ = 13.8 Hz, 1H, H10), 1.60 (t, $^3J_{HH}$ = 6.9 Hz, 3H, H11), 1.50 (d, $^4J_{PH}$ = 2.3 Hz, 15H, H32). ^{13}C{H} NMR (126 MHz, CD_2Cl_2): δ (ppm) = 171.8 (t, $^2J_{CP}$ = 8.7 Hz, C23), 159.5 (d, $^2J_{CP}$ = 12.1 Hz, C2), 140.8 (C17), 139.5 (C16), 136.7 (C4), 134.8 (C5), 134.8 (d, $^2J_{CP}$ = 10 Hz, $C34_C$), 134.6 (t, $^{2/4}J_{CP}$ = 6.3 Hz, C38), 134.4 (C25), 134.0 (C20), 133.9 (d, $^2J_{CP}$ = 10 Hz, $C34_A$), 133.9 (d, $^2J_{CP}$ = 10 Hz, $C34_B$), 133.8 (C13), 133.2 (C26), 132.5 ($C36_A$), 131.9 ($C36_C$), 131.5 (C40), 131.4 ($C36_B$), 131,1 (d, $^1J_{CP}$ = 44.7 Hz, $C33_A$), 130.6 (d, $^1J_{CP}$ = 45.3 Hz, $C33_C$), 130.5 (d, $^1J_{CP}$ = 49.2 Hz, $C33_B$), 129.6 (d, $^3J_{CP}$ = 11 Hz, $C35_A$), 129.4 (C19), 129.0 (C39), 129.0 (d, $^{1/3}J_{CP}$ = 25 Hz, C37), 128.7 (d, $^3J_{CP}$ = 11 Hz, $C35_C$), 128.5 (d, $^3J_{CP}$ = 11 Hz, $C35_B$), 127.6 (C15), 127.5 (C18), 126.8 (C14), 124.9 (C8), 124.4 (C29), 123.9 (C7), 123.5 (C28), 113.8 (C30), 113.6 (C6), 112.2 (C27), 110.2 (C9), 98.1 (d, $^2J_{CP}$ = 2.3 Hz, C31), 53.8 (C12), 52.8 (C21), 44.7 (C10), 16.6 (C11), 9.5 (C32). ^{31}P{H} NMR (162 MHz, CD_2Cl_2): δ = 21.0 (Pd-P), –2.1 (Ir-P). ^{19}F NMR (376 MHz, CD_2Cl_2): δ (ppm) = –151.6 (s). ^{11}B NMR (128 MHz, CD_2Cl_2): δ (ppm) = –1.0 (s).

Selected NMR data for atropisomer **B** obtained from the isomer mixture: ^{1}H NMR (500 MHz, CD_2Cl_2): δ = 11.17 (s, 1H, H24), 6.53 (d, $^2J_{HH}$ = 16.1 Hz, 1H, H12), 5.44 (d, $^2J_{HH}$ = 16.1 Hz, 1H, H12), 5.12 (s, 2H, H21), 4.43–4.33 (m, 1H, H10), 3.85 (dq, $^3J_{HH}$ = 7.2, $^2J_{HH}$ = 14.4 Hz, 1H, H10), 1.56 (d, $^4J_{PH}$ = 2.3 Hz, 15H, H32), 1.02 (t, $^3J_{HH}$ = 7.2, 1H, H11). ^{13}C{H} NMR (126 MHz, CD_2Cl_2): δ = 171.8 (t, $^2J_{CP}$ = 8.7 Hz, C23), 159.6 (d, $^2J_{CP}$ = 11.7 Hz, C2), 97.8 (d, $^2J_{CP}$ = 2.3 Hz, C31), 53.5 (C12), 52.8 (C21), 45.8 (C10), 14.4 (C11), 9.5 (C32). ^{31}P{H} NMR (162 MHz, CD_2Cl_2): δ = 20.8 (Pd-P), –2.4 (Ir-P). ^{19}F NMR (376 MHz, CD_2Cl_2): δ = –151.6 (s). ^{11}B NMR (128 MHz, CD_2Cl_2): δ = –1.0 (s).

Mass spectrometry (identical mass for both atropisomers). MS (ESI, positive ions) *m/z* (%) = 867.2049 (100, calcd for [**18**]$^{2+}$ 867.2059).

2. X-ray crystallography

2.1. General remarks

X-ray diffraction data were collected at $T = 100$ K with a Bruker AXS APEXII CCD diffractometer equipped with a microsource using Goebel mirror monochromated Mo Kα radiation ($\lambda = 0.71073$ Å). Semiemperical multi-scan absorption corrections were applied to all data sets.[16,17] Structure solutions were found with SHELXT (intrinsic phasing)[18] and were refined with SHELXL[19] against $|F^2|$ of all data using first isotropic and later anisotropic thermal parameters for all non-hydrogen atoms (for exceptions see description of the individual molecular structures). Hydrogen atoms were added to the structure models on calculated positions (except when noted otherwise for selected atoms).

2.2. Crystallographic data for 1

Single crystals were obtained by cooling a saturated *n*-hexane solution of **1**. Formula $C_{33}H_{66}N_9P$, $M = 619.91$ g·mol^{-1}, colorless plate, 0.27 × 0.24× 0.78 mm^3, $a = 28.1897(8)$, $b = 18.4244(5)$, $c = 21.7162(6)$ Å, $\beta = 100.636(2)°$, $V = 11085.1(5)$ Å^3, $\rho_{calc} = 1.114$ g·cm^{-3}, $\mu = 0.109$ mm^{-1}, monoclinic, space group $C2/c$, $Z = 12$, ω- and φ-scans, 78959 measured intensities ($3.8° \leq 2\theta \leq 56.65°$), semiempirical absorption correction ($0.880 \leq T \leq 1.000$), 13787 independent ($R_{int} = 0.0425$) and 10539 observed intensities ($I \geq 2\sigma(I)$), refinement of 708 parameters against $|F^2|$ of all measured intensities with hydrogen atoms on calculated positions. $R = 0.0519$, $wR = 0.1326$, $R_{all} = 0.0712$, $wR_{all} = 0.1444$. The asymmetric unit contains 1.5 molecules of **1**, one of which is spread over two asymmetric units located on a twofold axis. The PN_3 units of both independent molecules as well as the nitrogen atoms are disordered over two positions (see Figure S1).

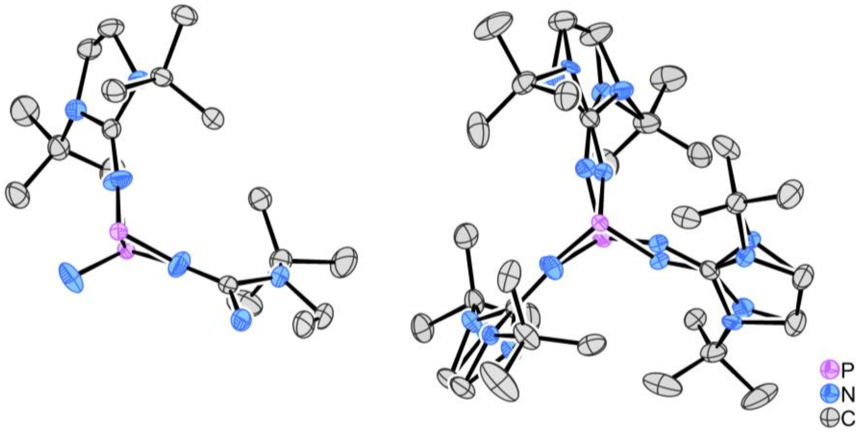

Figure S1. Contents of the asymmetric unit of **1** (1.5 molecules) with thermal ellipsoid plots at the 50% level of probability and hydrogen atoms omitted for clarity.

2.3. Crystallographic data for 1‘

Single crystals of **1’** were obtained by cooling a saturated *n*-hexane solution containing a mixture of **1** and **1’**. Formula $C_{33}H_{66}N_9P$, M = 619.91 g·mol^{-1}, colorless block, 0.60 × 0.34× 0.30 mm^3, a = 13.7379(2), b = 15.6992(2), c = 17.3874(2) Å, β = 103.0290(10)°, V = 3653.47(8) Å^3, ρ_{calc} = 1.127 g·cm^{-3}, μ = 0.110 mm^{-1}, monoclinic, space group *Cc*, Z = 4, ω- and φ-scans, 31642 measured intensities (4.0° ≤ 2θ ≤ 61.0°), semiempirical absorption correction (0.856 ≤ T ≤ 1.000), 11032 independent (R_{int} = 0.0221) and 10756 observed intensities ($I \geq 2\sigma(I)$), refinement of 406 parameters against $|F^2|$ of all measured intensities with hydrogen atoms on calculated positions. R = 0.0285, wR = 0.0745, R_{all} = 0.0295, wR_{all} = 0.0752. The asymmetric unit contains one molecule of **1’** (see Figure S2).

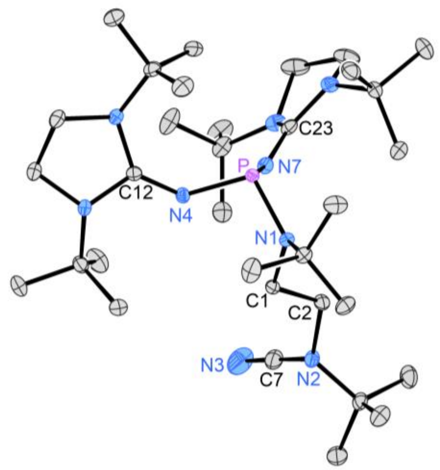

Figure S2. Molecular structure of **1’** with thermal ellipsoid plots at the 50% levels of probability and hydrogen atoms are omitted for clarity.

2.4. Crystallographic data for (1-Cl)Cl·THF

Single crystals of (**1**-Cl)Cl·THF were obtained at –40 °C from a saturated THF solution of the compound. Formula $C_{37}H_{74}N_9Cl_2OP$, M = 762.92 g·mol^{-1}, colorless block, 0.33 × 0.18× 0.17 mm^3, a = 15.9563(4), b = 16.7655(5), c = 17.4161(5) Å, β = 113.596(2)°, V = 4269.5(2) Å^3, ρ_{calc} = 1.187 g·cm^{-3}, μ = 0.229 mm^{-1}, monoclinic, space group *Cc*, Z = 4, ω- and φ-scans, 55625 measured intensities (3.5° ≤ 2θ ≤ 56.8°), semiempirical absorption correction (0.836 ≤ T ≤ 1.000), 10643 independent (R_{int} = 0.0828) and 7645 observed intensities ($I \geq 2\sigma(I)$), refinement of 469 parameters against $|F^2|$ of all measured intensities with hydrogen atoms on calculated positions. R = 0.0589, wR = 0.1444, R_{all} = 0.0906, wR_{all} = 0.1578. The asymmetric unit contains one molecule of (**1**-Cl)Cl and a THF molecule (see Figure S3).

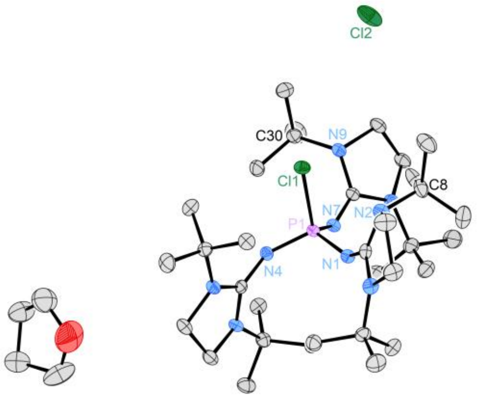

Figure S3. Contents of the asymmetric unit of (**1**-Cl)Cl·THF with thermal ellipsoid plots at the 50% levels of probability and hydrogens omitted for clarity.

2.5. Crystallographic data for (1-Cl)Cl

The molecular structure of (**1**-Cl)Cl containing no solvent molecule was also determined. Formula $C_{33}H_{66}N_9Cl_2P$, M = 690.81 g·mol^{-1}, colourless block, 0.52 × 0.38 × 0.34 mm^3, a = 9.3397(2), b = 11.9445(3), c = 18.3451(5) Å, α = 84.1130(10)°, β = 88.7370(10)°, γ = 71.1590(10)°, V = 1926.54(8) Å^3, ρ_{calc} = 1.191 g·cm^{-3}, μ = 0.245 mm^{-1}, triclinic, space group P–1, Z = 2, ω- and φ-scans, 35065 measured intensities (2.23° ≤ 2θ ≤ 61.05°), semiempirical absorption correction (0.890 ≤ T ≤ 1.000), 11646 independent (R_{int} = 0.0360) and 10777 observed intensities ($I \geq 2\sigma(I)$), refinement of 427 parameters against $|F^2|$ of all measured intensities with hydrogen atoms on calculated positions. R = 0.0348, wR = 0.0952, R_{all} = 0.0373, wR_{all} = 0.0972. The asymmetric unit contains one molecule of (**1**-Cl)Cl (see Figure S4). The chloride counterion is disordered (S = 0.5). Comparable metric parameters of cation (**1**-Cl)$^+$ in the molecular structure of (**1**-Cl)Cl are essentially identical to those found for (**1**-Cl)$^+$ in (**1**-Cl)Cl·THF.

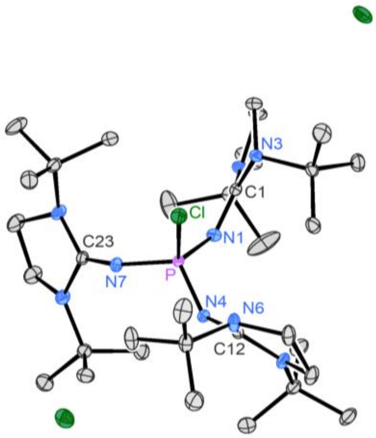

Figure S4. Contents of the asymmetric unit of (**1**-Cl)Cl with thermal ellipsoid plots at the 50% levels of probability and hydrogens omitted for clarity. Both chloride anions reside on special positions (SOF = 0.5)

2.6. Crystallographic data for [11]

Single crystals were grown by slow diffusion of diethyl ether into a saturated THF solution of [**11**] at ambient temperature. Formula $C_{41}H_{72}N_9ClIrP$, M = 949.69 g·mol^{-1}, orange plate, 0.35 × 0.24× 0.13 mm^3, a = 10.4679(6), b = 11.8606(7), c = 20.8708(13) Å, α = 80.210(2), β = 83.116(2), γ = 76.388(2)°, V = 2473.1(3) Å^3, ρ_{calc} = 1.275 g·cm^{-3}, μ = 2.821 mm^{-1}, monoclinic, space group Cc, Z = 4, ω- and φ-scans, 81992 measured intensities (3.6° ≤ 2θ ≤ 53.0°), semiempirical absorption correction (0.528 ≤ T ≤ 0.745), 10185 independent (R_{int} = 0.0665) and 9143 observed intensities ($I \geq 2\sigma(I)$), refinement of 496 parameters against $|F^2|$ of all measured intensities with hydrogen atoms on calculated positions. The effects of some strongly disordered solvent molecules were removed by the SQEEZE procedure. R = 0.0232, wR = 0.0494, R_{all} = 0.0295, wR_{all} = 0.0510. The asymmetric unit contains one molecule of [**11**] (see Figure S5).

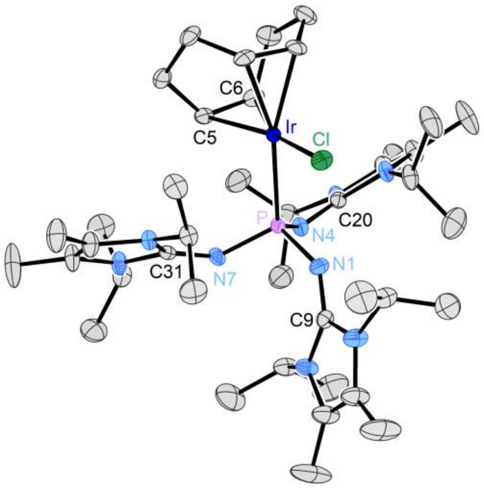

Figure S5. Molecular structure of [**11**] with thermal ellipsoid plots at the 50% levels of probability and hydrogen atoms are omitted for clarity.

2.7. Crystallographic data for [13]

Single crystals were grown by diffusion of pentane into a CH_2Cl_2 solution of [**13**] at ambient temperature. Formula $C_{40}H_{40}N_4Cl_3Ir$, M = 875.31 g·mol^{-1}, yellow plate, 0.18 × 0.15× 0.10 mm^3, a = 7.3017(2), b = 24.7872(8), c = 19.3229(6) Å, β = 90.715(2)°, V = 3496.9(2) Å^3, ρ_{calc} = 1.663 g·cm^{-3}, μ = 4.083 mm^{-1}, monoclinic, space group $P2_1/n$, Z = 4, ω- and φ-scans, 32958 measured intensities (3.3° ≤ 2θ ≤ 54.6°), semiempirical absorption correction (0.780 ≤ T ≤ 1.000), 7793 independent (R_{int} = 0.0552) and 6568 observed intensities ($I \geq 2\sigma(I)$), refinement of 439 parameters against $|F^2|$ of all measured intensities with hydrogen atoms on calculated positions. R = 0.0404, wR = 0.0997, R_{all} = 0.0503, wR_{all} = 0.1052. The asymmetric unit contains one molecule of [**13**] (see Figure S6).

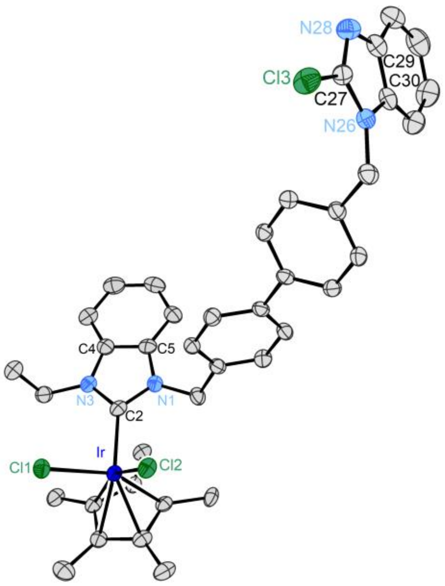

Figure S6. Molecular structures of complex [**13**] with thermal ellipsoid plots at the 50% levels of probability and hydrogen atoms are omitted for clarity.

2.8. Crystallographic data for [14]

Single crystals were grown by diffusion of pentane into a CH_2Cl_2 solution of [**14**] at ambient temperature. Formula $C_{40}H_{40}N_4Cl_3Rh$, M = 786.02 g·mol^{-1}, red plate, 0.17 × 0.13× 0.10 mm^3, a = 7.2839(3), b = 24.8808(11), c = 19.3651(8) Å, β = 91.103(3)°, V = 3508(3) Å^3, ρ_{calc} = 1.488 g·cm^{-3}, μ = 0.751 mm^{-1}, monoclinic, space group $P2_1/n$, Z = 4, ω- and φ-scans, 50042 measured intensities (3.9° ≤ 2θ ≤ 54.8°), semiempirical absorption correction (0.736 ≤ T ≤ 1.000), 7881 independent (R_{int} = 0.0847) and 5853 observed intensities ($I \geq 2\sigma(I)$), refinement of 439 parameters against $|F^2|$ of all measured intensities with hydrogen atoms on calculated positions. R = 0.0490, wR = 0.1185, R_{all} = 0.0714, wR_{all} = 0.1310. The asymmetric unit contains one molecule of [**14**] (see Figure S7).

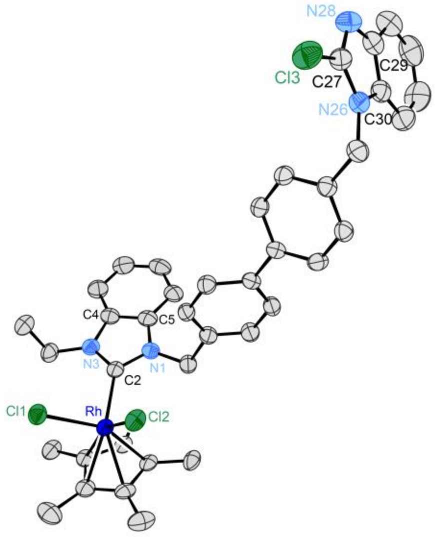

Figure S7. Molecular structure of complex [**14**] with thermal ellipsoid plots at the 50% levels of probability and hydrogen atoms are omitted for clarity.

2.9. Crystallographic data for [16]

Single crystals were grown by diffusion of pentane into a CH_2Cl_2 solution of [**16**] at ambient temperature. Formula $C_{38}H_{37}N_4Cl_2Rh$, M = 723.52 g·mol^{-1}, yellow block, 0.51 × 0.11× 0.10 mm^3, a = 9.4335(6), b = 20.1853(10), c = 18.5268(10) Å, β = 99.013(4)°, V = 3484.3(3) Å^3, ρ_{calc} = 1.379 g·cm^{-3}, μ = 0.676 mm^{-1}, monoclinic, space group $P2_1/c$, Z = 4, ω- and φ-scans, 38333 measured intensities (4.4° ≤ 2θ ≤ 53.5°), semiempirical absorption correction (0.598 ≤ T ≤ 1.000), 7318 independent (R_{int} = 0.0714) and 5808 observed intensities ($I \geq 2\sigma(I)$), refinement of 407 parameters against $|F^2|$ of all measured intensities with hydrogen atoms on calculated positions. R = 0.0569, wR = 0.1360, R_{all} = 0.0719, wR_{all} = 0.1434. The effects of some strongly disordered solvent molecules were removed by the SQEEZE procedure. The asymmetric unit contains one molecule of [**16**] (see Figure S8).

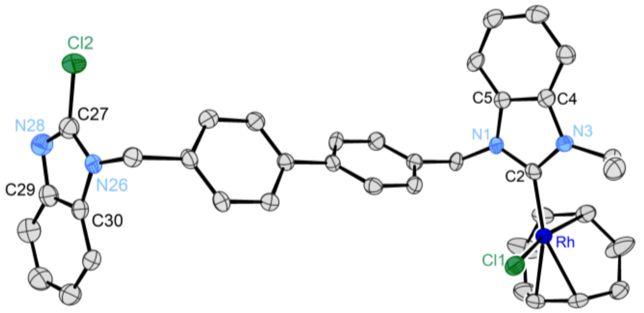

Figure S8. Molecular structure of complex [**16**] with thermal ellipsoid plots at the 50% levels of probability and hydrogen atoms are omitted for clarity.

2.10. Crystallographic data for [17']

Crystals of [**17'**] were obtained by diffusion of *n*-pentane into a saturated solution of [**17**] in dichloromethane over one week during which time the complex rearranged from [**17**] to [**17'**]. Formula $C_{30}H_{25}N_4Cl_3Ru$, M = 648.96 g·mol^{-1}, red needle, 0.246 × 0.072× 0.052 mm^3, a = 11.4755(3), b = 14.01165(3), c = 16.6181(4) Å, β = 105.8980(10)°, V = 2569.80(10) Å^3, ρ_{calc} = 1.667 g·cm^{-3}, μ = 0.952 mm^{-1}, monoclinic, space group $P2_1/c$, Z = 4, ω- and φ-scans, 33998 measured intensities (3.9° ≤ 2θ ≤ 54.3°), semiempirical absorption correction (0.859 ≤ T ≤ 1.000), 5681 independent (R_{int} = 0.0265) and 5681 observed intensities ($I \geq 2\sigma(I)$), refinement of 344 parameters against $|F^2|$ of all measured intensities with hydrogen atoms on calculated positions. R = 0.0294, wR = 0.0710, R_{all} = 0.0294, wR_{all} = 0.0721. The effects of some strongly disordered solvent molecules were removed by the SQEEZE procedure. The asymmetric unit contains one molecule of [**17'**] (see Figure S9).

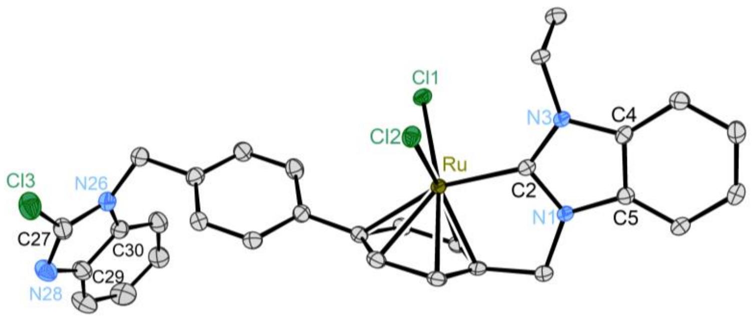

Figure S9. Molecular structure of complex [**17'**] with thermal ellipsoid plots at the 50% levels of probability and hydrogen atoms are omitted for clarity.

3. NMR Spectra

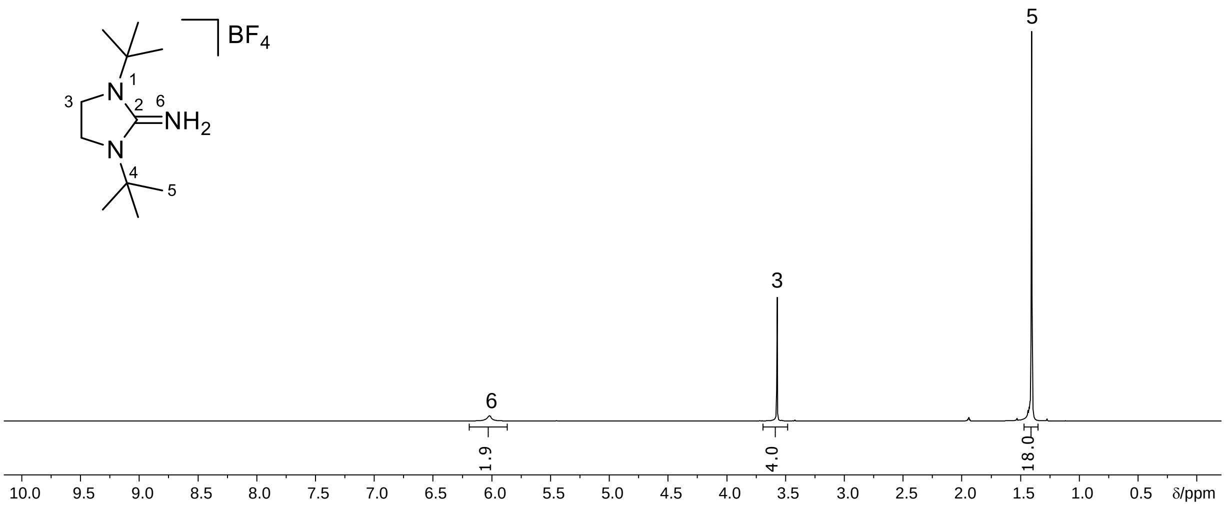

Figure S10. ^{1}H NMR spectrum of $R^{1}H{\cdot}HBF_4$ (500 MHz, CD_3CN).

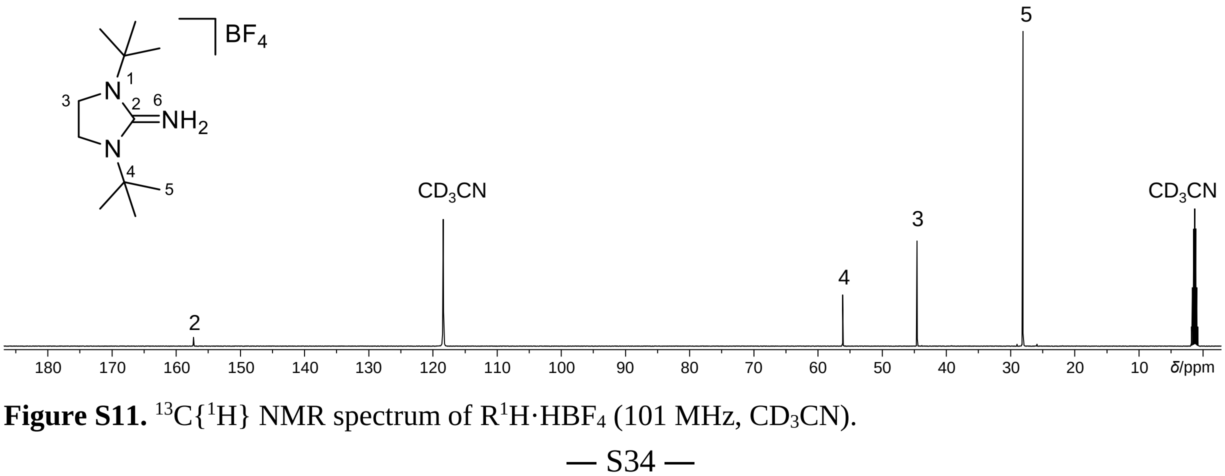

Figure S11. $^{13}C\{^{1}H\}$ NMR spectrum of $R^{1}H{\cdot}HBF_4$ (101 MHz, CD_3CN).

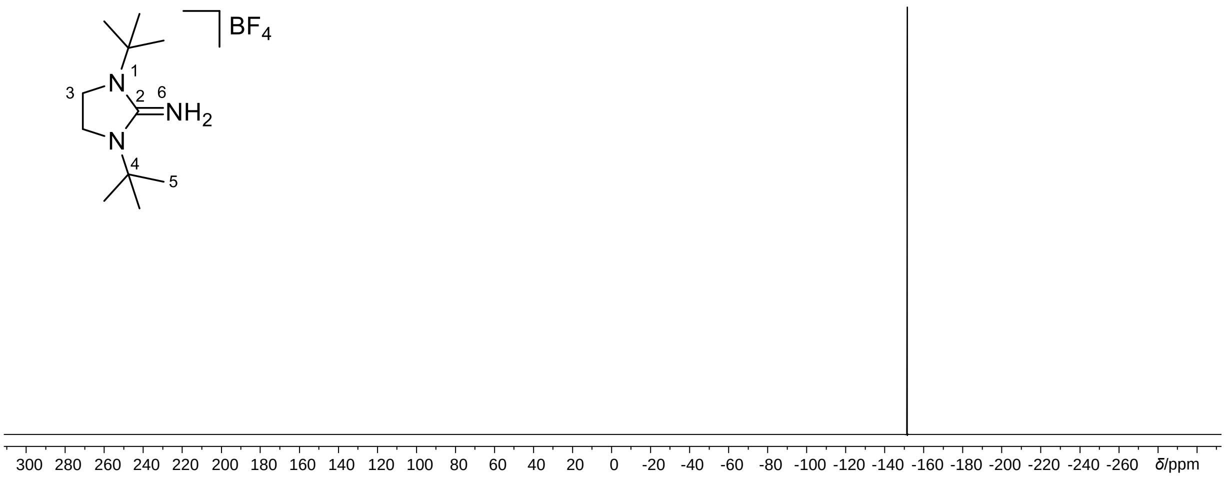

Figure S12. ^{19}F NMR spectrum of R^{1}H·HBF$_4$ (471 MHz, CD$_3$CN).

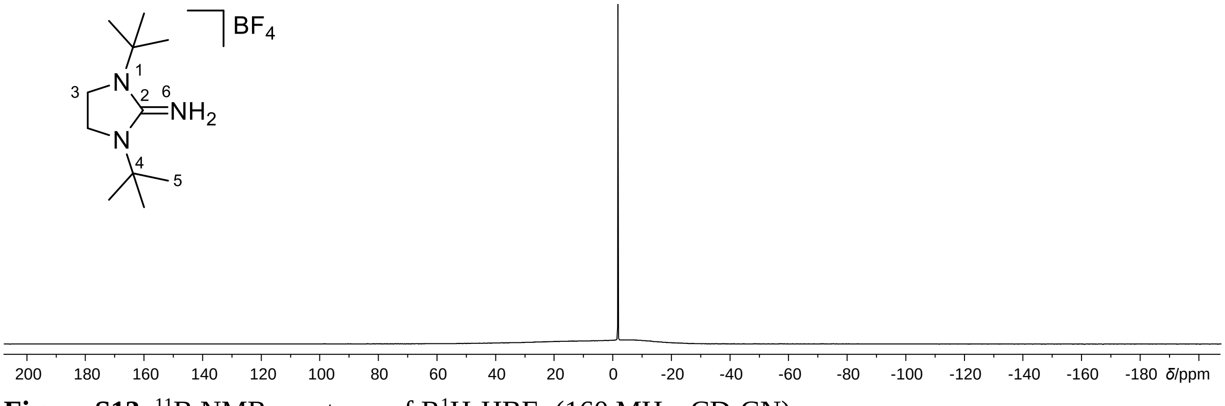

Figure S13. ^{11}B NMR spectrum of R^{1}H·HBF$_4$ (160 MHz, CD$_3$CN).

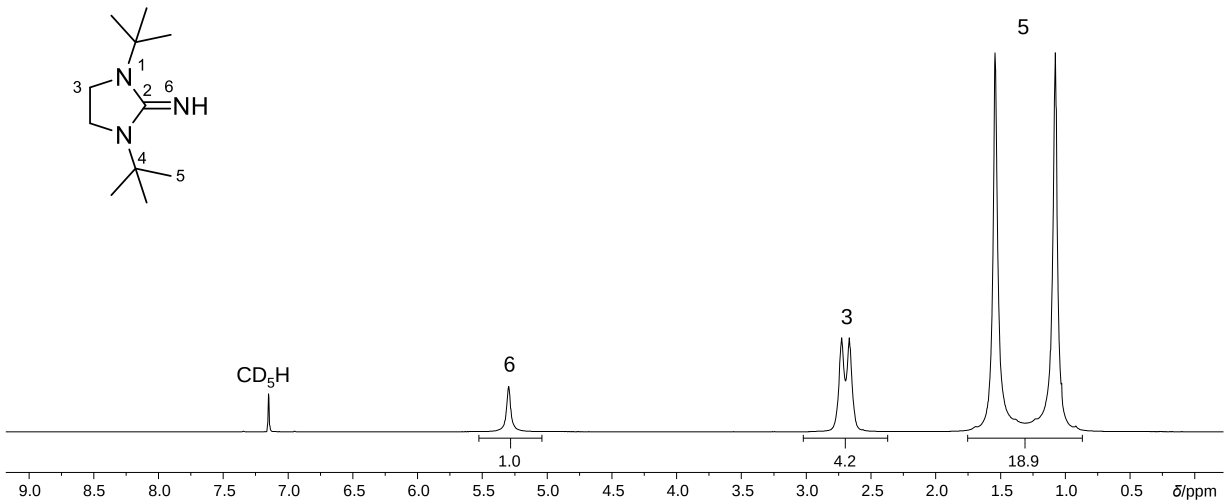

Figure S14. ^{1}H NMR spectrum of $R^{1}H$ (400 MHz, C_6D_6).

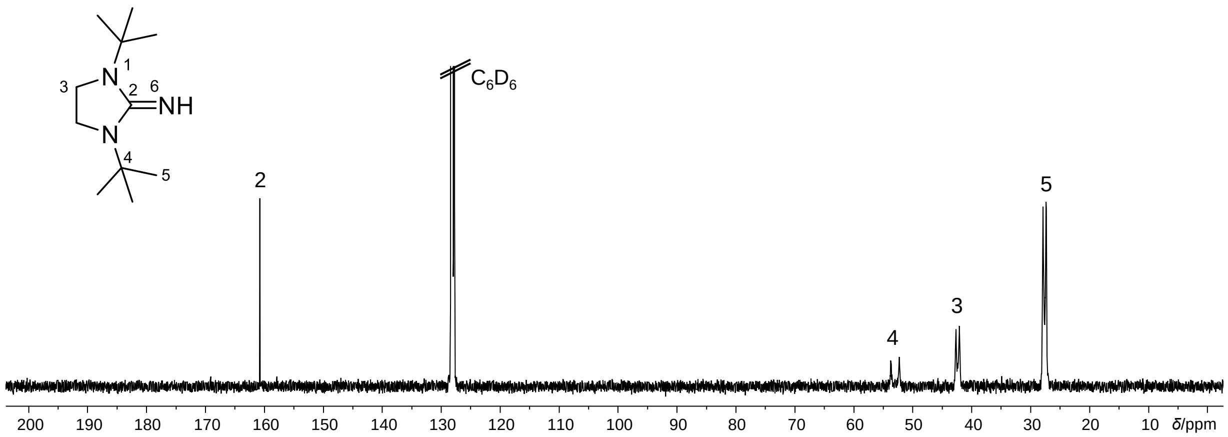

Figure S15. $^{13}C\{^{1}H\}$ NMR spectrum of $R^{1}H$ (101 MHz, C_6D_6).

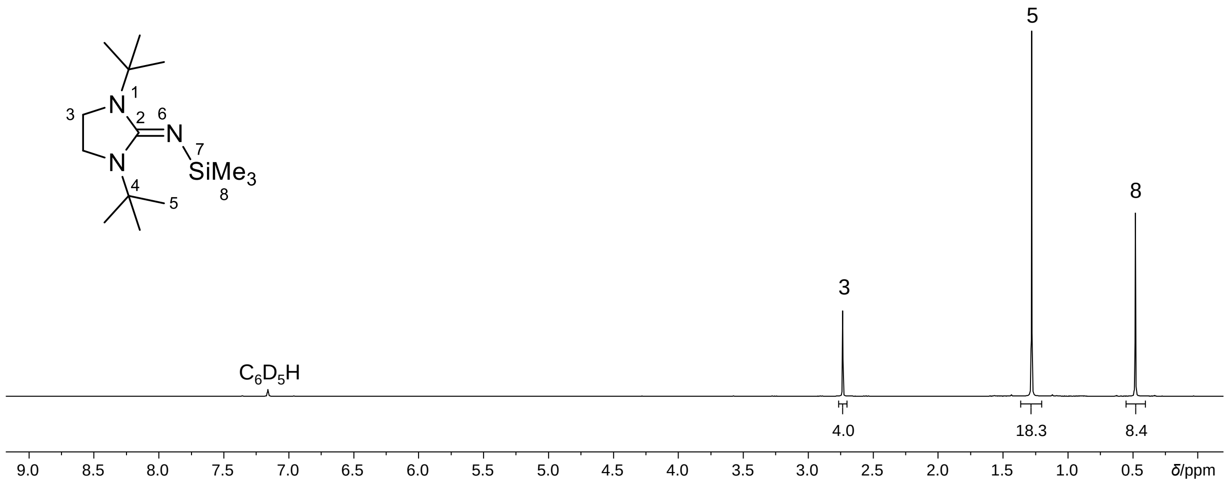

Figure S16. ^{1}H NMR spectrum of R^{1}SiMe$_3$ (500 MHz, C_6D_6).

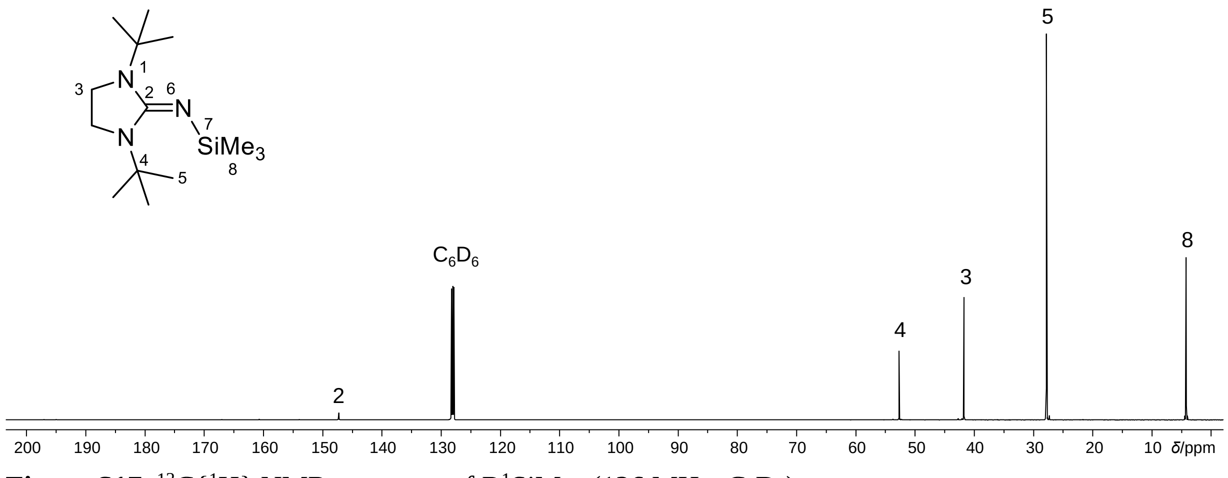

Figure S17. ^{13}C{^{1}H} NMR spectrum of R^{1}SiMe$_3$ (126 MHz, C_6D_6).

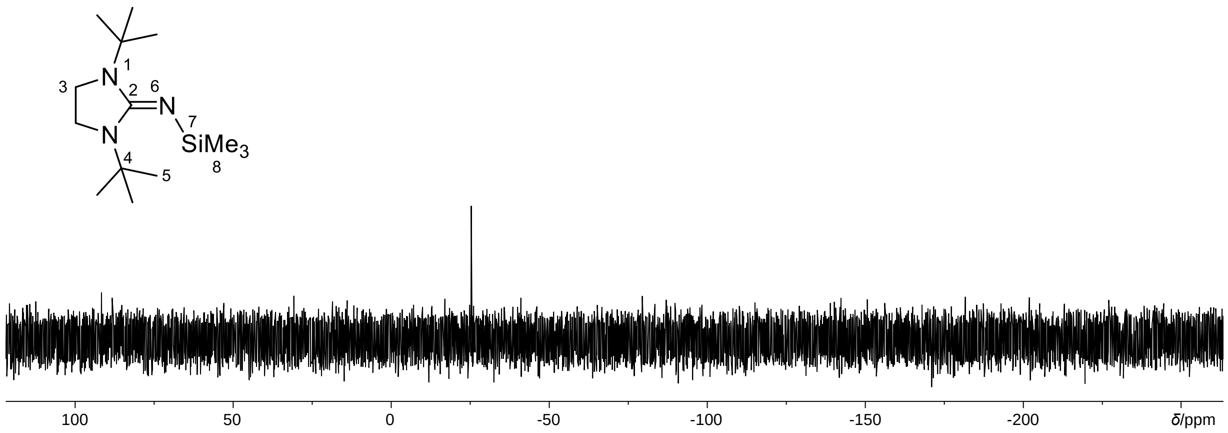

Figure S18. $^{29}Si\{^1H\}$ DEPT(19.5) NMR spectrum of R^1SiMe_3 (79 MHz, C_6D_6).

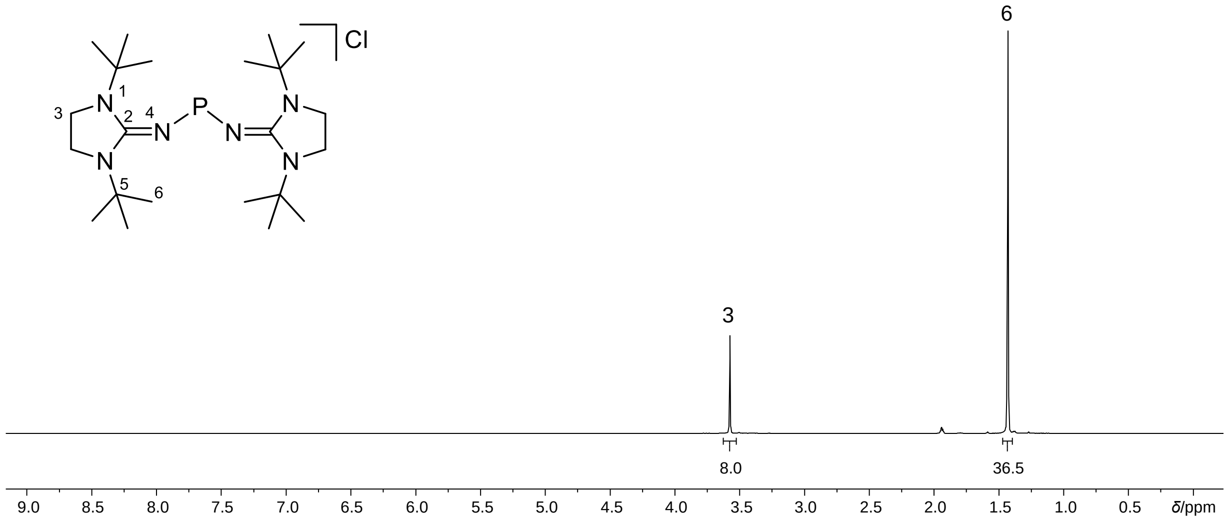

Figure S19. ^1H NMR spectrum of $(R^1{}_2P)Cl$ (400 MHz, CD_3CN).

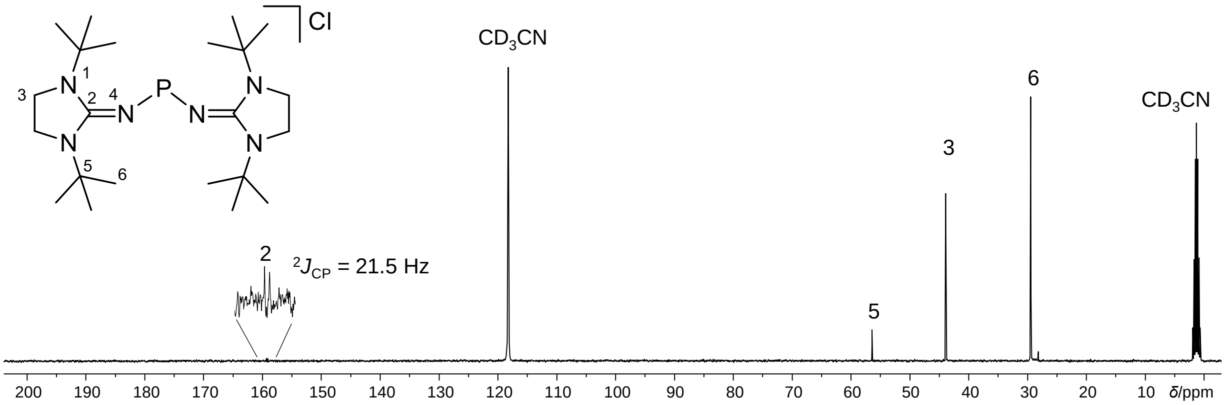

Figure S20. $^{13}C\{^{1}H\}$ NMR spectrum of ($R^{1}_{2}P$)Cl (101 MHz, CD_3CN).

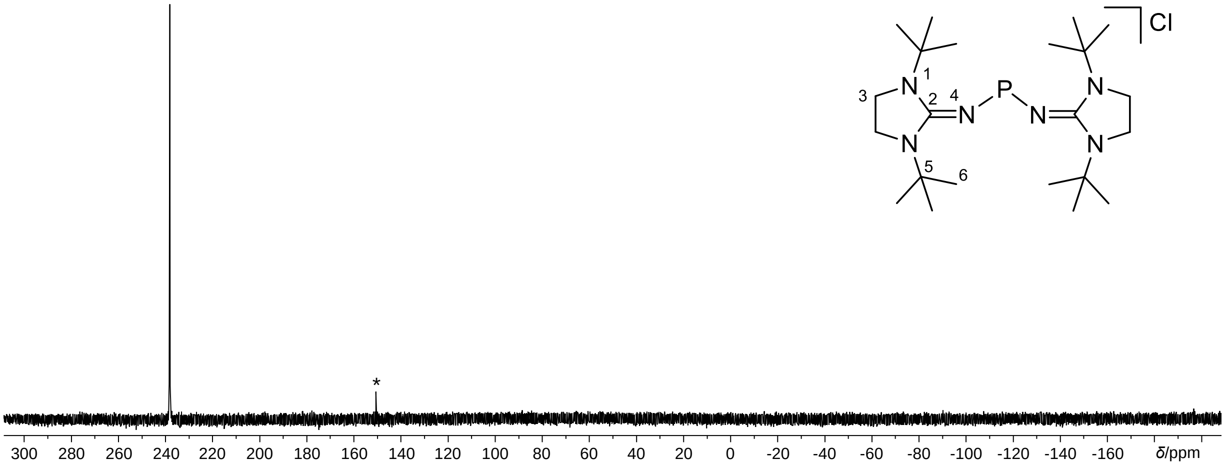

Figure S21. ^{31}P NMR spectrum of ($R^{1}_{2}P$)Cl (162 MHz, CD_3CN). * Artificial signal from NMR console.

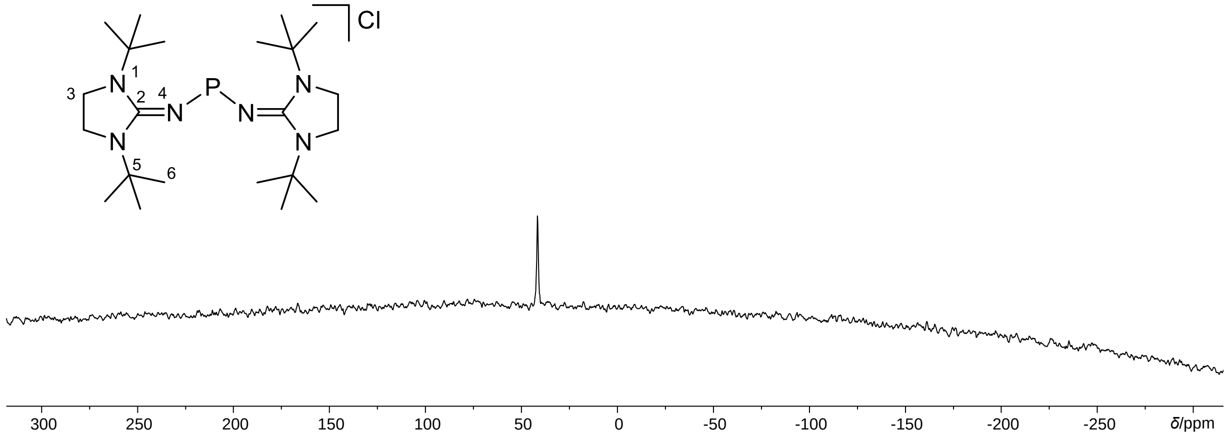

Figure S22. ^{35}Cl NMR spectrum of ($R^1{}_2$P)Cl (39 MHz, CD_3CN).

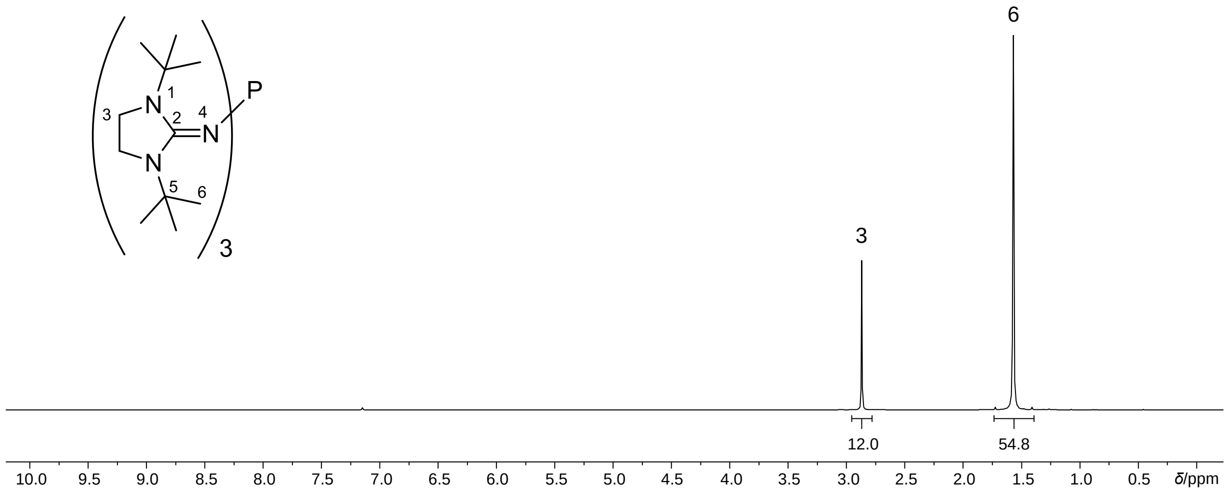

Figure S23. ^{1}H NMR spectrum of **1** (400 MHz, C_6D_6).

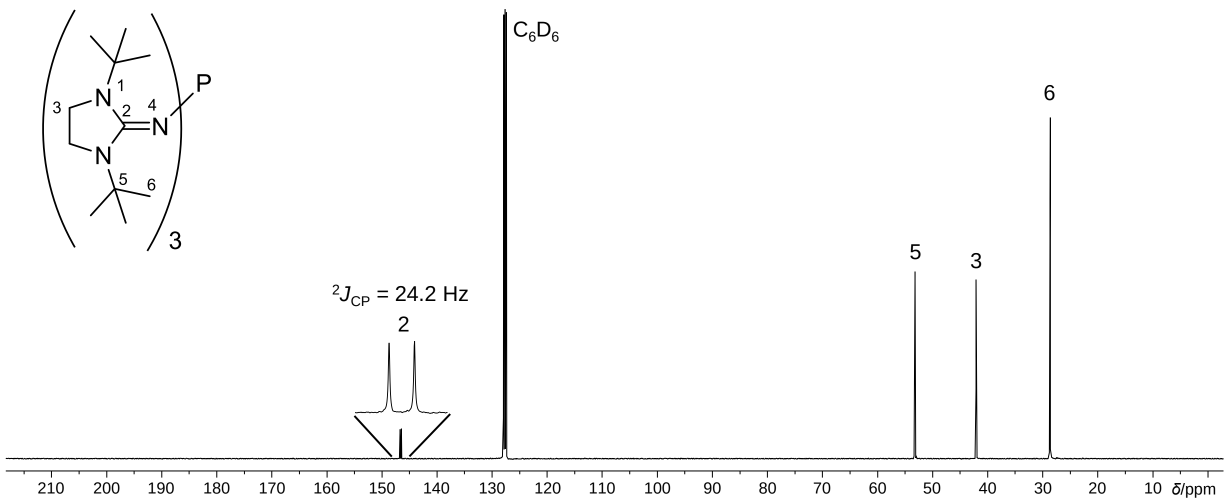

Figure S24. $^{13}C\{^1H\}$ NMR spectrum of **1** (101 MHz, C_6D_6).

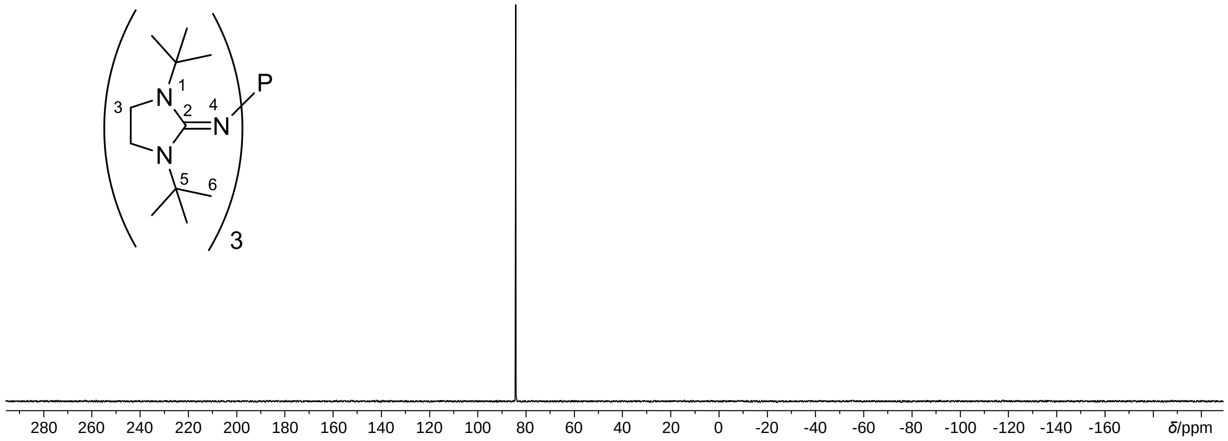

Figure S25. ^{31}P NMR spectrum of **1** (162 MHz, C_6D_6).

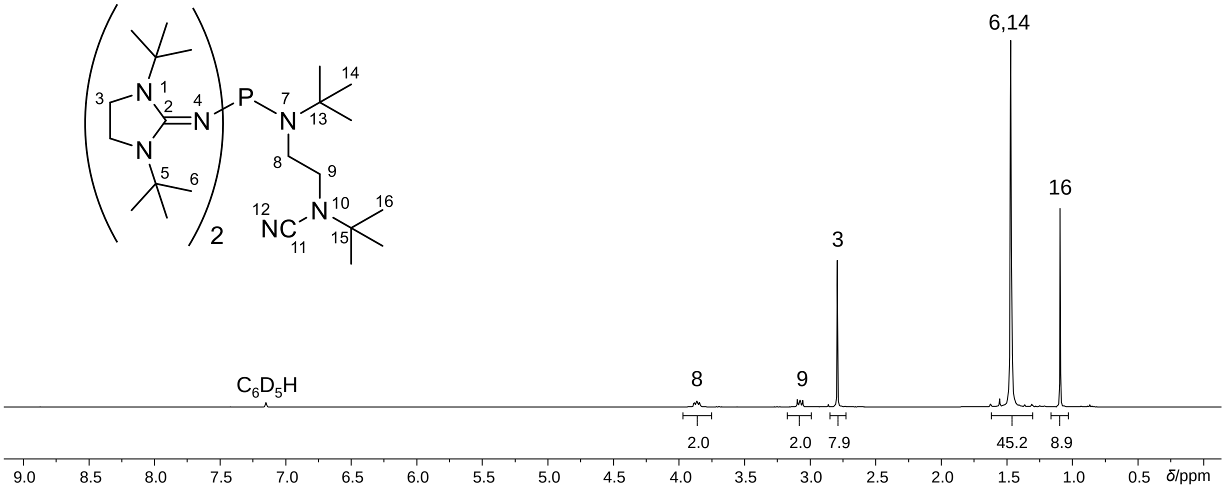

Figure S26. ^{1}H NMR spectrum of **1’** (400 MHz, C_6D_6).

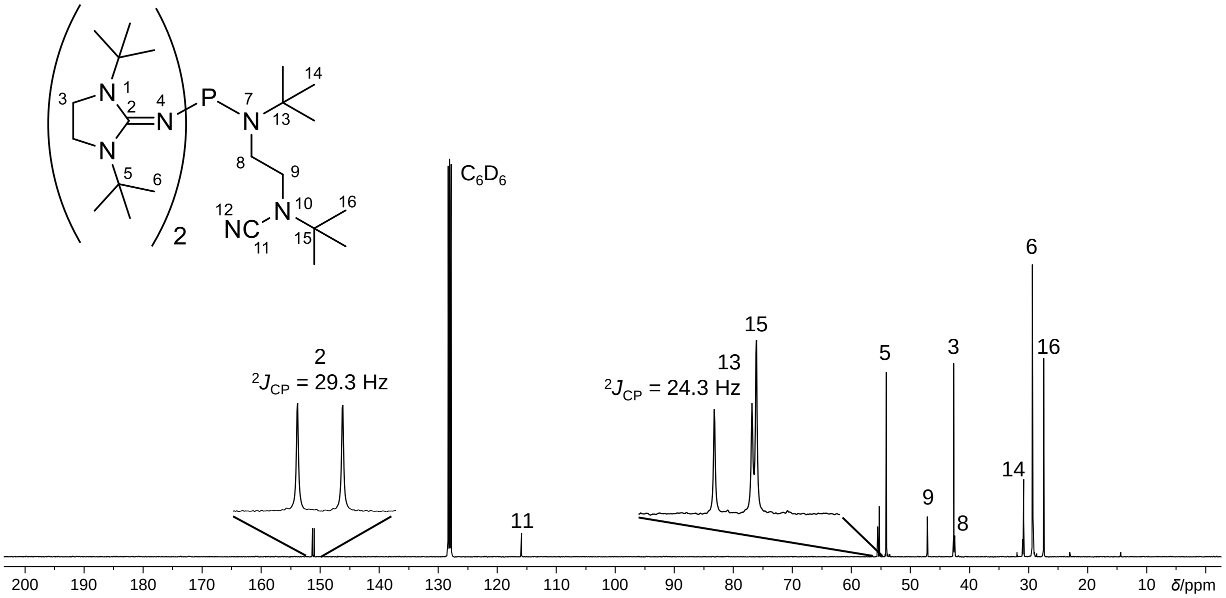

Figure S27. ^{13}C{^{1}H} NMR spectrum of **1’** (101 MHz, C_6D_6).

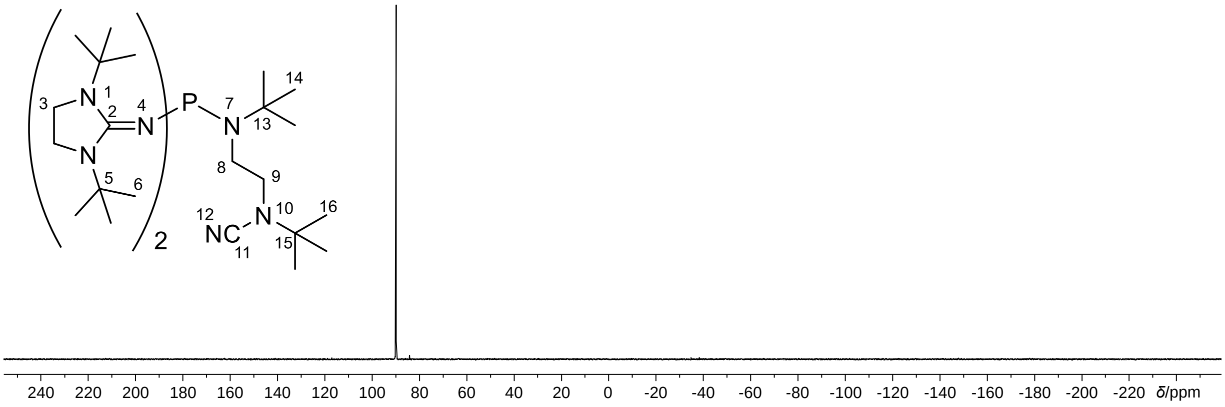

Figure S28. ^{31}P NMR spectrum of **1'** (162 MHz, C_6D_6).

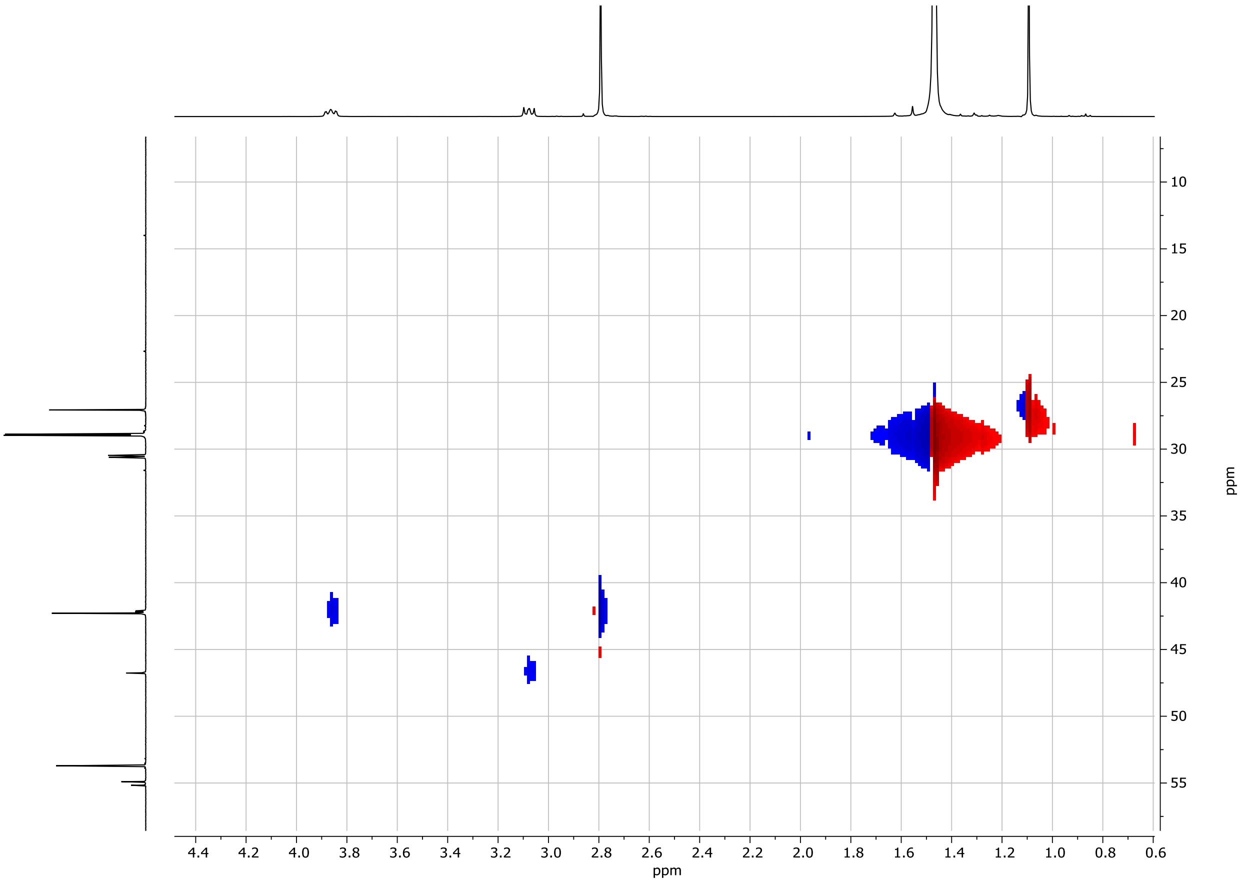

Figure S29. ^{1}H/^{13}C HSQC NMR spectrum of **1'** in C_6D_6.

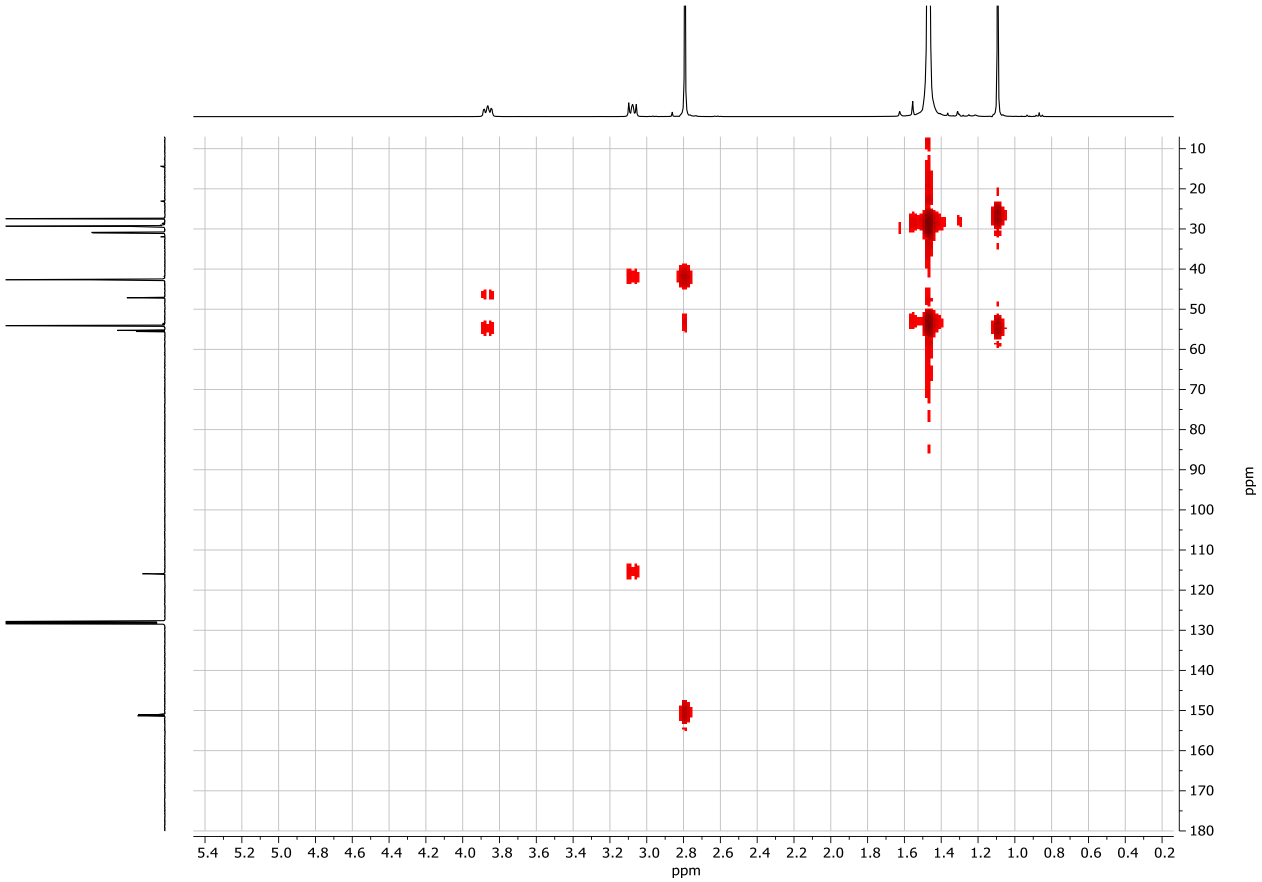

Figure S30. $^{1}H/^{13}C$ HMBC NMR spectrum of **1’** in C_6D_6.

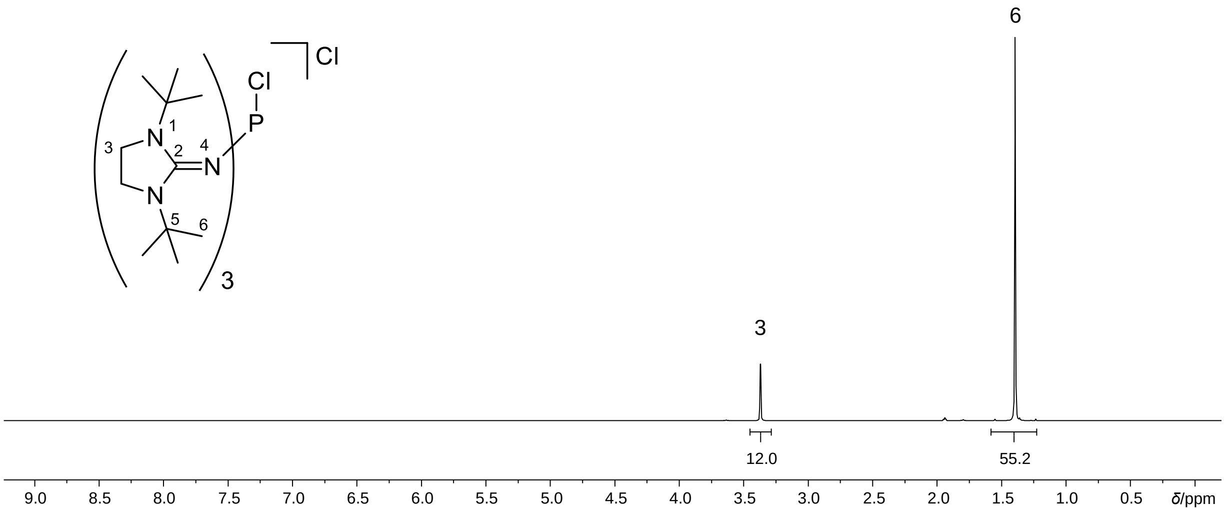

Figure S31. ^{1}H NMR spectrum of (**1**-Cl)Cl (400 MHz, CD_3CN).

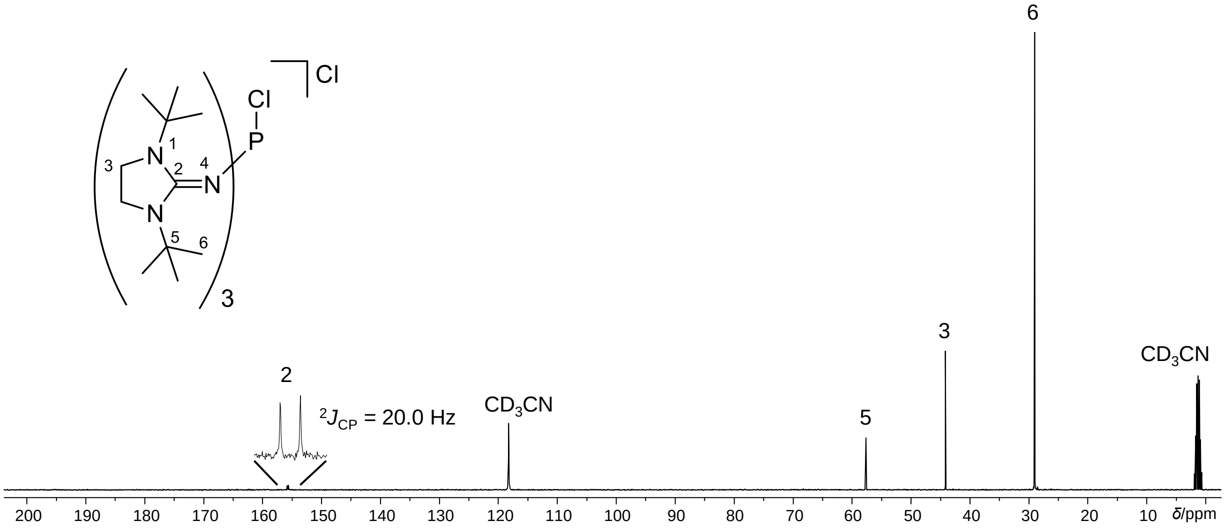

Figure S32. ^{13}C{^{1}H} NMR spectrum of (**1**-Cl)Cl (101 MHZ, CD_3CN).

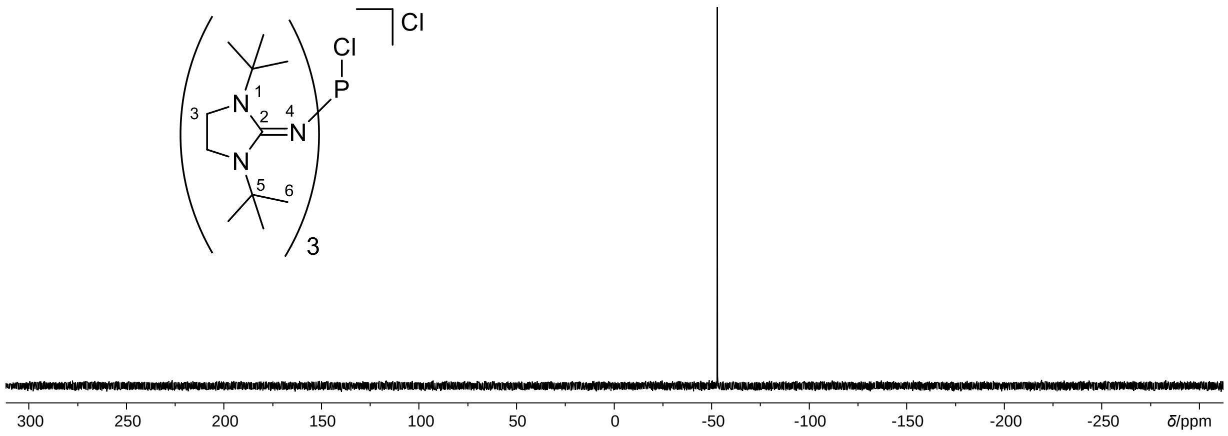

Figure S33. ^{31}P NMR spectrum of (**1**-Cl)Cl (162 MHz, CD_3CN).

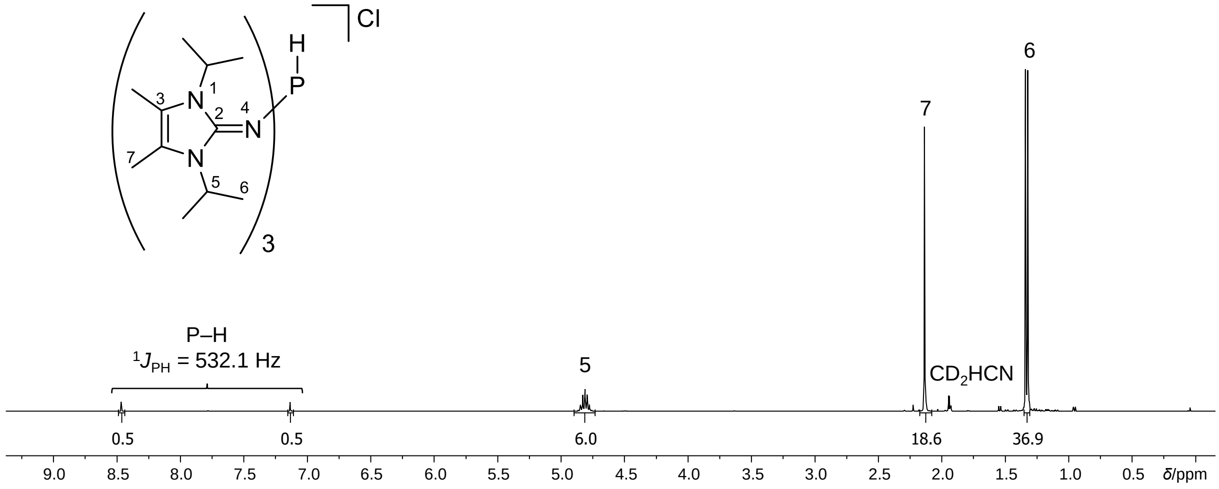

Figure S34. ^1H NMR spectrum of (**2**-H)Cl (400 MHz, CD_3CN).

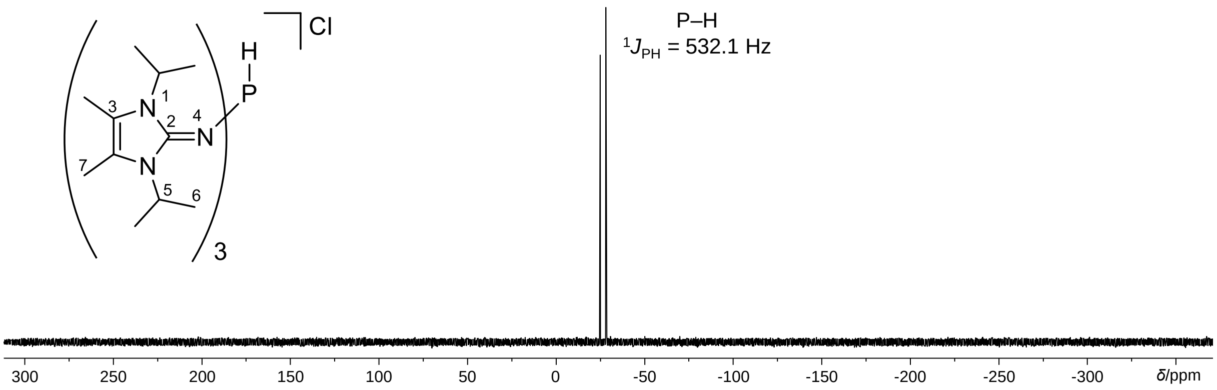

Figure S35. ^{31}P NMR spectrum of (**2**-H)Cl (162 MHz, CD_3CN).

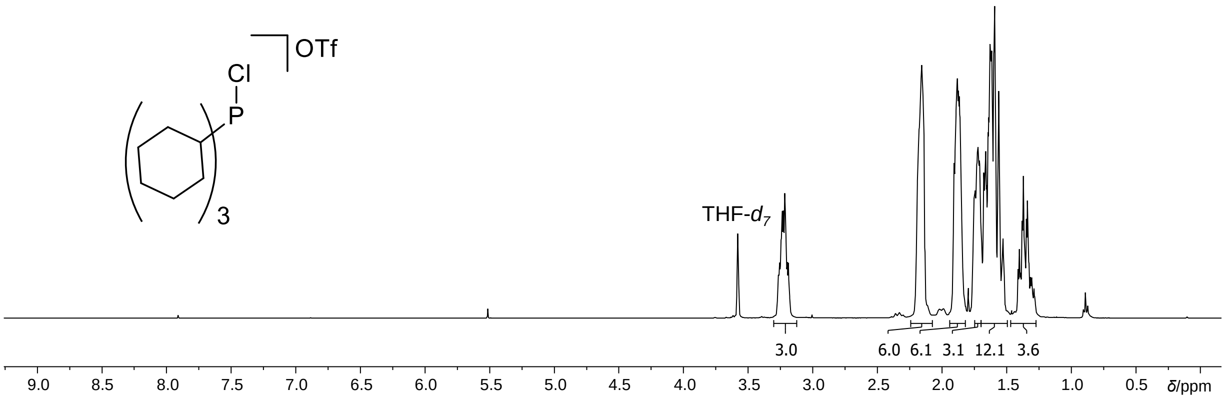

Figure S36. ^{1}H NMR spectrum of (Cy_3P-Cl)OTf (400 MHz, THF-d_8).

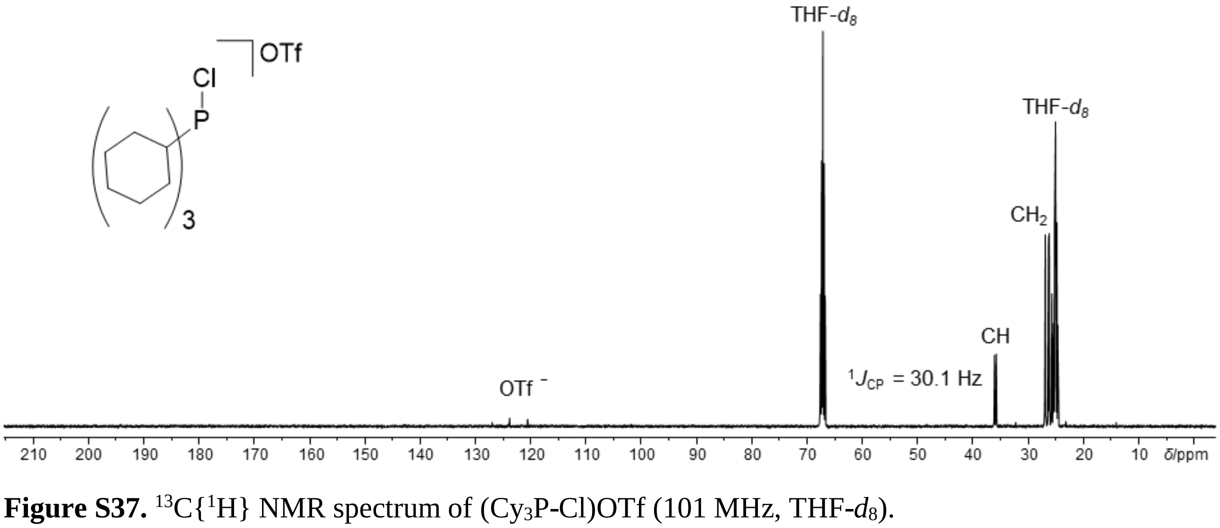

Figure S37. ^{13}C{^{1}H} NMR spectrum of (Cy_3P-Cl)OTf (101 MHz, THF-d_8).

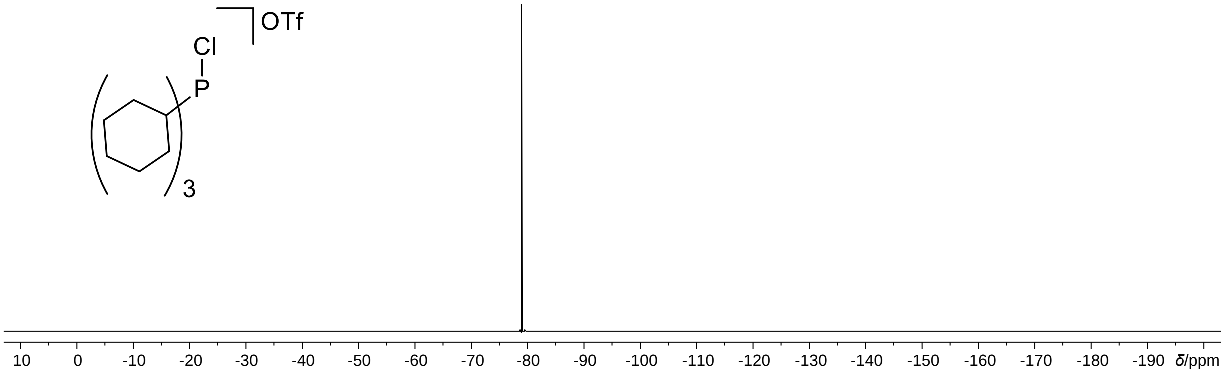

Figure S38. ^{19}F NMR spectrum of (Cy$_3$P-Cl)OTf (376 MHz, THF-d_8).

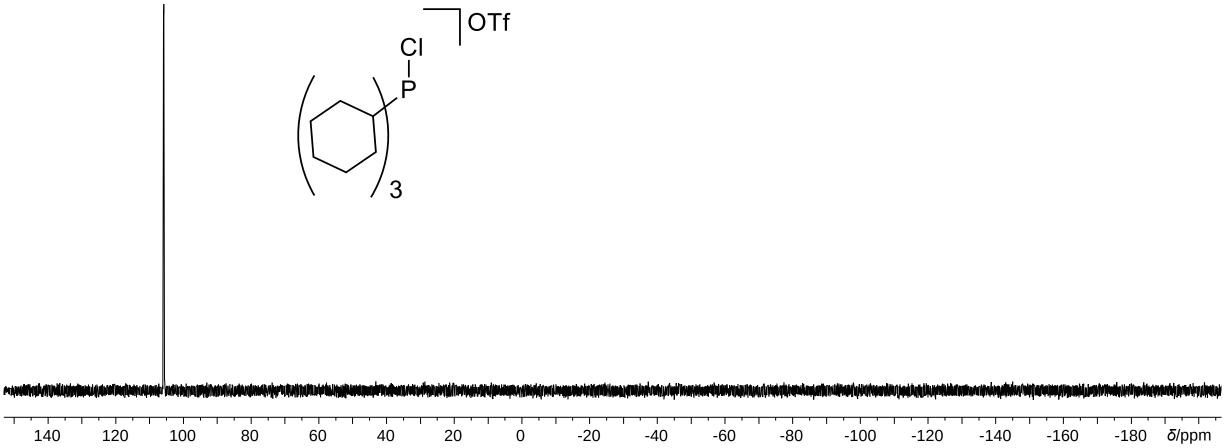

Figure S39. ^{31}P NMR spectrum of (Cy$_3$P-Cl)OTf (162 MHZ, THF-d_8).

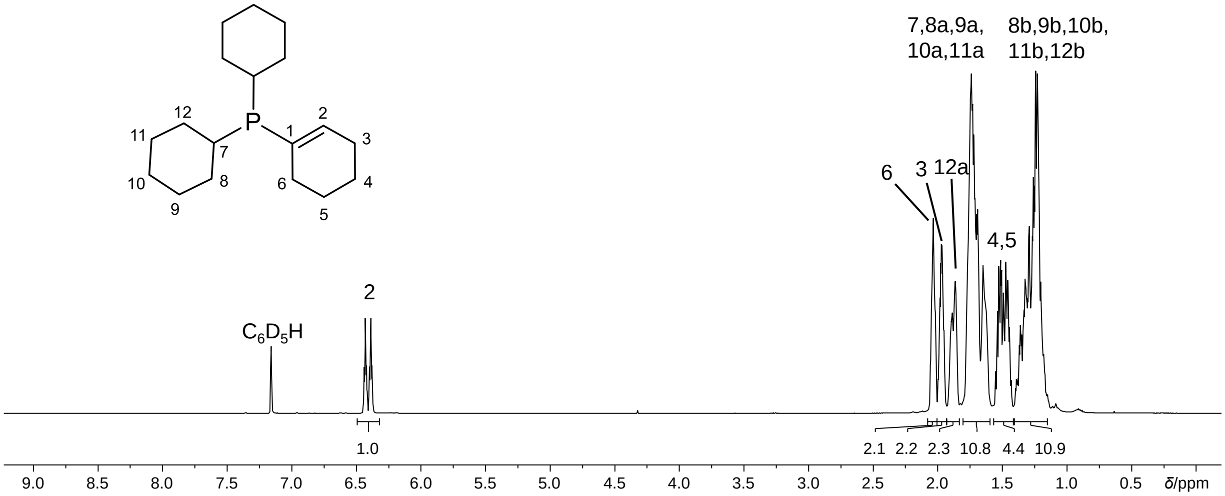

Figure S40. ^{1}H NMR spectrum of **4** (400 MHz, C_6D_6).

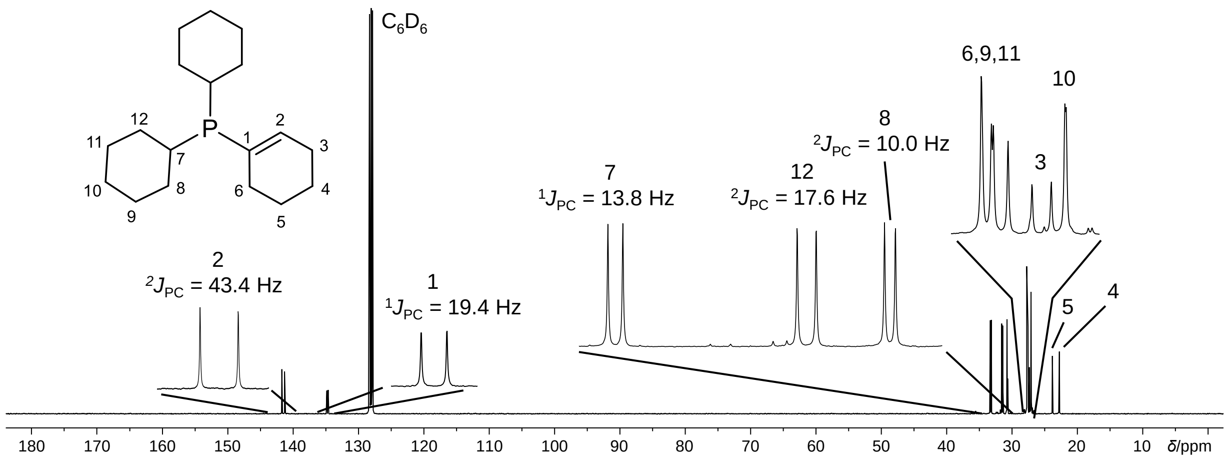

Figure S41. $^{13}C\{^{1}H\}$ NMR spectrum of **4** (101 MHz, C_6D_6).

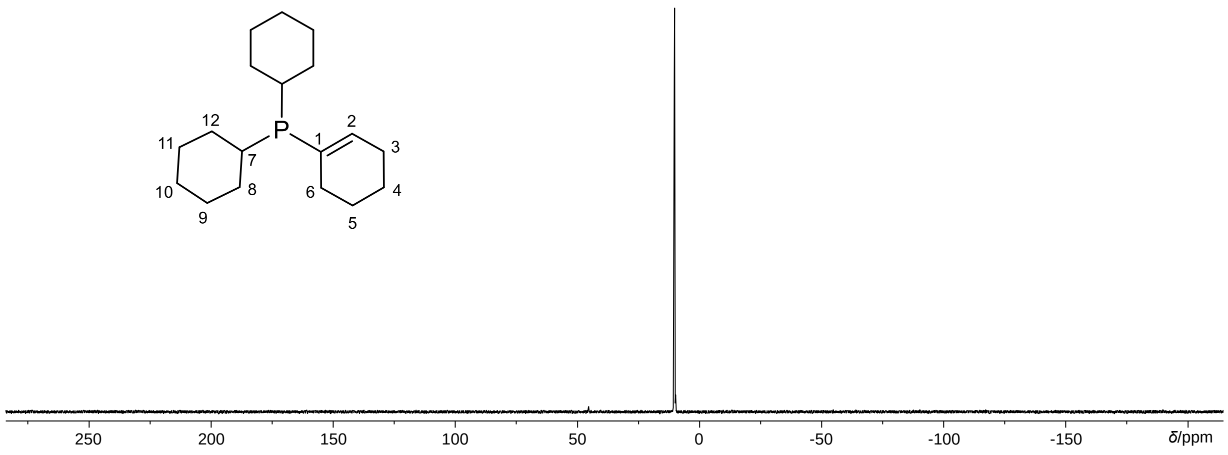

Figure S42. ^{31}P NMR spectrum of **4** (162 MHZ, C_6D_6).

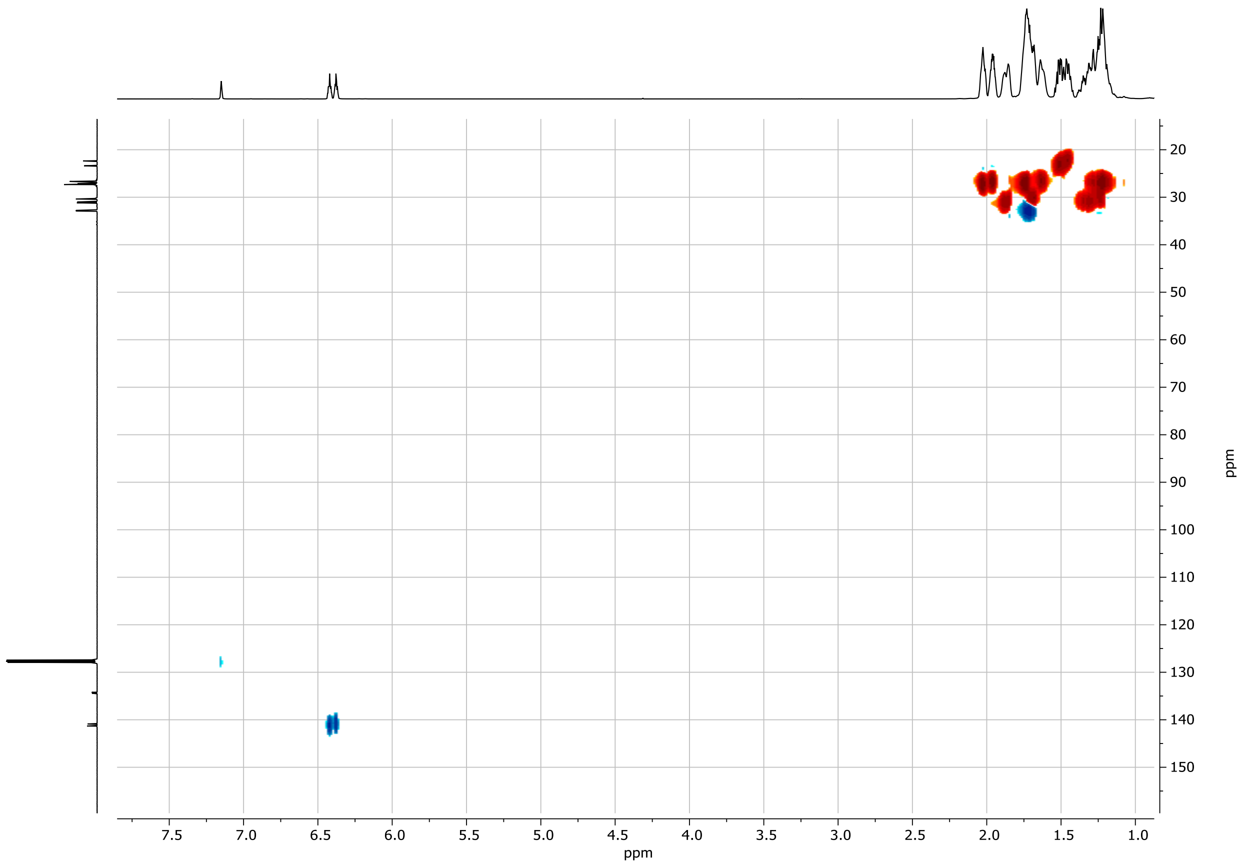

Figure S43. $^{1}H/^{13}C$ HSQC NMR spectrum of **4** in C_6D_6.

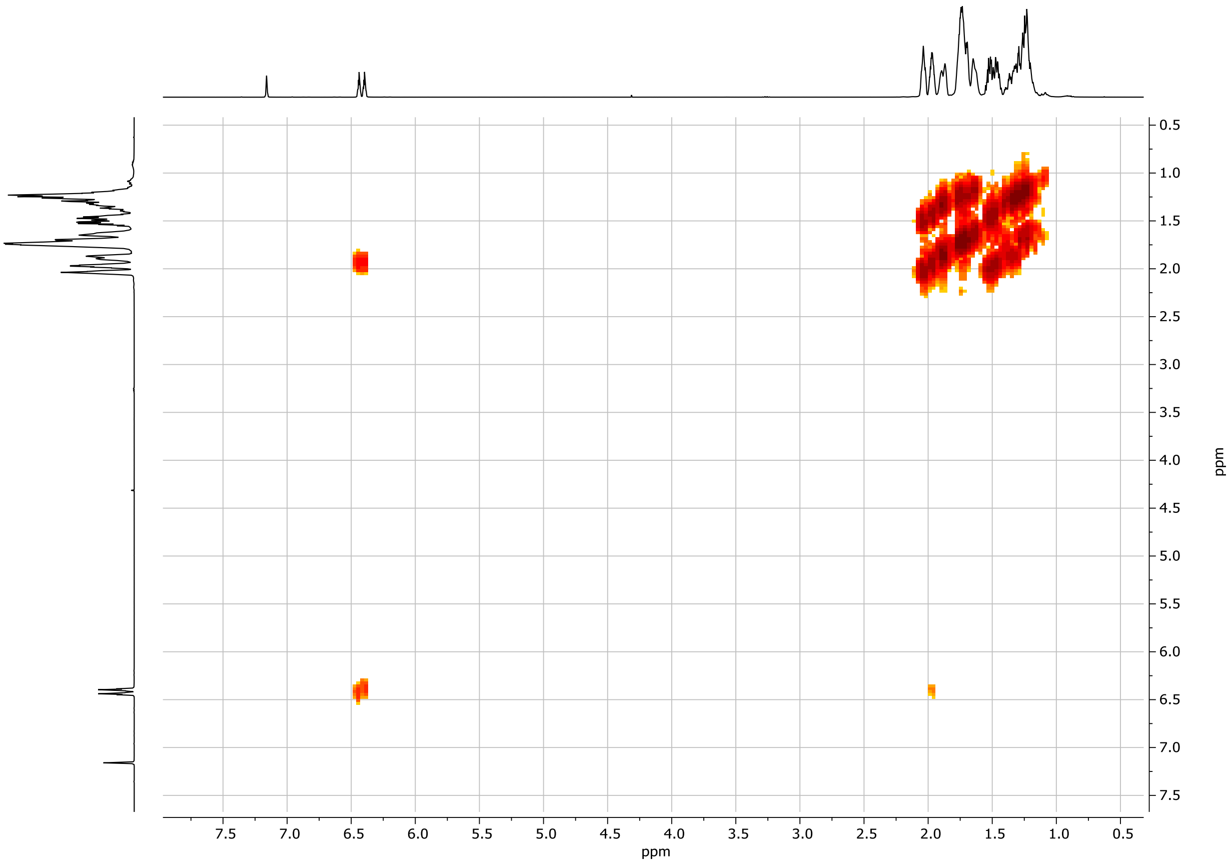

Figure S44. $^{1}H/^{1}H$ COSY NMR spectrum of **4** in C_6D_6.

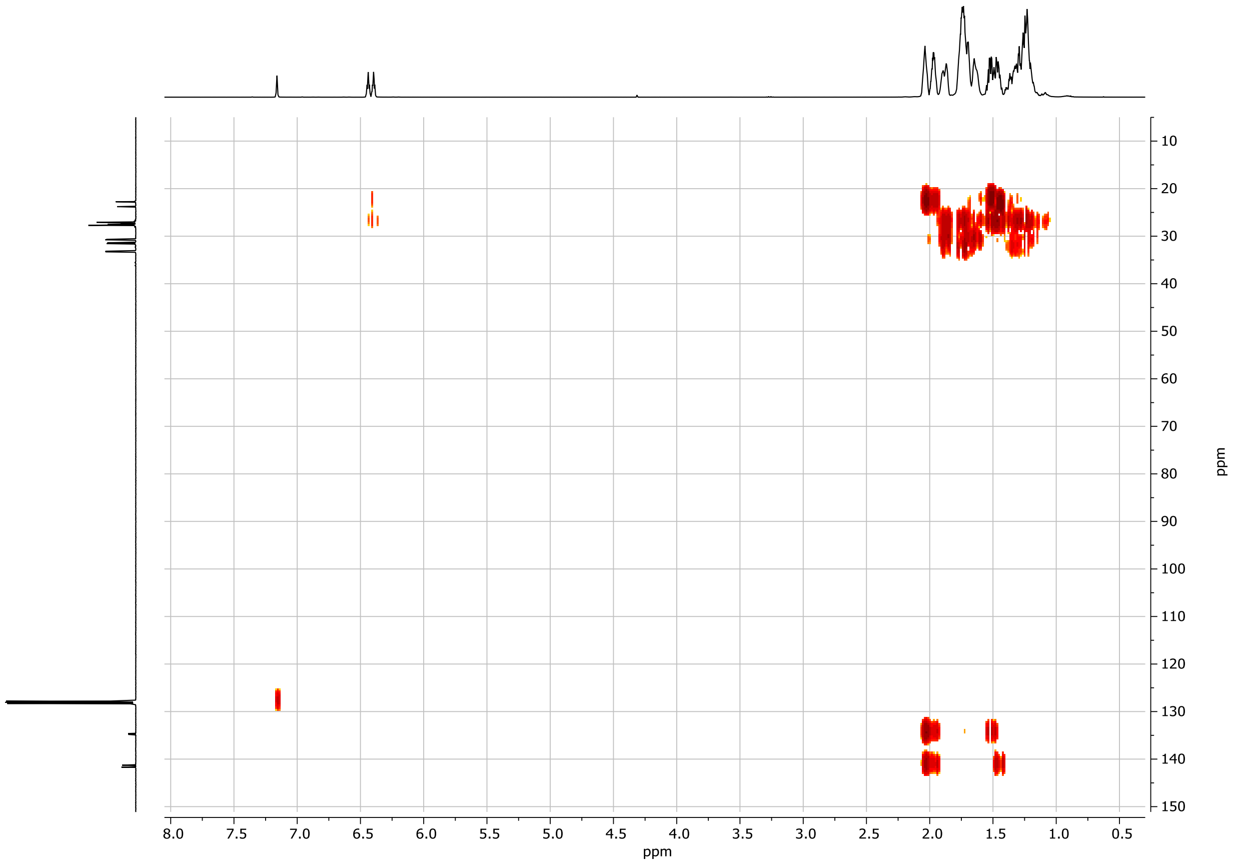

Figure S45. ^{1}H/^{13}C HMBC NMR spectrum of **4** in C_6D_6.

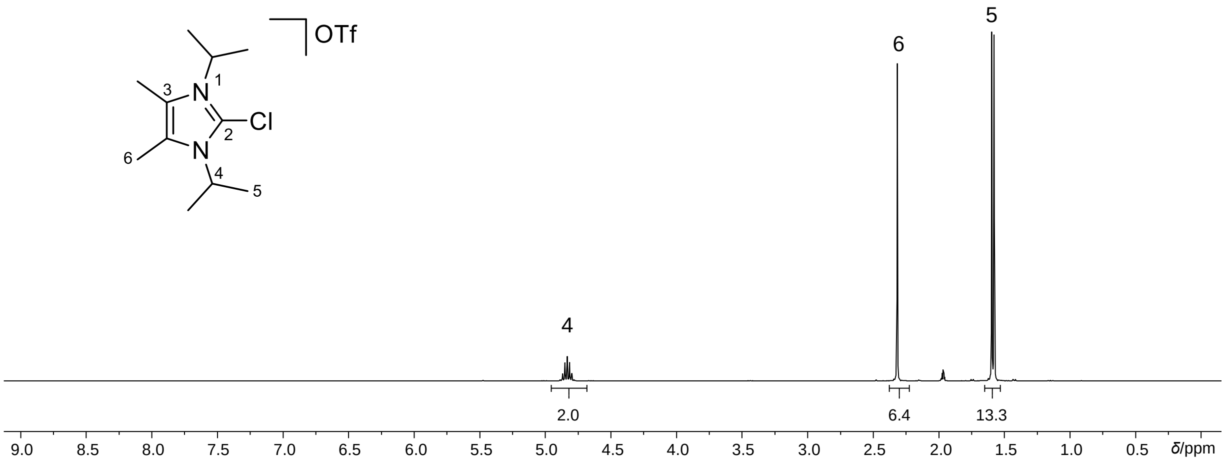

Figure S46. ^{1}H NMR spectrum (**3**-Cl)OTf (400 MHz, CD_3CN).

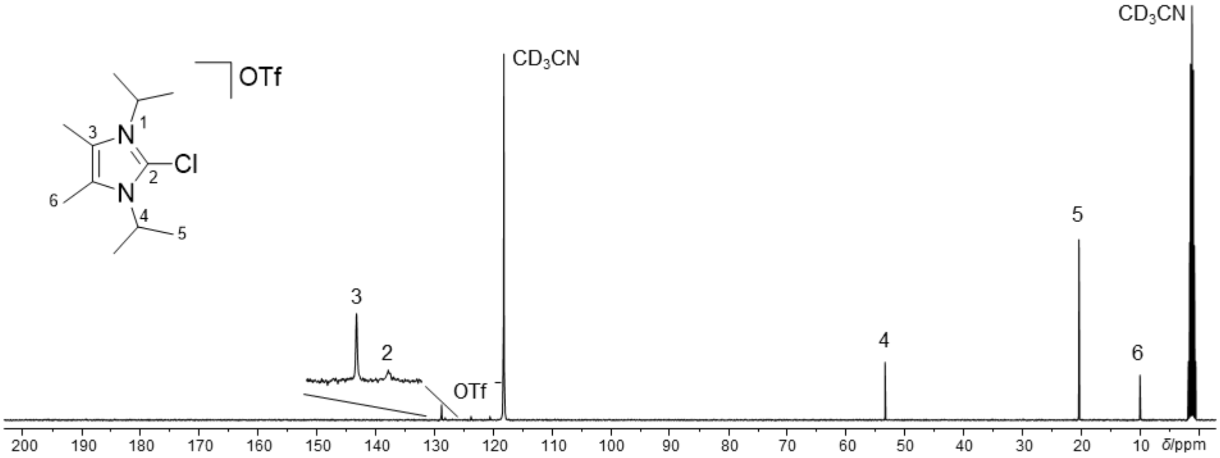

Figure S47. $^{13}C\{^{1}H\}$ NMR spectrum of (**3**-Cl)OTf (101 MHz, CD_3CN).

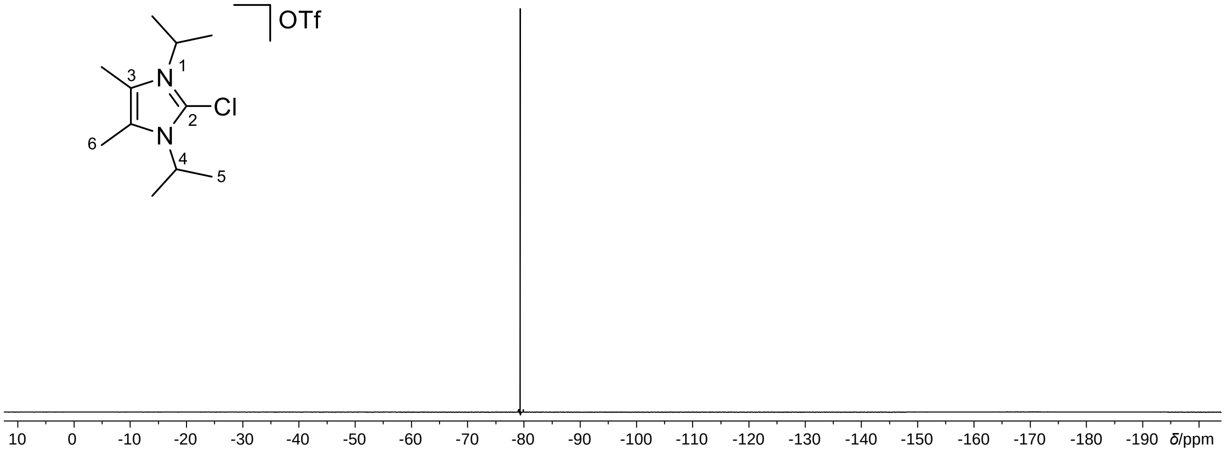

Figure S48. ^{19}F NMR spectrum of (**3**-Cl)OTf (376 MHz, CD_3CN).

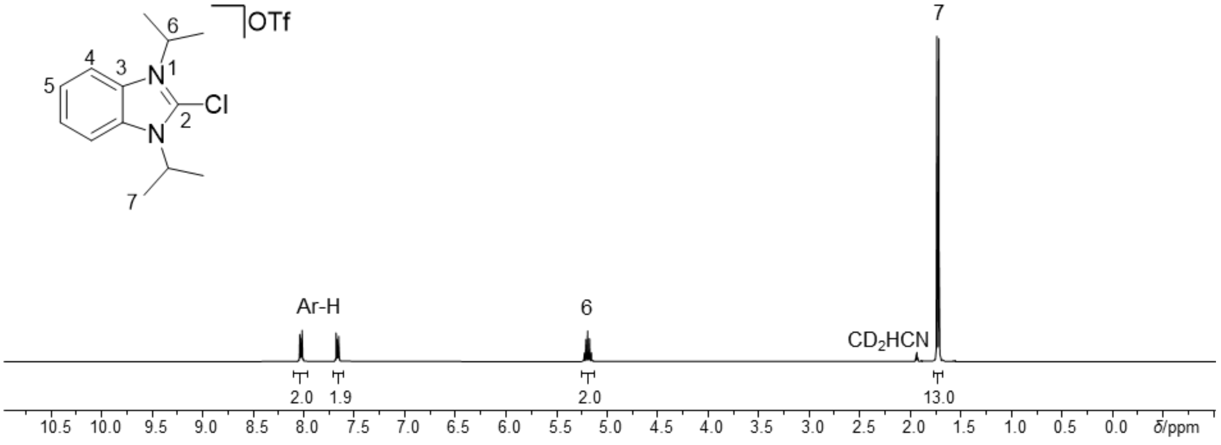

Figure S49. ^{1}H NMR spectrum of (**5**-Cl)OTf (400 MHz, CD_3CN).

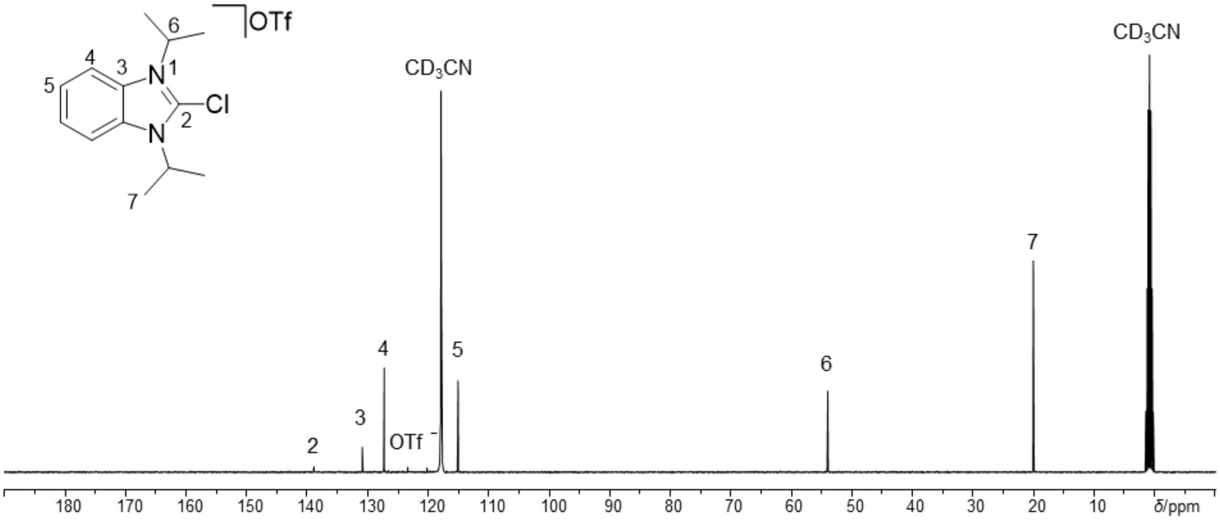

Figure S50. $^{13}C\{^{1}H\}$ NMR spectrum of (**5**-Cl)OTf (101 MHz, CD_3CN).

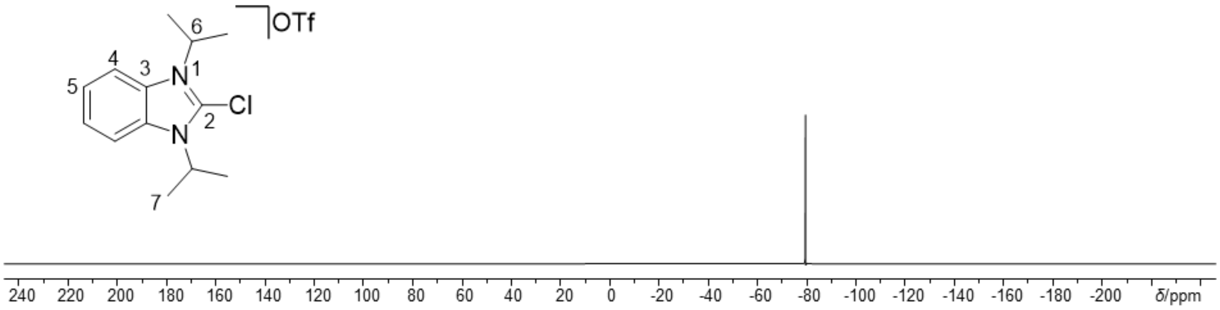

Figure S51. ^{19}F NMR spectrum of (**5**-Cl)OTf (376 MHz, CD_3CN).

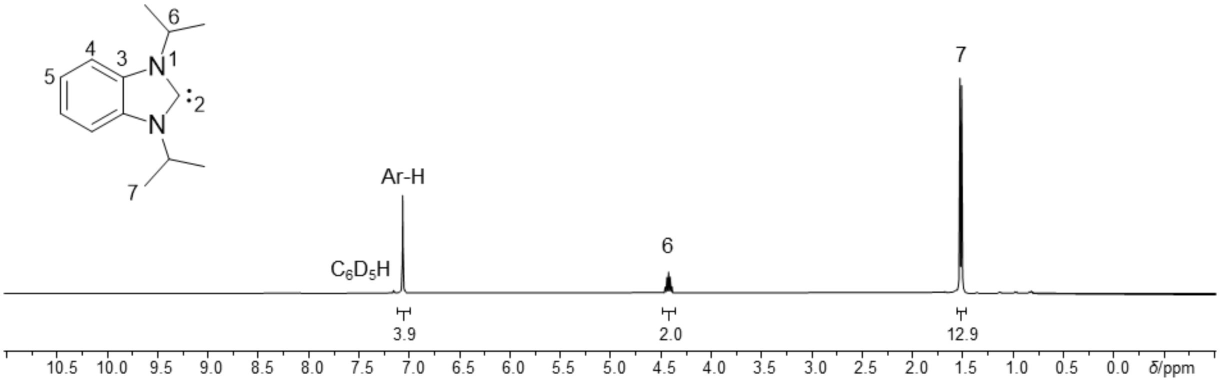

Figure S52. ^{1}H NMR spectrum of NHC **5** (400 MHz, C_6D_6).

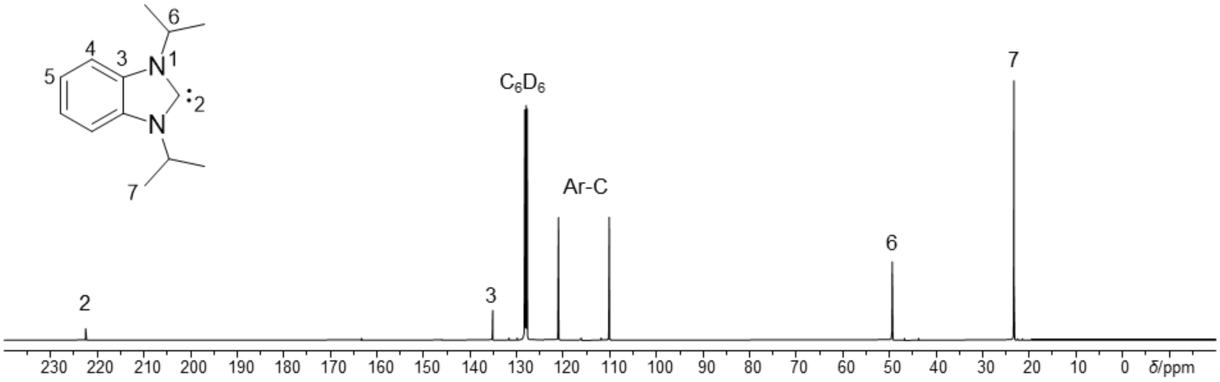

Figure S53. $^{13}C\{^{1}H\}$ NMR spectrum of NHC **5** (101 MHz, C_6D_6).

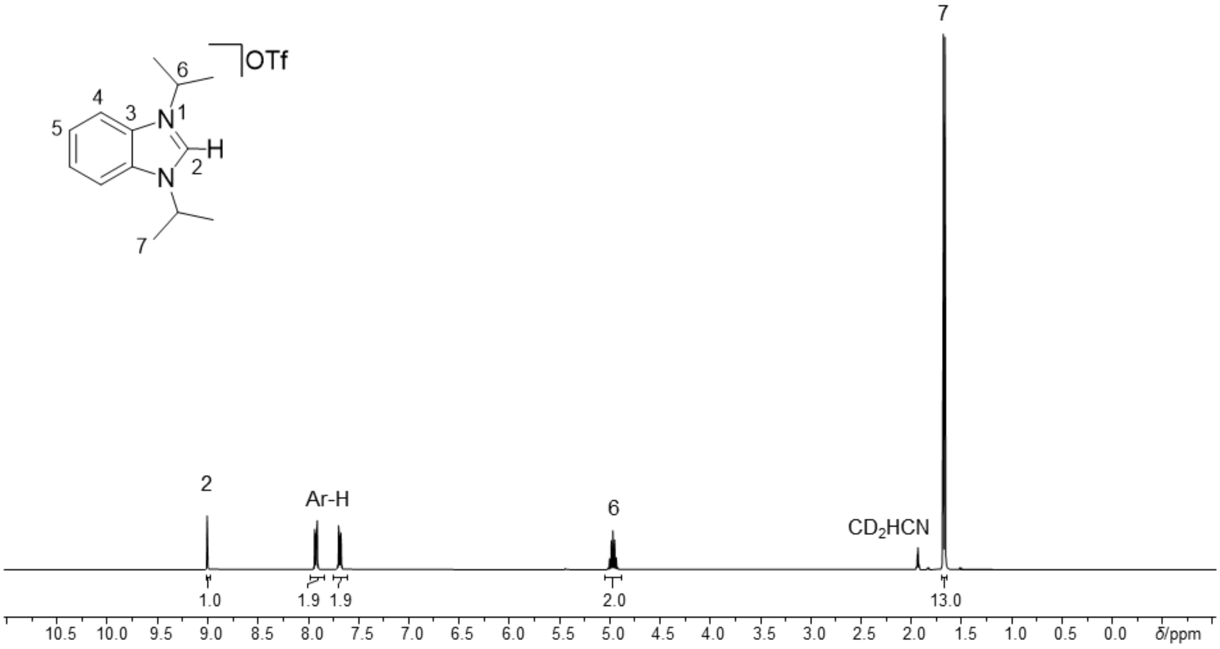

Figure S54. ^{1}H NMR spectrum of (**5**-H)OTf (400 MHz, CD_3CN).

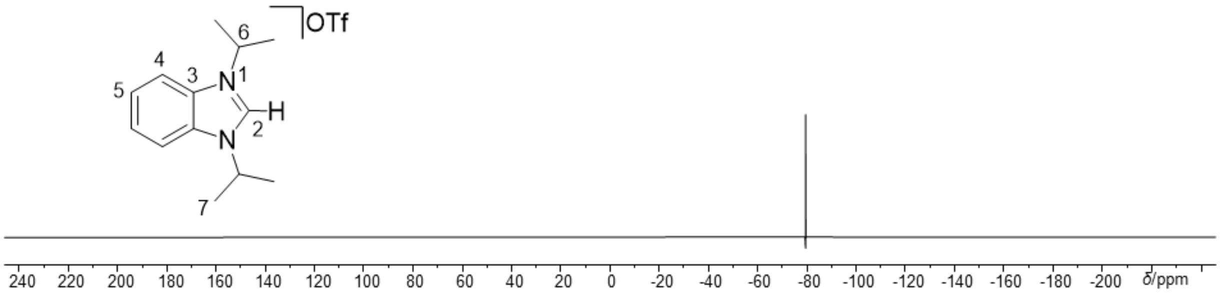

Figure S55. ^{19}F NMR spectrum of (**5**-H)OTf (376 MHz, CD_3CN).

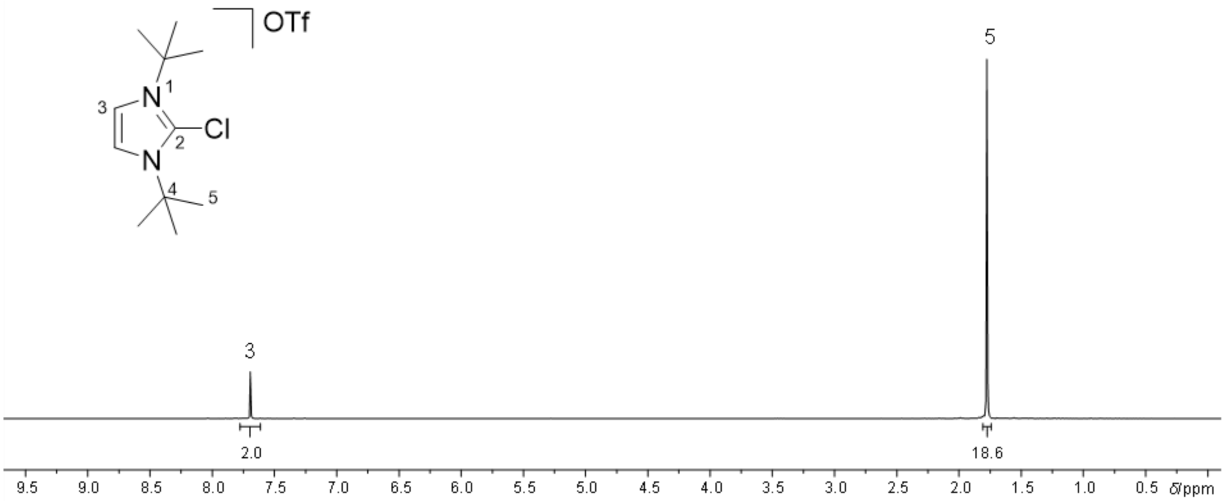

Figure S56. ^{1}H NMR spectrum of (**7**-Cl)OTf (300 MHz, $CDCl_3$).

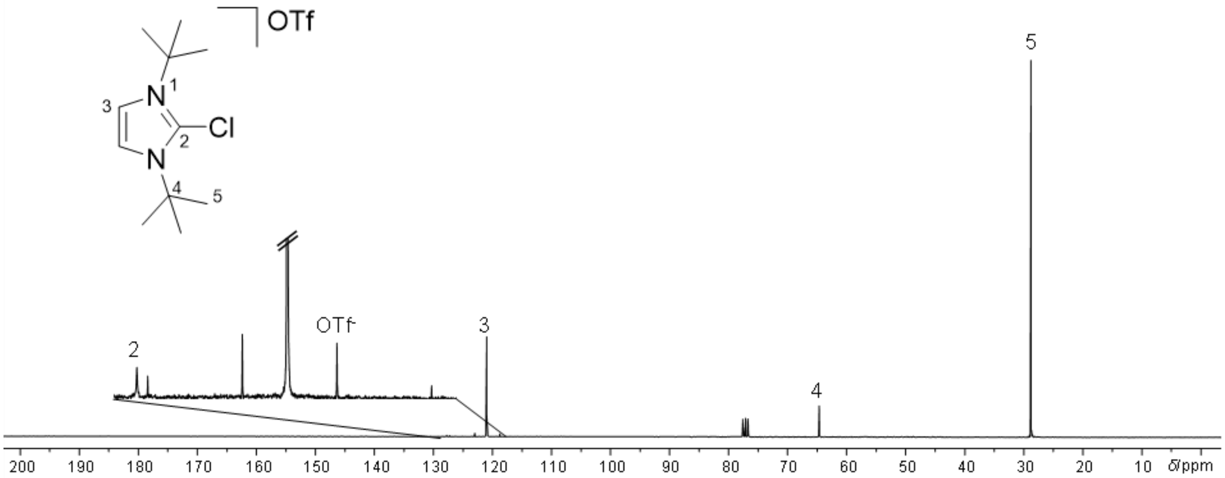

Figure S57. $^{13}C\{^{1}H\}$ NMR spectrum of (**7**-Cl)OTf (75 MHz, $CDCl_3$).

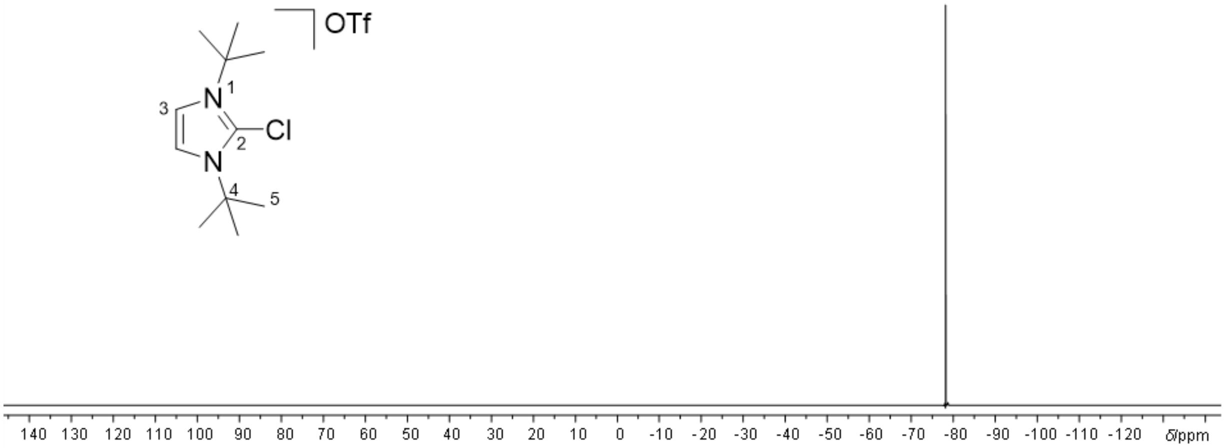

Figure S58. ^{19}F NMR spectrum of (**7**-Cl)OTf (376 MHz, $CDCl_3$).

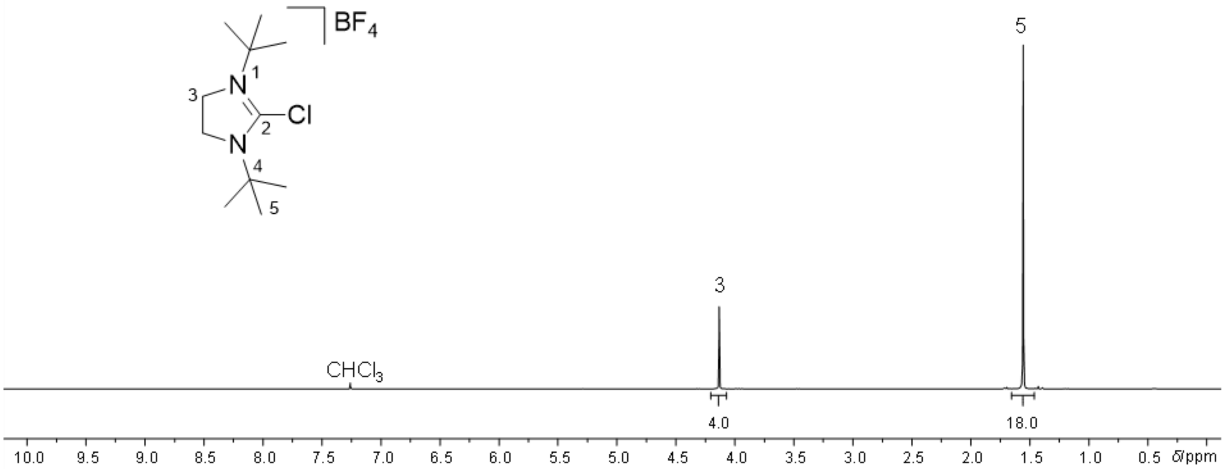

Figure S59. ^{1}H NMR spectrum of (**8**-Cl)BF_4 (400 MHz, $CDCl_3$).

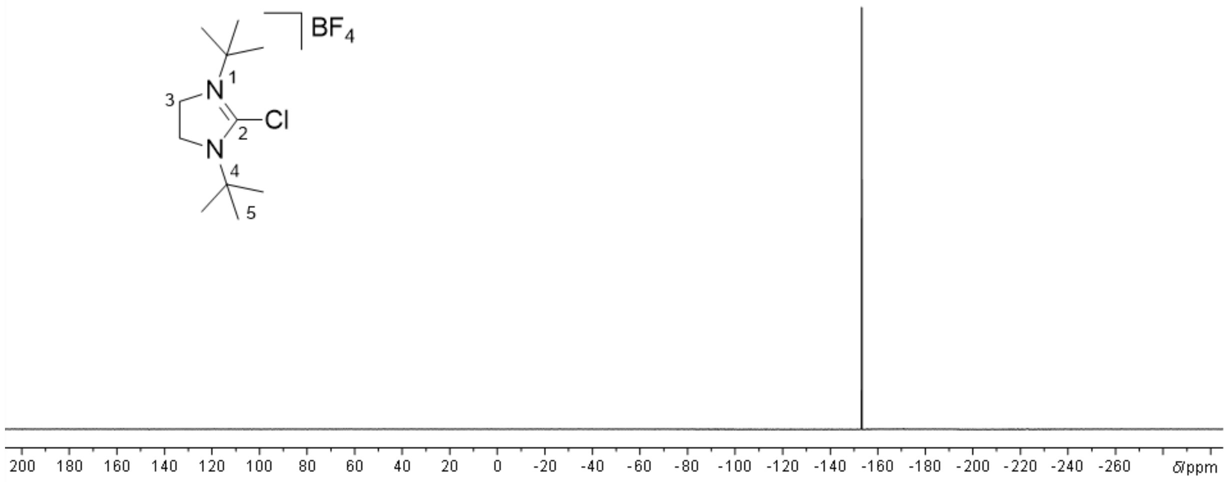

Figure S60. ^{19}F NMR spectrum of (**8**-Cl)BF_4 (471 MHz, $CDCl_3$).

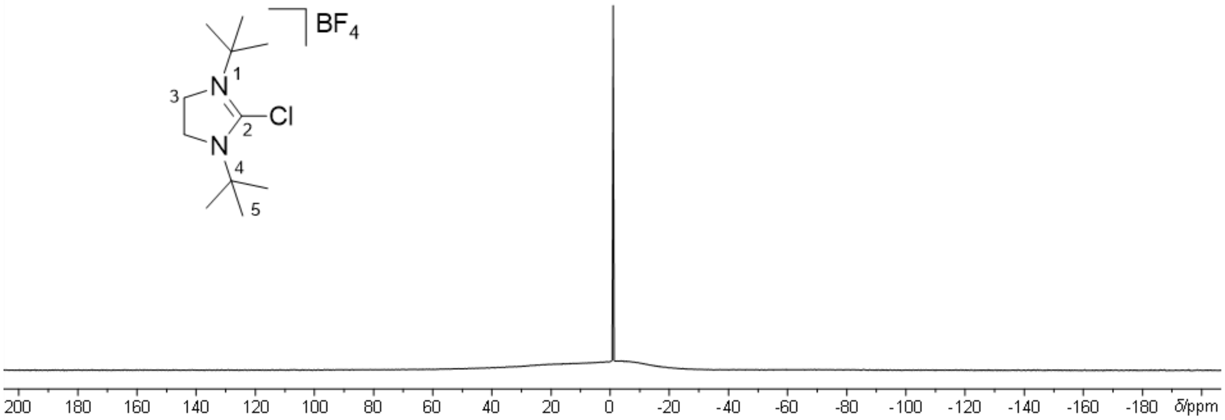

Figure S61. ^{11}B NMR spectrum of (**8**-Cl)BF_4 (160 MHz, $CDCl_3$).

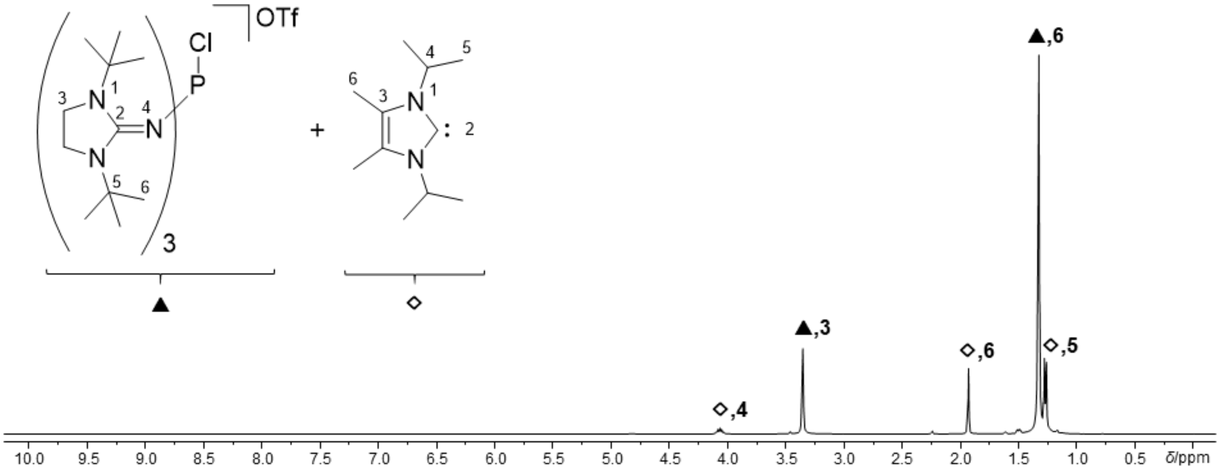

Figure S62. ^{1}H NMR spectrum of the reaction mixture IAP **1** + (**3**-Cl)OTf to give (**1**-Cl)OTf + **3** (400 MHz, THF-d_8).

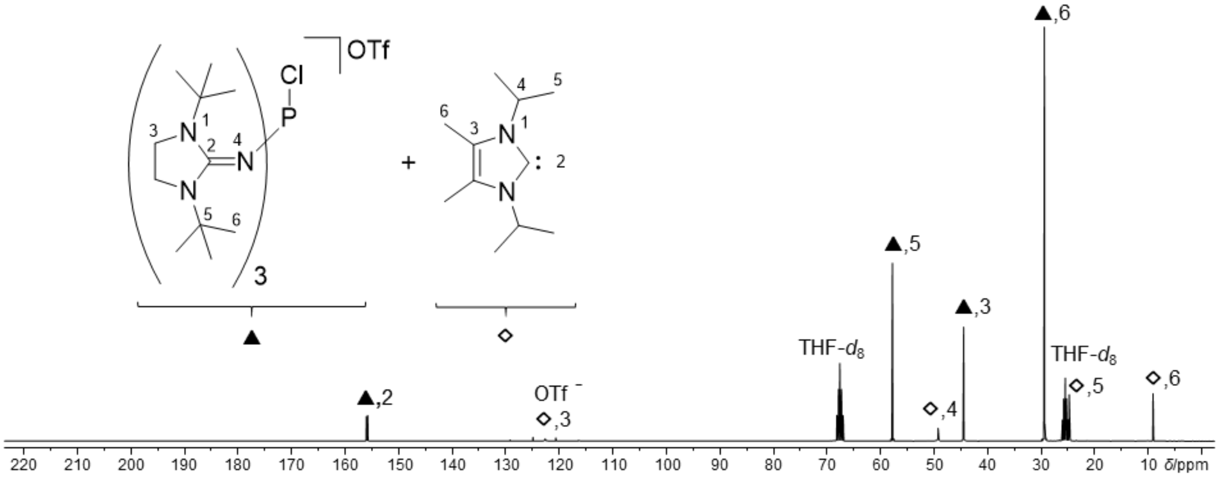

Figure S63. ^{13}C{^{1}H} NMR spectrum of the reaction mixture IAP **1** + (**3**-Cl)OTf to give (**1**-Cl)OTf + **3** (101 MHz, THF-d_8). The resonance for the carbene carbon atom C2 of NHC **3** was not detected. It was detected for **3** isolated from the mixture(**1**-Cl)OTf + **3** (Figure S71).

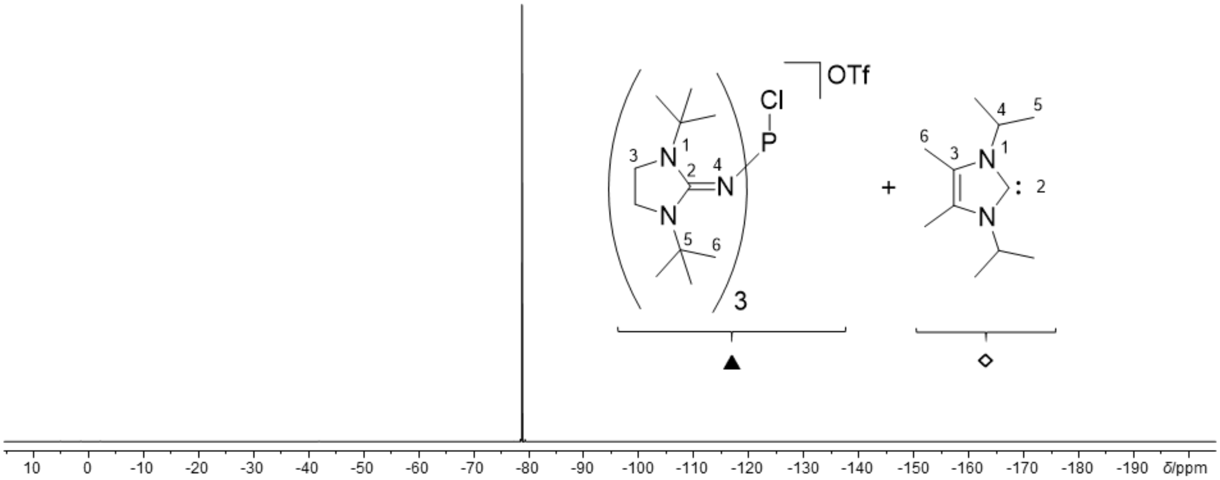

Figure S64. ^{19}F NMR spectrum of the reaction mixture **1** + (**3**-Cl)OTf to give (**1**-Cl)OTf + **3** (376 MHz, THF-d_8).

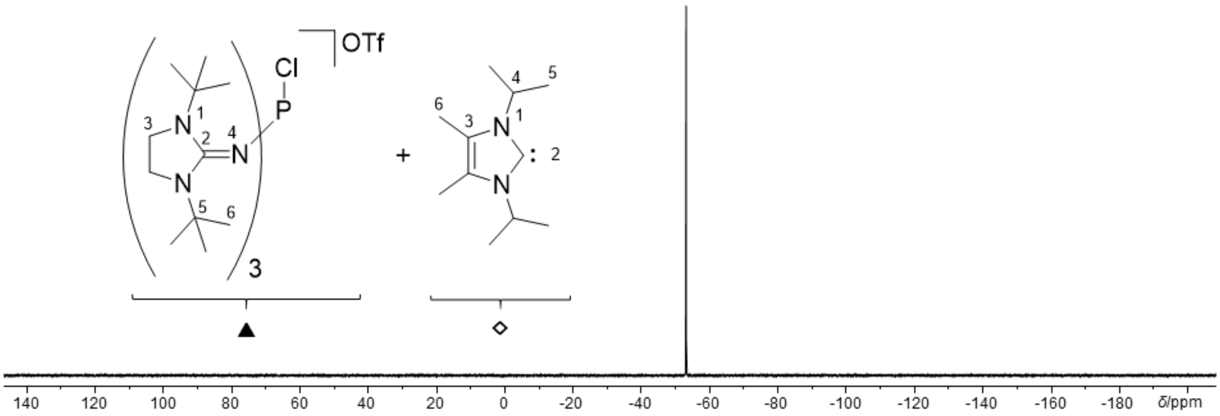

Figure S65. ^{31}P NMR spectrum of the reaction mixture **1** + (**3**-Cl)OTf to give (**1**-Cl)OTf + **3** (162 MHz, THF-d_8).

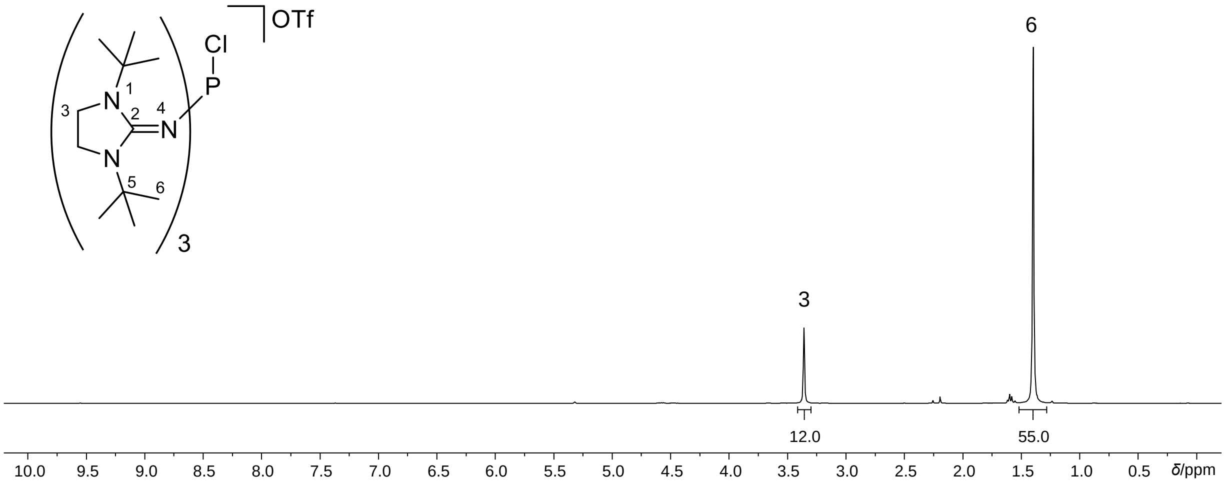

Figure S66. ^{1}H NMR spectrum of (**1**-Cl)OTf isolated from the reaction **1** + (**3**-Cl)OTf (400 MHz, CD_2Cl_2).

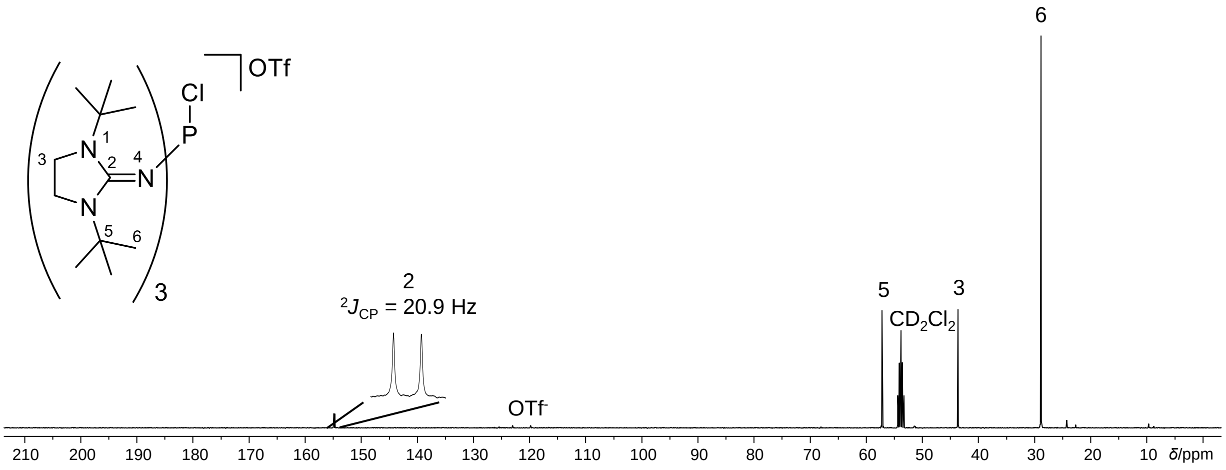

Figure S67. ^{13}C{^{1}H} NMR spectrum of (**1**-Cl)OTf isolated from the reaction **1** + (**3**-Cl)OTf (101 MHz, CD_2Cl_2).

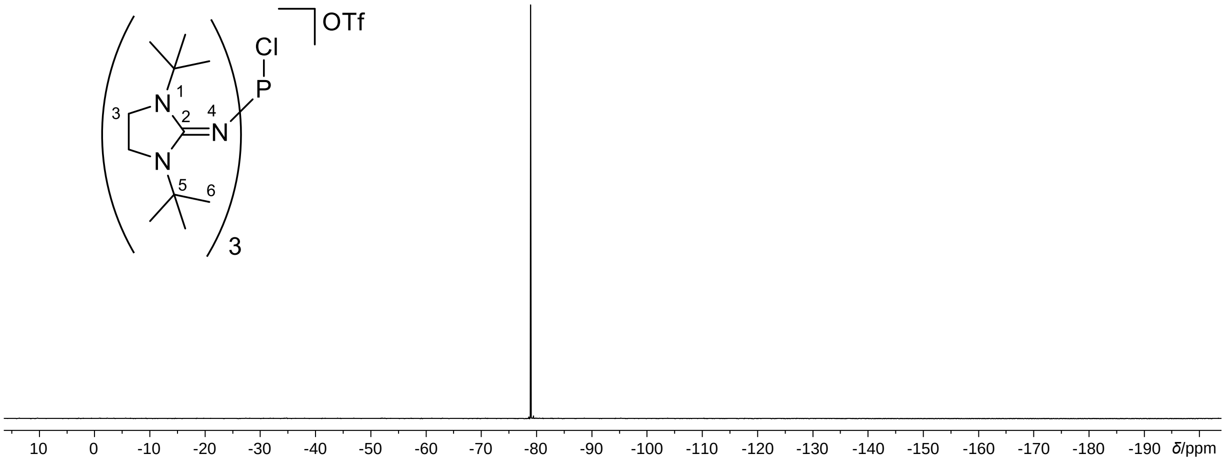

Figure S68. ^{19}F NMR spectrum of (**1**-Cl)OTf isolated from the reaction **1** + (**3**-Cl)OTf (376 MHz, CD_2Cl_2).

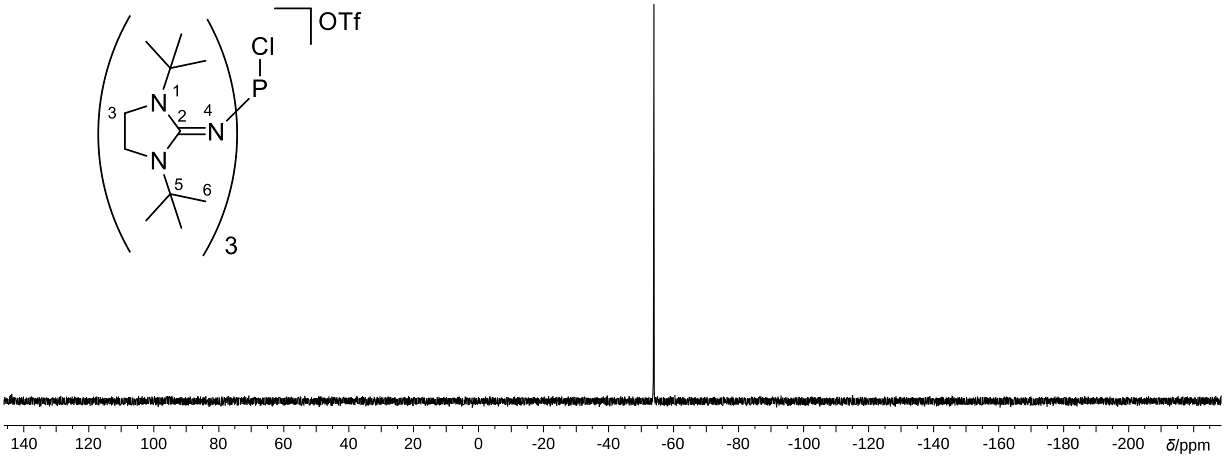

Figure S69. ^{31}P NMR spectrum of (**1**-Cl)OTf isolated from the mixture **1** + (**3**-Cl)OTf (162 MHz, CD_2Cl_2).

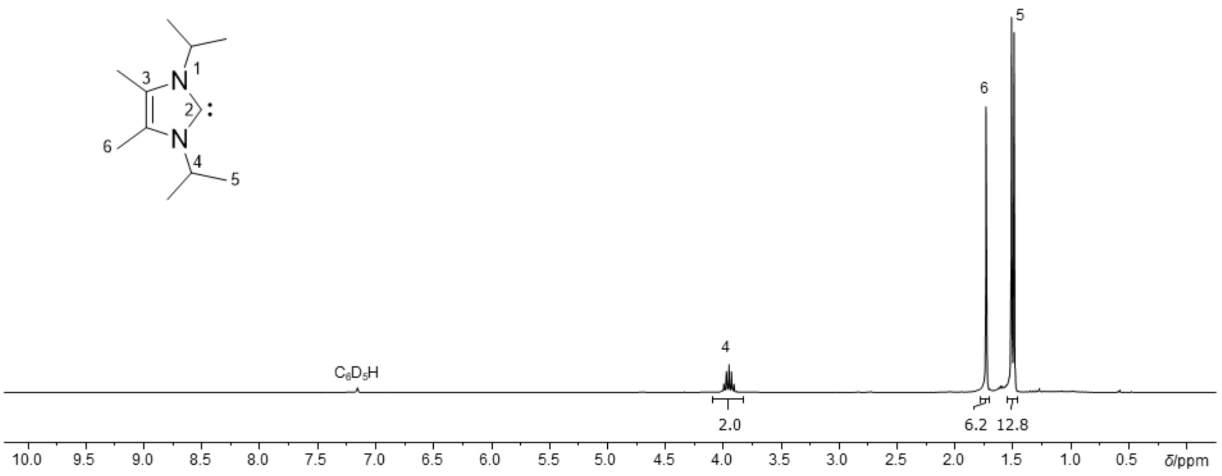

Figure S70. ^{1}H NMR spectrum of the *n*-hexane extract of the reaction **1** + (**3**-Cl)OTf containing NHC **3** (400 MHz, C_6D_6).

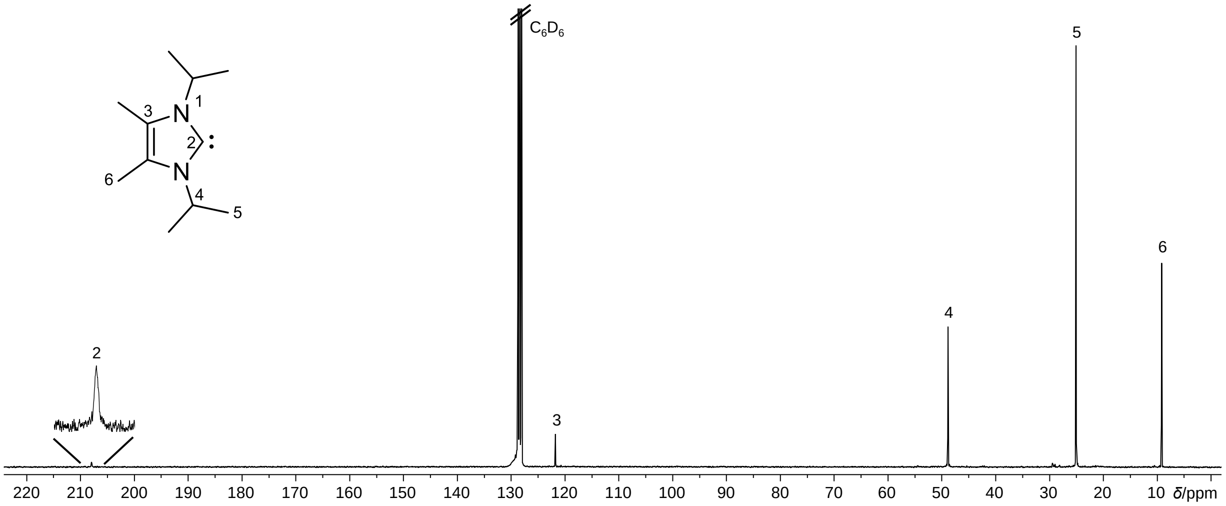

Figure S71. ^{13}C{^{1}H} NMR spectrum of the *n*-hexane extract of the reaction **1** + (**3**-Cl)OTf containing NHC **3** (101 MHz, C_6D_6).

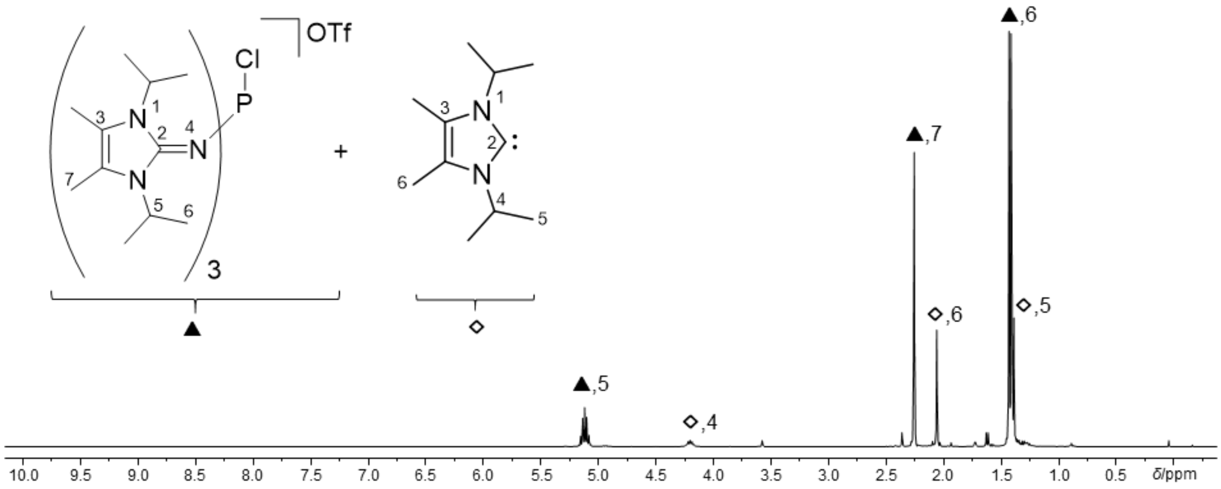

Figure S72. ^{1}H NMR spectrum of the reaction mixture IAP **2** + (**3**-Cl)OTf to give (**2**-Cl)OTf + **3** (400 MHz, THF-d_8).

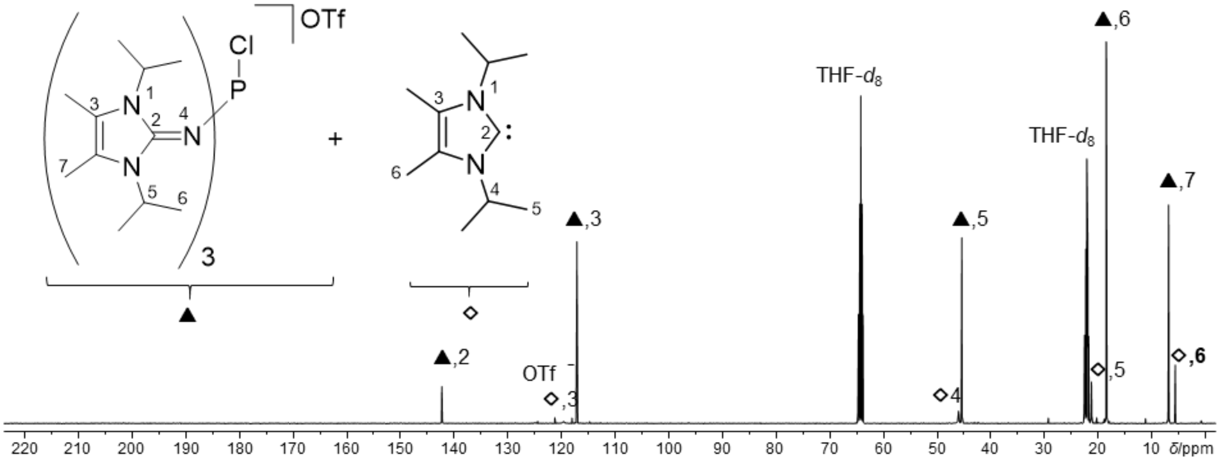

Figure S73. ^{13}C{^{1}H} NMR spectrum of the reaction mixture IAP **2** + (**3**-Cl)OTf to give (**2**-Cl)OTf + **3** (101 MHz, THF-d_8). The resonance for the carbene carbon atom C2 of NHC **3** was not detected. It was detected for **3** isolated from the mixture (Figure S81).

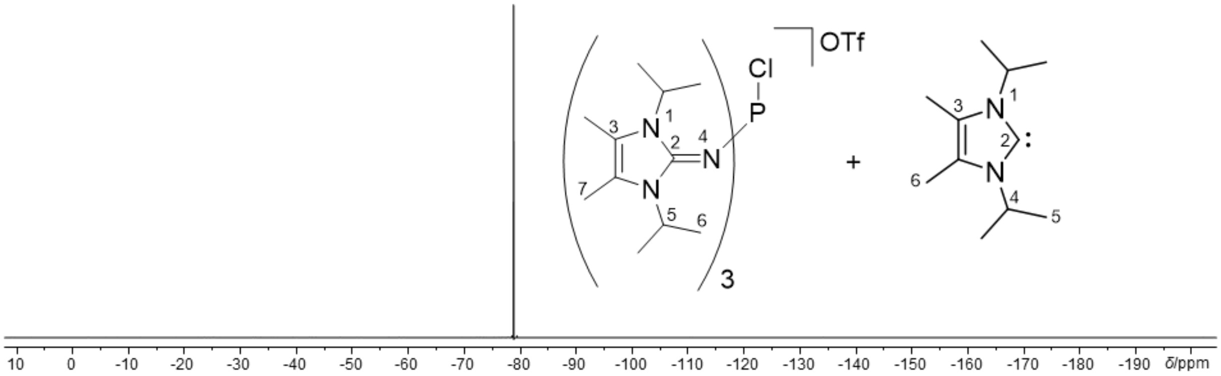

Figure S74. ^{19}F NMR spectrum of the reaction mixture reaction mixture IAP **2** + (**3**-Cl)OTf to give (**2**-Cl)OTf + **3** (376 MHz, THF-d_8).

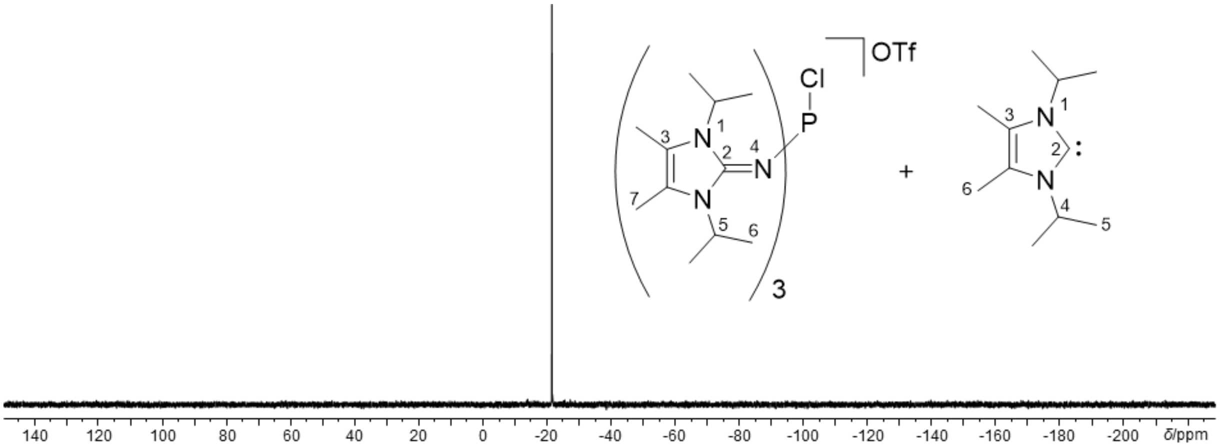

Figure S75. ^{31}P NMR spectrum of the reaction mixture reaction mixture IAP **2** + (**3**-Cl)OTf to give (**2**-Cl)OTf + **3** (162 MHz, THF-d_8).

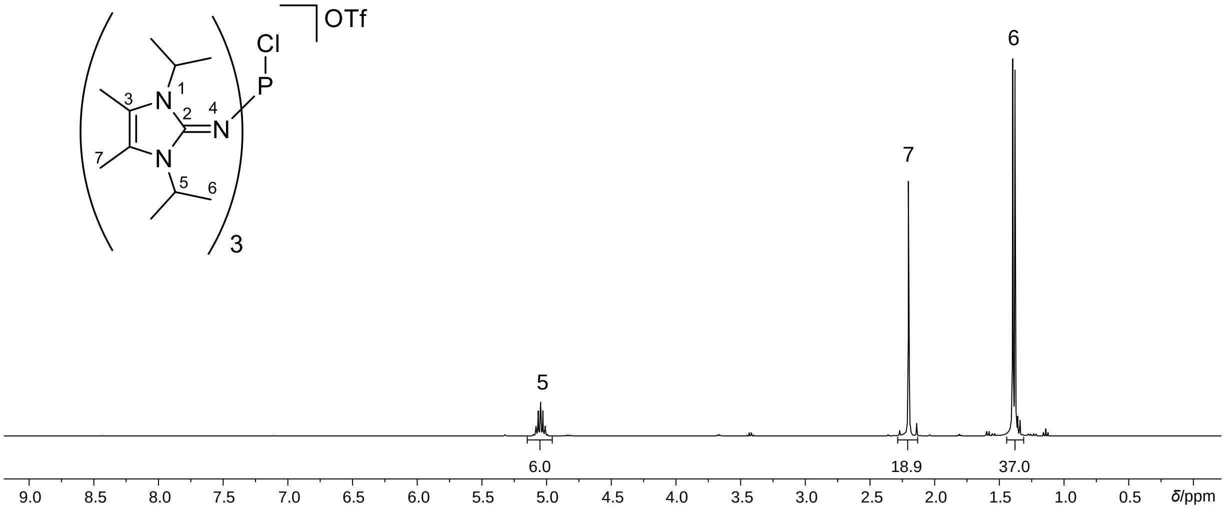

Figure S76. ^{1}H NMR spectrum of (**2**-Cl)OTf isolated from the mixture **2** + (**3**-Cl)OTf (400 MHz, CD_2Cl_2).

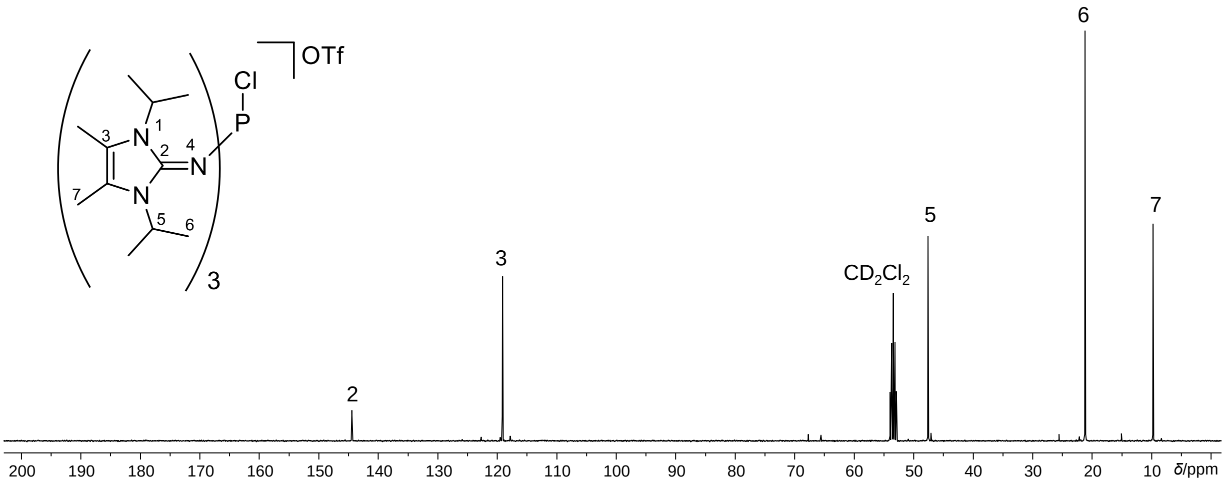

Figure S77. $^{13}C\{^{1}H\}$ NMR spectrum of (**2**-Cl)OTf isolated from the mixture **2** + (**3**-Cl)OTf (101 MHz, CD_2Cl_2).

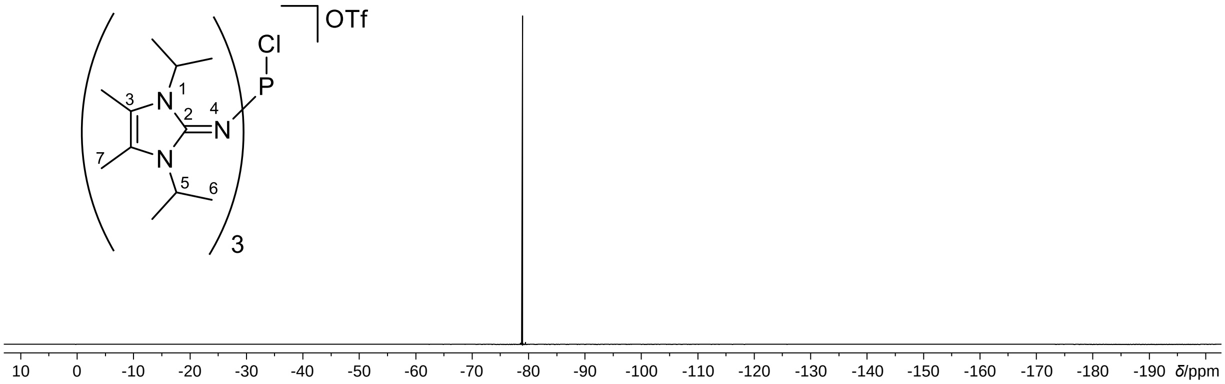

Figure S78. ^{19}F NMR spectrum of (**2**-Cl)OTf isolated from the mixture **2** + (**3**-Cl)OTf (376 MHz, CD_2Cl_2).

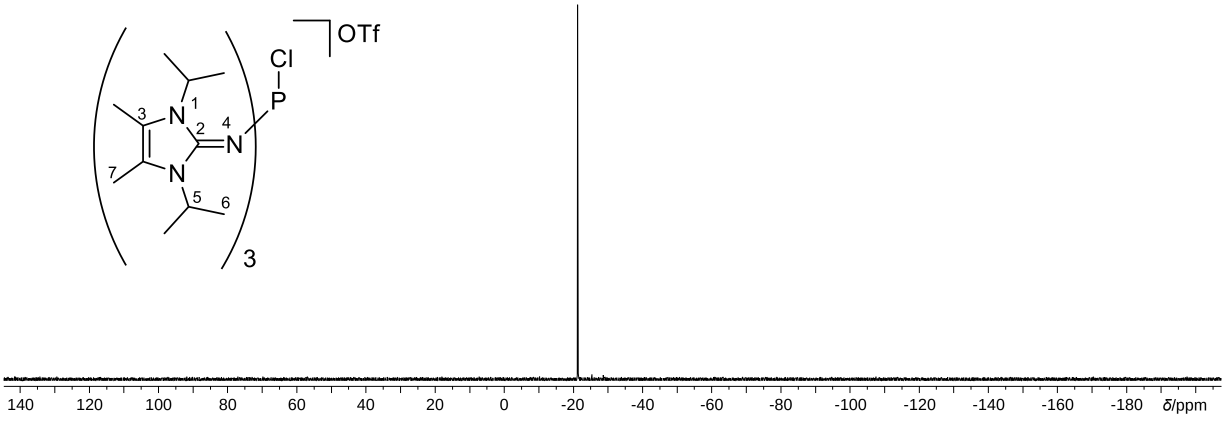

Figure S79. ^{31}P NMR spectrum of (**2**-Cl)OTf isolated from the mixture **2** + (**3**-Cl)OTf (162 MHz, CD_2Cl_2).

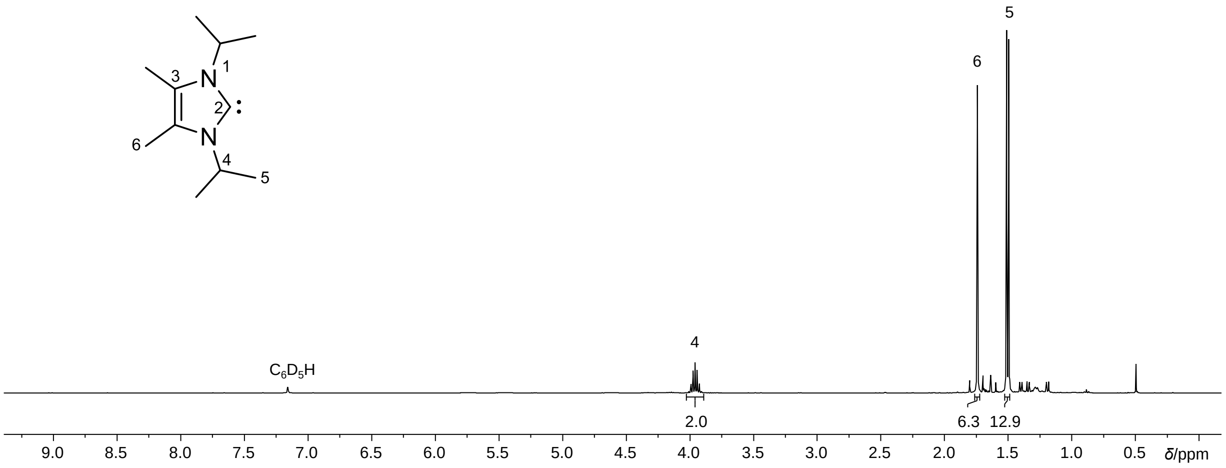

Figure S80. ^{1}H NMR spectrum of the *n*-hexane extract of the reaction **2** + (**3**-Cl)OTf containing NHC **3** (400 MHz, C_6D_6).

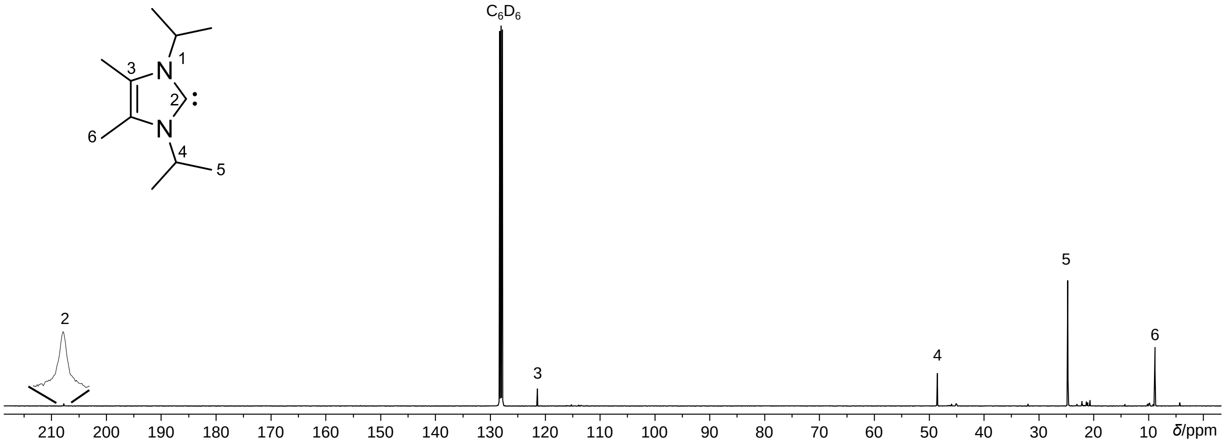

Figure S81. $^{13}C\{^{1}H\}$ NMR spectrum of the *n*-hexane extract of the reaction **2** + (**3**-Cl)OTf containing NHC **3** (101 MHz, C_6D_6).

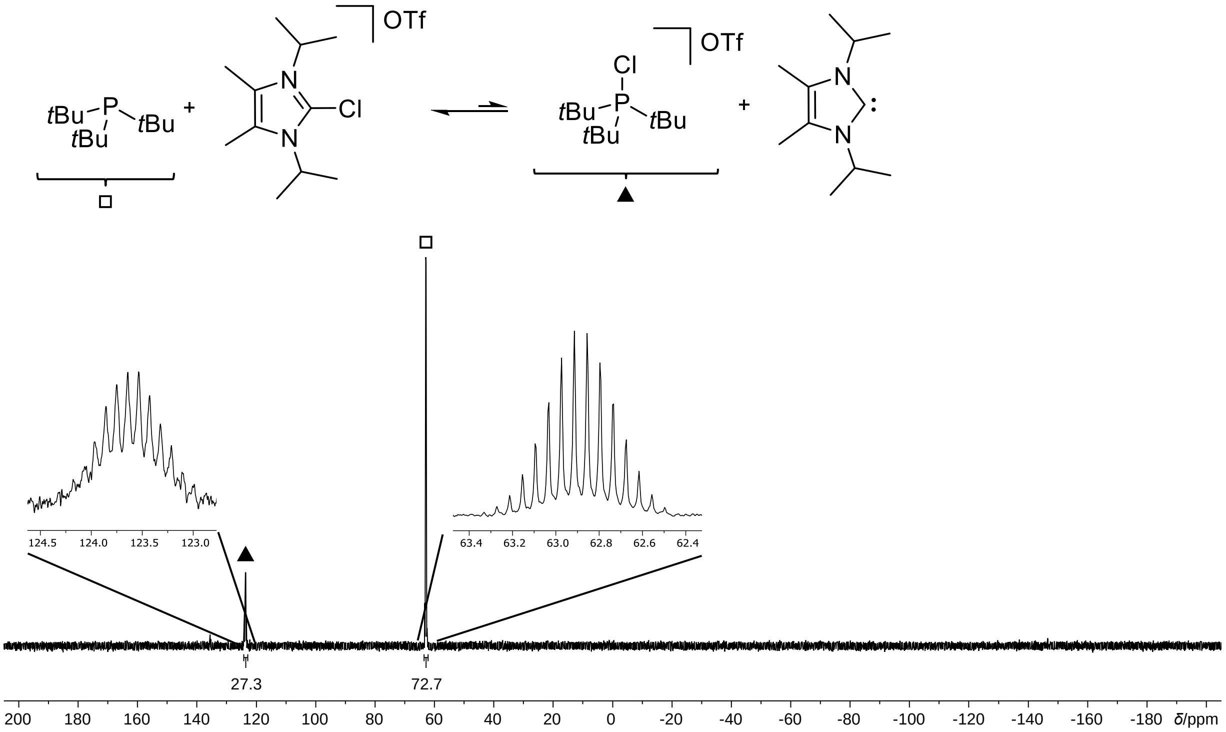

Figure S82. ^{31}P NMR spectrum (D_1 25 sec, acquisition time 0.66 sec, zg30) of the reaction of P*t*Bu$_3$ with (**3**-Cl)OTf after 1 h (162 MHz, THF-*d*$_8$).

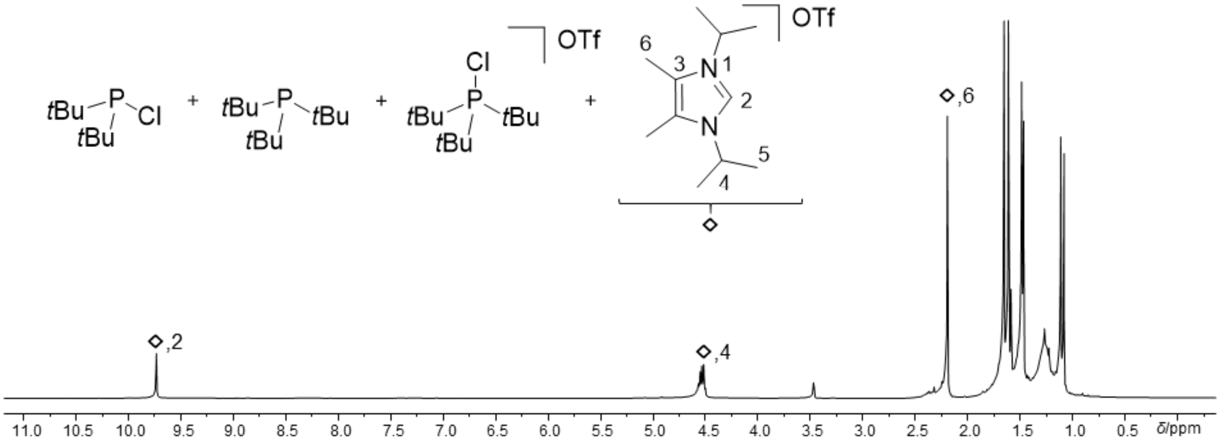

Figure S83. ^{1}H NMR spectrum of the reaction of P*t*Bu$_3$ with (**3**-Cl)OTf after 72 h (400 MHz, THF-*d*$_8$).

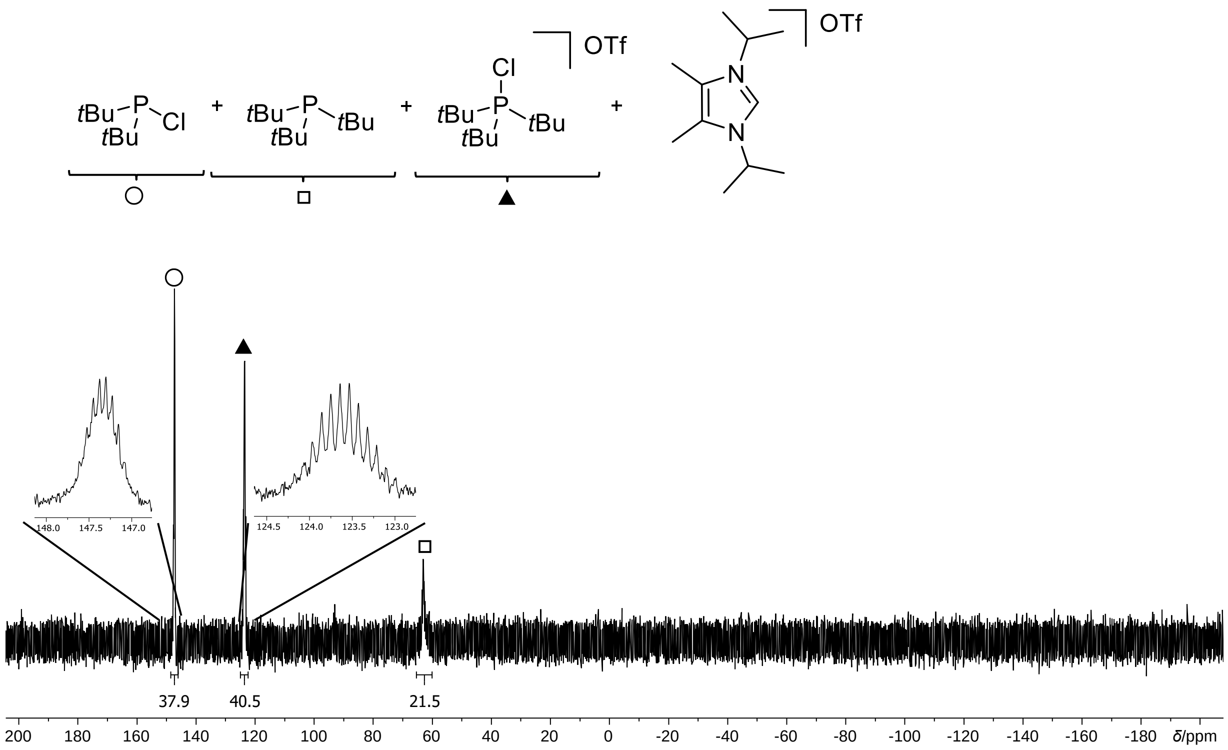

Figure S84. ^{31}P NMR spectrum (D_1 25 sec, acquisition time 0.66 sec, zg30) of the reaction of P*t*Bu_3 with (**3**-Cl)OTf after 72 h (162 MHz, THF-d_8).

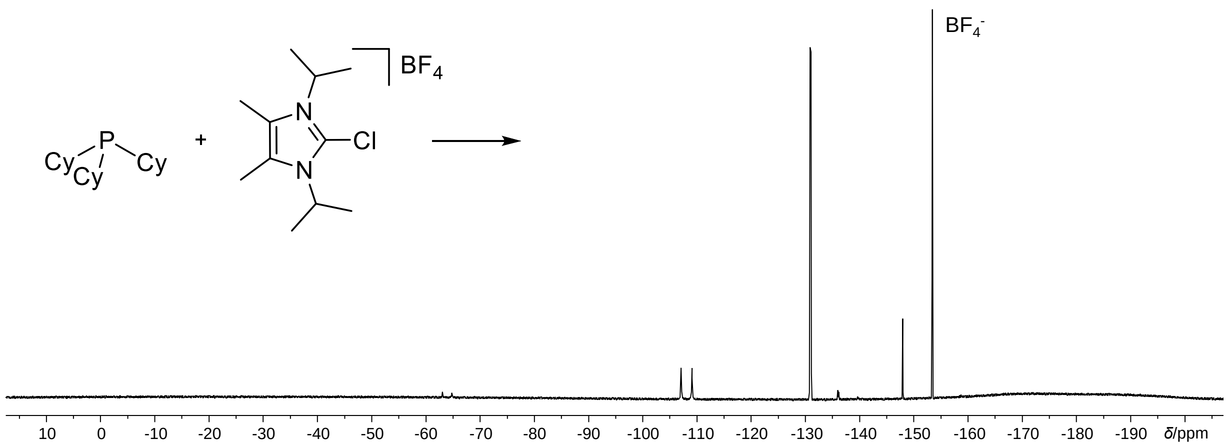

Figure S85. ^{19}F NMR spectrum of the products of the reaction of PCy_3 with (**3**-Cl)BF_4 (376 MHz, THF-d_8).

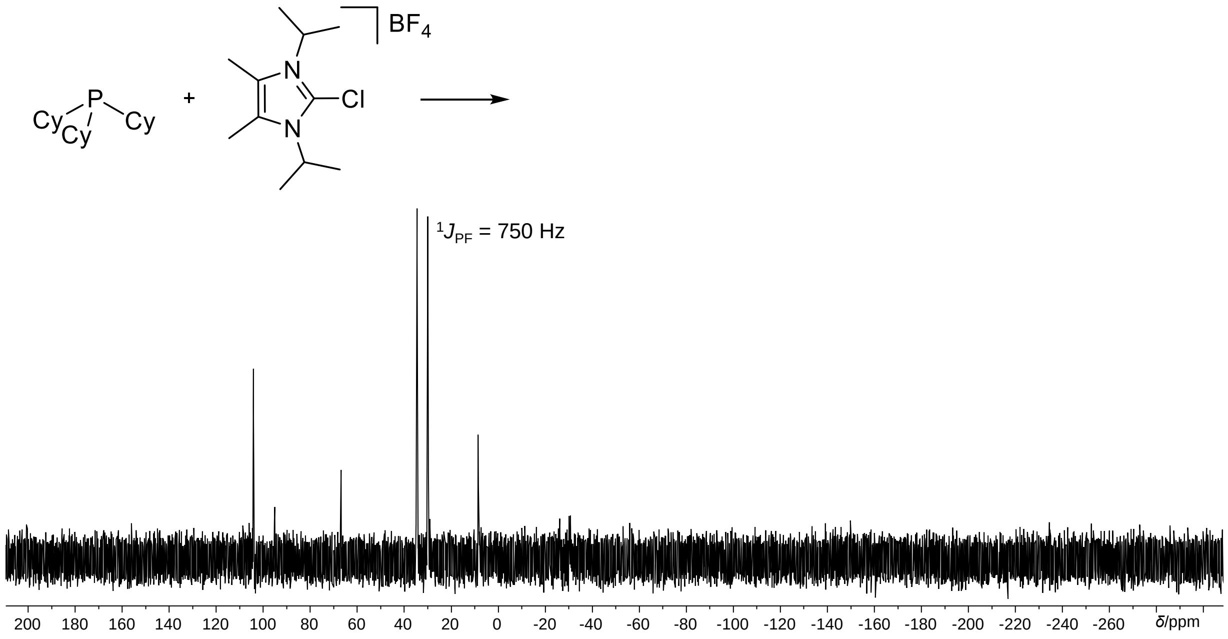

Figure S86. ^{31}P NMR spectrum of the products of the reaction of PCy_3 with (**3**-Cl)BF_4 (162 MHz, THF-d_8).

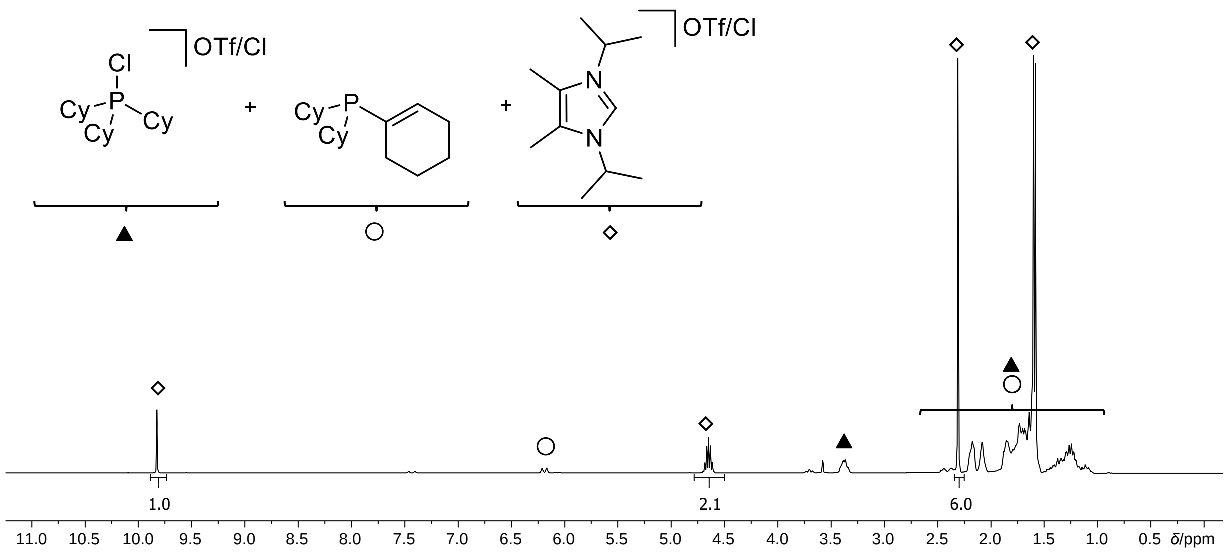

Figure S87. ^{1}H NMR spectrum of the reaction of PCy_3 with (**3**-Cl)OTf after 72 h (400 MHz, THF-d_8).

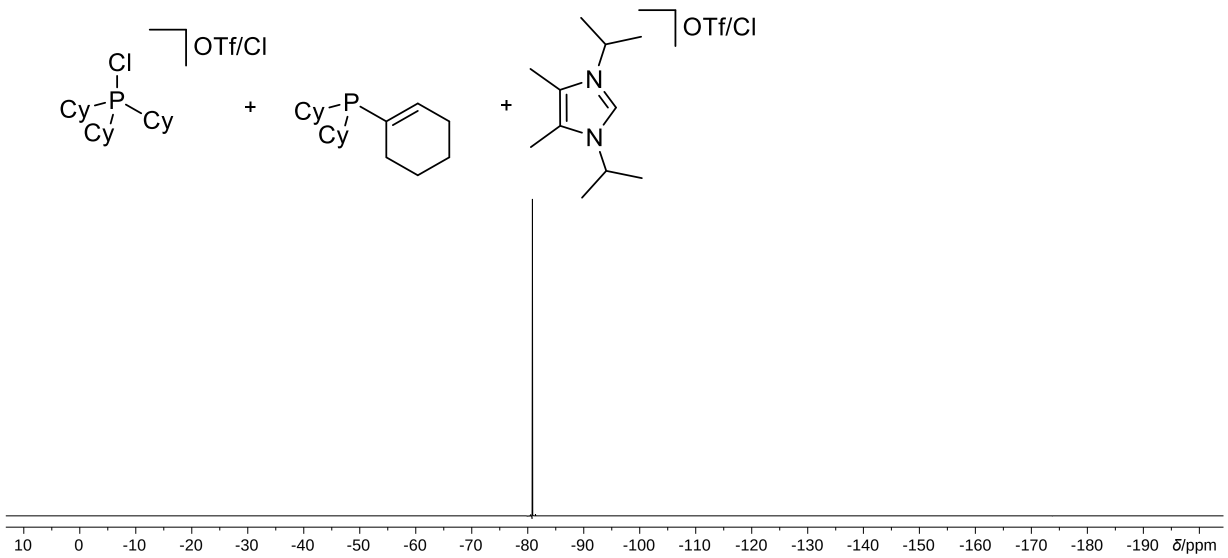

Figure S88. ^{19}F NMR spectrum of the reaction of PCy_3 with (**3**-Cl)OTf after 72 h (376 MHz, THF-d_8).

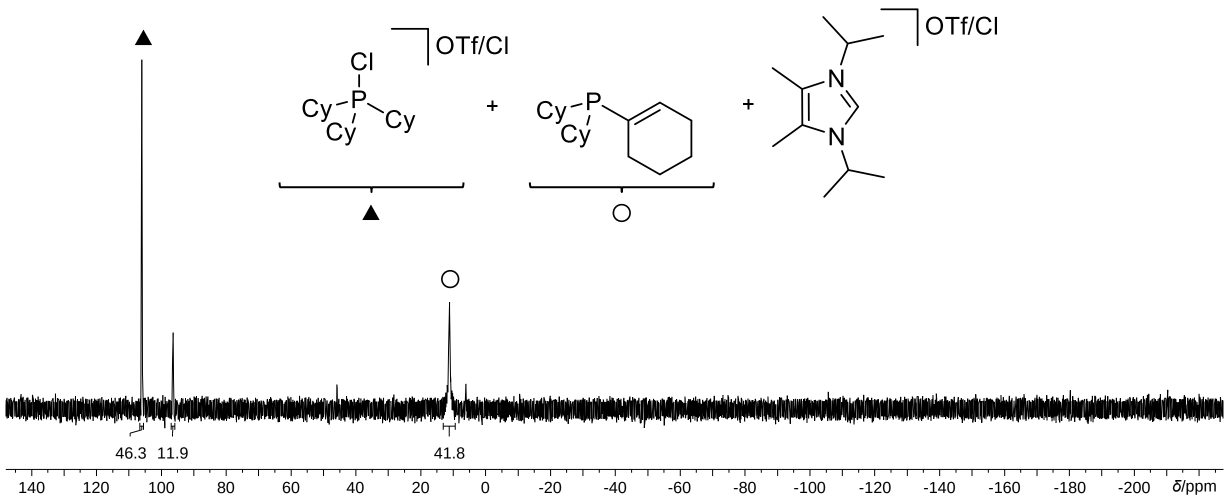

Figure S89. ^{31}P NMR spectrum (D_1 = 25 sec, aq. time = 0.66 sec., zg30) of the reaction of PCy_3 with (**3**-Cl)OTf after 72 h (162 MHz, THF-d_8).

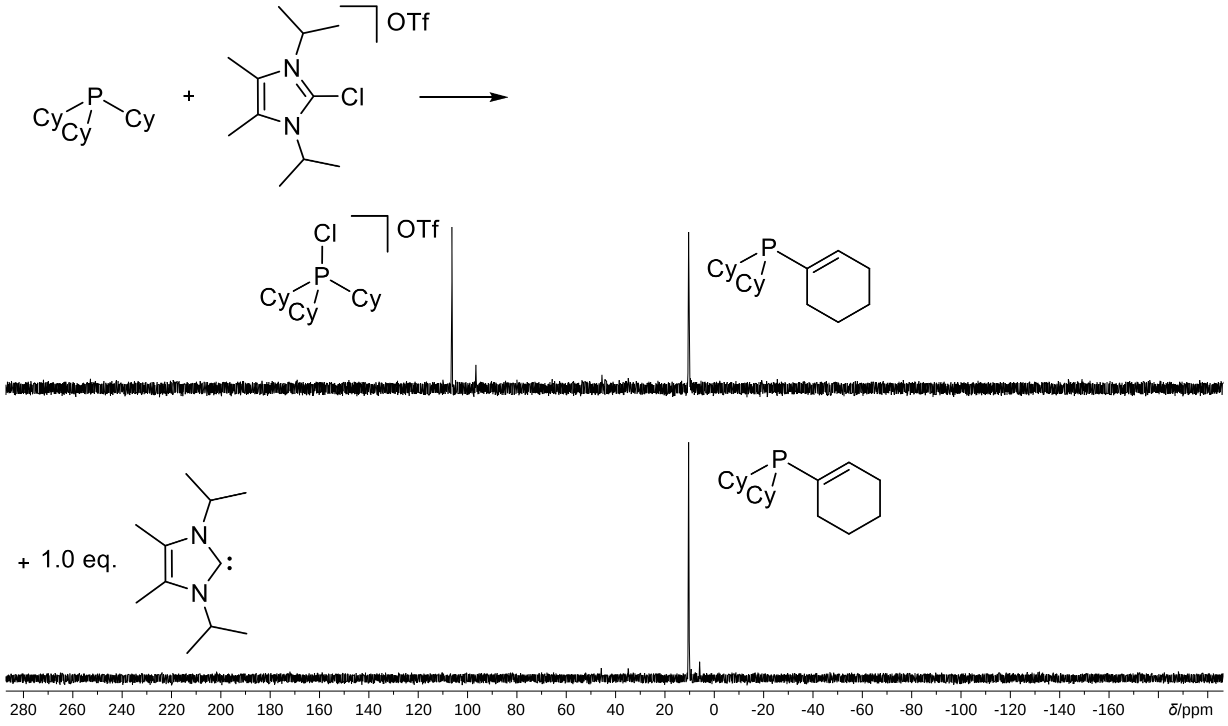

Figure S90. ^{31}P NMR spectra of the reaction mixture of PCy_3 with (**3**-Cl)OTf (top) and after the addition of 1.0 equivalent of the carbene **3** (bottom) showing complete conversion of the chlorophosphonium salt to the vinylenephosphine **4** (162 MHz, THF-d_8).

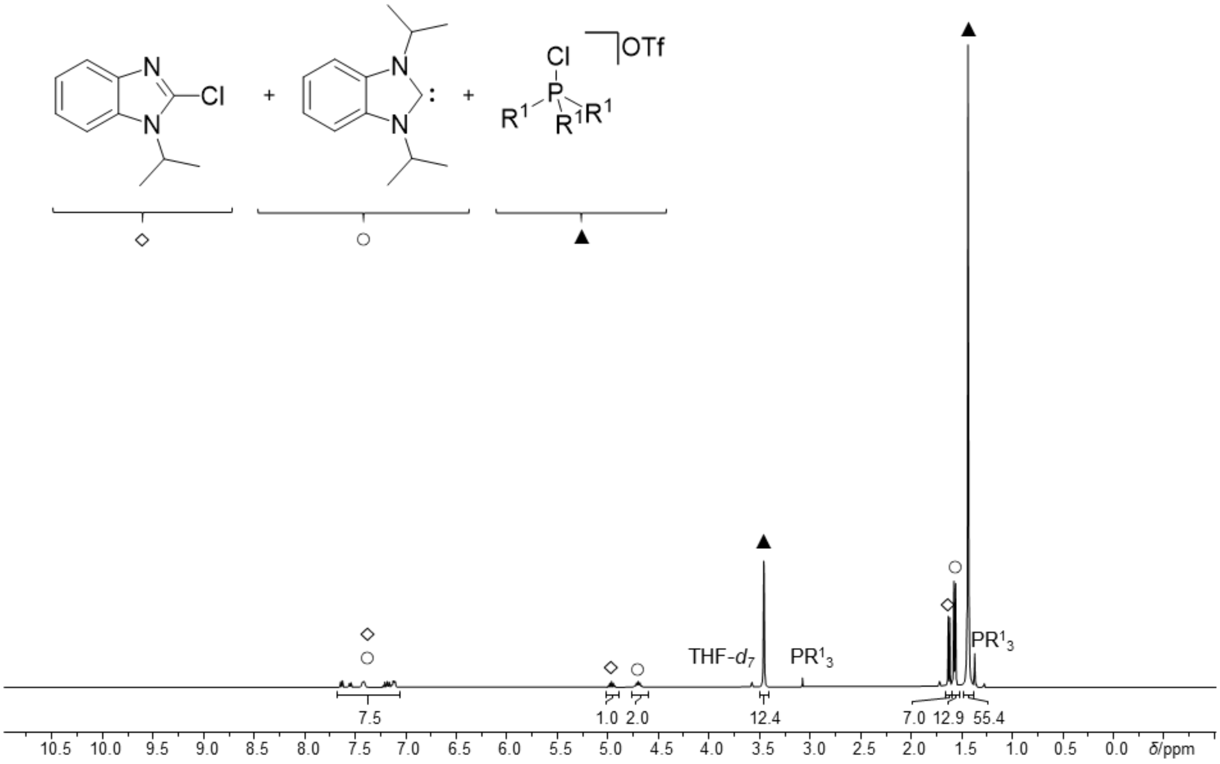

Figure S91. ^{1}H NMR spectrum of the product mixture from the reaction of phosphine **1** with (**5-**Cl)OTf in the presence of **6** (400 MHz, THF-d_8).

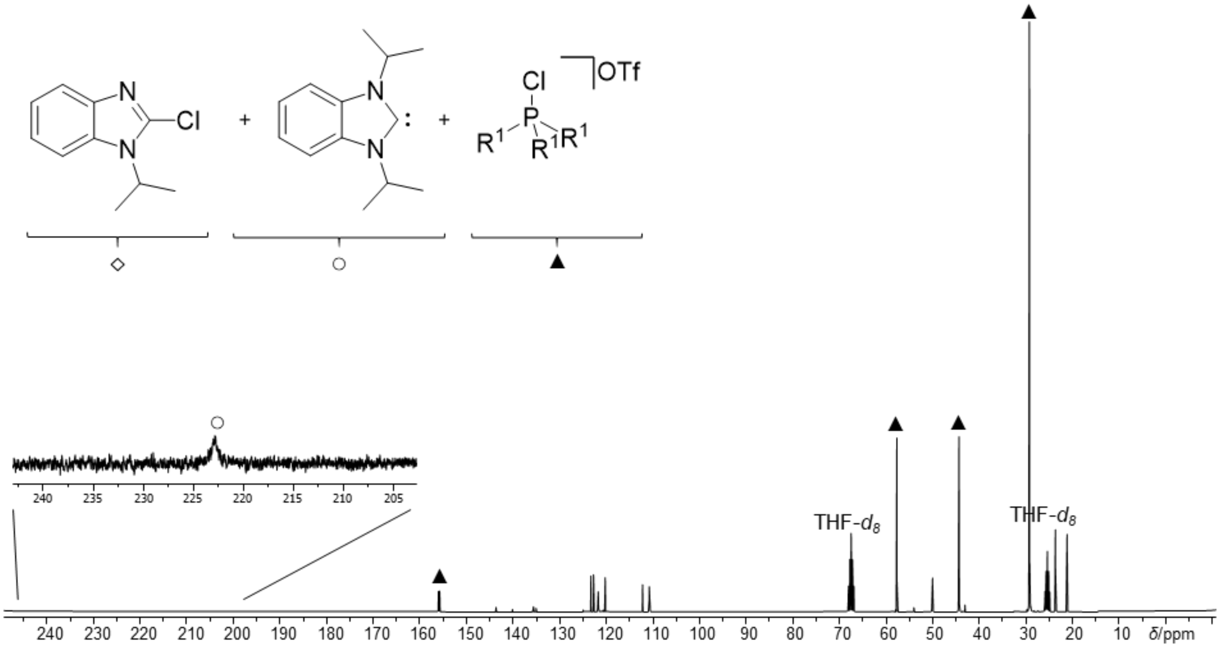

Figure S92. ^{13}C{^{1}H} NMR spectrum of the product mixture from the reaction of phosphine **1** with (**5-**Cl)OTf in the presence of **6** (75 MHz, THF-d_8). Only the characteristic resonances for the carbene carbon atom and the resonances of phosphine **1** are assigned.

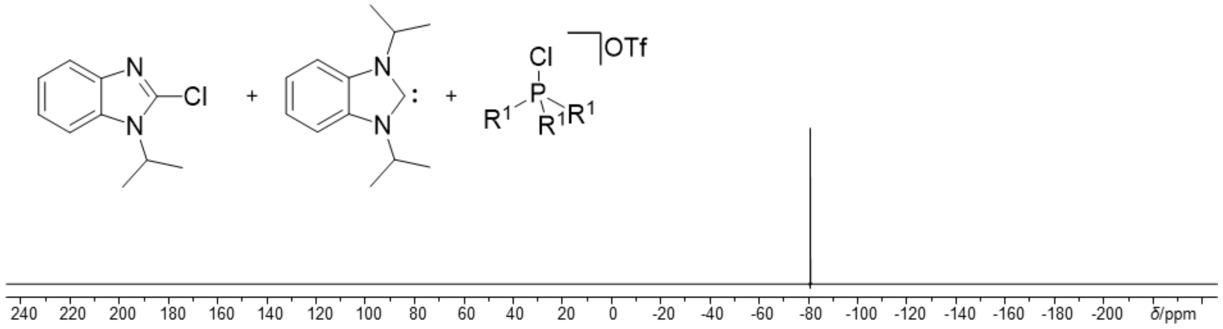

Figure S93. ^{19}F NMR spectrum of the product mixture from the reaction of phosphine **1** with (**5-**Cl)OTf in the presence of **6** (373 MHz, THF-d_8).

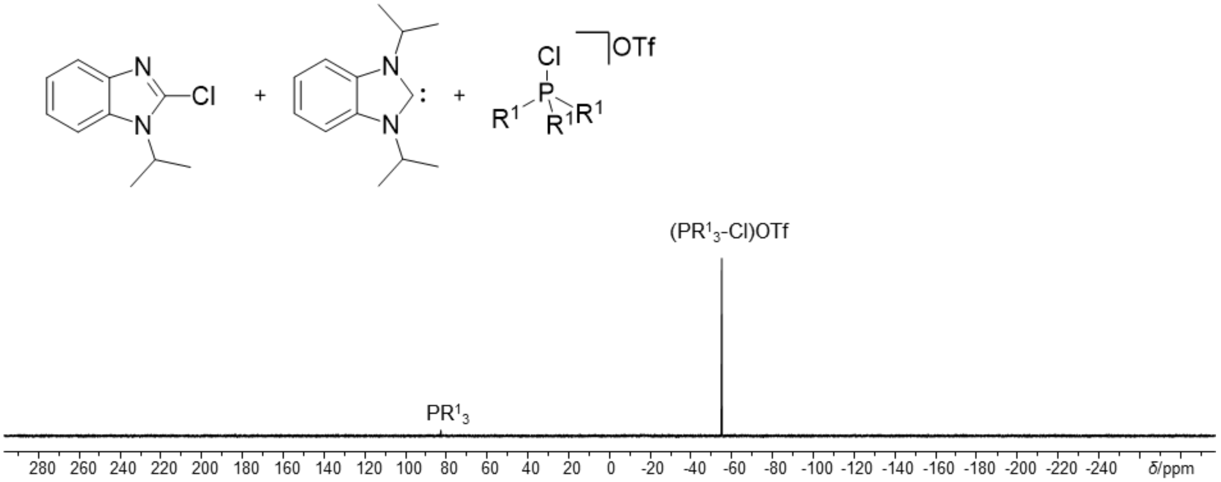

Figure S94. ^{31}P NMR spectrum of the product mixture from the reaction of phosphine **1** with (**5-**Cl)OTf in the presence of **6** (162 MHz, THF-d_8).

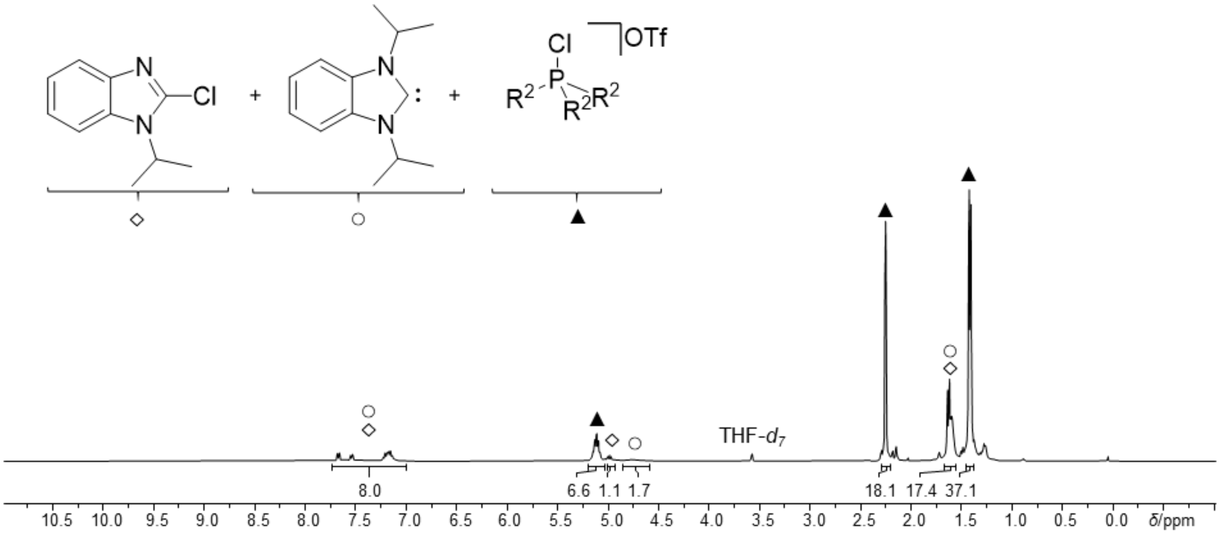

Figure S95. ^{1}H NMR spectrum of the product mixture from the reaction of phosphine **2** with (**5-**Cl)OTf in the presence of **6** (400 MHz, THF-d_8).

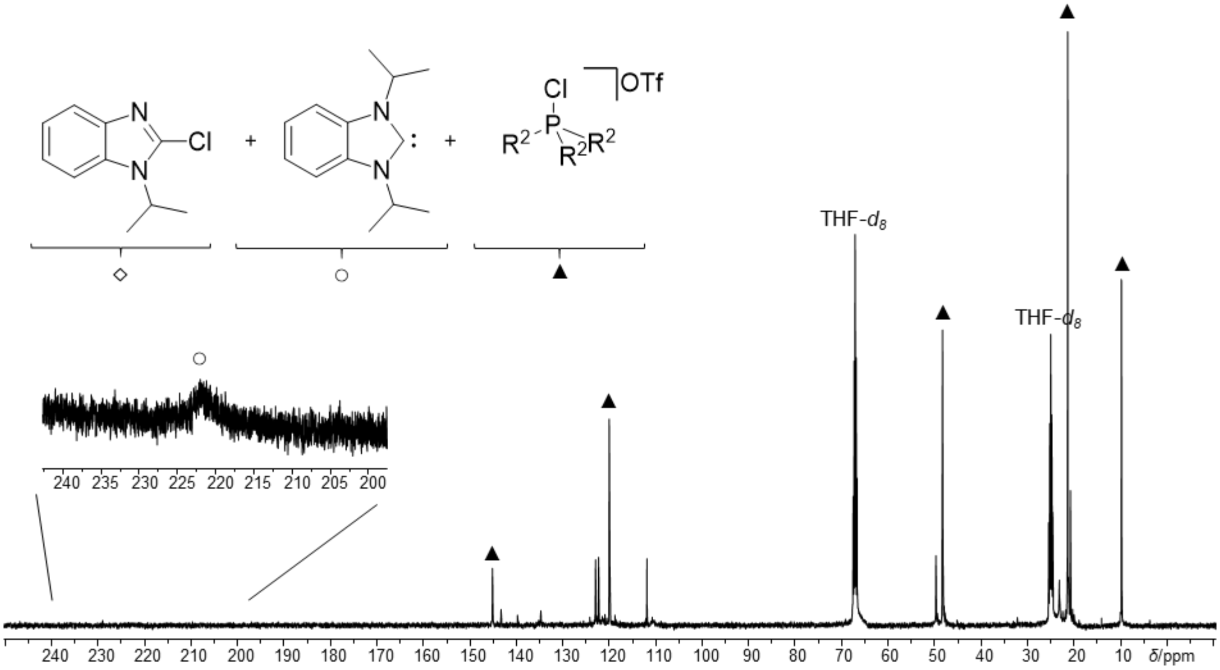

Figure S96. $^{13}C\{^{1}H\}$ NMR spectrum of the product mixture from the reaction of phosphine **2** with (**5-**Cl)OTf in the presence of **6** (75 MHz, THF-d_8). Only the characteristic resonances for the carbene carbon atom and the resonances of phosphine **2** are assigned.

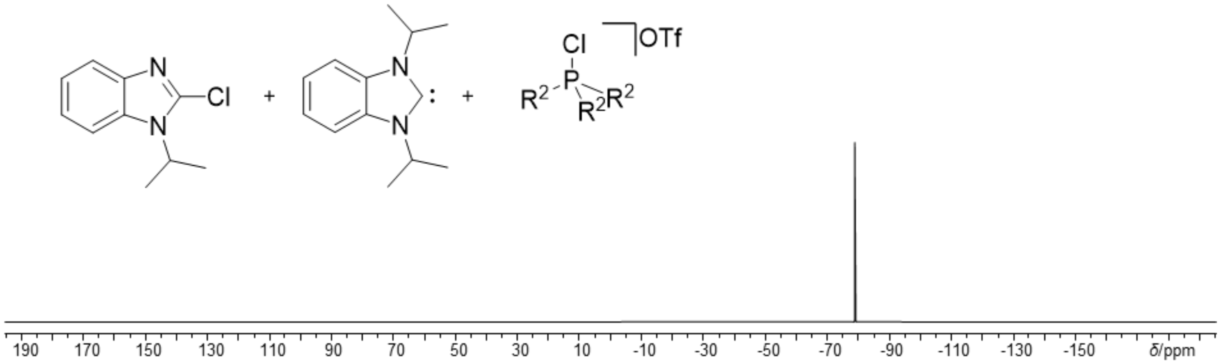

Figure S97. ^{19}F NMR spectrum of the product mixture from the reaction of phosphine **2** with (**5-**Cl)OTf in the presence of **6** (376 MHz, THF-d_8).

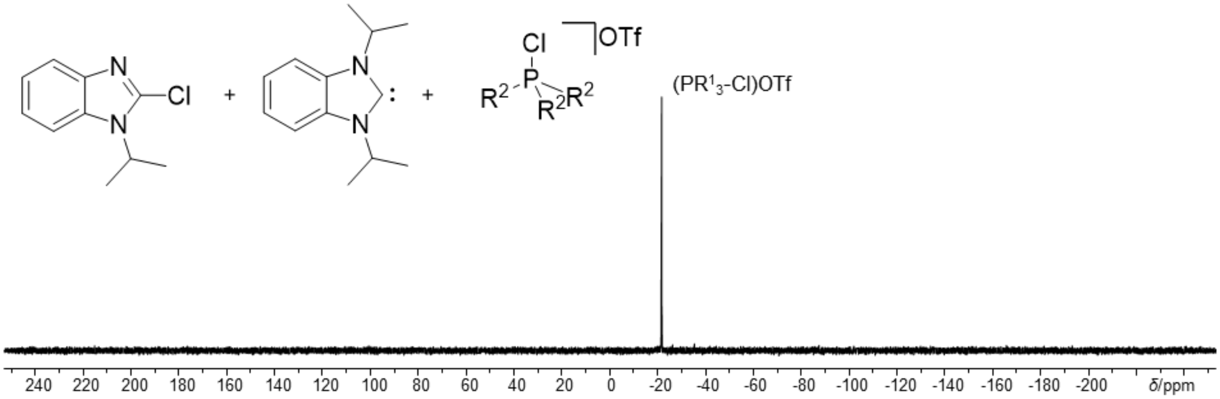

Figure S98. ^{31}P NMR spectrum of the product mixture from the reaction of phosphine **2** with (**5-**Cl)OTf in the presence of **6** (162 MHz, THF-d_8).

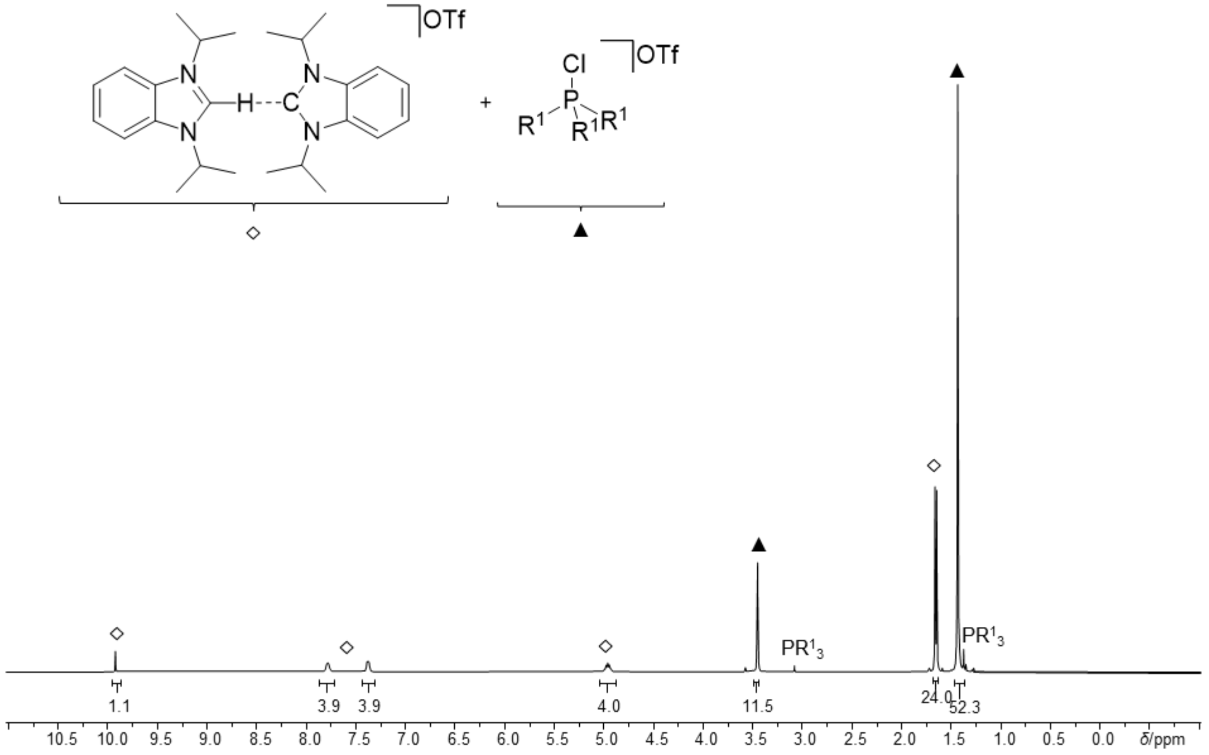

Figure S99. ^{1}H NMR spectrum of the products from the reaction of phosphine **1** with a mixture of 1,3-di-*iso*-propylbenzimidazolium triflate (**5-**H)OTf and 2-chloro-1,3-di-*iso*-propylbenzimidazolium triflate (**5**-Cl)OTf (400 MHz, THF-*d*8).

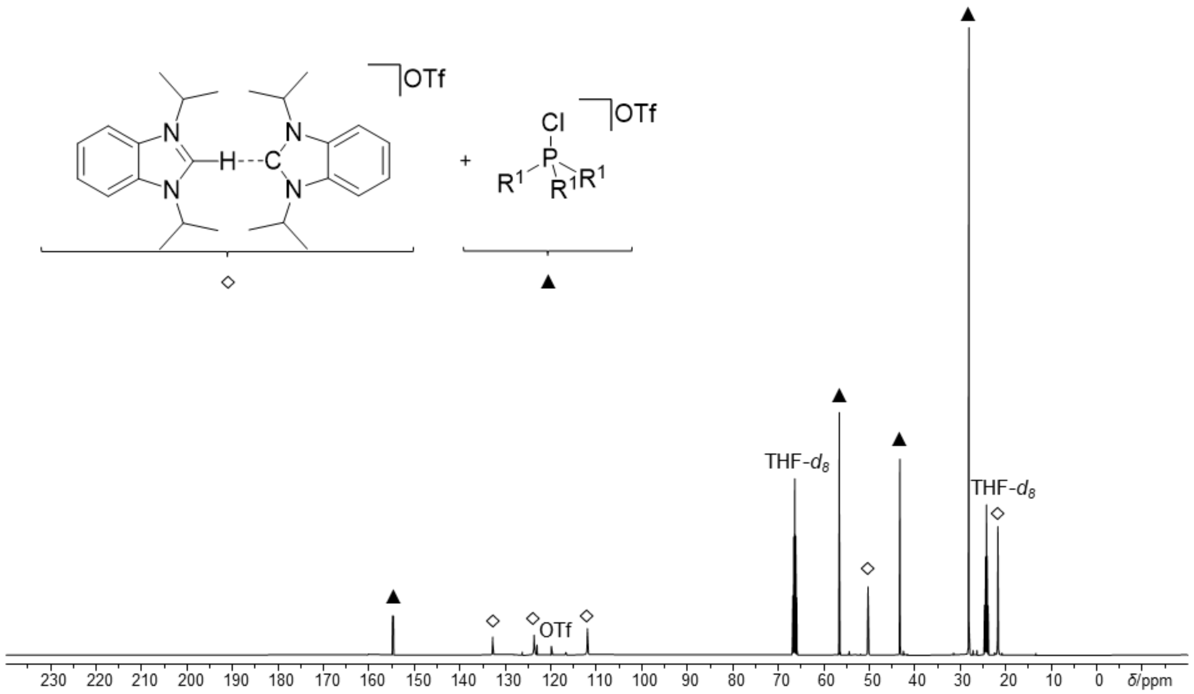

Figure S100. $^{13}C\{^{1}H\}$ NMR spectrum of the reaction of phosphine **1** with 1,3-di-*iso*-propylbenzimidazolium triflate (**5**-H)OTf and 2-chloro-1,3-di-*iso*-propylbenzimidazolium triflate (**5**-Cl)OTf (400 MHz, THF-*d*8). *Note: No resonances associated to the C2 atoms are detected due to the dynamic exchange of the imidazolium proton.*

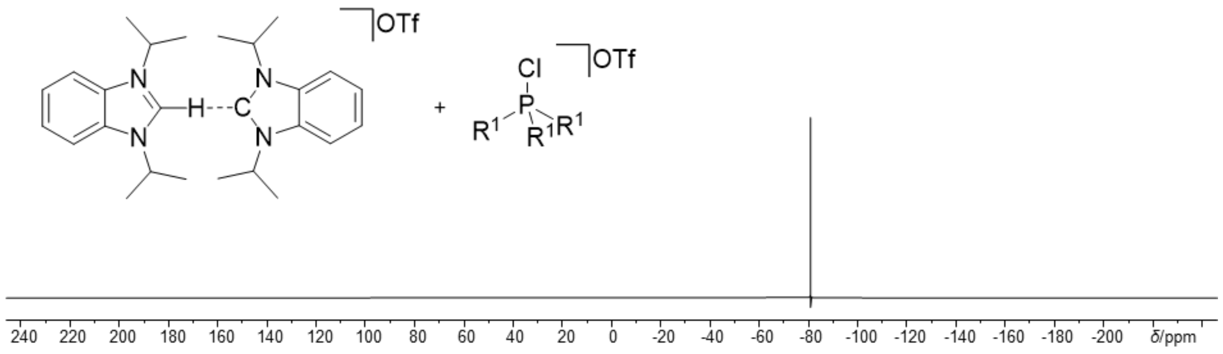

Figure S101. ^{19}F NMR spectrum of the reaction of phosphine **1** with 1,3-di-*iso*-propylbenzimidazolium triflate (**5**-H)OTf and 2-chloro-1,3-di-*iso*-propylbenzimidazolium triflate (**5**-Cl)OTf (373 MHz, THF-d_8).

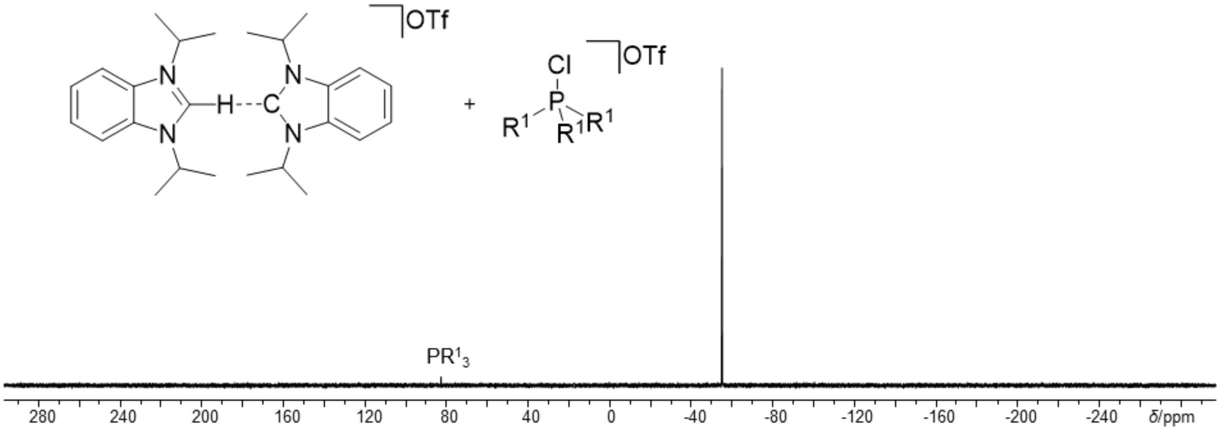

Figure S102. ^{31}P NMR spectrum of the reaction of phosphine **1** with 1,3-di-*iso*-propylbenzimidazolium triflate (**5**-H)OTf and 2-chloro-1,3-di-*iso*-propylbenzimidazolium triflate (**5**-Cl)OTf (162 MHz, THF-d_8).

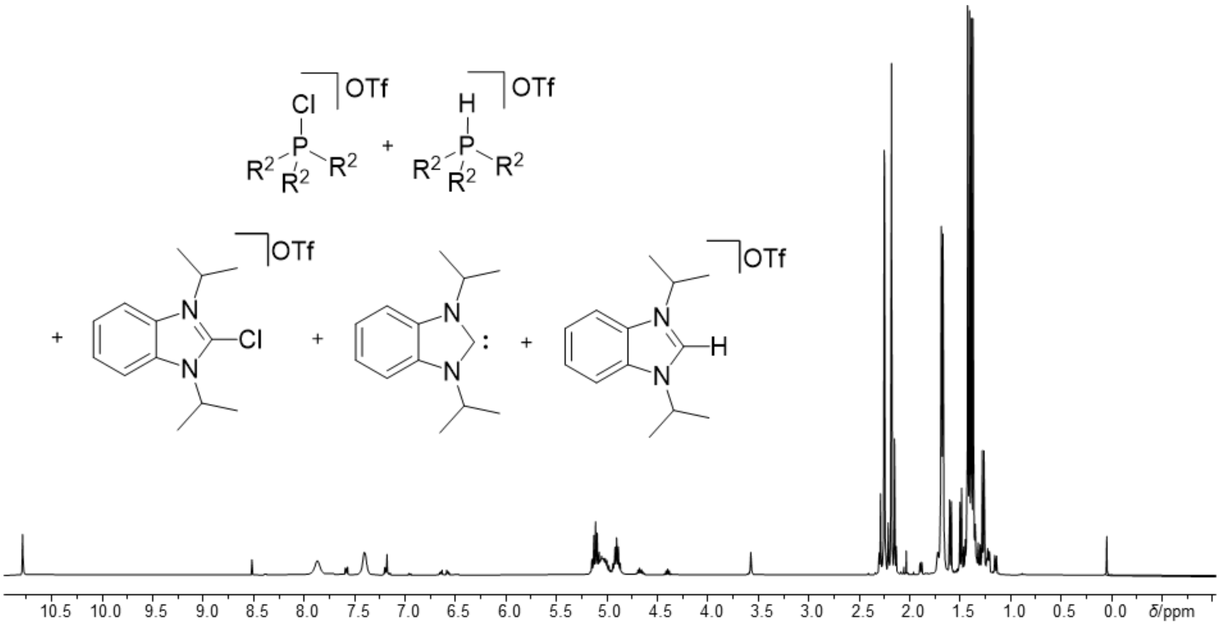

Figure S103. ^{1}H NMR spectrum of the reaction of phosphine **2** with 1,3-di-*iso*-propylbenzimidazolium triflate (**5**-H)OTf and 2-chloro-1,3-di-*iso*-propylbenzimidazolium triflate (**5**-Cl)OTf (400 MHz, THF-d_8).

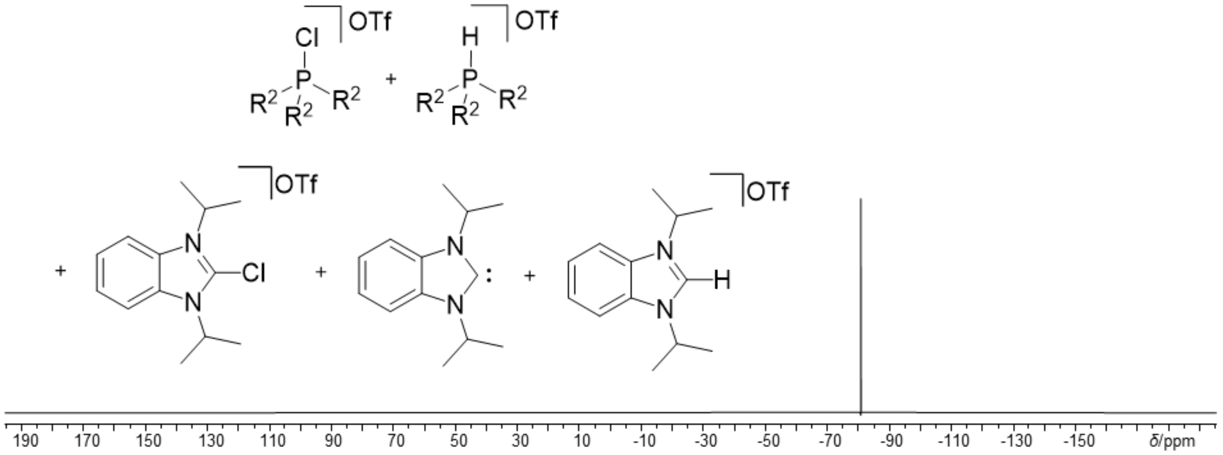

Figure S104. ^{19}F NMR spectrum of the reaction of phosphine **2** with 1,3-di-*iso*-propylbenzimidazolium triflate (**5**-H)OTf and 2-chloro-1,3-di-*iso*-propylbenzimidazolium triflate (**5**-Cl)OTf (377 MHz, THF-d_8).

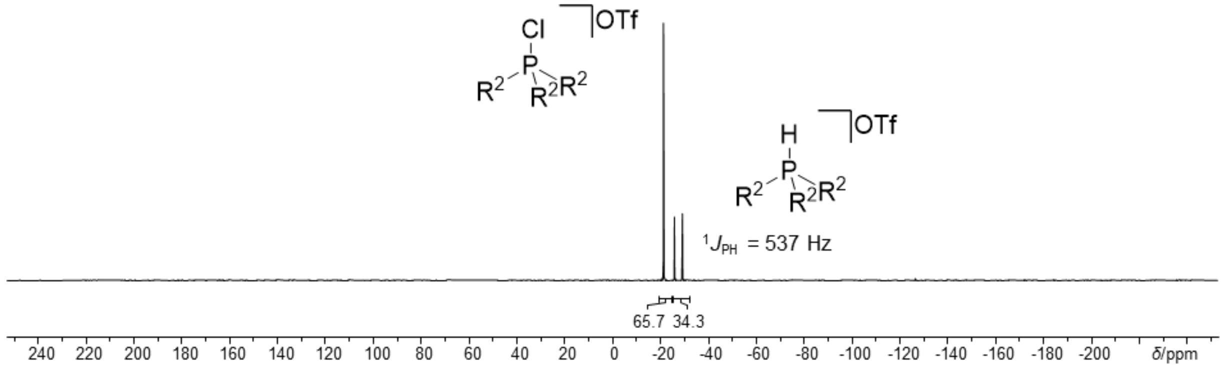

Figure S105. ^{31}P NMR spectrum (D_1 = 25 sec, aq. time = 0.66 sec., zg30) of the reaction of phosphine **2** with 1,3-di-*iso*-propylbenzimidazolium triflate (**5**-H)OTf and 2-chloro-1,3-di-*iso*-propylbenzimidazolium triflate (**5**-Cl)OTf (162 MHz, THF-d_8).

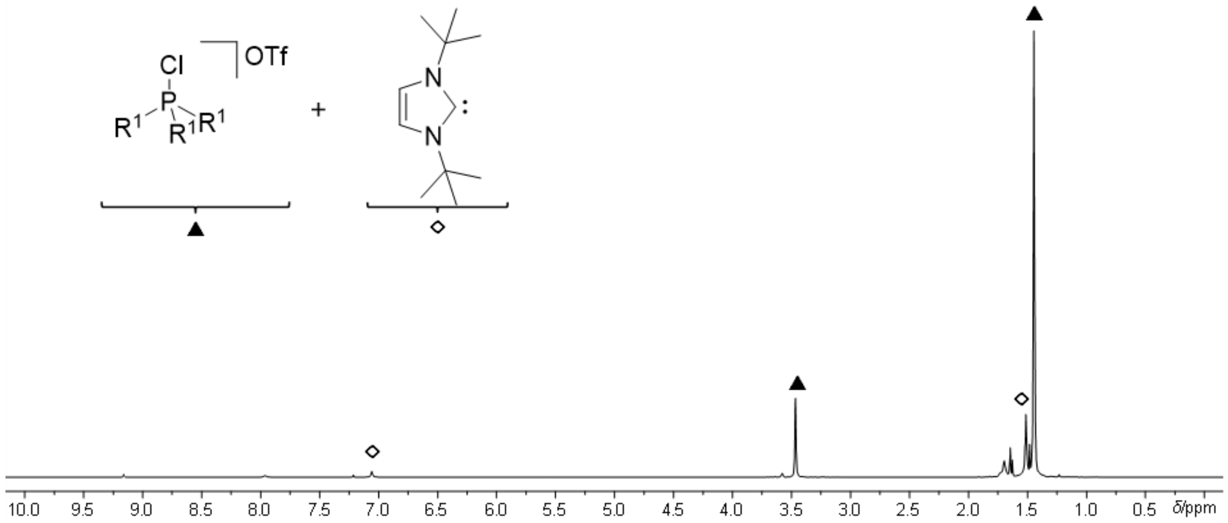

Figure S106. ^{1}H NMR spectrum of the reaction mixture IAP **1** + (**7**-Cl)OTf to give (**1**-Cl)OTf + **7** (300 MHz, THF-d_8).

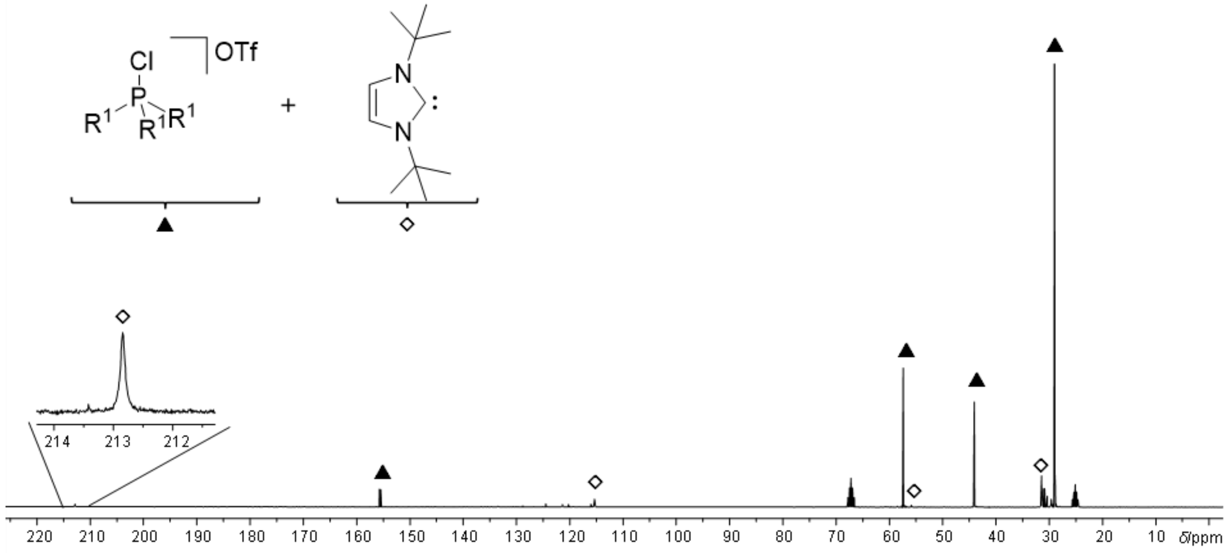

Figure S107. ^{13}C{^{1}H} NMR spectrum of the reaction mixture IAP **1** + (**7**-Cl)OTf to give (**1**-Cl)OTf + **7** (75 MHz, THF-d_8).

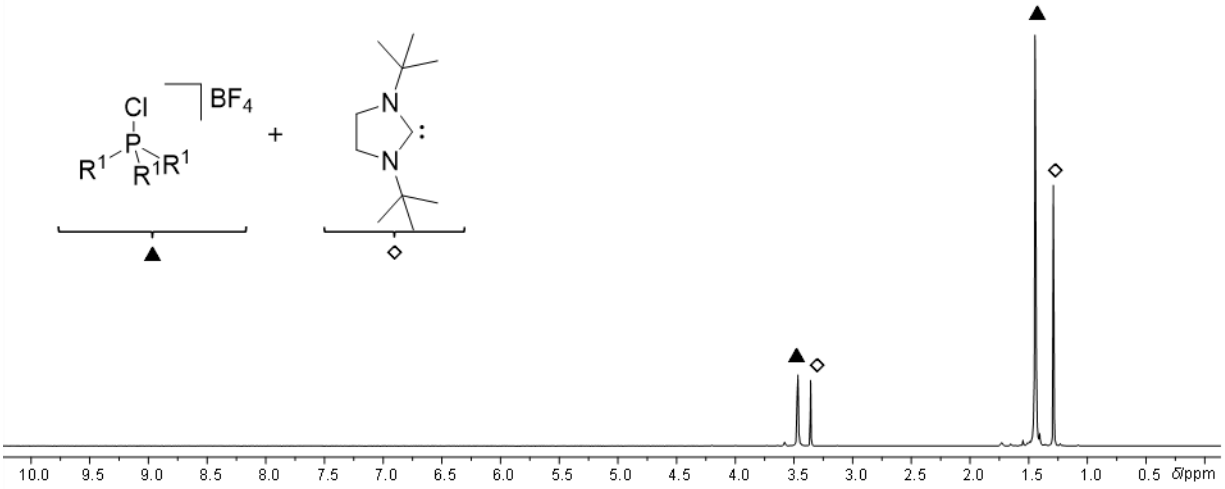

Figure S108. ^{1}H NMR spectrum of the reaction mixture IAP **1** + (**8**-Cl)BF_4 to give (**1**-Cl)BF_4 + **8** (300 MHz, THF-d_8).

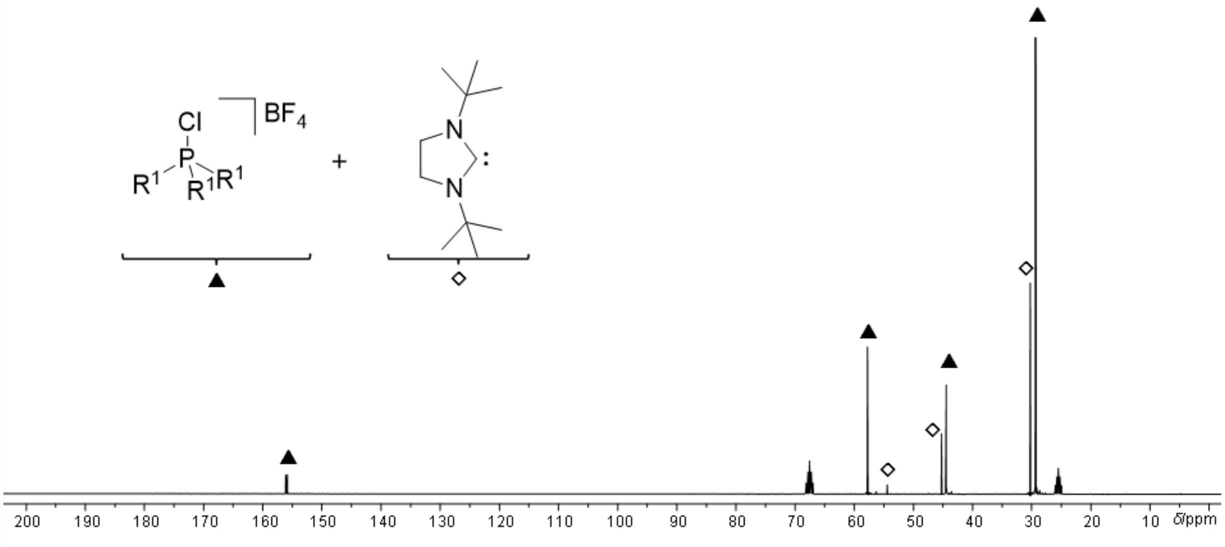

Figure S109. ^{13}C{^{1}H} NMR spectrum of the reaction mixture IAP **1** + (**8**-Cl)BF_4 to give (**1**-Cl)BF_4 + **8** (75 MHz, THF-d_8). The resonance for the C_{NHC} carbon atom of NHC **8** was not detected. It was detected for **8** isolated from the mixture (Figure S111).

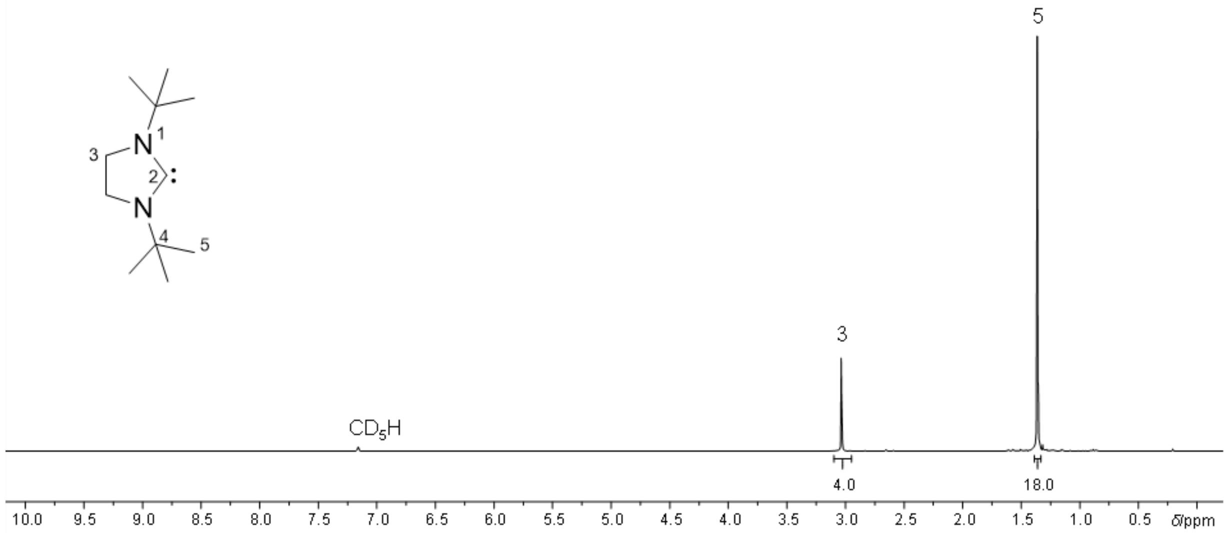

Figure S110. ^{1}H NMR spectrum of the *n*-hexane extract from the reaction **1** + (**8**-Cl)BF_4 containing NHC **8** (300 MHz, C_6D_6).

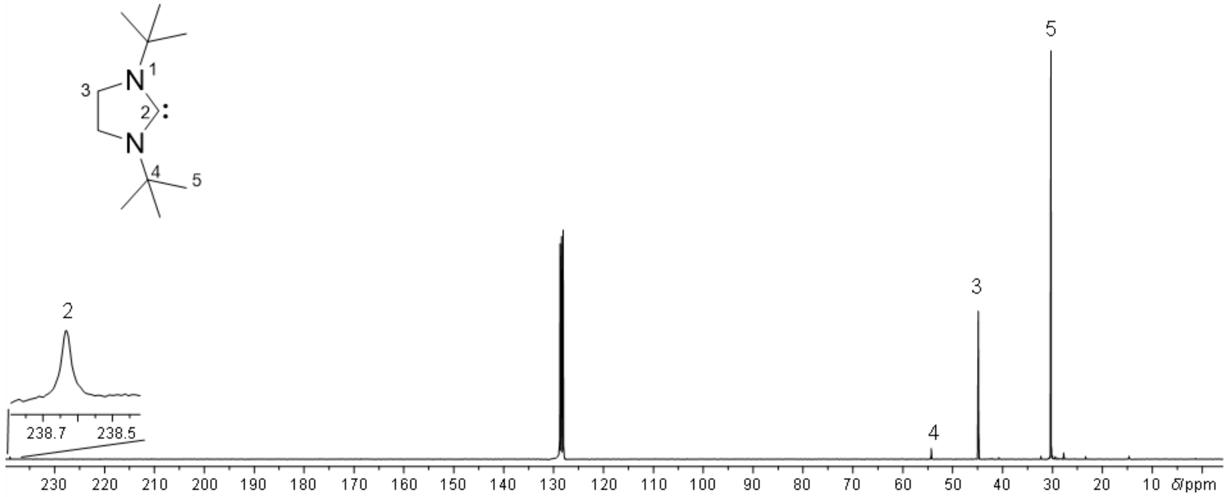

Figure S111. $^{13}C\{^{1}H\}$ NMR spectrum of the *n*-hexane extract from the reaction **1** + (**8**-Cl)BF_4 containing NHC **8** (75 MHz, C_6D_6).

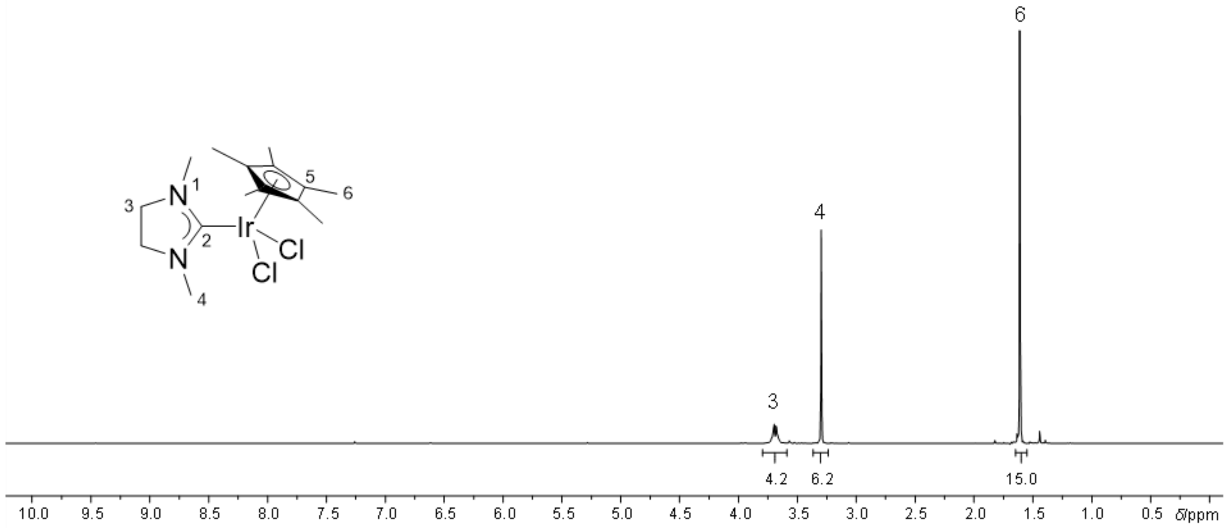

Figure S112. ^{1}H NMR spectrum of complex [Ir(**9**)Cl_2Cp*] [**10**] (300 MHz, $CDCl_3$).

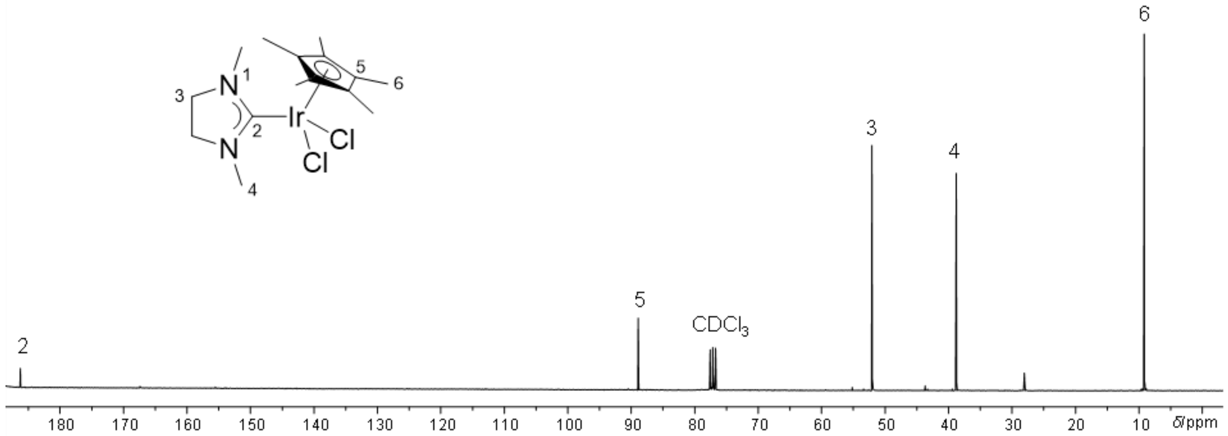

Figure S113. ^{13}C{^{1}H} NMR spectrum of complex [Ir(**9**)Cl_2Cp*] [**10**] (75 MHz, $CDCl_3$).

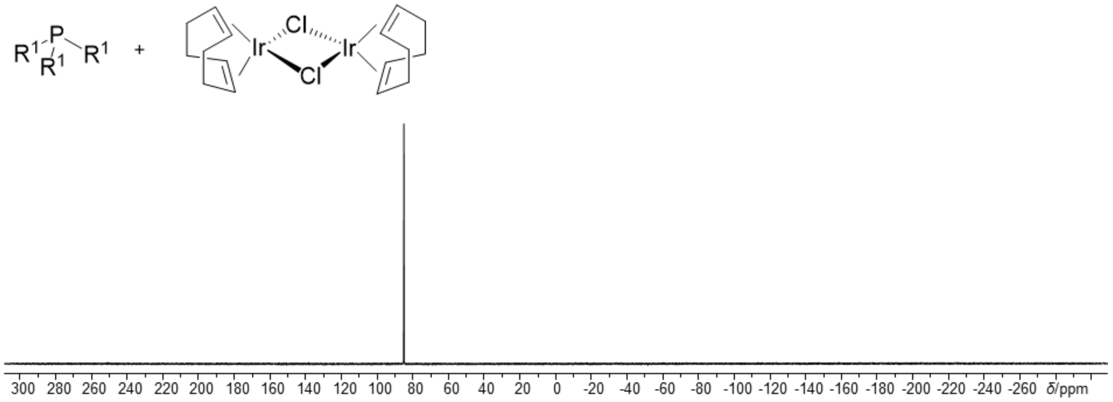

Figure S114. ^{31}P NMR spectrum of the reaction products from the reaction of phosphine **1** with $[IrCl(cod)]_2$ (162 MHz, THF).

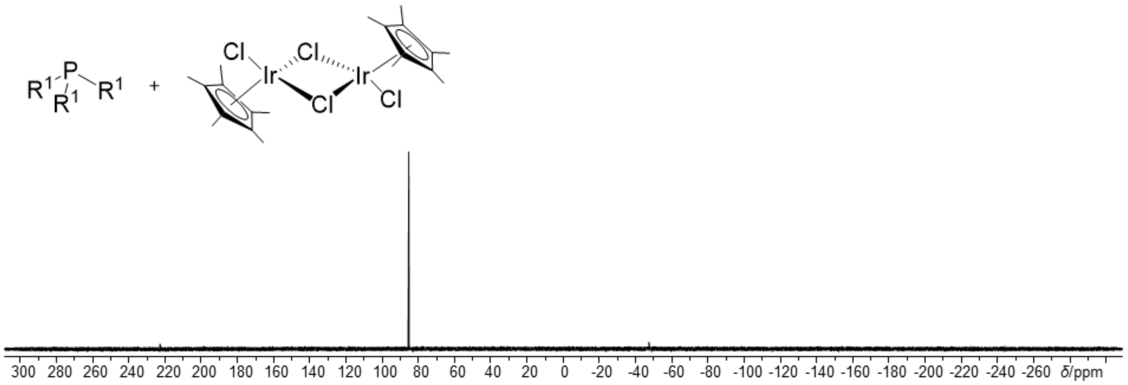

Figure S115. ^{31}P NMR spectrum of the reaction products from the reaction of phosphine **1** with $[IrCl(Cp^*)]_2$ (162 MHz, THF).

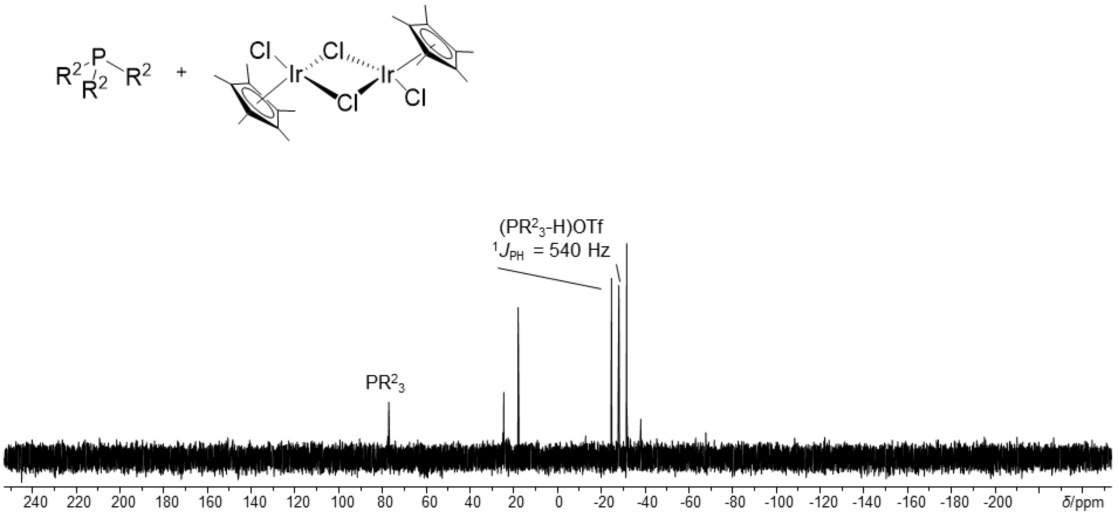

Figure S116. ^{31}P NMR spectrum of the reaction products from the reaction of phosphine **2** with $[IrCl_2(Cp^*)]_2$ (162 MHz, THF).

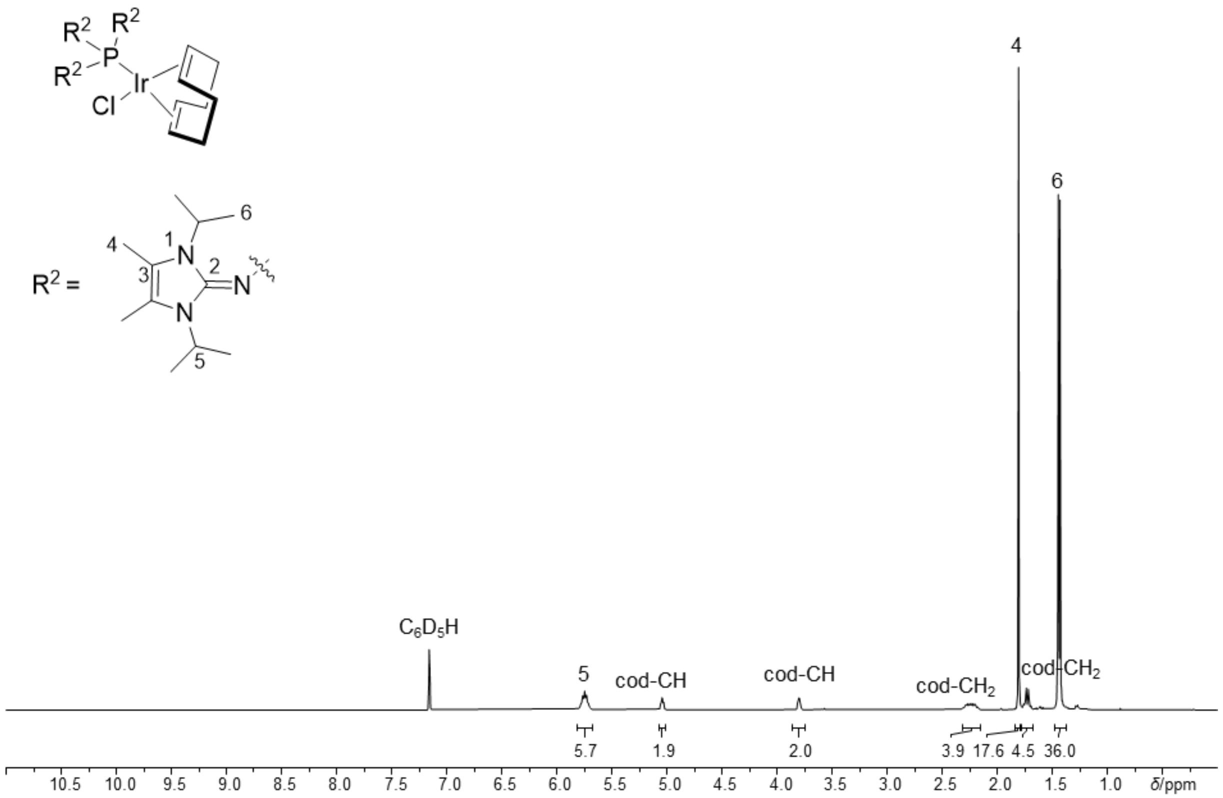

Figure S117. ^{1}H NMR spectrum of complex [**11**] (400 MHz, C_6D_6).

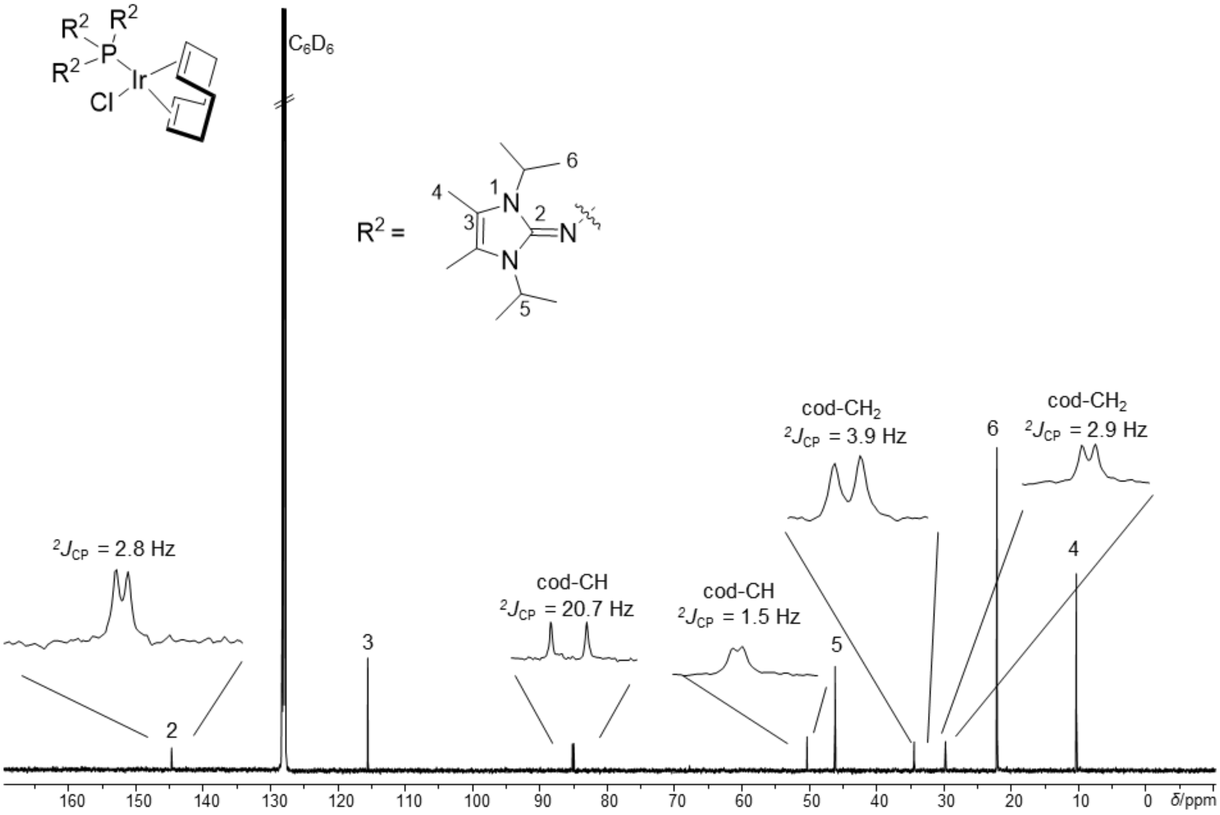

Figure S118. $^{13}C\{^{1}H\}$ NMR spectrum of complex [**11**] (101 MHz, C_6D_6).

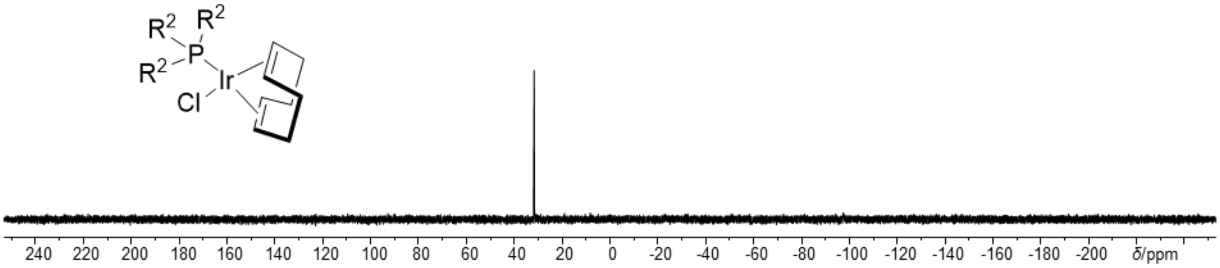

Figure S119. ^{31}P NMR spectrum of complex [**11**] (162 MHz, C_6D_6).

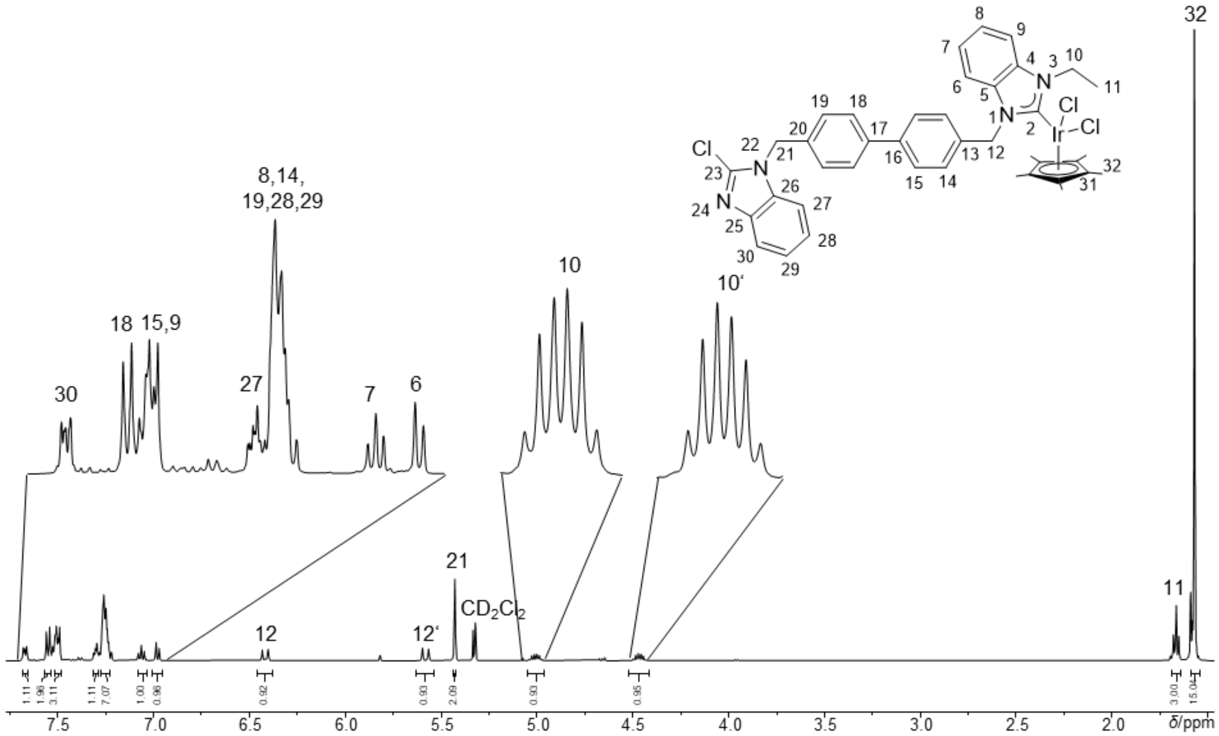

Figure S120. ^{1}H NMR spectrum of complex [**13**] (500 MHz, CD_2Cl_2).

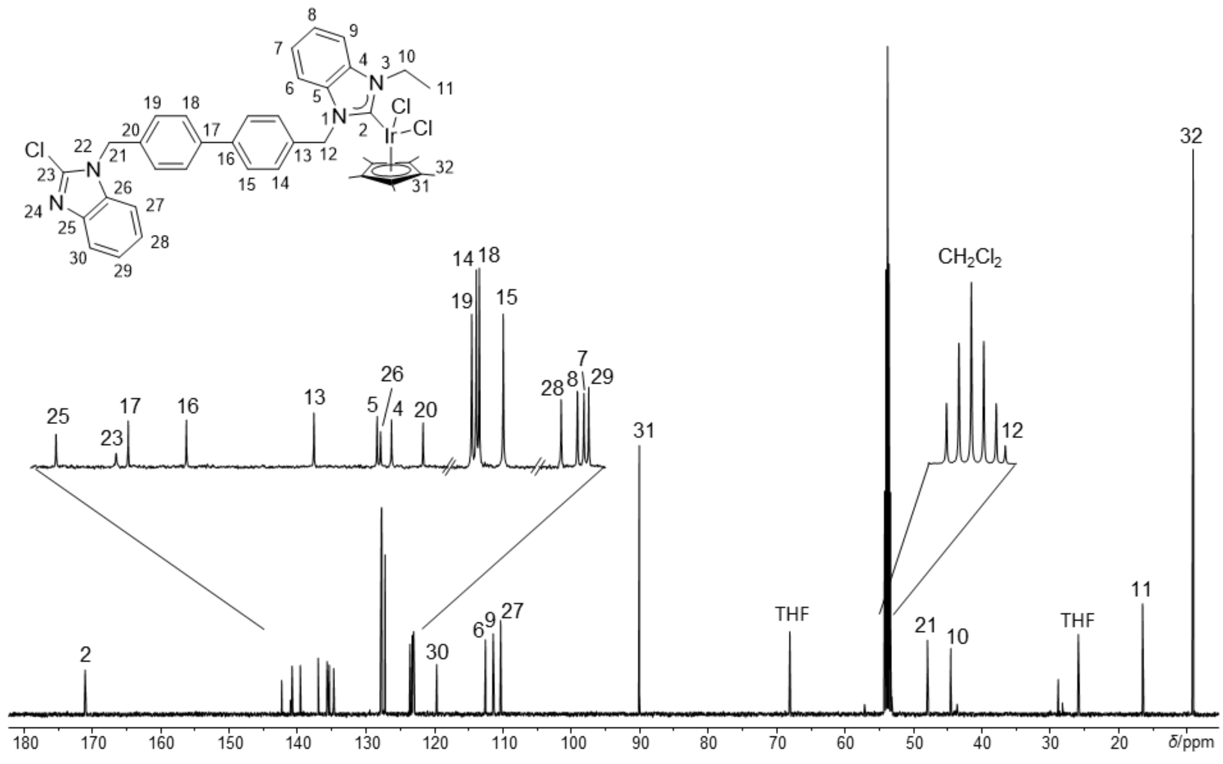

Figure S121. $^{13}C\{^1H\}$ NMR spectrum of complex [**13**] (126 MHz, CD_2Cl_2).

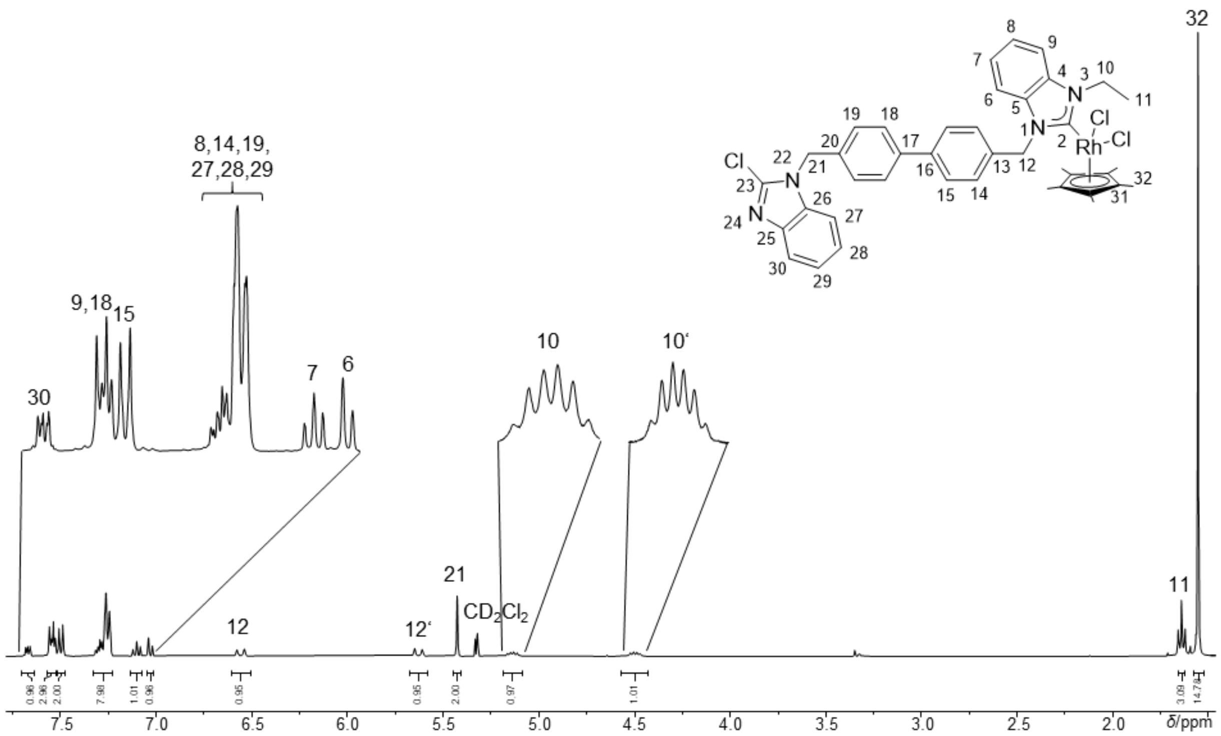

Figure S122. ^{1}H NMR spectrum of complex [**14**] (400 MHz, CD_2Cl_2).

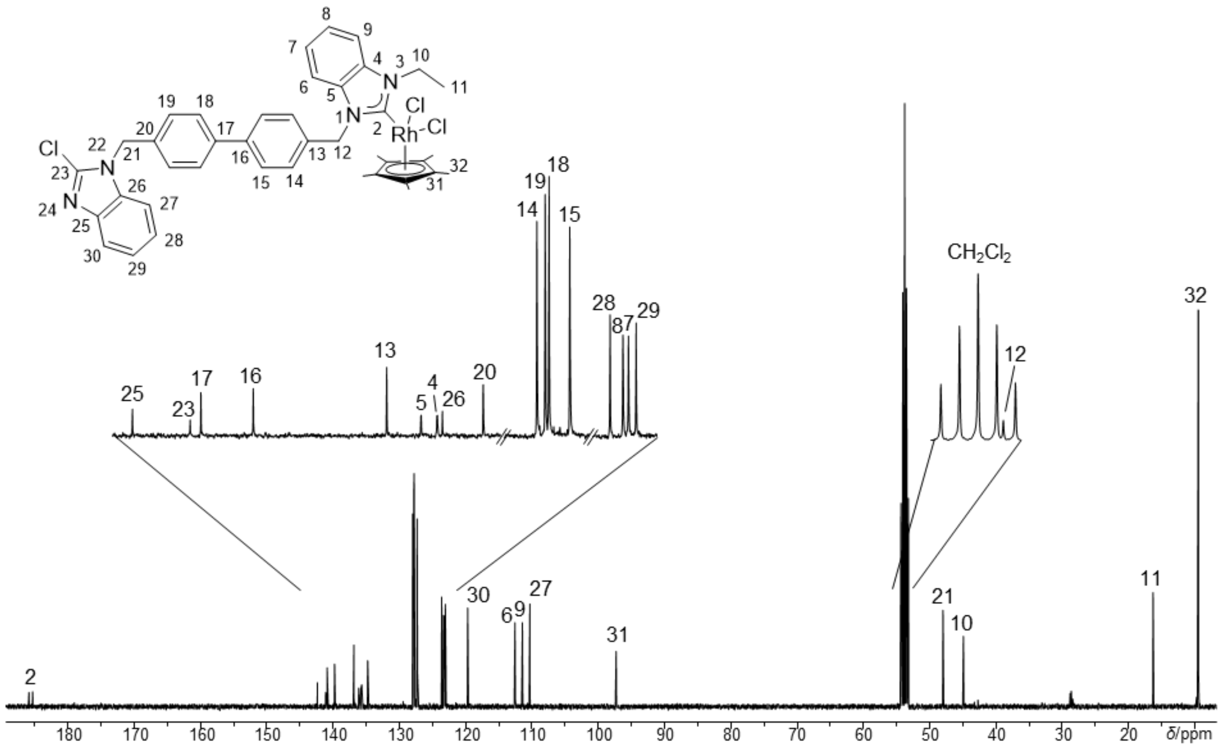

Figure S123. $^{13}C\{^{1}H\}$ NMR spectrum of complex [**14**] (101 MHz, CD_2Cl_2).

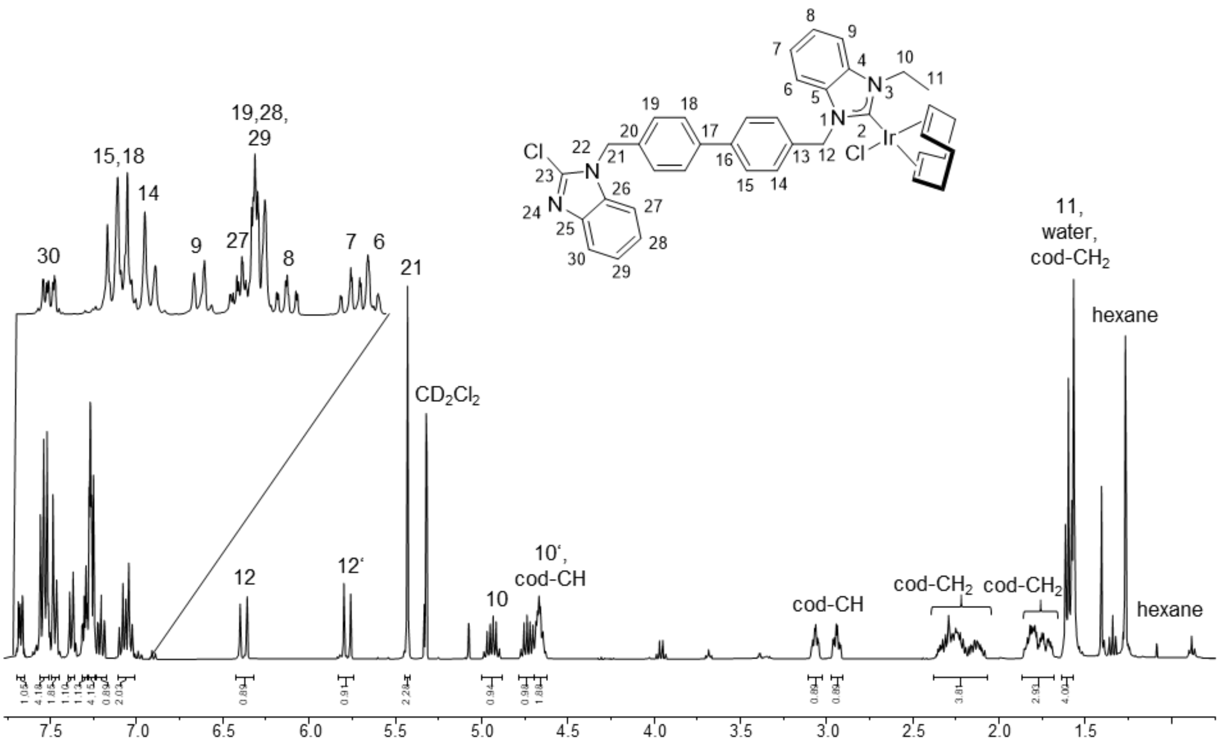

Figure S124. ^{1}H NMR spectrum of complex [**15**] (400 MHz, CD_2Cl_2).

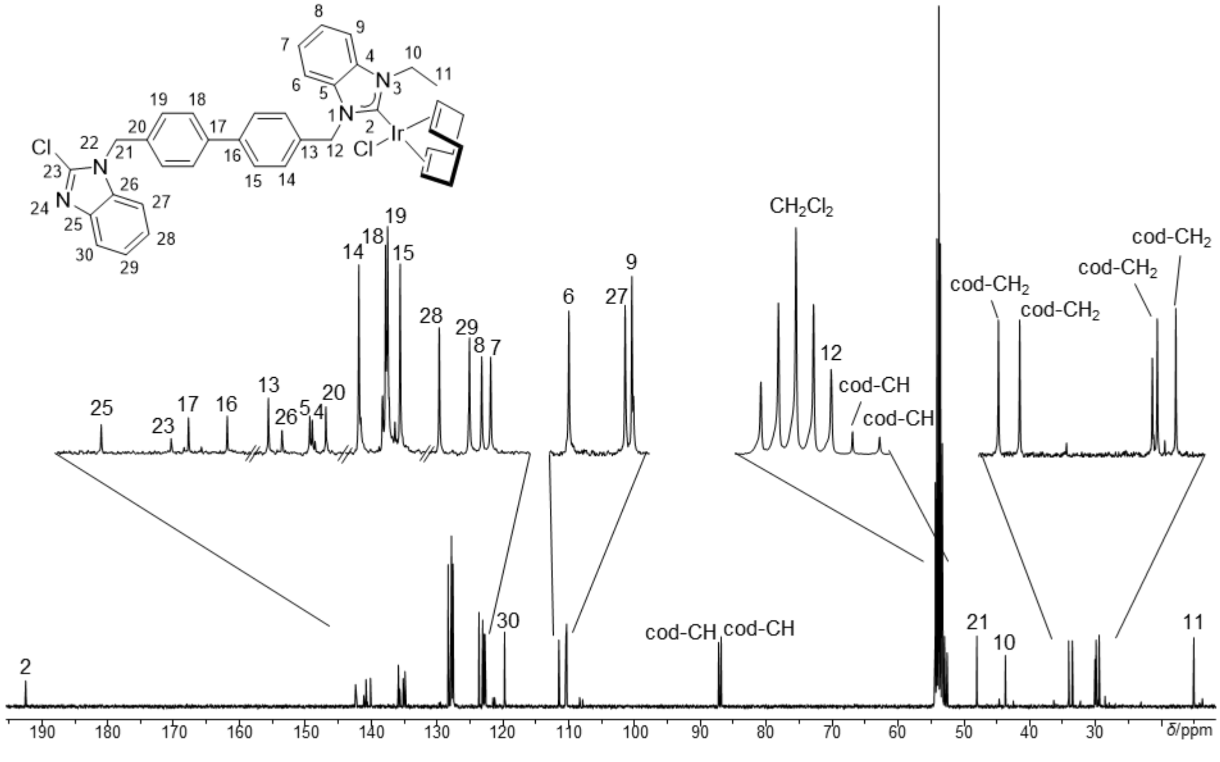

Figure S125. $^{13}C\{^{1}H\}$ NMR spectrum of complex [**15**] (101 MHz, CD_2Cl_2).

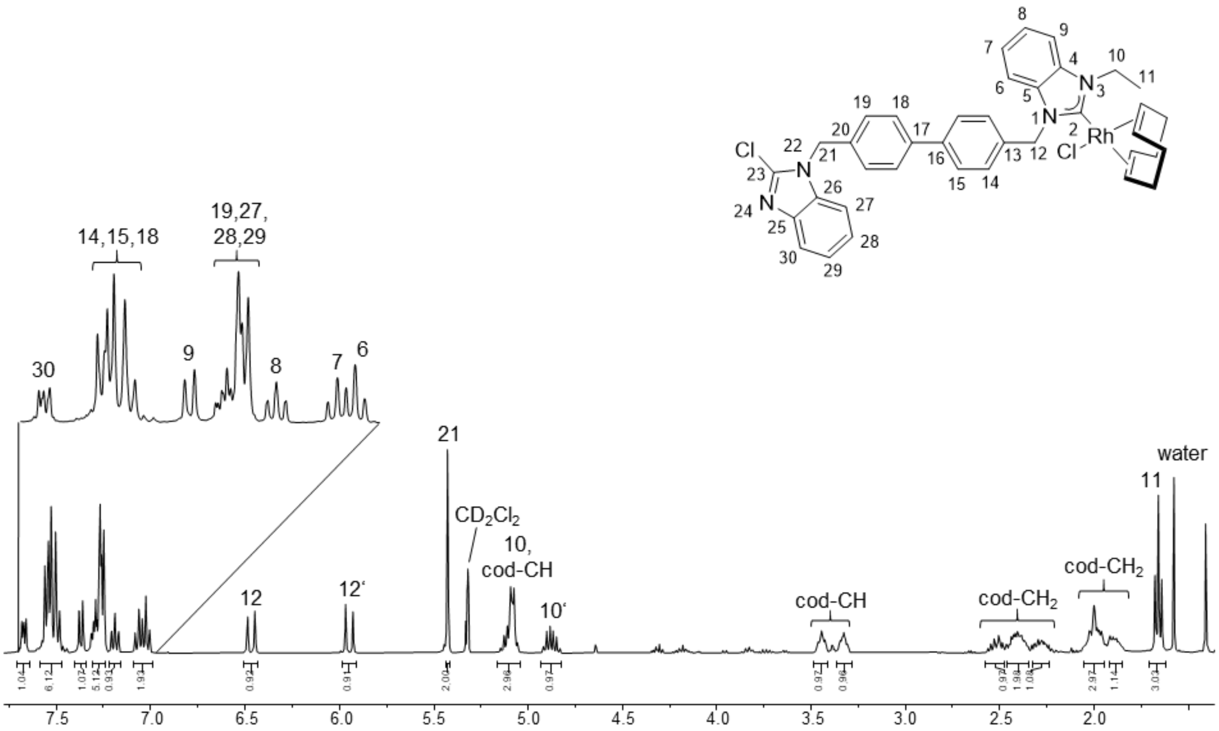

Figure S126. ^{1}H NMR spectrum of complex [**16**] (400 MHz, CD_2Cl_2).

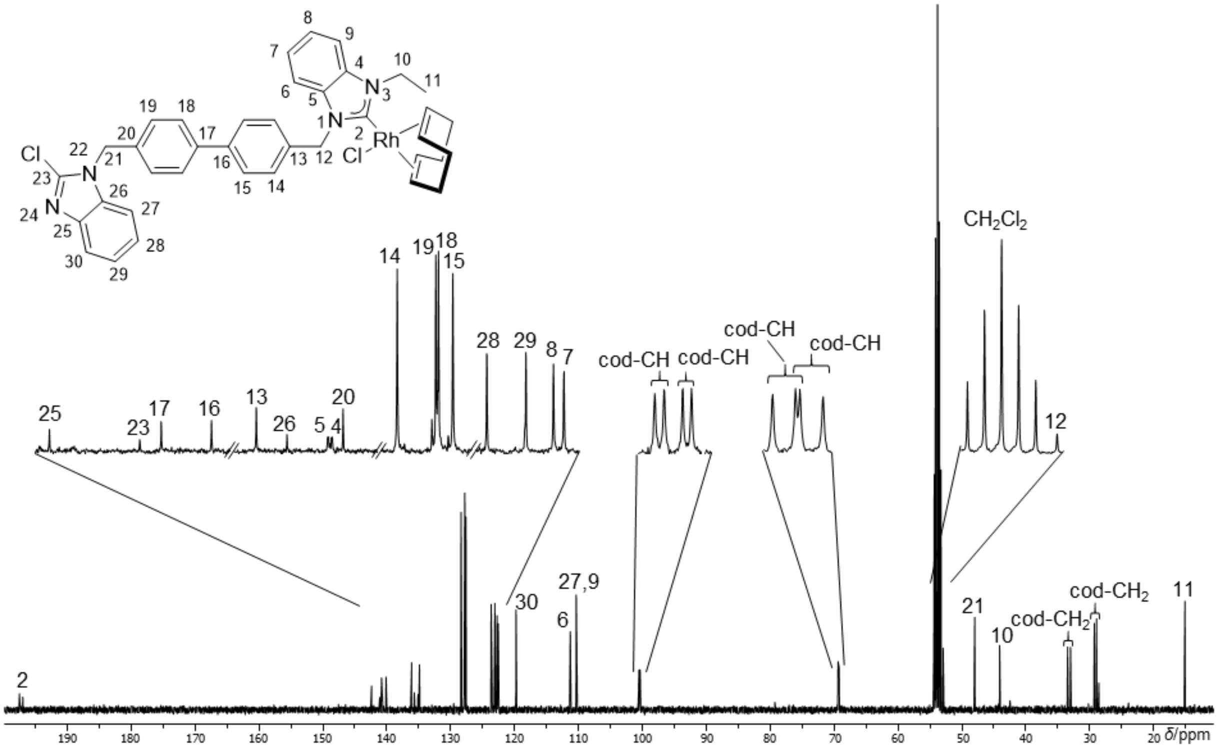

Figure S127. $^{13}C\{^{1}H\}$ NMR spectrum of complex [**16**] (101 MHz, CD_2Cl_2).

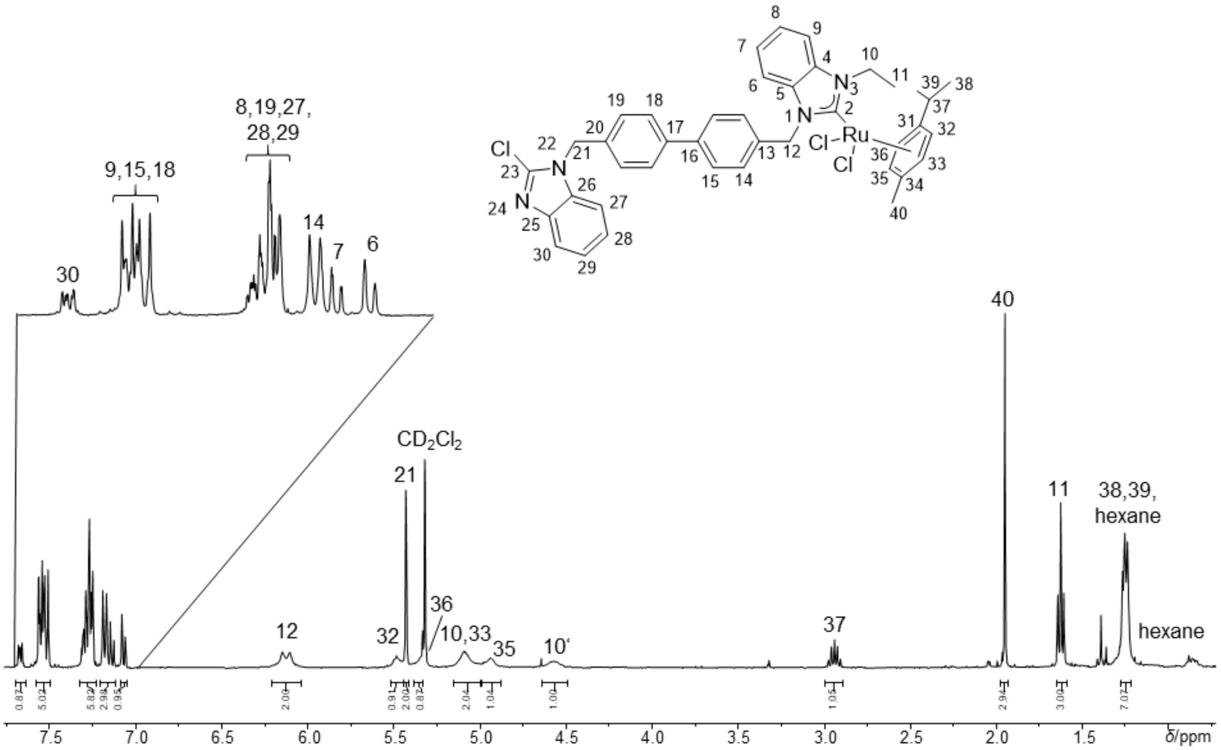

Figure S128. ^{1}H NMR spectrum of complex [**17**] (400 MHz, CD_2Cl_2).

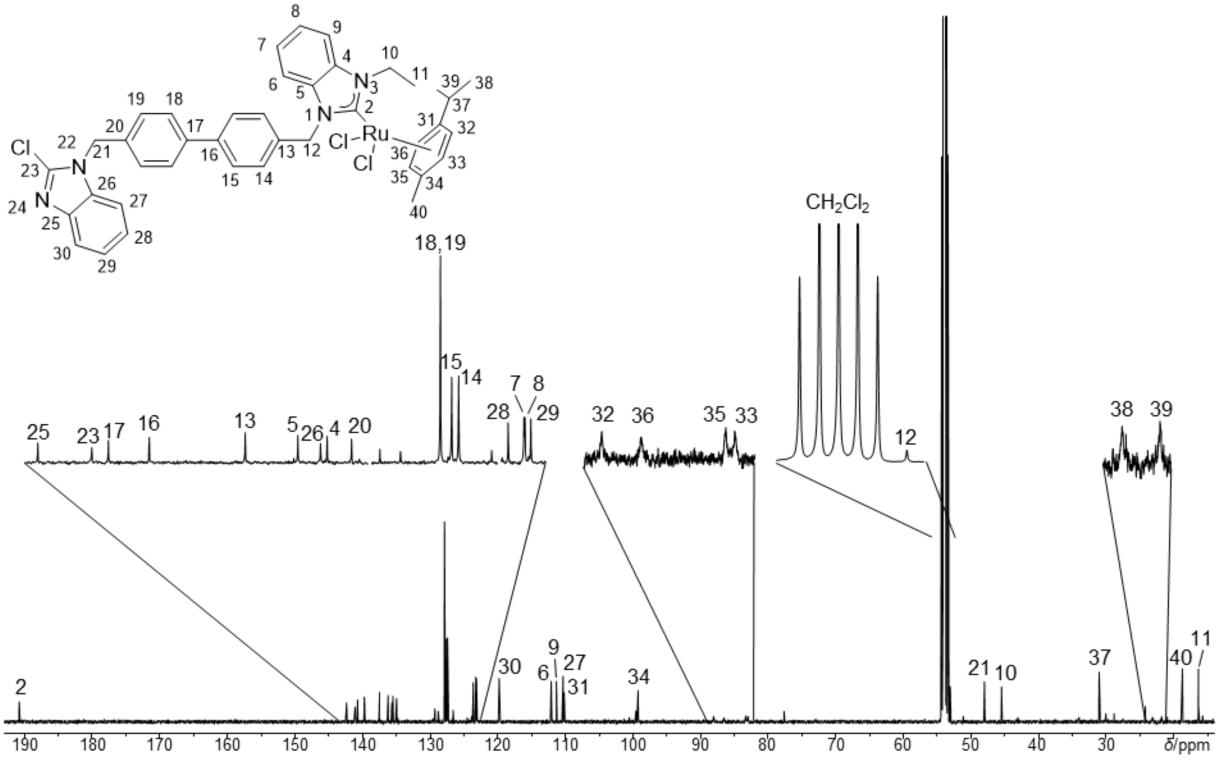

Figure S129. $^{13}C\{^{1}H\}$ NMR spectrum of complex [**17**] (126 MHz, CD_2Cl_2).

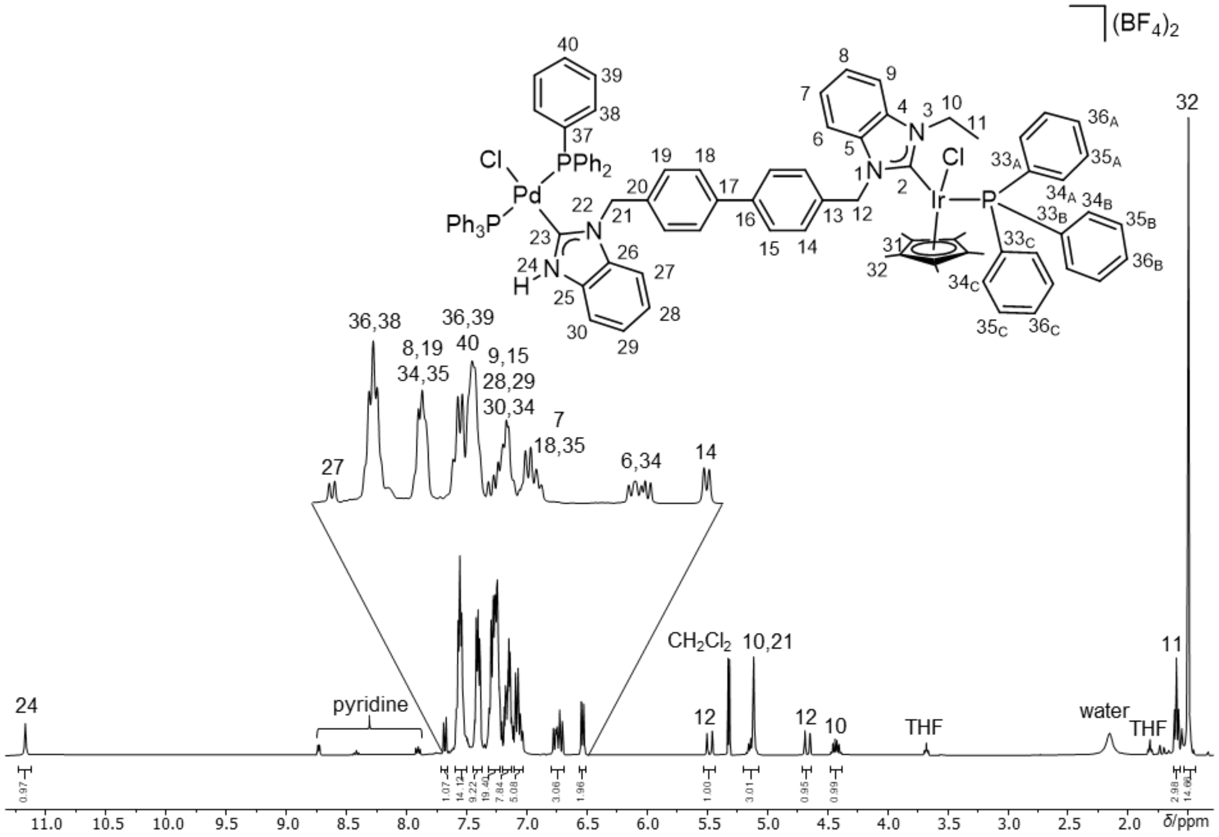

Figure S130. ^1H NMR spectrum of atropisomer **A** of [**18**]$(BF_4)_2$. The phenyl groups of the Ir-PPh_3 ligand are chemically different leading to three sets of resonaces A, B, C due to the chirality at the iridium atom. These resonances have been completely assigned in section 1.32 while in the plot only the atom numbers of the phenyl group atoms are given (400 MHz, CD_2Cl_2).

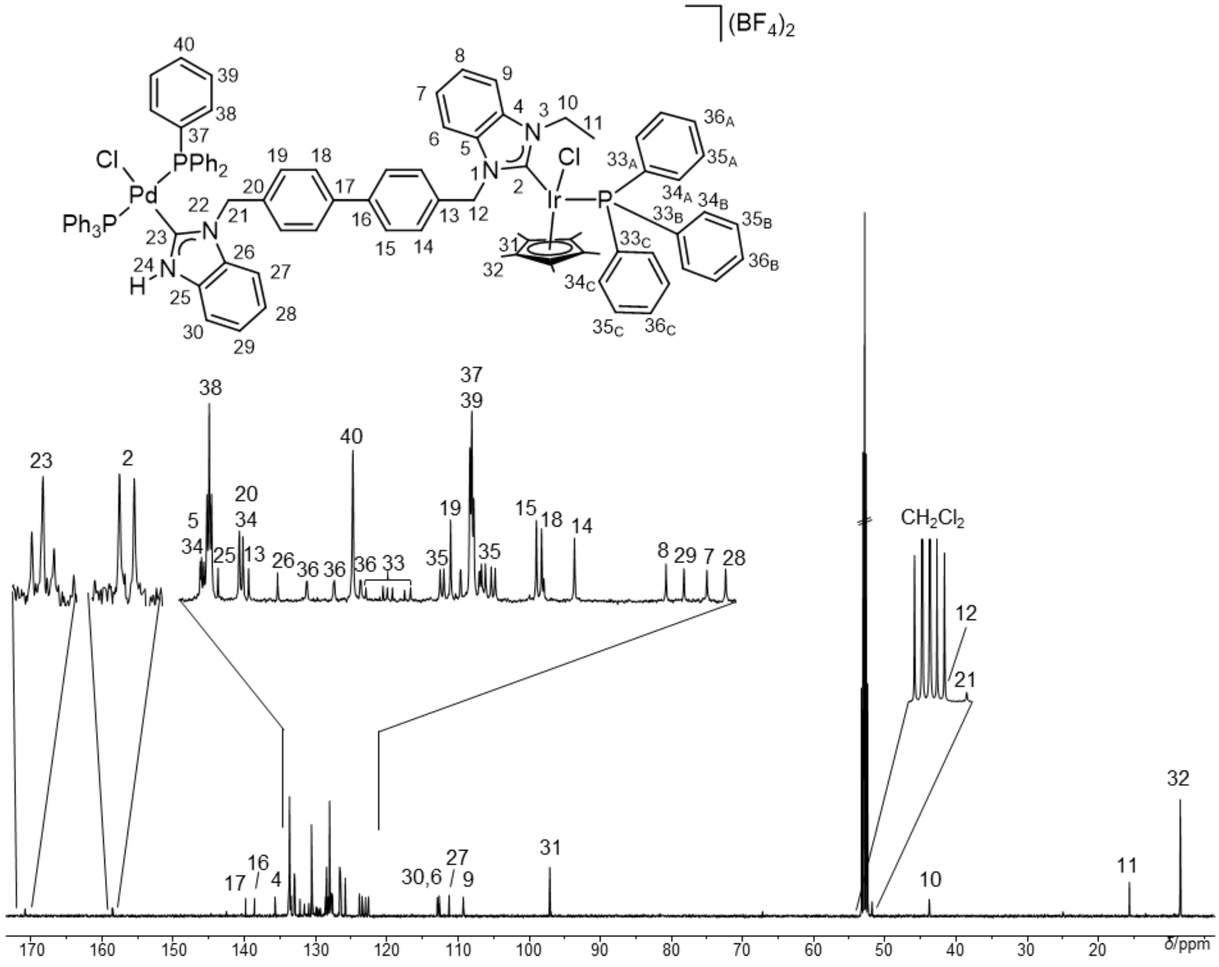

Figure S131. $^{13}C\{^{1}H\}$ NMR spectrum of atropisomer **A** of [**18**]$(BF_4)_2$. The phenyl groups of the Ir-PPh_3 ligand are chemically different leading to three sets of resonaces A, B, C due to the chirality at the iridium atom. These resonances have been completely assigned in section 1.32 while in this plot only the atom numbers of the phenyl group atoms are given (126 MHz, CD_2Cl_2).

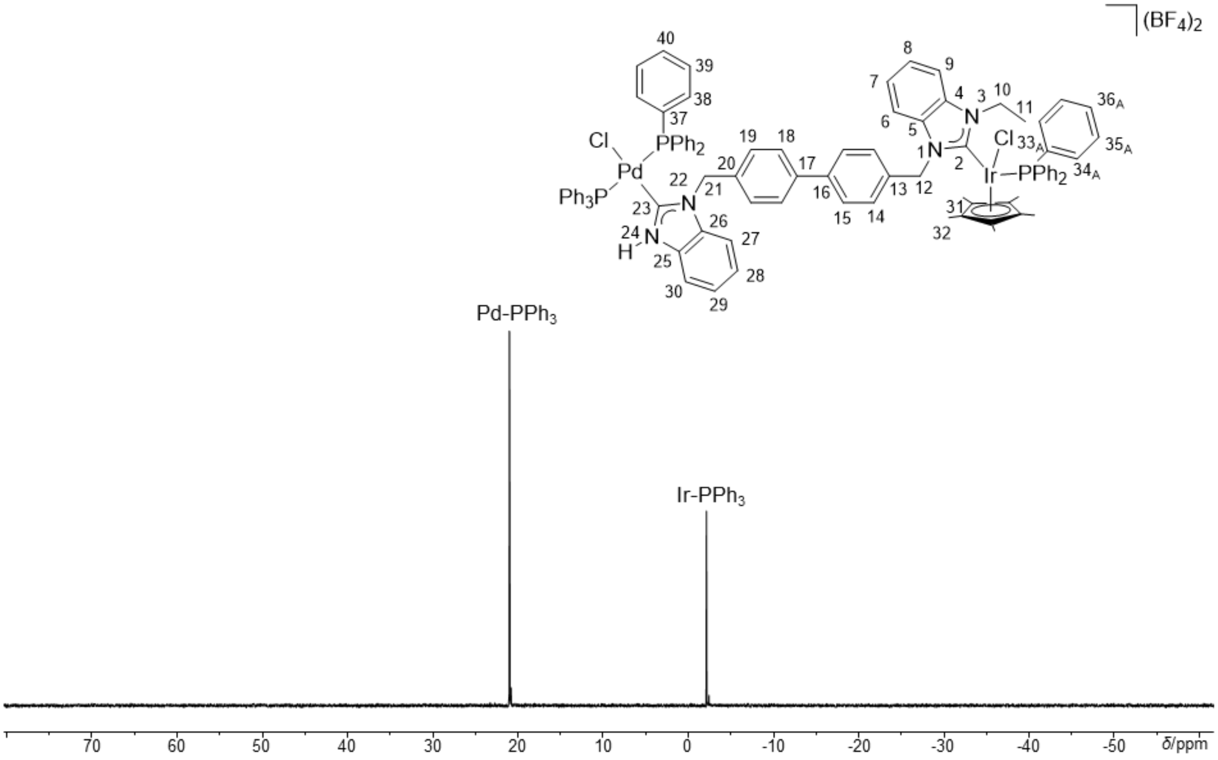

Figure S132. ^{31}P NMR spectrum of isomer **A** of [**18**]$(BF_4)_2$ (162 MHz, CD_2Cl_2).

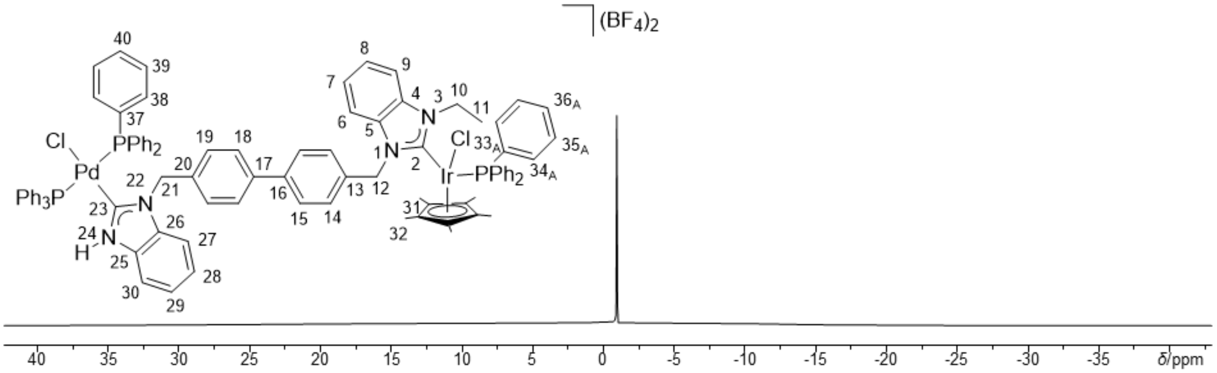

Figure S133. ^{11}B-NMR spectrum of isomer **A** of [**18**]$(BF_4)_2$ (128 MHz, CD_2Cl_2).

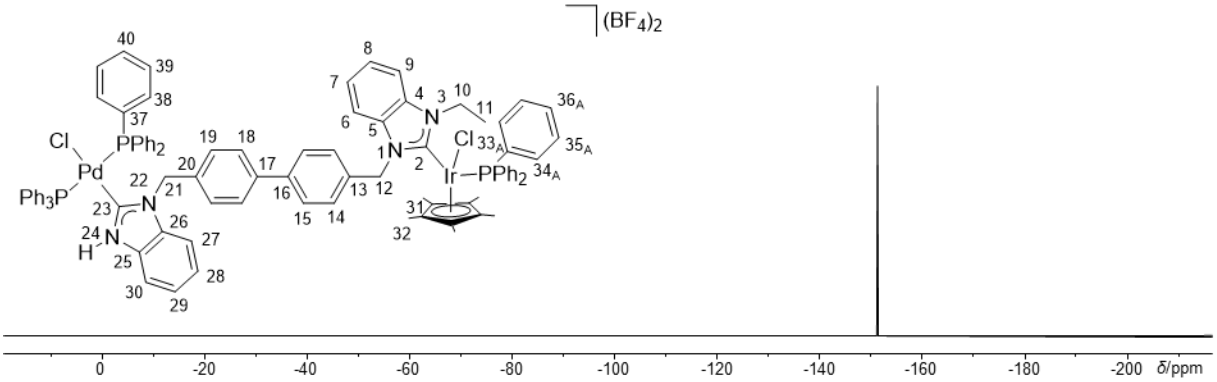

Figure S134. ^{19}F NMR spectrum of ismer **A** of [**18**]$(BF_4)_2$ (376 MHz, CD_2Cl_2).

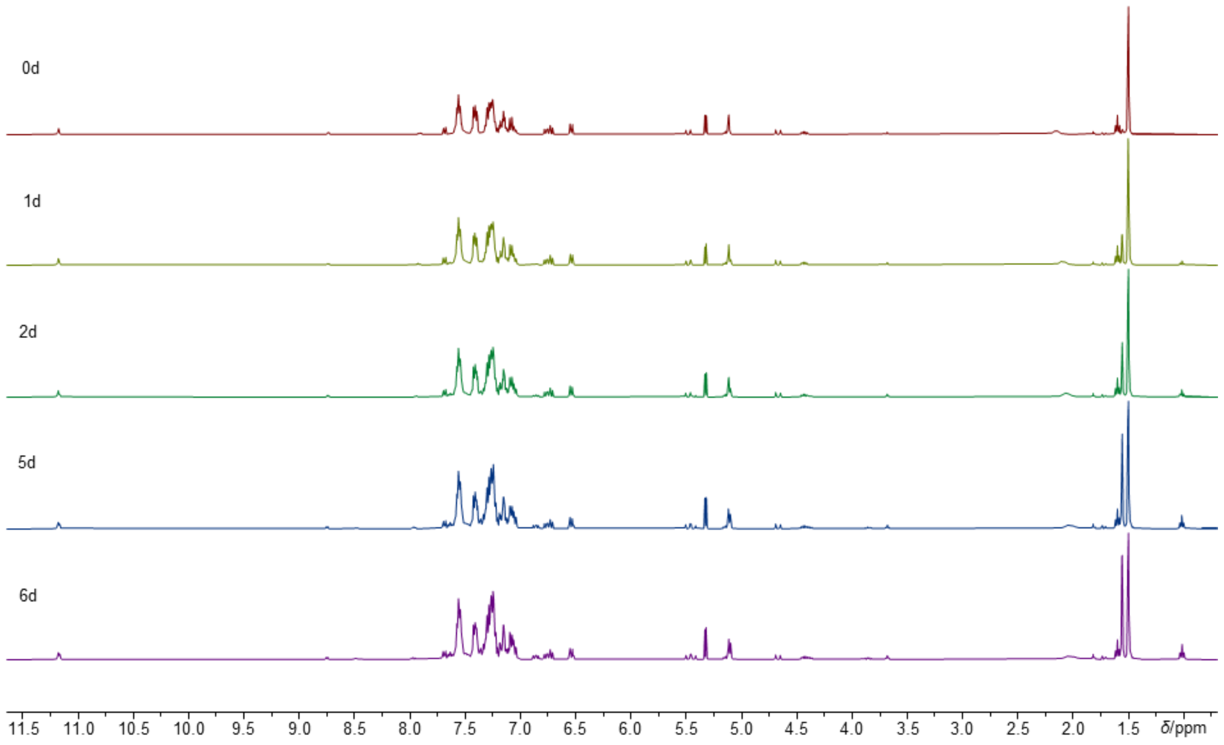

Figure S135. Time dependent ^{1}H NMR spectra showing the conversion of atropisomer **A** of [**18**](BF_4)$_2$ into atropisomer **B** at 25 °C in CD_2Cl_2 over 6d (full spectrum).

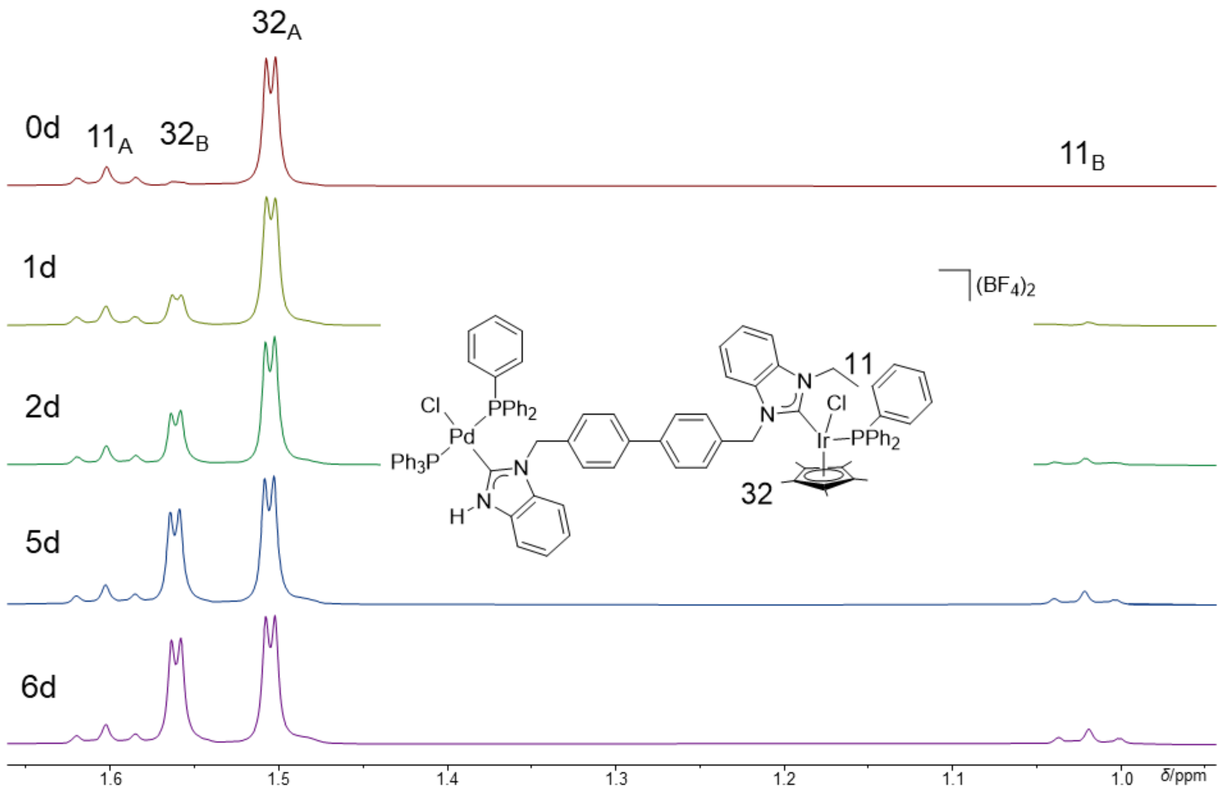

Figure S136. Sections of the ^{1}H NMR spectra showing the conversion of atropisomer **A** of [**18**]$(BF_4)_2$ into atropisomer **B** at 25 °C in CD_2Cl_2 over 6d. Characteristic change of the chemical shifts of the resonances for the methyl goups C11 and C32 are depicted.

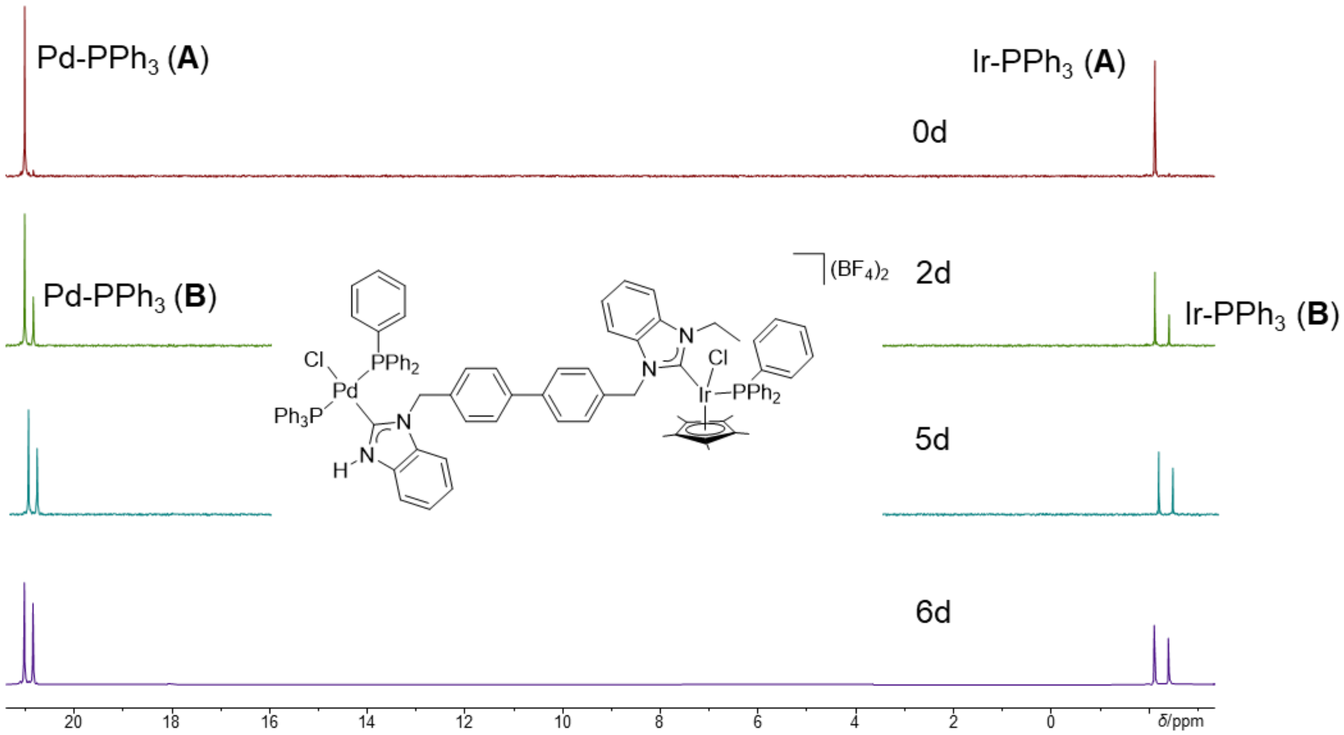

Figure S137. Time dependent ^{31}P NMR spectra showing the conversion of atropisomer **A** of [**18**]$(BF_4)_2$ into atropisomer **B** at 25 °C in CD_2Cl_2 over 6d.

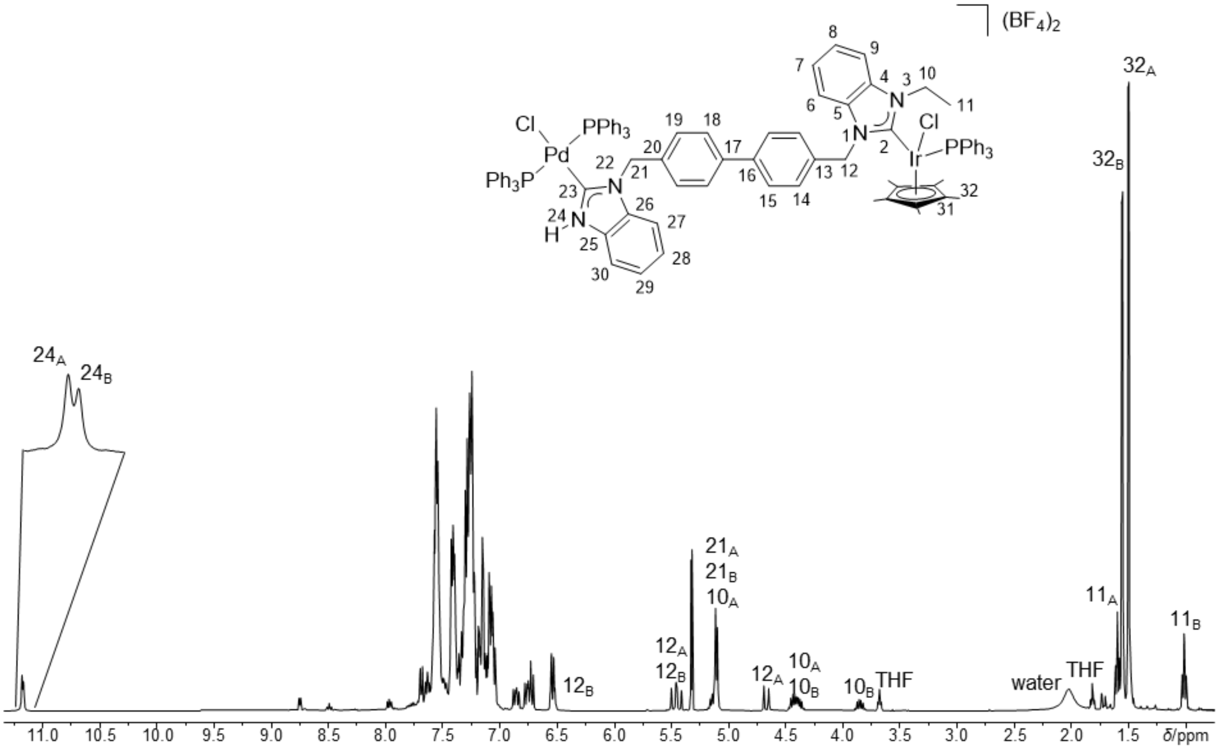

Figure S138. ^1H NMR spectrum of the mixture of atropisomers **A** and **B** of [**18**]$(BF_4)_2$ with only selected resonances assigned (400 MHz, CD_2Cl_2).

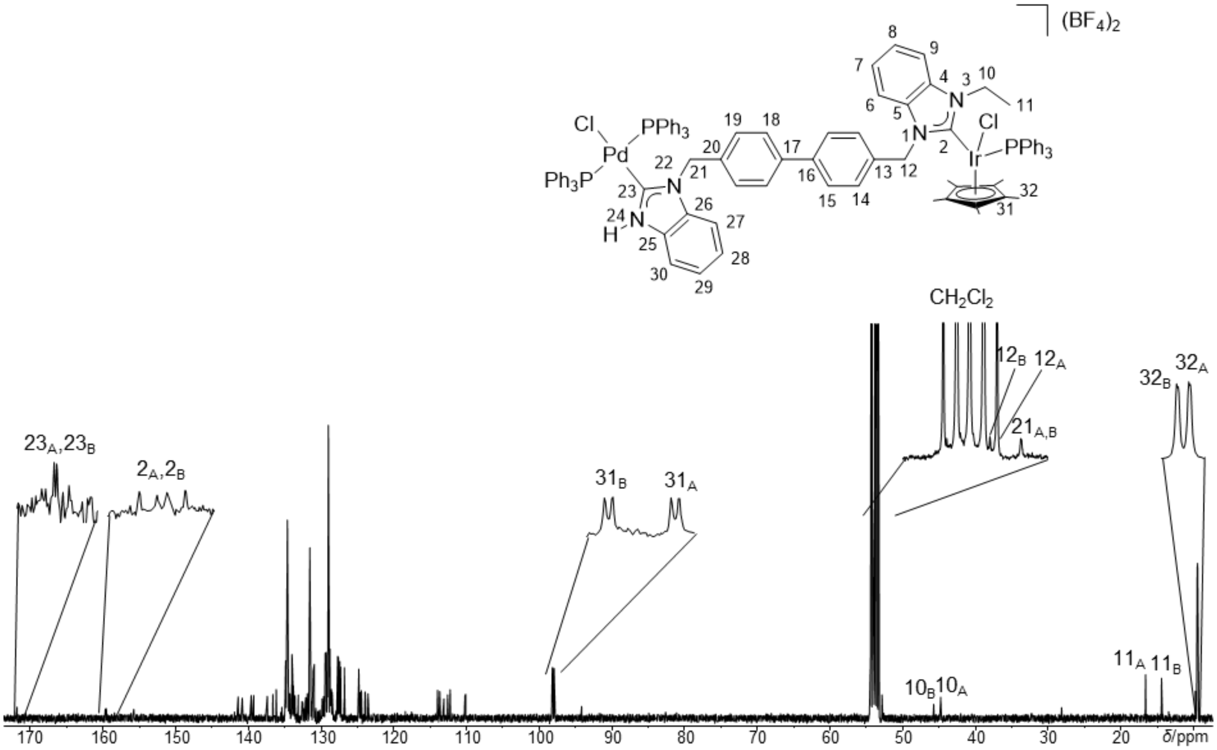

Figure S139. $^{13}C\{^1H\}$ NMR spectrum of the mixture of atropisomers **A** and **B** of [**18**]$(BF_4)_2$ with only selected resonances assigned (126 MHz, CD_2Cl_2).

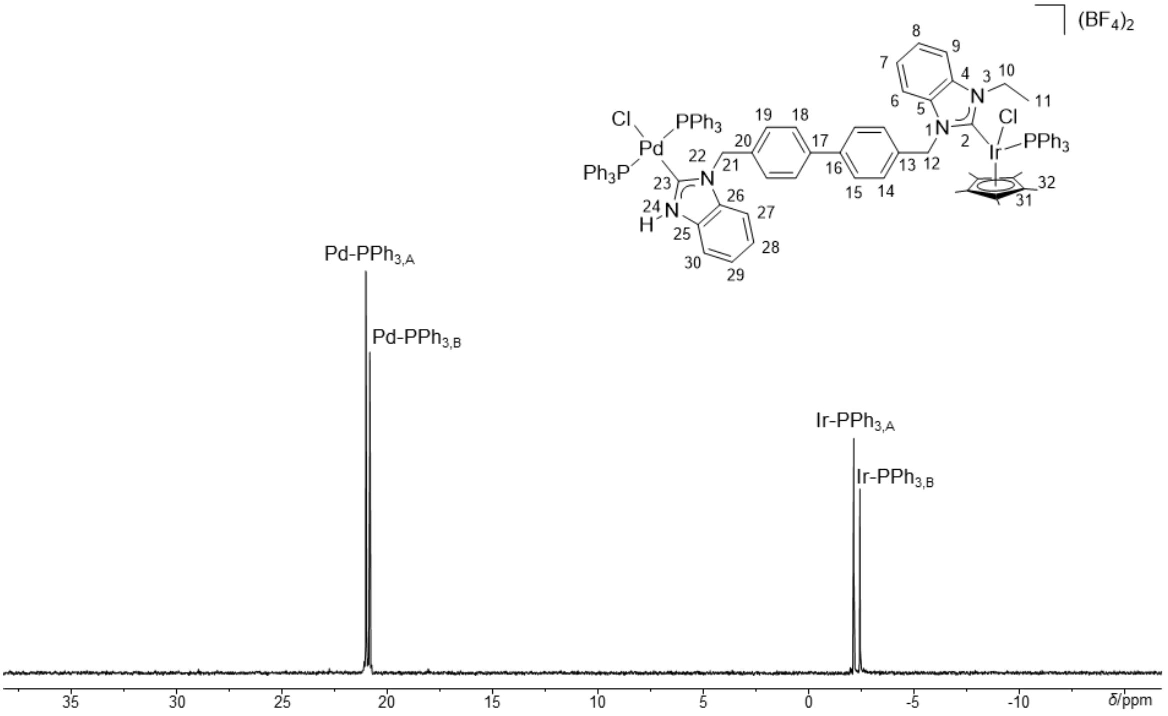

Figure S140. ^{31}P NMR spectrum of the mixture of atropisomers **A** and **B** of [**18**]$(BF_4)_2$ (162 MHz, CD_2Cl_2).

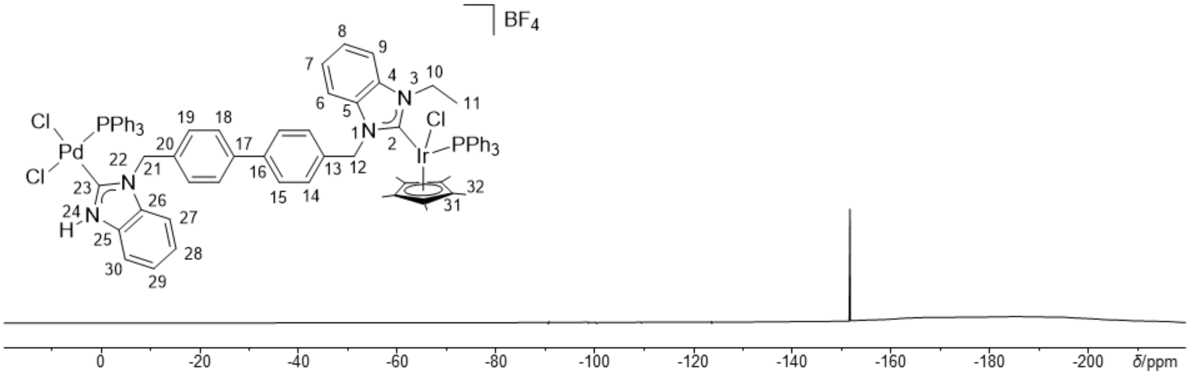

Figure S141. ^{19}F NMR spectrum of the mixture of atropisomers **A** and **B** of [**18**]$(BF_4)_2$ (376 MHz, CD_2Cl_2).

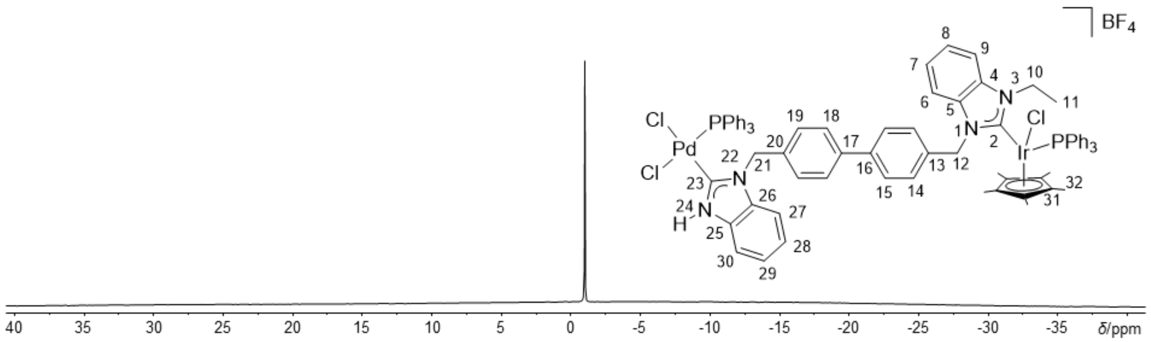

Figure S142. ^{11}B NMR spectrum of the mixture of atropisomers **A** and **B** of [**18**]$(BF_4)_2$ (128 MHz, CD_2Cl_2).

4. References

1. P. Mehlmann, C. Mück-Lichtenfeld, T. T. Y. Tan, F. Dielmann, *Chem. Eur. J.* **2017**, *23*, 5929–5933.
2. N. Kuhn, T. Kratz, *Synthesis* **1993,** 561–562.
3. R. A. Kunetskiy, I. Císařová, D. Šaman, I. M. Lyapkalo, *Chem. Eur. J.* **2009**, *15*, 9477–9485.
4. N. Kuhn, A. Abu-Rayyan, M. Göhner, M. Steinmann, *Z. Anorg. Allg. Chem.* **2002**, *628*, 1721–1723.
5. H. Shen, G. Deng, S. Kaappa, T. Tan, Y.-Z. Han, S. Malola, S.-C. Lin, B. K. Teo, H. Häkkinen, N. Zheng, *Angew. Chem.* **2019**, *131*, 17895–17899; *Angew. Chem. Int. Ed.* **2019**, *58*, 17731–17735.
6. Y. Luo, F. Xiao, S. Qian, W. Lu, B. Yang, *Eur. J. Med. Chem.* **2011**, *46*, 417–422.
7. L. F. B. Wilm, T. Eder, C. Mück-Lichtenfeld, P. Mehlmann, M. Wünsche, F. Buß, F. Dielmann, *Green Chem.* **2019**, *21*, 640–648.
8. M. Kitamura, M. Yano, N. Tashiro, S. Miyagawa, M. Sando, T. Okauchi, *Eur. J. Org. Chem.* **2011**, 458–462.
9. M. D. Böhme, L. F. B. Wilm, A. Hepp, F. E. Hahn, *Eur. J. Inorg. Chem.* **2021**, 1971–1975.
10. S. Lysenko, C. G. Daniliuc, P. G. Jones, M. Tamm, *J. Organomet. Chem.* **2013**, *744*, 7–14.
11. a) C. Lensink, S. K. Xi, L. M. Daniels, J. G. Verkade, *J. Am. Chem. Soc.* **1989**, *111*, 3478–3479; b) S. Ullrich, B. Kovačević, X. Xie, J. Sundermeyer, *Angew. Chem.* **2019**, *131*, 10443–10447; *Angew. Chem. Int. Ed.* **2019**, *58*, 10335–10339.
12. P. Mehlmann, T. Witteler, L. F. B. Wilm, F. Dielmann, *Nat. Chem.* **2019**, *11*, 1139–1143.
13. Ł. Kapuśniak, P. N. Plessow, D. Trzybiński, K. Woźniak, P. Hofmann, P. I. Jolly, *Organometallics* **2021**, *40*, 693–701.
14. J. Schraml, M. Čapka, V. Blechta, *Magn. Res. Chem.* **1992**, *30*, 544–547.
15. H. V. Huynh, T. T. Lam, H. T. T. Luong, *RSC Adv.* **2018**, *8*, 34960–34966.
16. L. Krause, R. Herbst-Irmer, G. M. Sheldrick, D. Stalke, *Appl. Crystallogr.* **2015**, *48*, 3–10.
17. R. H. Blessing, *Acta. Cryst.* **1995**, *A51*, 33–38.
18. G. M. Sheldrick, *Acta. Cryst.* **2015,** *A71*, 3–8.
19. G. M. Sheldrick, Acta. Cryst. **2015**, *C71*, 3–8.
